# Incidence, risk factors, and outcomes of contrast-associated acute kidney injury following interventional radiology procedures: a systematic review and meta-analysis

**DOI:** 10.3389/fradi.2026.1947256

**Published:** 2026-09-23

**Authors:** Saad Alhumaid, Mohammed Yousef Almulhim, Hessah Abdulaziz Al Hussaini, Qasem M. Alalwan, Hani Almohammed Ali, Sarah A. Al Ruzaihan, Fatima A. Aborshaid, Abdulrhman Khaled Al Abdulqader, Nourah Al Dossary, Muneera Alabdulqader, Shaden Saleh AlMousa, Khalid Suliman Aljoqiman

**Affiliations:** 1 Ministry of Health, Pharmacy Department, King Fahad Hofuf Hospital, Al-Ahsa Health Cluster, Al-Ahsa, Saudi Arabia; 2 Division of Nephrology, Department of Internal Medicine, King Faisal University, Al-Ahsa, Saudi Arabia; 3 Division of Endocrinology, Department of Internal Medicine, King Faisal University, Al-Ahsa, Saudi Arabia; 4 Radiology Department, King Fahad Hofuf Hospital, Al-Ahsa Health Cluster, Ministry of Health, Al-Ahsa, Saudi Arabia; 5 Department of Internal Medicine, College of Medicine, King Faisal University, Al-Ahsa, Saudi Arabia; 6 Department of Pediatric Allergy, Asthma and Clinical Immunology, Dr. Sulaiman Al-Habib Hospital, Al Khobar, Saudi Arabia; 7 Ministry of Health, General Surgery Department, King Fahad Hofuf Hospital, Al-Ahsa Health Cluster, Al-Ahsa, Saudi Arabia; 8 Pediatric Nephrology Specialty, Pediatric Department, Medical College, King Faisal University, Al-Ahsa, Saudi Arabia; 9 Division of Radiology, Department of General Surgery, College of Medicine, King Faisal University, Al-Ahsa, Saudi Arabia

**Keywords:** clinical outcomes, contrast-associated acute kidney injury, contrast-induced nephropathy, incidence, interventional radiology, iodinated contrast media, risk factors

## Abstract

**Background:**

Contrast-associated acute kidney injury (CA-AKI) is a recognized complication of iodinated contrast administration. Although extensively studied in coronary angiography, its incidence, associated factors, and outcomes following interventional radiology and other non-coronary endovascular procedures remain incompletely characterized. We evaluated the incidence, associated factors, and outcomes of CA-AKI in these populations.

**Methods:**

This systematic review followed PRISMA 2020 guidelines and a prospectively registered PROSPERO protocol (CRD420261385592). PubMed/MEDLINE, Embase, Scopus, CINAHL, and Web of Science were searched from inception to 30 April 2026. Eligible studies evaluated CA-AKI following catheter-based non-coronary endovascular or interventional radiology procedures. Random-effects meta-analysis estimated pooled incidence, with subgroup and sensitivity analyses.

**Results:**

Thirty-nine studies were included, of which 38 contributed to the meta-analysis. The pooled CA-AKI incidence was 6.91% (95% CI 5.69–8.24; I^2^ = 89.5%). In a sensitivity analysis restricted to patient-level estimates (30 studies; 16,825 unique patients), incidence was 7.27% (95% CI 5.77–8.92; I^2^ = 91.5%). Leave-one-out estimates ranged from 6.56% to 7.16%, indicating numerical stability despite substantial heterogeneity. Exploratory subgroup analysis showed higher pooled incidence following embolization/interventional oncology than neuroendovascular/carotid procedures (8.44% vs. 5.48%; *p* = 0.0171), although substantial residual heterogeneity renders this finding hypothesis-generating. Frequently reported associated factors included baseline renal dysfunction, advanced liver disease, diabetes mellitus, anaemia, hypoalbuminaemia, inflammatory burden, and greater contrast exposure. Heterogeneous observational evidence did not establish a clinically reliable contrast-volume threshold. Across individual studies, CA-AKI was associated with increased mortality, poorer functional outcomes, prolonged hospitalisation, and persistent renal dysfunction, although associations were not consistent across all studies and outcomes.

**Conclusion:**

CA-AKI occurs after a range of non-coronary endovascular and interventional radiology procedures and may be associated with clinically important adverse outcomes. Substantial heterogeneity limits the generalizability of a single pooled incidence estimate, and procedure- and population-specific estimates should remain central to interpretation. Standardised definitions and prospective multicentre studies are needed to establish procedure-specific risk profiles and clinically useful preventive strategies.

**Systematic Review Registration:**

https://www.crd.york.ac.uk/PROSPERO/view/CRD420261385592, PROSPERO, CRD420261385592.

## Background

Contrast-associated acute kidney injury (CA-AKI) is a clinically important complication that may occur following exposure to iodinated contrast media during diagnostic and interventional procedures. Traditionally referred to as contrast-induced nephropathy (CIN), CA-AKI is generally characterized by an acute deterioration in renal function occurring within hours to days after contrast administration in the absence of an alternative identifiable cause. Although the exact pathophysiology remains incompletely understood, proposed mechanisms include renal vasoconstriction, medullary hypoxia, oxidative stress, endothelial dysfunction, tubular toxicity, and inflammatory activation (1–4). The risk of CA-AKI is influenced by multiple patient-related and procedural factors, including baseline renal impairment, diabetes mellitus, advanced age, hemodynamic instability, cumulative contrast exposure, and comorbid systemic disease (1–4).

Over recent decades, interventional radiology and related non-coronary endovascular procedures have expanded substantially across a wide range of clinical settings, including neuroendovascular thrombectomy, transarterial chemoembolization (TACE), embolization procedures, carotid artery stenting, and image-guided vascular interventions (5–43). Many of these procedures require repeated or high-volume administration of iodinated contrast media, often in medically complex populations with multiple risk factors for renal dysfunction (5–43). Patients undergoing TACE or hepatic embolization, for example, frequently have cirrhosis, hepatocellular carcinoma, ascites, hypoalbuminaemia, or pre-existing renal impairment (5, 9–12, 18, 22, 24, 26, 32–43), whereas neuroendovascular stroke populations may present with severe systemic inflammation, hemodynamic instability, or critical neurological illness (6–8, 13–17, 19–21, 23, 25, 27–31, 38, 39).

Despite increasing procedural complexity and contrast exposure in contemporary interventional radiology practice, the reported incidence of CA-AKI following non-coronary endovascular procedures remains highly variable across the literature (5–43). Differences in patient populations, procedural characteristics, contrast protocols, and diagnostic definitions contribute substantially to this variability (5–43). Furthermore, while some studies suggest that CA-AKI may be associated with adverse outcomes such as prolonged hospitalisation, dialysis requirement, poor functional recovery, and increased mortality (5–8, 11, 18, 25–28, 32–36, 39, 42), the overall clinical impact of CA-AKI in interventional radiology populations remains incompletely characterized.

Most previous literature evaluating contrast-associated renal injury has focused primarily on coronary angiography and interventional cardiology populations (1, 2), whereas evidence specific to interventional radiology and related endovascular procedures remains comparatively fragmented (5–43). In addition, evolving terminology and diagnostic frameworks, including the transition from traditional CIN definitions toward broader CA-AKI and Kidney Disease: Improving Global Outcomes-based classifications, further complicate interpretation and comparison across studies (1–4, 6, 13, 14, 16, 23, 28, 35).

Accordingly, this systematic review and meta-analysis aimed to evaluate the incidence, risk factors, and clinical outcomes associated with contrast-associated acute kidney injury following interventional radiology and related non-coronary endovascular procedures. Secondary objectives included exploring heterogeneity according to procedural category, baseline renal function, and study-level diagnostic definitions of CA-AKI.

## Methods

### Study design and reporting

This systematic review was conducted in accordance with the PRISMA (Preferred Reporting Items for Systematic Reviews and Meta-Analyses) 2020 guideline (44). The protocol was developed *a priori* and registered in the PROSPERO database (registration number: CRD420261385592).

### Eligibility criteria (PICOS framework)

Eligibility criteria were defined *a priori* using the PICOS (Population, Intervention/Exposure, Comparator, Outcomes, Study design) framework, as summarized in Table 1 (45).

**Table 1:** PICOS eligibility criteria.

Component	Description
Population	Adult or pediatric patients undergoing catheter-based non-coronary endovascular or interventional radiology procedures involving iodinated contrast media
Intervention	Administration of contrast agents during interventional radiology procedures
Comparator	Not required; studies with or without comparator groups were eligible
Outcomes	Incidence of contrast-associated acute kidney injury (CA-AKI), risk factors, and clinical outcomes (e.g., mortality, hospitalisation, renal recovery). Studies using the term contrast-induced nephropathy (CIN) were included and considered within the broader definition of CA-AKI
Study design	Observational studies (cohort, case–control, cross-sectional) and randomised controlled trials

In brief, studies were included if they enrolled adult or pediatric patients undergoing catheter-based non-coronary endovascular or interventional radiology procedures involving iodinated contrast media and systematically evaluated CA-AKI, CIN, or acute kidney injury (AKI) as a primary or clinically meaningful secondary outcome. Observational studies and randomised controlled trials were eligible.

Eligible procedures included fluoroscopy-guided arterial or venous endovascular interventions commonly performed within interventional radiology or related non-coronary endovascular practice. Eligibility decisions prioritized fluoroscopy-guided catheter-based procedures performed within interventional radiology or closely related non-coronary endovascular specialties, including neurointerventional procedures, while excluding predominantly coronary and cardiovascular surgical/endovascular cohorts. Purely diagnostic angiography without intervention and procedures performed exclusively within interventional cardiology were excluded.

The primary contrast exposure of interest was intra-arterial administration of iodinated contrast during catheter-based endovascular or interventional radiology procedures. Studies in which patients also received intravenous iodinated contrast as part of the same diagnostic–therapeutic pathway [e.g., preprocedural computed tomography angiography [CTA] or computed tomography perfusion [CTP] followed by catheter angiography or endovascular intervention] were eligible when renal outcomes were reported for the overall procedural pathway. Where intravenous and intra-arterial exposures could not be separately attributed to the reported CA-AKI outcome, the exposure was considered mixed-route and was not interpreted as reflecting the renal effect of intra-arterial contrast alone.

For mixed procedural cohorts, eligibility was determined according to whether the study population predominantly represented the predefined non-coronary endovascular/interventional radiology setting and whether the reported renal outcome was assessed in relation to that procedural pathway. No quantitative threshold defining the proportion of eligible procedures within mixed cohorts was prespecified.

Studies were excluded if they were case reports or small case series (<5 patients), reviews, editorials, conference abstracts without full data, non-English publications, coronary angiography or percutaneous coronary intervention studies, or did not systematically evaluate CA-AKI, CIN, or AKI outcomes.

The PRISMA 2020 checklist was followed and is provided in the Supplementary Materials (44).

### Data sources and search strategy

A comprehensive literature search was performed across PubMed/MEDLINE, Embase, Scopus, CINAHL, and Web of Science Core Collection, from database inception to 30 April 2026.

The search strategy combined Medical Subject Headings (MeSH) and free-text terms related to acute kidney injury and contrast-associated nephropathy with endovascular and interventional radiology procedures. To improve specificity, selected procedure-related terms (e.g., angiography and embolization) were combined with contrast-related keywords. Free-text terms were restricted to the title and abstract fields. Boolean operators (AND, OR) were applied to optimize retrieval.

For PubMed, a combination of Medical Subject Headings (MeSH) and free-text terms was used, with free-text terms searched within titles and abstracts to optimize retrieval of relevant studies.

For Scopus, the search was conducted using the TITLE-ABS-KEY field to capture records indexed by title, abstract, and keywords. To improve specificity, selected procedural terms (e.g., angiography) were combined with contrast-related keywords to reduce retrieval of non-relevant studies.

For Embase, both Emtree terms and free-text keywords were used. Controlled vocabulary terms were exploded to include all relevant subheadings, and free-text terms were searched within titles and abstracts.

For Web of Science, topic searches (TS) were used to capture terms within titles, abstracts, and keywords.

For CINAHL, keyword-based searches were performed to capture relevant records indexed within titles, abstracts, and subject headings.

Reference lists of included studies and relevant reviews were also screened to identify additional eligible studies. Full database-specific search strategies are provided in Supplementary Table S1.

### Study selection

All retrieved records were imported into EndNote (version 21.2) for deduplication and subsequently uploaded to Covidence (Veritas Health Innovation, Melbourne, Australia) for screening.

Two reviewers independently screened titles and abstracts for eligibility. Full-text articles were then assessed against the predefined inclusion criteria. Eligibility decisions were based on the predefined operational criteria for catheter-based non-coronary endovascular and interventional radiology procedures. Disagreements were resolved through discussion, with arbitration by a third reviewer where necessary.

Reasons for exclusion at the full-text stage were recorded and are presented in Supplementary Table S2.

The study selection process is summarized in a PRISMA flow diagram (Figure 1).

**Figure 1:**
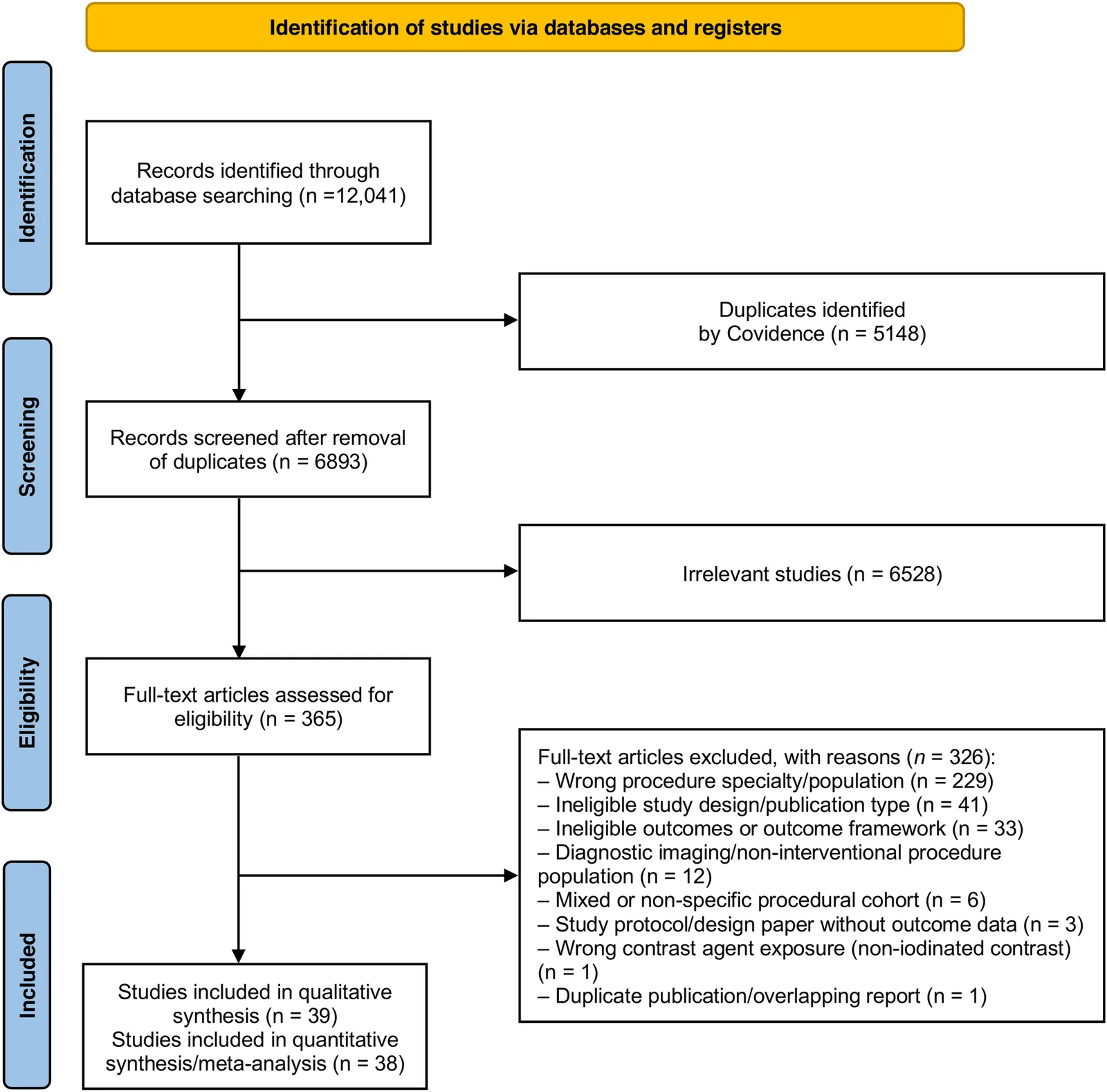
Flow diagram of studies included in the systematic review.

### Data extraction

Extracted variables included study characteristics (author, year, country, and study design), population characteristics (sample size, age, and comorbidities), procedure type, contrast exposure details, including route of administration and contrast-media type where reported, periprocedural hydration or renal-protective strategies, periprocedural hemodynamic factors including hypotension where reported, definitions of CA-AKI, incidence of CA-AKI, reported risk factors, and clinical outcomes such as mortality, hospitalisation, dialysis requirement, and renal recovery.

Where studies reported multiple definitions of CA-AKI, the original study definitions were retained. Studies using alternative terminology, including CIN, were included and harmonized under the broader CA-AKI framework where appropriate. Definitions were extracted as reported by the original studies and varied in both creatinine thresholds and ascertainment windows, ranging from approximately 24 h to 7 days after contrast exposure. Given the evolution of terminology and diagnostic criteria over time, studies were eligible for quantitative incidence synthesis when they reported a discrete post-procedural AKI, CIN, or CA-AKI event count and corresponding denominator, even when the diagnostic threshold or ascertainment window differed from contemporary criteria. Study-specific definitions and assessment windows were retained as reported rather than retrospectively reclassified.

### Risk of bias assessment

The methodological quality of observational studies was assessed using the Newcastle–Ottawa Scale (NOS) (46), evaluating the Selection, Comparability, and Outcome domains. Total NOS scores ranged from 0 to 9; for interpretive purposes, scores of 7–9 were classified as low risk of bias, 4–6 as moderate risk of bias, and 0–3 as high risk of bias. Risk-of-bias assessment was conducted independently by two reviewers, with disagreements resolved by consensus. The Cochrane Risk of Bias 2 (RoB 2) tool was prespecified for randomised controlled trials; however, no eligible randomised controlled trials were identified, and therefore RoB 2 was not applied (47).

### Certainty of evidence assessment

The certainty of evidence for the principal quantitative CA-AKI incidence estimates was assessed using the Grading of Recommendations Assessment, Development and Evaluation (GRADE) framework (48). Certainty was evaluated across the domains of risk of bias, inconsistency, indirectness, imprecision, and publication bias and categorized as high, moderate, low, or very low. Given that all included studies contributing to the incidence synthesis were observational, the initial certainty was considered low and was downgraded where important concerns were identified. GRADE assessments and the rationale for each judgment are presented in Supplementary Table S8.

### Data synthesis

Given the substantial clinical and methodological heterogeneity across studies, a hybrid synthesis approach was undertaken. A meta-analysis was performed for the incidence of CA-AKI, where appropriate, while risk factors and clinical outcomes were synthesized narratively. Risk factors and clinical outcomes were not quantitatively pooled because of substantial heterogeneity in exposure definitions, outcome ascertainment, effect measures, and adjustment strategies across studies. Where substantial clinical heterogeneity existed, findings were synthesized narratively rather than quantitatively pooled.

Incidence was extracted according to the unit of analysis reported in each original study and was defined as the proportion of patients, procedures, or treatment sessions complicated by CA-AKI. The denominator therefore represented unique patients for patient-level studies and procedures or treatment sessions for procedure/session-level studies. Because procedure/session-level studies could include repeated observations from the same patient, the unit of analysis was recorded for each study and considered when interpreting the pooled estimates.

For quantitative incidence synthesis, studies were required to report a discrete study-defined AKI, CIN, or CA-AKI event count with a corresponding denominator. A formal creatinine-based threshold was not required if the original study explicitly reported AKI as a discrete post-procedural clinical outcome. Studies reporting only continuous changes or trajectories in renal-function measures, without a discrete AKI/CIN/CA-AKI outcome suitable for incidence estimation, were retained in the qualitative synthesis but excluded from the primary incidence meta-analysis.

For the meta-analysis, pooled incidence estimates were calculated using a random-effects model. The primary effect measure was the pooled incidence proportion with 95% confidence intervals. Proportions were transformed using the Freeman–Tukey double arcsine method to stabilize variance. Statistical heterogeneity was assessed using the I^2^ statistic, with values >50% indicating substantial heterogeneity. Analyses were conducted using R version 4.1.0 (R Foundation for Statistical Computing, Vienna, Austria), with the *meta*, *finalfit*, and *forestplot* packages.

To address differences in unit of analysis, an additional sensitivity analysis was performed using patient-level incidence estimates only. Studies for which the primary incidence denominator represented procedures or treatment sessions were excluded unless the original report also provided an extractable patient-level incidence estimate; where available, that patient-level estimate was used. This sensitivity analysis included 30 studies representing 16,825 unique patients.

Potential cohort overlap was assessed based on study setting, recruitment periods, study populations, and author groups. Where distinct reports originated from the same institution and patient overlap could not be excluded, this was considered in sensitivity analyses and interpretation of the pooled estimates. Because one study reported AKI as a discrete post-procedural adverse event without specifying a formal creatinine-based diagnostic threshold (40), an additional sensitivity analysis was performed excluding this study to assess the robustness of the pooled incidence estimate to incomplete outcome-definition reporting.

To assess the influence of individual studies on the overall pooled incidence estimate, a leave-one-out sensitivity analysis was performed by sequentially excluding each study and recalculating the random-effects pooled estimate. Particular attention was given to Schwarz et al. (15), the largest included cohort (*n* = 6,638), to assess whether its substantially larger sample size disproportionately influenced the overall estimate.

Subgroup analyses were planned based on clinically relevant study-level factors, including type of interventional radiology procedure, baseline renal function and chronic kidney disease (CKD) status, and study-level definitions of CA-AKI. Differences in diagnostic criteria and ascertainment timing were considered potential sources of clinical and methodological heterogeneity. Heterogeneity related to diagnostic criteria was explored through subgroup analysis according to study-level CA-AKI definition.

### Publication bias

Small-study effects were assessed for the primary incidence meta-analysis through visual inspection of a funnel plot and Egger's linear regression test of funnel-plot asymmetry. Because funnel-plot asymmetry in meta-analyses of proportions may arise from substantial between-study heterogeneity, variation in underlying incidence, study size, or methodological characteristics rather than selective publication alone, these findings were interpreted cautiously and were not considered definitive evidence of publication bias.

## Results

### Study selection

The database search identified 12,041 records. After removal of 5,148 duplicates, 6,893 records underwent title and abstract screening. Of these, 6,528 records were excluded during initial screening, and 365 full-text articles were assessed for eligibility. A total of 326 full-text articles were excluded for predefined reasons, most commonly because they involved interventional cardiology, vascular surgery, or non-interventional imaging populations rather than interventional radiology procedures. Thirty-nine studies were included in the qualitative synthesis (5–43), of which 38 studies were eligible for quantitative meta-analysis of pooled incidence (5–23, 25–43). The unit of analysis varied across studies, with most reporting patient-level incidence and several reporting incidence per procedure or treatment session. One study was excluded from the primary quantitative incidence synthesis because it evaluated serial renal-function deterioration and creatinine/CG-GFR trajectories without reporting a discrete study-defined AKI, CIN, or CA-AKI outcome comparable with the binary incidence outcomes used in the meta-analysis (24). The study selection process is summarized in Figure 1, with detailed reasons for exclusion provided in Supplementary Table S2.

### Study characteristics

The included studies were published between 2004 and 2026 and predominantly consisted of retrospective or prospective observational cohort studies. Most studies were conducted in East Asia, particularly Japan, South Korea, China, and Taiwan, with additional studies originating from Europe, the United States, and multinational cohorts. Included procedural categories primarily involved TACE, neuroendovascular thrombectomy or coiling procedures, bronchial artery embolization, hepatic artery embolization, and cerebral angiography/endovascular interventions. Study sizes varied considerably, with analytic denominators reported as patients, procedures, or treatment sessions according to the original study design. Definitions of CA-AKI varied across studies and included Kidney Disease: Improving Global Outcomes (KDIGO), Acute Kidney Injury Network (AKIN), International Club of Ascites (ICA), European Society of Urogenital Radiology (ESUR), and study-specific CIN criteria. No randomised controlled trials met the eligibility criteria; all included studies were observational in design. Detailed study characteristics are presented in Supplementary Table S3.

Contrast administration during the catheter-based procedures was predominantly intra-arterial. However, several neurovascular studies evaluated diagnostic–therapeutic pathways involving intravenous contrast-enhanced imaging, including CTA and/or CTP, before subsequent intra-arterial catheter angiography or endovascular intervention. In these studies, the contributions of intravenous and intra-arterial contrast exposure to the reported CA-AKI outcome could not always be separately determined.

Contrast-media characteristics also varied across studies, with low-osmolar, iso-osmolar, and mixed-agent use reported, while some studies did not provide sufficient detail regarding contrast-media type (Supplementary Table S3). Reporting of other clinically relevant procedural factors was limited. Periprocedural hydration strategies and hemodynamic factors, including procedural hypotension, were not consistently reported across the included studies. Where reported, Prasad et al. indicated that all patients received procedural fluids, although hydration volume was not quantified, while Schwarz et al. reported peri-EVT intravenous hydration and procedural hypotension (15, 17). Given the heterogeneity in contrast-media characteristics and the sparse and inconsistent reporting of hydration and hemodynamic factors, reliable subgroup analyses according to these procedural characteristics were not considered feasible. These factors were therefore considered potential sources of residual clinical heterogeneity rather than suitable variables for quantitative synthesis.

Although both adult and pediatric populations were eligible *a priori*, no pediatric studies met all eligibility criteria; therefore, the included evidence was derived exclusively from adult populations.

### Incidence of contrast-associated acute kidney injury (CA-AKI)

Across 38 studies included in the quantitative synthesis, the pooled incidence of CA-AKI following interventional radiology procedures was 6.91% (95% CI 5.69–8.24). Substantial between-study heterogeneity was observed (I^2^ = 89.5%). Incidence estimates varied considerably across studies, ranging from 0% in selected low-risk embolization cohorts to 23.8% in high-risk hepatocellular carcinoma populations undergoing repeated TACE procedures. Higher incidences were generally observed in studies involving advanced liver disease, repeated TACE exposure, CKD, or severe baseline renal impairment. The pooled incidence forest plot is shown in Figure 2, while individual study-level incidence estimates are summarized in Table 2. Additional subgroup-, severity-specific, and timepoint-specific incidence findings are presented in Supplementary Table S7.

**Figure 2:**
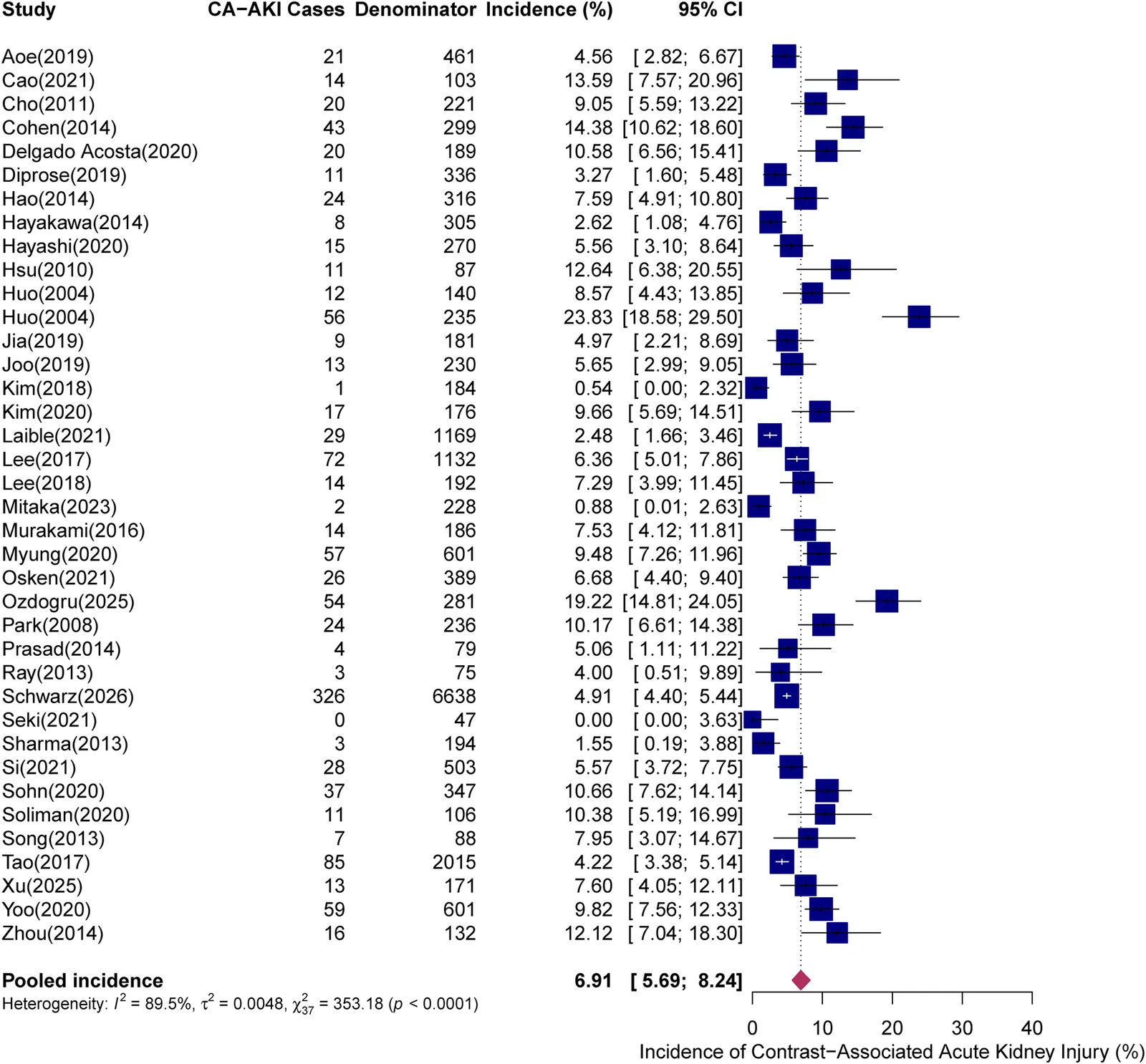
Forest plot showing pooled incidence of contrast-associated acute kidney injury following interventional radiology procedures. Study denominators represent patients, procedures, or treatment sessions according to the unit of analysis reported in the original study.

**Table 2:** Incidence of contrast-associated acute kidney injury across included studies.

Study (Author, Year)	Sample size (n)	CA-AKI Cases (n)	Incidence (%)	Definition of CA-AKI	Timing of AKI Assessment
Aoe (2019) (43)	461 TACE sessions (260 patients)	21	4.6%	CIN defined as increase in serum creatinine ≥0.5 mg/dL or ≥25% from baseline	24–72 h after TACE
Cao (2021) (42)	103 patients	14	13.6%	CIN defined as increase in serum creatinine by ≥0.5 mg/dL within 48–72 h after contrast administration	48–72 h after TACE
Cho (2011) (41)	221 TACE treatment sessions (101 patients)	20	9.0%	RCIN defined as increase in serum creatinine ≥25% from baseline	48–96 h after TACE
Cohen (2014) (40)	299 patients with available AKI data	43^a^	14.4%^a^	AKI reported as post-TACE complication; no formal creatinine-based definition reported	Post-TACE follow-up; exact timing not reported
Delgado Acosta (2020) (39)	189 patients	20	10.6%	CIN defined as increase in serum creatinine >25% from baseline or absolute increase ≥0.50 mg/dL after intravascular contrast administration	Typically 48–72 h after contrast exposure during hospitalisation
Diprose (2019) (38)	336 patients	11	3.3%	CA-AKI defined as increase in serum creatinine ≥26.5 µmol/L or ≥1.5 × baseline serum creatinine following contrast exposure	24–72 h after EVT
Hao (2014) (37)	316 TACE procedures in 166 patients	24	7.6%	AKIN criteria: absolute increase ≥26.4 µmol/L (0.3 mg/dL), ≥50% relative increase in serum creatinine, or requirement for postoperative haemodialysis	Within 72 h after TACE
Hayakawa (2014) (36)	305 TACE sessions in 141 patients	8	2.6%	CIN defined as increase in serum creatinine >0.5 mg/dL or >25% from baseline within 3 days after TACE without alternative cause of AKI	Postoperative day 2–3
Hayashi (2020) (35)	270 platinum-based TACE/TAI sessions in 129 patients	15	5.6%	ICA cirrhosis-specific AKI criteria: increase in serum creatinine ≥0.3 mg/dL or ≥50% from baseline within 7 days after TACE/TAI	Days 1, 5, and 30 after TACE/TAI
Hsu (2010) (34)	87 HCC patients with ascites undergoing first TACE session	11	12.6%	ARF/CA-AKI defined as increase in serum creatinine ≥50% from baseline or absolute increase ≥0.5 mg/dL to >1.5 mg/dL within 7 days after TACE	Within 7 days after TACE
Huo (2004) (33)	140 TACE treatment sessions in 140 patients	12	8.6%	ARF defined as increase in serum creatinine ≥50% from baseline or absolute increase ≥0.5 mg/dL to >1.5 mg/dL within 7 days after TACE	Days 3, 6, and 9 after TACE
Huo (2004) (32)	235 patients undergoing 843 TACE sessions	56	23.8%	ARF defined as increase in serum creatinine ≥50% from baseline or absolute increase ≥0.5 mg/dL to >1.5 mg/dL within 7 days after TACE	Within 7 days after TACE
Jia (2019) (31)	181 patients with AIS	9	5.0%	AKI defined as ≥25% increase in serum creatinine from baseline at 48 h	48 h after CTA
Joo (2019) (30)	230 patients with unruptured cerebral aneurysms undergoing elective coiling	13	5.6%	CIN defined as absolute increase in serum creatinine ≥0.5 mg/dL or relative increase ≥25% from baseline serum creatinine value	48–72 h after contrast exposure
Kim (2018) (29)	184 patients with CKD undergoing neuroendovascular procedures	1	0.5%	CIN defined as absolute increase in serum creatinine ≥0.5 mg/dL or ≥25% rise from baseline after excluding other causes of renal impairment	48–72 h after contrast exposure
Kim (2020) (28)	176 patients undergoing elective neurointervention procedures	17	9.5%	CIN defined as increase in serum creatinine >0.5 mg/dL or ≥25% from baseline in the absence of an alternative cause	Within 48 h after contrast exposure
Laible (2021) (27)	1,169 patients undergoing EVT for AIS	29	2.5%	PC-AKI defined as increase in serum creatinine ≥0.5 mg/dL or >25% above baseline creatinine value	Within 48 h after admission creatinine measurement
Lee (2017) (26)	1,132 HCC patients undergoing cTACE	72	6.4%	AKI identified using ICD-9-CM diagnostic code 584 during follow-up	During longitudinal follow-up after cTACE
Lee (2018) (25)	192 patients with aSAH undergoing coil embolization	14	7.3%	CI-AKI defined as increase in serum creatinine >25% or >0.5 mg/dL from baseline	Within 72 h after coil embolization
Mitaka (2023) (23)	228 patients undergoing elective neuroendovascular surgery	2	0.9%	Clinical CA-AKI defined using KDIGO criteria: increase in serum creatinine ≥0.3 mg/dL within 48 h, ≥1.5 × baseline serum creatinine, or urine output <0.5 mL/kg/h for 6 h	POD 0–3 after neuroendovascular surgery
Murakami (2016) (22)	186 TACE sessions in 122 patients	14	7.5%	CIN defined as increase in serum creatinine ≥0.5 mg/dL or ≥25% from baseline	48–72 h after TACE
Myung (2020) (20)	601 patients undergoing sequential CTA and cerebral angiography	57	9.5%	PC-AKI defined as absolute increase in serum creatinine ≥0.5 mg/dL or relative increase ≥25% from baseline	Within 72 h after contrast exposure
Ösken (2021) (21)	389 patients undergoing carotid artery stenting	26	6.6%	CI-AKI defined as increase in serum creatinine ≥0.3 mg/dL or ≥50% from baseline according to AKIN criteria	Within 48–72 h after contrast exposure
Ozdogru (2025) (19)	281 patients undergoing EVT/MT for AIS	54	19.0%	CI-AKI defined as increase in serum creatinine ≥0.5 mg/dL or >25% within 48–72 h after EVT compared with baseline	Serum creatinine assessed at baseline and 48–72 h after EVT
Park (2008) (18)	236 patients undergoing TACE	24	10.2%	AKI defined according to AKIN criteria as absolute increase in serum creatinine ≥0.3 mg/dL or ≥50% from baseline within 48 h after TACE	Within first 48 h after TACE
Prasad (2014) (17)	79 neuroendovascular procedures with contrast media dose ≥250 mL	4	5.0%	CIN defined as increase in serum creatinine ≥50% above baseline or absolute increase ≥0.3 mg/dL at either 24 or 48 h after procedure	24 and 48 h after neuroendovascular procedure
Ray (2013) (16)	75 patients with SAH undergoing at least one contrast-enhanced imaging study	3	4.0%	CIN defined according to KDIGO criteria as serum creatinine increase >1.5 × baseline or >0.3 mg/dL above baseline, or urine output <0.5 mL/kg/h during one 6-h block	Serum creatinine followed up to 72 h after contrast study
Schwarz (2026) (15)	6,638 patients undergoing EVT for AIS	326	4.9%	CA-AKI defined according to KDIGO criteria as increase in serum creatinine ≥0.3 mg/dL or ≥1.5 × baseline within 48 h after contrast administration	Within 48 h after EVT contrast exposure
Seki (2021) (14)	47 patients undergoing emergency TAE for uncontrollable postpartum haemorrhage	0	0%	PC-AKI defined according to ESUR Guidelines v10.0 as increase in serum creatinine >0.3 mg/dL or >1.5 × baseline within 48–72 h after intravascular contrast administration	Serum creatinine assessed 48–72 h after TAE and before discharge
Sharma (2013) (13)	194 patients undergoing emergency endovascular treatment for AIS	3	1.5%	CIN defined according to AKIN criteria as relative increase in serum creatinine ≥50% above baseline or absolute increase ≥0.3 mg/dL	Within 48 h after endovascular procedure
Si (2021) (12)	503 consecutive TACE sessions in 284 patients with HCC	28	5.6%	CIN defined as increase in serum creatinine ≥0.5 mg/dL (50 µmol/L) or ≥25% from baseline within 48–72 h after intravascular contrast administration in absence of alternative causes	Serum creatinine assessed 48–96 h after TACE
Sohn (2020) (11)	347 HCC patients undergoing TACE	37	11.0%	ICA-AKI criteria: increase in serum creatinine ≥0.3 mg/dL within 48 h or ≥50% increase from baseline within prior 7 days	AKI assessed at 1 day, 2 months, and 4 months after TACE
Soliman (2020) (10)	106 HAE procedures in 84 patients	11	10.3%	CIN defined as 25% increase above baseline serum creatinine or absolute increase ≥0.5 mg/dL within 72 h post-embolization	Within 72 h after hepatic artery embolization
Song (2013) (9)	88 patients undergoing bronchial arteriography or BAE	7	8.0%	CIN defined as increase in serum creatinine >25% from baseline or absolute increase ≥0.5 mg/dL	Between 48 and 96 h after bronchial arteriography or BAE
Tao (2017) (8)	2015 patients with stroke undergoing cerebral angiography ± endovascular therapy	85	4.2%	CI-AKI defined according to ESUR criteria as increase in serum creatinine by ≥44 μmol/L or ≥25% from baseline within 48 h after contrast exposure in absence of alternative etiology	Within 48 h after cerebral angiography
Xu (2025) (7)	171 patients with haemoptysis undergoing sequential CTA and BAE	13	7.6%	CA-AKI defined as increase in serum creatinine ≥26.5 μmol/L or ≥1.5 × baseline serum creatinine after contrast exposure	24–72 h after BAE
Yoo (2020) (6)	601 patients undergoing EVT for AIS	59	9.8%	AKI defined according to KDIGO criteria as increase in serum creatinine ≥0.3 mg/dL within 48 h or ≥1.5 × baseline serum creatinine within 7 days	Follow-up creatinine assessed from next day to 7 days after EVT
Zhou (2014) (5)	132 patients with HCC undergoing TACE	16	12.1%	AKI defined according to AKIN criteria as increase in serum creatinine ≥0.3 mg/dL or ≥50% from baseline within 48 h after TACE	Serum creatinine assessed within 48 h after TACE

Incidence (%) was calculated using the unit of analysis reported by each original study, with CA-AKI events divided by the corresponding number of patients, procedures, or treatment sessions. Some studies reported patient-level incidence, whereas others reported procedure- or treatment-session-level incidence. Where available, both the number of procedures/sessions and the number of unique patients are presented.

AIS, acute ischemic stroke; AKI, acute kidney injury; AKIN, Acute Kidney Injury Network; ARF, acute renal failure; aSAH, aneurysmal subarachnoid haemorrhage; BAE, bronchial artery embolization; CA-AKI, contrast-associated acute kidney injury; CI-AKI, contrast-induced acute kidney injury; CIN, contrast-induced nephropathy; CKD, chronic kidney disease; cTACE, conventional transarterial chemoembolization; CTA, computed tomography angiography; ESUR, European Society of Urogenital Radiology; EVT, endovascular thrombectomy; h, hour; HAE, hepatic artery embolization; HCC, hepatocellular carcinoma; ICA, International Club of Ascites; ICD-9-CM, International Classification of Diseases, Ninth Revision, Clinical Modification; KDIGO, Kidney Disease: Improving Global Outcomes; MT, mechanical thrombectomy; PC-AKI, post-contrast acute kidney injury; POD, postoperative day; RCIN, radiocontrast-induced nephropathy; SAH, subarachnoid haemorrhage; TACE, transcatheter arterial chemoembolization; TAE, transcatheter arterial embolization; TAI, transarterial infusion chemotherapy.

a Acute kidney injury incidence derived from 43/299 patients in the Israeli and Chinese cohorts with available adverse-event data. Although a formal creatinine-based diagnostic threshold was not reported, AKI was explicitly reported as a discrete post-TACE adverse event and was therefore eligible for the primary incidence synthesis.

Sensitivity analysis restricted to patient-level estimates included 30 studies (16,825 unique patients). The pooled CA-AKI incidence was 7.27% (95% CI 5.77–8.92), with substantial heterogeneity (I^2^ = 91.5%). This estimate was similar to the primary mixed-unit estimate of 6.91% (95% CI 5.69–8.24), indicating that the overall pooled incidence remained similar after restriction to patient-level data (Supplementary Figure S4).

In a further sensitivity analysis addressing potential cohort overlap, Huo et al. (32) was retained while Huo et al. (33) and Hsu et al. (34) were excluded. Across the remaining 36 studies, the pooled CA-AKI incidence was 6.77% (95% CI 5.53–8.12; I^2^ = 89.9%), which was similar to the primary pooled estimate of 6.91%, indicating that potential cohort overlap did not materially influence the overall incidence estimate (Supplementary Figure S5).

In an additional sensitivity analysis excluding Cohen et al. (40), in which AKI was reported as a discrete post-TACE adverse event without a formal creatinine-based diagnostic threshold, the pooled incidence across the remaining 37 studies was 6.73% (95% CI 5.53–8.03; I^2^ = 89.0%). This was similar to the primary pooled estimate of 6.91% (95% CI 5.69–8.24; I^2^ = 89.5%), indicating that inclusion of this study did not materially influence the overall incidence estimate (Supplementary Figure S6).

Leave-one-out sensitivity analysis demonstrated that the pooled CA-AKI incidence remained numerically stable after sequential exclusion of individual studies, ranging from 6.56% to 7.16%. Exclusion of Schwarz et al. (15), the largest included cohort (*n* = 6,638), yielded a pooled incidence of 6.99% (95% CI 5.60–8.52; I^2^ = 89.3%), compared with 6.91% (95% CI 5.69–8.24; I^2^ = 89.5%) in the primary analysis. No individual study materially altered the overall summary estimate; however, substantial heterogeneity persisted across all leave-one-out analyses (I^2^ range, 87.4–89.8%) (Supplementary Figure S7).

Visual inspection of the funnel plot suggested asymmetry, and Egger's linear regression test identified statistically significant funnel-plot asymmetry (t = 2.35, df = 36, *p* = 0.0246), indicating the presence of small-study effects (Supplementary Figure S8). However, given the substantial between-study heterogeneity in the primary analysis (I^2^ = 89.5%), this finding was not interpreted as definitive evidence of publication bias.

### Subgroup analyses

In exploratory subgroup analysis, pooled CA-AKI incidence differed by procedure category, with a higher estimate among embolization/interventional oncology procedures (20 studies) than among neuroendovascular/carotid procedures (18 studies) (8.44% vs. 5.48%; *p* = 0.0171). However, substantial heterogeneity persisted within both subgroups (I^2^ = 84.4% and 90.5%, respectively); therefore, this between-subgroup finding should be regarded as hypothesis-generating rather than evidence of a definitive procedure-specific difference.

When stratified by baseline renal function, the CKD subgroup included only two studies with an aggregate denominator of 287 and demonstrated a pooled CA-AKI incidence of 5.04% (95% CI 0.00–24.65; I^2^ = 95.6%). Studies involving mixed or non-CKD populations (36 studies) demonstrated a pooled incidence of 7.04% (95% CI 5.80–8.38; I^2^ = 89.3%). The between-subgroup difference was not statistically significant (*p* = 0.7921). However, given the very small number of dedicated CKD studies, wide confidence interval, and substantial within-subgroup heterogeneity, this analysis had limited statistical power and should be considered exploratory; the absence of a statistically significant subgroup difference should not be interpreted as evidence that CA-AKI incidence is similar between CKD and non-CKD populations.

Similarly, pooled incidence differed numerically according to study-level diagnostic criteria. Studies using standardised CA-AKI definitions based on KDIGO, AKIN, ICA, or ESUR criteria (14 studies) demonstrated a pooled incidence of 5.67% (95% CI 4.10–7.47), whereas studies using study-specific or CIN-based definitions (24 studies) demonstrated a pooled incidence of 7.76% (95% CI 5.89–9.87). The subgroup difference did not reach statistical significance (*p* = 0.1175). Detailed subgroup findings are presented in Table 3 and Supplementary Figures S1–S3.

**Table 3:** Subgroup analysis of pooled incidence of contrast-associated acute kidney injury following interventional radiology procedures.

Subgroup category	Subgroup	No. of studies	Aggregate denominator (n)^a^	Pooled incidence (%)	95% CI	I^2^ (%)	*P*-value for subgroup difference
Procedure type	Neuroendovascular/carotid procedures	18	13,541	5.48	4.05–7.09	90.5	0.0171
	Embolization/interventional oncology	20	5,602	8.44	6.53–10.56	84.4	
Baseline renal function	CKD populations	2	287	5.04	0.00–24.65	95.6	0.7921
	Non-CKD/mixed renal function populations	36	18,856	7.04	5.80–8.38	89.3	
Definition of CA-AKI	Standardised criteria (KDIGO/AKIN/ICA/ESUR)	14	9,341	5.67	4.10–7.47	85.3	0.1175
	Study-specific or CIN-based definitions	24	9,802	7.76	5.89–9.87	91.1	

Subgroup analyses were conducted using random-effects meta-analysis of proportions. *P*-values represent between-subgroup differences derived from subgroup comparison analyses. Definitions of CA-AKI were classified according to the original study-reported diagnostic criteria.

AKIN, Acute Kidney Injury Network; CA-AKI, contrast-associated acute kidney injury; CI, confidence interval; CIN, contrast-induced nephropathy; CKD, chronic kidney disease; ESUR, European Society of Urogenital Radiology; I^2^, Higgins inconsistency statistic; ICA, International Club of Ascites; KDIGO, Kidney Disease: Improving Global Outcomes.

a Aggregate denominator represents the sum of the study-specific analytic denominators and may comprise patients, procedures, or treatment sessions according to the unit of analysis reported by the original studies; it should not be interpreted as the number of unique patients.

### Risk factors associated with contrast-associated acute kidney injury (CA-AKI)

Reported risk factors for CA-AKI varied across studies but consistently included impaired baseline renal function, diabetes mellitus, hypertension, anaemia or low haemoglobin, hypoalbuminaemia, higher contrast volume, advanced liver dysfunction, ascites, and greater procedural complexity. Several studies also identified inflammatory biomarkers, including neutrophil-to-lymphocyte ratio, systemic inflammatory indices, and high-sensitivity C-reactive protein, as predictors of CA-AKI following neuroendovascular procedures. In hepatocellular carcinoma cohorts undergoing TACE, advanced liver disease severity, repeated TACE exposure, and low serum albumin were among the most consistently reported predictors. Detailed risk factor analyses are summarized in Supplementary Table S4.

### Clinical outcomes associated with contrast-associated acute kidney injury (CA-AKI)

Across the included studies, CA-AKI was associated with a range of adverse clinical outcomes, although the magnitude and statistical significance of these associations varied across studies and outcomes. In neuroendovascular stroke cohorts, CA-AKI was associated in several studies with increased mortality, poorer functional outcomes, and haemorrhagic complications, although findings were not consistent across all studies and outcomes. Similarly, studies involving TACE procedures demonstrated associations between CA-AKI and reduced long-term survival, particularly among patients with advanced hepatocellular carcinoma or severe liver dysfunction. Although most CA-AKI episodes were mild and transient, a minority of patients required renal replacement therapy or developed persistent renal impairment. Detailed clinical outcome findings are presented in Supplementary Table S5.

### Risk of bias assessment

Assessment using the Newcastle–Ottawa Scale demonstrated variation in methodological quality across the included observational studies. Total NOS scores ranged from 3 to 8 out of 9. Fourteen studies were classified as having low risk of bias, 24 as moderate risk of bias, and one as high risk of bias. The most frequent methodological limitations related to retrospective study design, selection based on availability of post-procedural renal measurements, incomplete follow-up or missing outcome data, limited adjustment for potential confounding, and analysis of repeated procedures or treatment sessions. Detailed study-level assessments are presented in Supplementary Table S6.

### Certainty of evidence

Using the GRADE framework, the certainty of evidence for the overall CA-AKI incidence estimate and the procedure-specific incidence estimates was rated as very low. The principal reasons for downgrading were substantial risk-of-bias concerns across the observational evidence base and marked inconsistency, with considerable between-study heterogeneity. Additional concerns related to clinical and methodological indirectness arising from differences in procedural populations, CA-AKI definitions, ascertainment windows, and units of analysis. Detailed GRADE judgments are presented in Supplementary Table S8.

## Discussion

This systematic review and meta-analysis evaluated the incidence, risk factors, and clinical outcomes of CA-AKI following interventional radiology and related non-coronary endovascular procedures. Across 38 studies included in the quantitative synthesis, the pooled incidence of CA-AKI was 6.91%, although substantial heterogeneity was observed across included studies. The incidence of CA-AKI varied considerably according to procedural type, baseline renal function, underlying disease severity, and study-level diagnostic criteria. Importantly, several included studies demonstrated clinically meaningful associations between CA-AKI and adverse outcomes, including increased mortality, poor functional outcomes, prolonged hospitalisation, and persistent renal dysfunction. The unit-of-analysis sensitivity analysis yielded a similar pooled incidence when restricted to patient-level estimates (7.27% versus 6.91% in the primary analysis), indicating numerical stability of the summary estimate after restriction to patient-level data while avoiding interpretation of procedure/session denominators as unique patients.

The exploratory procedure-category subgroup analysis suggested a higher pooled CA-AKI incidence among embolization/interventional oncology procedures than among neuroendovascular/carotid procedures (8.44% vs. 5.48%; *p* = 0.0171). However, this finding should be interpreted as hypothesis-generating rather than as evidence of a definitive procedure-specific effect. Substantial heterogeneity persisted within both subgroups, and the comparison was based on study-level rather than individual-patient characteristics. Differences in underlying populations, including liver disease severity and baseline renal function (5, 9–12, 18, 22, 24, 26, 32–43), as well as variation in procedural characteristics, contrast exposure, CA-AKI definitions, and ascertainment practices across studies (5–43), may have contributed to the observed difference. Consequently, the subgroup result does not establish that procedure type itself is responsible for the difference in CA-AKI incidence.

The substantial heterogeneity observed in the primary analysis persisted within major subgroups and across leave-one-out analyses, indicating that the pooled incidence should not be interpreted as a single expected incidence applicable to all interventional radiology populations. Rather, the overall random-effects estimate provides a descriptive summary across clinically and methodologically diverse studies, and procedure- and population-specific estimates remain important for interpretation. Nevertheless, the leave-one-out analysis demonstrated numerical stability of the summary estimate, with pooled incidences ranging from 6.56% to 7.16% after sequential exclusion of individual studies. Notably, exclusion of Schwarz et al. (15), the largest cohort (*n* = 6,638), yielded a pooled incidence of 6.99%, compared with 6.91% in the primary analysis, indicating that the overall summary estimate was not disproportionately driven by this large study.

The heterogeneity observed across included studies was expected given the substantial variability in procedural indications, baseline patient characteristics, definitions of CA-AKI, and follow-up intervals (5–43). Importantly, heterogeneity likely also reflects evolving conceptual distinctions between CA-AKI and CIN (1–4). Contemporary evidence increasingly suggests that renal dysfunction following contrast exposure may often be multifactorial rather than exclusively attributable to iodinated contrast media itself (1–3). Several included studies involved critically ill patients with stroke, advanced malignancy, sepsis, hypotension, liver failure, or severe systemic inflammation, all of which may independently contribute to acute kidney injury risk (6, 8, 15, 19, 20, 27, 38, 39). Consequently, the broader CA-AKI terminology used throughout this review likely provides a more clinically appropriate framework than traditional CIN terminology alone (1–4).

Baseline renal dysfunction was among the most consistently reported predictors of CA-AKI across included studies (5–43). Multiple studies demonstrated increased risk among patients with reduced estimated glomerular filtration rate (eGFR), CKD, or elevated baseline serum creatinine (7, 8, 19–21, 24, 33, 38, 43). However, subgroup analysis according to baseline CKD status did not demonstrate a statistically significant difference in pooled incidence. This finding should be interpreted with substantial caution because only two studies specifically enrolled dedicated CKD cohorts (29, 42), comprising an aggregate denominator of 287, resulting in limited statistical power, a very wide confidence interval around the pooled CKD estimate, and substantial within-subgroup heterogeneity (I^2^ = 95.6%). Accordingly, this subgroup analysis should be considered exploratory, and the absence of statistical significance should not be interpreted as evidence of comparable CA-AKI incidence between CKD and non-CKD populations. Moreover, several studies excluded patients with severe renal impairment, potentially underestimating the true incidence of CA-AKI in high-risk populations (11, 12, 18, 24, 32, 33). Advanced liver dysfunction also emerged as an important contributor to CA-AKI risk in interventional oncology cohorts (5, 11, 18, 22, 32–37, 41). Several TACE studies identified hypoalbuminaemia, ascites, higher Child–Pugh class, elevated bilirubin levels, and advanced tumor burden as predictors of postprocedural AKI (5, 11, 18, 22, 32–37, 41). These findings likely reflect the complex hemodynamic and inflammatory disturbances associated with advanced cirrhosis and hepatocellular carcinoma, including impaired renal perfusion, systemic vasodilation, portal hypertension, and increased susceptibility to hepatorenal physiology (5, 11, 18, 22, 32–37, 41). Studies using ICA cirrhosis-specific AKI definitions additionally highlighted the importance of applying liver disease-specific renal criteria when evaluating renal injury in cirrhotic populations (11, 35).

Several neuroendovascular studies identified inflammatory and vascular risk markers associated with CA-AKI development (6–8, 13–17, 19–21, 23, 25, 27–31, 38, 39). Elevated neutrophil-to-lymphocyte ratio, systemic inflammatory response indices, high-sensitivity C-reactive protein, diabetes mellitus, severe stroke burden, and unsuccessful reperfusion were repeatedly associated with higher AKI risk (6, 19, 21, 28). These findings support the concept that systemic inflammation, ischemia-reperfusion injury, endothelial dysfunction, and hemodynamic instability may substantially contribute to renal injury following neurovascular interventions (6–8, 13–17, 19–21, 23, 25, 27–31, 38, 39). Similarly, studies involving acute ischemic stroke populations frequently demonstrated worse neurological and functional outcomes among patients who developed CA-AKI (6, 19, 27, 39).

Importantly, although many CA-AKI episodes were mild and transient, several studies demonstrated clinically meaningful long-term consequences (5–43). CA-AKI was associated with increased short-term and long-term mortality across multiple cohorts (6, 8, 11, 15, 25–27, 32, 34, 35, 38, 41). In stroke populations, CA-AKI was associated with worse modified Rankin Scale outcomes, increased haemorrhagic transformation, and higher 3-month mortality following endovascular thrombectomy (6, 19, 27). In hepatocellular carcinoma cohorts, AKI following TACE was associated with reduced long-term survival and persistent renal dysfunction (6, 8, 11, 15, 25–27, 32, 34, 35, 38, 41). These findings emphasize that CA-AKI should not be considered a purely transient biochemical abnormality, particularly among medically complex patients undergoing repeated endovascular procedures (6, 8, 11, 15, 25–27, 32, 34, 35, 38, 41).

Mitaka et al. additionally demonstrated significant postoperative reductions in serum creatinine and transient increases in eGFR following neuroendovascular procedures despite evidence of subclinical renal injury identified using urine neutrophil gelatinase-associated lipocalin (uNGAL) (23). The authors proposed that perioperative haemodilution, transcapillary leakage, and postoperative physiologic changes may partially mask early renal dysfunction when relying solely on serum creatinine-based definitions (23). Similarly, Prasad et al. observed paradoxical reductions in serum creatinine following high-dose neuroendovascular contrast exposure, which were attributed to aggressive hydration and perioperative hemodynamic changes (17). These findings collectively highlight the limitations of serum creatinine as the sole biomarker of renal injury in interventional settings and suggest that conventional creatinine-based criteria may underestimate the true incidence of contrast-associated renal injury following neuroendovascular procedures (17). Despite increased PC-AKI incidence among patients with severe baseline renal impairment, Myung et al. reported no fatal renal adverse outcomes and emphasized that aggressive neurovascular management should not be delayed when clinically indicated (20).

The findings of this review have several important clinical implications (5–43). First, patients with advanced liver disease, baseline renal dysfunction, diabetes mellitus, anaemia, hypoalbuminaemia, or high inflammatory burden may benefit from enhanced renal-protective strategies before and after interventional radiology procedures (5, 11, 18, 22, 32–37, 41). Second, procedural planning should minimise unnecessary contrast exposure whenever clinically feasible. However, the available evidence did not establish a reliable contrast-volume threshold, and associations with repeated procedures should be interpreted cautiously because of potential confounding by disease severity and treatment complexity (5, 11, 18, 22, 32–37, 41). Third, the substantial heterogeneity in CA-AKI definitions identified across included studies underscores the need for greater standardization of renal outcome reporting in future interventional radiology research (1–4). Broader adoption of contemporary KDIGO-based definitions may improve comparability across future studies (1–4).

This review has several limitations. First, substantial clinical and methodological heterogeneity was present across studies, including differences in patient populations, procedural types, CA-AKI definitions, ascertainment windows, contrast protocols, and follow-up. Periprocedural hydration and hemodynamic factors were also inconsistently reported and could not be systematically accounted for. Accordingly, the pooled incidence should be interpreted as a descriptive summary rather than a universally applicable estimate. The primary analysis additionally combined patient-level and procedure/session-level estimates, which represent different units of analysis and may include repeated observations within patients; however, restriction to patient-level estimates yielded a similar pooled incidence. Potential cohort overlap among several reports could not be completely excluded, although sensitivity analysis suggested that this did not materially affect the overall estimate.

Second, eligibility assessment of mixed procedural cohorts was not based on a prespecified quantitative threshold and therefore required reviewer judgement. All included studies were observational, and a substantial proportion had moderate or high risk of bias, particularly because of selection, incomplete outcome ascertainment, and limited adjustment for confounding. CA-AKI definitions and assessment windows also varied considerably, limiting direct comparability across studies. Some neurovascular cohorts included both intravenous contrast from preprocedural CTA/CTP and intra-arterial contrast during the intervention; consequently, the relative contribution of each route to CA-AKI could not be determined.

Third, several studies excluded patients with severe renal dysfunction or dialysis dependence, and the CKD subgroup included only two dedicated studies with a small aggregate denominator, wide confidence interval, and substantial heterogeneity. Its findings should therefore be considered exploratory. The restriction to English-language publications may have introduced language bias, while the predominance of studies from East Asia may limit geographic generalisability. Although pediatric populations were eligible, no pediatric studies met the inclusion criteria, and the findings should therefore not be extrapolated to children. Finally, funnel-plot asymmetry and Egger's test indicated small-study effects; however, given the substantial between-study heterogeneity, these findings cannot be attributed specifically to publication bias.

## Conclusion

Contrast-associated acute kidney injury (CA-AKI) represents a clinically important complication following interventional radiology and related non-coronary endovascular procedures, with a pooled incidence of approximately 7% across the study-reported units of analysis; a patient-level sensitivity analysis produced a similar estimate of 7.27%. Exploratory subgroup analysis suggested a higher pooled incidence among embolization/interventional oncology procedures than among neuroendovascular/carotid procedures; however, given the substantial residual heterogeneity and observational nature of the evidence, this finding should be considered hypothesis-generating and requires confirmation in prospective studies. Several studies reported associations between CA-AKI and adverse clinical outcomes, including increased mortality, poorer functional outcomes, prolonged hospitalisation, and persistent renal dysfunction.

Across heterogeneous observational studies, baseline renal impairment, advanced liver disease, diabetes mellitus, anaemia, hypoalbuminaemia, inflammatory burden, and greater contrast exposure were frequently associated with CA-AKI. However, the available evidence did not establish a clinically reliable contrast-volume threshold, and associations with repeated procedures may partly reflect underlying disease severity, cumulative illness burden, and treatment complexity. Although many CA-AKI episodes were mild and reversible, severe renal injury requiring renal replacement therapy occurred in a subset of patients, particularly among medically complex populations.

These findings provide a quantitative benchmark for CA-AKI incidence across non-coronary interventional procedures and highlight potential variation across procedural categories. Greater standardisation of CA-AKI definitions and reporting frameworks is needed to improve comparability across studies. Further prospective multicentre research is warranted to confirm procedure-specific patterns, define clinically relevant risk profiles, evaluate preventive strategies, and clarify the long-term renal and clinical consequences of CA-AKI in interventional radiology populations.

## Data Availability

The original contributions presented in the study are included in the article/Supplementary Material, further inquiries can be directed to the corresponding author.

## Glossary

ACE: angiotensin-converting enzyme
AFP: alpha-fetoprotein
AIS: acute ischemic stroke
AJCC: American Joint Committee on Cancer
AKI: acute kidney injury
AKIN: Acute Kidney Injury Network
ALBI: albumin–bilirubin
ARB: angiotensin II receptor blocker
ARF: acute renal failure
ASPECTS: Alberta Stroke Program Early CT Score
AST: aspartate aminotransferase
aSAH: aneurysmal subarachnoid haemorrhage
AUC: area under the curve
AVM: arteriovenous malformation
BAE: bronchial artery embolization
BCLC: Barcelona Clinic Liver Cancer staging system
CA-AKI: contrast-associated acute kidney injury
CAN-REST: Contrast-Associated Nephropathy Risk Evaluation in Acute Ischemic Stroke After Endovascular Thrombectomy
CAS: carotid artery stenosis
CG-GFR: Cockcroft–Gault glomerular filtration rate
CI: confidence interval
CI-AKI: contrast-induced acute kidney injury
CIN: contrast-induced nephropathy
CKD: chronic kidney disease
CRRT: continuous renal replacement therapy
CT: computed tomography
CTA: computed tomography angiography
cTACE: conventional transarterial chemoembolization
CTP: computed tomography perfusion
CVVH: continuous veno-venous hemofiltration
DAVF: dural arteriovenous fistula
DEMs: drug-eluting microspheres
DSA: digital subtraction angiography
eGFR: estimated glomerular filtration rate
ESUR: European Society of Urogenital Radiology
EVT: endovascular thrombectomy
GCS: Glasgow Coma Scale
GEE: generalized estimating equation
GFR: glomerular filtration rate
h: hour
HbA1c: glycated haemoglobin
HAE: hepatic artery embolization
HBsAg: hepatitis B surface antigen
HCC: hepatocellular carcinoma
HCV-Ab: hepatitis C virus antibody
HR: hazard ratio
hs-CRP: high-sensitivity C-reactive protein
IA: intra-arterial
ICA: International Club of Ascites
ICD-9-CM: International Classification of Diseases, Ninth Revision, Clinical Modification
ICU: intensive care unit
IgA: immunoglobulin A
IQR: interquartile range
IV: intravenous
KDIGO: Kidney Disease: Improving Global Outcomes
MACD: maximum allowable contrast dose
MCD: maximum contrast dose
MDRD: Modification of Diet in Renal Disease
MELD: Model for End-Stage Liver Disease
mRS: modified Rankin Scale
MT: mechanical thrombectomy
mTICI: modified thrombolysis in cerebral infarction
NGAL: neutrophil gelatinase-associated lipocalin
NIHSS: National Institutes of Health Stroke Scale
NR: not reported
NSAID: nonsteroidal anti-inflammatory drug
OR: odds ratio
PC-AKI: post-contrast acute kidney injury
POD: postoperative day
PRISMA: Preferred Reporting Items for Systematic Reviews and Meta-Analyses
PSM: propensity score matching
PT-INR: prothrombin time–international normalized ratio
RCIN: radiocontrast-induced nephropathy
ROC: receiver operating characteristic
RR: relative risk
RRT: renal replacement therapy
SAH: subarachnoid haemorrhage
TACE: transcatheter arterial chemoembolization
TAE: transcatheter arterial embolization
TAI: transarterial infusion chemotherapy
TICI: thrombolysis in cerebral infarction
tPA: tissue plasminogen activator
uNGAL: urine neutrophil gelatinase-associated lipocalin.
