# Supplementary material for: Incidence, risk factors, and outcomes of contrast-associated acute kidney injury following interventional radiology procedures: a systematic review and meta-analysis
Source: Front Radiol. 2026 Sep 23;6:1947256. doi: 10.3389/fradi.2026.1947256 (PMC13642721; doi:10.3389/fradi.2026.1947256)
Supplement: Supplementary file 1 [file Supplementaryfile1.docx]

Supplementary Figure S1. Forest plot of subgroup analysis of pooled incidence of contrast-associated acute kidney injury according to procedure category. Study denominators represent patients, procedures, or treatment sessions according to the unit of analysis reported in the original study.

Supplementary Figure S2. Forest plot of subgroup analysis of pooled incidence of contrast-associated acute kidney injury according to baseline chronic kidney disease status. Study denominators represent patients, procedures, or treatment sessions according to the unit of analysis reported in the original study.

Supplementary Figure S3. Forest plot of subgroup analysis of pooled incidence of contrast-associated acute kidney injury according to study-level diagnostic definitions. Study denominators represent patients, procedures, or treatment sessions according to the unit of analysis reported in the original study.

Supplementary Figure S4. Sensitivity analysis of pooled contrast-associated acute kidney injury incidence restricted to patient-level estimates. The analysis included 30 studies representing 16,825 unique patients. Studies reporting incidence only per procedure or treatment session were excluded; where an extractable patient-level estimate was available from the same study, the patient-level estimate was used. The pooled random-effects incidence was 7.27% (95% CI 5.77–8.92; I² = 91.5%), compared with 6.91% (95% CI 5.69–8.24; I² = 89.5%) in the primary mixed-unit analysis.

Supplementary Figure S5. Sensitivity analysis of pooled contrast-associated acute kidney injury incidence addressing potential cohort overlap. Huo et al. [32] was retained, while Huo et al. [33] and Hsu et al. [34] were excluded because potential participant overlap across these reports could not be excluded. The pooled random-effects incidence across the remaining 36 studies was 6.77% (95% CI 5.53–8.12; I² = 89.9%), compared with 6.91% (95% CI 5.69–8.24; I² = 89.5%) in the primary analysis.

Supplementary Figure S6. Sensitivity analysis of pooled contrast-associated acute kidney injury incidence excluding Cohen et al. [40], which reported acute kidney injury as a discrete post-TACE adverse event without specifying a formal creatinine-based diagnostic threshold. The pooled random-effects incidence across the remaining 37 studies was 6.73% (95% CI 5.53–8.03; I² = 89.0%), compared with 6.91% (95% CI 5.69–8.24; I² = 89.5%) in the primary analysis.

Supplementary Figure S7. Leave-one-out sensitivity analysis of pooled contrast-associated acute kidney injury incidence. Each study was sequentially excluded and the random-effects pooled incidence recalculated using the same analytical approach as the primary meta-analysis. Pooled incidence estimates ranged from 6.56% to 7.16%, while heterogeneity remained substantial across all iterations (I² range, 87.4–89.8%). Exclusion of Schwarz et al. [15], the largest included cohort (n = 6,638), yielded a pooled incidence of 6.99% (95% CI 5.60–8.52; I² = 89.3%), compared with 6.91% (95% CI 5.69–8.24; I² = 89.5%) in the primary analysis.

Supplementary Figure S8. Funnel plot for assessment of small-study effects in the primary meta-analysis of contrast-associated acute kidney injury incidence. The funnel plot includes all 38 studies contributing to the primary random-effects meta-analysis. Egger’s linear regression test identified statistically significant funnel-plot asymmetry (t = 2.35, df = 36, p = 0.0246). Given the substantial between-study heterogeneity (I² = 89.5%), the observed asymmetry should be interpreted as evidence of small-study effects rather than definitive evidence of publication bias.
