# Supplementary material for: Incidence, risk factors, and outcomes of contrast-associated acute kidney injury following interventional radiology procedures: a systematic review and meta-analysis
Source: Front Radiol. 2026 Sep 23;6:1947256. doi: 10.3389/fradi.2026.1947256 (PMC13642721; doi:10.3389/fradi.2026.1947256)
Supplement: Supplementary file 3 [file Table1.docx]

Supplementary Table S1. Database-specific search syntax.

**Scopus**

S1 TITLE-ABS-KEY ( ( "contrast-associated acute kidney injury" OR "contrast-induced nephropathy" OR "CA-AKI" OR "CIN" ) AND ( "interventional radiology" OR "image guided intervention*" OR "endovascular procedure*" OR embolization OR angiography ) ) (2,732)

**Embase <1974 to 2026 April 30>**

1	('acute kidney injury' or 'contrast induced nephropathy' or 'contrast associated acute kidney injury' or CA-AKI or CIN).mp. [mp=title, abstract, heading word, drug trade name, original title, device manufacturer, drug manufacturer, device trade name, keyword heading word, floating subheading word, candidate term word]	117128

2	('interventional radiology' or 'image guided intervention*' or 'endovascular procedure*' or embolization or angiography).mp. [mp=title, abstract, heading word, drug trade name, original title, device manufacturer, drug manufacturer, device trade name, keyword heading word, floating subheading word, candidate term word]	651576

3	(('acute kidney injury' or 'contrast induced nephropathy' or 'contrast associated acute kidney injury' or CA-AKI or CIN) and ('interventional radiology' or 'image guided intervention*' or 'endovascular procedure*' or embolization or angiography)).mp. [mp=title, abstract, heading word, drug trade name, original title, device manufacturer, drug manufacturer, device trade name, keyword heading word, floating subheading word, candidate term word] 5595

**CINAHL**

S3 ("contrast-associated acute kidney injury" OR "contrast-induced nephropathy" OR "CA-AKI" OR "CIN") AND ("interventional radiology" OR "image guided intervention*" OR "image-guided intervention*" OR "endovascular procedure*" OR embolization OR angiography) (352)

S2 ("interventional radiology" OR "image guided intervention*" OR "image-guided intervention*" OR "endovascular procedure*" OR embolization OR angiography) (81,462)

S1 ("contrast-associated acute kidney injury" OR "contrast-induced nephropathy" OR "CA-AKI" OR "CIN") (2,844)

**PubMed**

**3 Search:** ("Acute Kidney Injury"[MeSH Terms] OR "contrast-induced nephropathy"[Title/Abstract] OR "contrast-associated acute kidney injury"[Title/Abstract] OR "CA-AKI"[Title/Abstract] OR "CIN"[Title/Abstract]) AND ("radiology, interventional"[MeSH Terms] OR "interventional radiology"[Title/Abstract] OR ("angiography"[Title/Abstract] AND "contrast"[Title/Abstract]) OR ("embolization"[Title/Abstract] AND "contrast"[Title/Abstract]) OR "endovascular procedure*"[Title/Abstract] OR "image guided intervention*"[Title/Abstract]) (1,423)

**2 Search:** "radiology, interventional"[MeSH Terms] OR "interventional radiology"[Title/Abstract] OR ("angiography"[Title/Abstract] AND "contrast"[Title/Abstract]) OR ("embolization"[Title/Abstract] AND "contrast"[Title/Abstract]) OR "endovascular procedure*"[Title/Abstract] OR "image guided intervention*"[Title/Abstract] (51,442)

**1 Search:** "Acute Kidney Injury"[MeSH Terms] OR "contrast-induced nephropathy"[Title/Abstract] OR "contrast-associated acute kidney injury"[Title/Abstract] OR "CA-AKI"[Title/Abstract] OR "CIN"[Title/Abstract] (77,991)

**Web of Science Core Collection**

S3 ("contrast-associated acute kidney injury" OR "contrast-induced nephropathy" OR "CA-AKI" OR "CIN") (Topic) and ("interventional radiology" OR "image guided intervention*" OR "image-guided intervention*" OR "endovascular procedure*" OR embolization OR angiography) (All Fields) (1,941)

S2 ("interventional radiology" OR "image guided intervention*" OR "image-guided intervention*" OR "endovascular procedure*" OR embolization OR angiography) (Topic) (305,509)

S1 ("contrast-associated acute kidney injury" OR "contrast-induced nephropathy" OR "CA-AKI" OR "CIN") (Topic) (18,640)

Supplementary Table S2. Studies excluded at full-text stage.

| Article excluded | Reason for exclusion |
| --- | --- |
| Abdel-Hady Y, Taha M, El Barbary A, et al. Trimetazidine effect on kidney function in patients undergoing coronary procedures. BMC Pharmacol Toxicol. 2025;26:85. https://doi.org/10.1186/s40360-025-00913-3 | Wrong procedure specialty/population. This study evaluated contrast-induced nephropathy following coronary angiography and percutaneous coronary intervention in patients with chronic coronary syndrome. The procedures were performed in an interventional cardiology setting rather than interventional radiology and therefore did not meet the predefined eligibility criteria for this review. |
| Abe M, Kimura T, Morimoto T, et al. Incidence of and risk factors for contrast-induced nephropathy after cardiac catheterization in Japanese patients. Circ J. 2009;73:1518–1522. https://doi.org/10.1253/circj.CJ-08-0784 | Wrong procedure specialty/population. This study evaluated contrast-induced nephropathy following cardiac catheterization and percutaneous coronary intervention in a cardiology population. The procedures were performed in an interventional cardiology setting rather than interventional radiology and therefore did not meet the predefined eligibility criteria for this review. |
| Abe M, Morimoto T, Akao M, et al. Relation of contrast-induced nephropathy to long-term mortality after percutaneous coronary intervention. Am J Cardiol. 2014;114:362–368. https://doi.org/10.1016/j.amjcard.2014.05.009 | Wrong procedure specialty/population. This study evaluated contrast-induced nephropathy and long-term mortality following percutaneous coronary intervention in a cardiology population. The procedures were performed in an interventional cardiology setting rather than interventional radiology and therefore did not meet the predefined eligibility criteria for this review. |
| Abe M, Morimoto T, Nakagawa Y, et al. Impact of transient or persistent contrast-induced nephropathy on long-term mortality after elective percutaneous coronary intervention. Am J Cardiol. 2017;120:2146–2153. https://doi.org/10.1016/j.amjcard.2017.08.036 | Wrong procedure specialty/population. This study evaluated contrast-induced nephropathy and long-term mortality following elective percutaneous coronary intervention in a cardiology population. The procedures were performed in an interventional cardiology setting rather than interventional radiology and therefore did not meet the predefined eligibility criteria for this review. |
| Abood ZM, Rasheed MK, Omran HH. Significance of cystatin C for early diagnosis of contrast-induced nephropathy in Iraqi patients undergoing coronary angiography. Med J Babylon. 2021;18:191–194. https://doi.org/10.4103/MJBL.MJBL_88_20 | Wrong procedure specialty/population. This study evaluated contrast-induced nephropathy biomarkers in patients undergoing coronary angiography and percutaneous coronary intervention in a cardiology population. The procedures were performed in an interventional cardiology setting rather than interventional radiology and therefore did not meet the predefined eligibility criteria for this review. |
| ACT Trial Investigators. Rationale, design, and baseline characteristics of the Acetylcystein for Contrast-Induced nephropaThy (ACT) Trial: a pragmatic randomized controlled trial to evaluate the efficacy of acetylcysteine for the prevention of contrast-induced nephropathy. Trials. 2009;10:38. https://doi.org/10.1186/1745-6215-10-38 | Wrong procedure specialty/population and study type. This article describes the rationale, design, and baseline characteristics of the ACT Trial evaluating acetylcysteine for prevention of contrast-induced nephropathy in patients undergoing coronary or peripheral angiographic procedures. It is a study protocol and baseline report rather than a completed clinical study with extractable outcome data, and the included procedures were predominantly performed in cardiology settings rather than interventional radiology. |
| ACT Investigators. Acetylcysteine for prevention of renal outcomes in patients undergoing coronary and peripheral vascular angiography: main results from the randomized Acetylcysteine for Contrast-Induced Nephropathy Trial (ACT). Circulation. 2011;124:1250–1259. https://doi.org/10.1161/CIRCULATIONAHA.111.038943 | Wrong procedure specialty/population. This randomized controlled trial evaluated acetylcysteine for prevention of contrast-induced acute kidney injury in patients undergoing coronary and peripheral vascular angiography, including percutaneous coronary intervention procedures. The included procedures were predominantly performed in interventional cardiology settings rather than interventional radiology and therefore did not meet the predefined eligibility criteria for this review. |
| Adıyaman M, Bora R, Toprak B, et al. Renal safety of SGLT-2 inhibitors in patients undergoing coronary angiography: a focus on glycemic status and contrast-induced nephropathy. Heart Surg Forum. 2026;29(3):47635. https://doi.org/10.31083/HSF47635 | Wrong procedure specialty/population. This study evaluated contrast-induced nephropathy and renal outcomes in patients with type 2 diabetes undergoing coronary angiography and related cardiac procedures. The procedures were performed in an interventional cardiology setting rather than interventional radiology and therefore did not meet the predefined eligibility criteria for this review. |
| Agrawal M, Stouffer GA. Contrast Induced Nephropathy after Angiography. Am J Med Sci. 2002;323:252–258. http://doi.org/10.1097/00000441-200205000-00004 | Ineligible publication type and wrong procedure specialty. This article was a narrative review discussing contrast-induced nephropathy after coronary angiography and interventional cardiology procedures. The publication did not report original patient-level data from interventional radiology procedures and therefore did not meet the predefined eligibility criteria for inclusion in this review. |
| Agrawal V, Swami A, Kosuri R, et al. Contrast-induced acute kidney injury in renal transplant recipients after cardiac catheterization. Clin Nephrol. 2009;71(6):687–696. https://doi.org/10.5414/CNP71687 | Wrong procedure specialty/population. This study evaluated contrast-induced acute kidney injury in renal transplant recipients undergoing cardiac catheterization and coronary angiography procedures. The procedures were performed in an interventional cardiology setting rather than interventional radiology and therefore did not meet the predefined eligibility criteria for this review. |
| Agrawal M, Wodlinger AM, Huggins CE, et al. Effect of N-acetylcysteine on serum creatinine concentration in patients with chronic renal insufficiency who are undergoing coronary angiography. Heart Drug. 2004;4:87–91. https://doi.org/10.1159/000077294 | Wrong procedure specialty/population. This randomized study evaluated N-acetylcysteine for prevention of contrast-induced nephropathy in patients with chronic renal insufficiency undergoing coronary angiography and percutaneous coronary intervention. The procedures were performed in an interventional cardiology setting rather than interventional radiology and therefore did not meet the predefined eligibility criteria for this review. |
| Aguiar-Souto P, Ferrante G, Del Furia F, et al. Frequency and predictors of contrast-induced nephropathy after angioplasty for chronic total occlusions. Int J Cardiol. 2010;139:68–74. https://doi.org/10.1016/j.ijcard.2008.10.006 | Wrong procedure specialty/population. This study evaluated contrast-induced nephropathy following percutaneous coronary intervention for chronic total coronary occlusions. The procedures were performed in an interventional cardiology setting rather than interventional radiology and therefore did not meet the predefined eligibility criteria for this review. |
| Ajami G, Derakhshan A, Amoozgar H, et al. Risk of nephropathy after consumption of nonionic contrast media by children undergoing cardiac angiography: a prospective study. Pediatr Cardiol. 2010;31:668–673. https://doi.org/10.1007/s00246-010-9680-2 | Wrong procedure specialty/population. This prospective study evaluated contrast-induced nephropathy in pediatric patients undergoing cardiac angiography for congenital heart disease assessment and intervention. The procedures were performed in a pediatric cardiology/cardiac catheterization setting rather than interventional radiology and therefore did not meet the predefined eligibility criteria for this review. |
| Akgül A, Tunca Ç, Taş A, et al. Association of serum uric acid and CRP/albumin ratio with contrast-induced nephropathy in acute myocardial infarction patients. Angiology. 2025. https://doi.org/10.1177/00033197251376082 | Wrong procedure specialty/population. This study evaluated predictors of contrast-induced nephropathy in patients with acute myocardial infarction undergoing coronary angiography and percutaneous coronary intervention. The procedures were performed in an interventional cardiology setting rather than interventional radiology and therefore did not meet the predefined eligibility criteria for this review. |
| Akkaya S, Cakmak U. Association between pan-immune-inflammation value and contrast-induced nephropathy with coronary angiography. Medicina (Kaunas). 2024;60:1012. https://doi.org/10.3390/medicina60061012 | Wrong procedure specialty/population. This study evaluated the association between pan-immune-inflammation value and contrast-induced nephropathy in patients undergoing coronary angiography for stable ischemic heart disease. The procedures were performed in an interventional cardiology setting rather than interventional radiology and therefore did not meet the predefined eligibility criteria for this review. |
| Aktas H, Inci S, Gul M, et al. Increased triglyceride-glucose index predicts contrast-induced nephropathy in non-diabetic NSTEMI patients: a prospective study. J Investig Med. 2023;71(8):838–844. https://doi.org/10.1177/10815589231182317 | Wrong procedure specialty/population. This prospective study evaluated the relationship between triglyceride-glucose index and contrast-induced nephropathy in non-diabetic patients with non-ST elevation myocardial infarction undergoing coronary angiography and percutaneous coronary intervention. The procedures were performed in an interventional cardiology setting rather than interventional radiology and therefore did not meet the predefined eligibility criteria for this review. |
| Al Adas Z, Lodewyk K, Robinson D, et al. Contrast-induced nephropathy after peripheral vascular intervention: long-term renal outcome and risk factors for progressive renal dysfunction. J Vasc Surg. 2019;69:913–920. https://doi.org/10.1016/j.jvs.2018.06.196 | Wrong procedure specialty/population. This study evaluated contrast-induced nephropathy and long-term renal outcomes following peripheral vascular interventions performed within a vascular surgery/endovascular intervention setting. Although the procedures involved peripheral vascular interventions, they were not conducted within an interventional radiology framework and therefore did not meet the predefined eligibility criteria for this review. |
| Al Adas Z, Shepard AD, Nypaver TJ, et al. Long-term decline in renal function is more significant after endovascular repair of infrarenal abdominal aortic aneurysms. J Vasc Surg. 2018;68:739–748. https://doi.org/10.1016/j.jvs.2017.12.051 | Wrong procedure specialty/population. This study evaluated acute kidney injury and long-term renal outcomes following endovascular aneurysm repair and open abdominal aortic aneurysm repair performed within a vascular surgery/endovascular surgery setting. The procedures were not conducted within an interventional radiology framework and therefore did not meet the predefined eligibility criteria for this review. |
| Al Awadhi A, Aljabbari SMA, Anwar S, et al. Incidence of contrast-induced nephropathy after coronary procedures in the United Arab Emirates: a single-center study. Saudi J Kidney Dis Transpl. 2020;31(5):1034–1041. http://doi.org/10.4103/1319-2442.301168 | Wrong procedure specialty/population. This study evaluated contrast-induced nephropathy following coronary angiography and coronary interventions in a cardiology population. The procedures were performed in an interventional cardiology setting rather than interventional radiology and therefore did not meet the predefined eligibility criteria for this review. |
| Alakbarzade V, Pereira AC. Cerebral catheter angiography and its complications. Pract Neurol. 2018;18:393–398. https://doi.org/10.1136/practneurol-2018-001986 | Ineligible study design. This article is a narrative review discussing cerebral catheter angiography and its complications, including contrast-induced nephropathy, rather than an original clinical study with extractable patient-level outcome data. Therefore, it did not meet the predefined eligibility criteria for inclusion in this review. |
| Albrecht MH, Vogl TJ, Wichmann JL, et al. Dynamic 4D-CT angiography for guiding transarterial chemoembolization: impact on the reduction of contrast material, operator radiation exposure, catheter consumption, and diagnostic confidence. Fortschr Röntgenstr. 2018;190:513–520. https://doi.org/10.1055/a-0595-7964 | Ineligible outcomes. This study evaluated the use of dynamic 4D-CT angiography for planning transarterial chemoembolization and focused on reduction of contrast material utilization, radiation exposure, catheter consumption, and diagnostic confidence. It did not report extractable incidence, risk factor, or clinical outcome data for contrast-associated acute kidney injury/contrast-induced nephropathy and therefore did not meet the predefined eligibility criteria for this review. |
| Al-Harazi SM, Azzani M, Shirin L, et al. Neutrophil gelatinase-associated lipocalin in contrast-induced acute kidney injury post cardiac procedure: a systematic review. Clin Chim Acta. 2026;588:120977. https://doi.org/10.1016/j.cca.2026.120977 | Ineligible study design and wrong procedure specialty/population. This article is a systematic review evaluating neutrophil gelatinase-associated lipocalin as a biomarker for contrast-induced acute kidney injury following coronary angiography and percutaneous coronary intervention in cardiology populations. It was not an original clinical study and did not involve interventional radiology procedures, and therefore did not meet the predefined eligibility criteria for this review. |
| Alharazy SM, Kong N, Saidin R, et al. Neutrophil gelatinase-associated lipocalin as an early marker of contrast-induced nephropathy after coronary angiography. Angiology. 2014;65(3):216–223. https://doi.org/10.1177/0003319712474947 | Wrong procedure specialty/population. This prospective study evaluated neutrophil gelatinase-associated lipocalin as an early biomarker of contrast-induced nephropathy in patients with chronic kidney disease undergoing coronary angiography and cardiac catheterization procedures. The procedures were performed in an interventional cardiology setting rather than interventional radiology and therefore did not meet the predefined eligibility criteria for this review. |
| Alhudairy M, Abdelazeem M, Aoun J. New perspectives on contrast-induced nephropathy during coronary interventions. Future Cardiol. 2022;18(7):519–522. https://doi.org/10.2217/fca-2021-0080 | Ineligible study design and wrong procedure specialty/population. This article is an editorial/review discussing contrast-induced nephropathy prevention strategies during coronary interventions and percutaneous coronary intervention procedures in cardiology populations. It was not an original clinical study and did not involve interventional radiology procedures, and therefore did not meet the predefined eligibility criteria for this review. |
| Ali M, Noureldin M, Elokda A, et al. Comparative study between mechanical rotational atherectomy combined with drug-coated balloon versus drug-coated balloon alone for treatment of in-stent restenosis during peripheral endovascular interventions: a multicentric trial. J Vasc Dis. 2024;3:290–305. https://doi.org/10.3390/jvd3030023 | Ineligible outcomes. This multicentric study evaluated endovascular treatment strategies for peripheral in-stent restenosis during peripheral endovascular interventions. Although contrast-induced nephropathy was reported as a procedural complication in a small number of patients, the study did not primarily investigate the incidence, risk factors, or clinical outcomes of contrast-associated acute kidney injury/contrast-induced nephropathy and therefore did not meet the predefined eligibility criteria for this review. |
| Ali A, Bhan C, Malik MB, et al. The prevention and management of contrast-induced acute kidney injury: a mini-review of the literature. Cureus. 2018;10(9):e3284. https://doi.org/10.7759/cureus.3284 | Ineligible study design and wrong procedure specialty/population. This article is a narrative mini-review discussing prevention and management strategies for contrast-induced acute kidney injury following radiologic and coronary interventional procedures. It was not an original clinical study and did not specifically evaluate interventional radiology procedures, and therefore did not meet the predefined eligibility criteria for this review. |
| Allen DW, Ma B, Leung KC, et al. Risk prediction models for contrast-induced acute kidney injury accompanying cardiac catheterization: systematic review and meta-analysis. Can J Cardiol. 2017. https://doi.org/10.1016/j.cjca.2017.01.018 | Ineligible study design and wrong procedure specialty/population. This article is a systematic review and meta-analysis evaluating risk prediction models for contrast-induced acute kidney injury following cardiac catheterization and percutaneous coronary intervention procedures in cardiology populations. It was not an original clinical study and did not involve interventional radiology procedures, and therefore did not meet the predefined eligibility criteria for this review. |
| Almalki WH, Abdalla AN, Elkeraie AF, et al. Effect of fluorescein angiography on renal functions in type 2 diabetes patients: a pilot study. Saudi J Kidney Dis Transpl. 2017;28(3):491–498. http://doi.org/10.4103/1319-2442.206444 | Wrong procedure specialty/population. This prospective observational study evaluated renal biomarker changes following fluorescein angiography in patients with diabetic retinopathy. The procedure was performed in an ophthalmologic diagnostic setting rather than an interventional radiology framework and therefore did not meet the predefined eligibility criteria for this review. |
| Althoff CE, Günther RW, Hamm B, et al. Intra-arterial ultra low iodine CT angiography of renal transplant arteries. Cardiovasc Intervent Radiol. 2014;37:1062–1067. https://doi.org/10.1007/s00270-014-0838-9 | Small feasibility/imaging study. This technical feasibility study evaluated ultra-low-dose intra-arterial CT angiography for the diagnosis of transplant renal artery stenosis in 10 patients with impaired renal transplant function. The primary focus was diagnostic image quality and feasibility rather than systematic evaluation of contrast-associated acute kidney injury outcomes according to the predefined eligibility criteria for this review. |
| Amiri A, Ghanavati R, Riahi Beni H, et al. Metabolic syndrome and the iodine-dose/creatinine clearance ratio as determinants of contrast-induced acute kidney injury. Cardiorenal Med. 2018;8:217–227. https://doi.org/10.1159/000488374 | Wrong procedure specialty/population. This prospective cohort study evaluated risk factors for contrast-induced acute kidney injury in patients undergoing elective coronary angiography and percutaneous coronary intervention procedures. The procedures were performed in an interventional cardiology setting rather than interventional radiology and therefore did not meet the predefined eligibility criteria for this review. |
| Annoni AD, Andreini D, Pontone G, et al. CT angiography prior to TAVI procedure using third-generation scanner with wide volume coverage: feasibility, renal safety and diagnostic accuracy for coronary tree. Br J Radiol. 2018;91:20180196. https://doi.org/10.1259/bjr.20180196 | Wrong procedure specialty/population. This study evaluated renal safety and diagnostic performance of low-contrast CT angiography performed for transcatheter aortic valve implantation planning in patients with severe aortic stenosis. The procedures were conducted within a cardiovascular/cardiology setting rather than an interventional radiology framework and therefore did not meet the predefined eligibility criteria for this review. |
| Ansaripour A, Moloney E, Branagan-Harris M, et al. Digital variance angiography in patients undergoing lower limb arterial recanalization: cost-effectiveness analysis within the English healthcare setting. J Comp Eff Res. 2024:e230068. https://doi.org/10.57264/cer-2023-0068 | Ineligible study design. This article was a cost-effectiveness and health economic modeling study evaluating digital variance angiography during lower limb arterial recanalization procedures. Although the analysis considered contrast-associated acute kidney injury and radiation-related outcomes, it did not report original patient-level clinical outcome data and therefore did not meet the predefined eligibility criteria for this review. |
| Antoń B, Małyszko J, Stabiszewski P, et al. Differences in mortality and risk factors, two years after endovascular repair of ruptured abdominal aortic aneurysms – reassessment analysis. Ren Fail. 2024;46(2):2397051. https://doi.org/10.1080/0886022X.2024.2397051 | Wrong procedure specialty/population. This retrospective reassessment study evaluated post-contrast acute kidney injury, chronic kidney disease, and mortality following endovascular repair of ruptured abdominal aortic aneurysms. The procedures were performed within a vascular surgery/endovascular surgery setting rather than an interventional radiology framework and therefore did not meet the predefined eligibility criteria for this review. |
| Antoń B, Nazarewski S, Małyszko J. Kidney function, male gender, and aneurysm diameter are predictors of acute kidney injury in patients with abdominal aortic aneurysms treated endovascularly. Toxins. 2023;15:130. https://doi.org/10.3390/toxins15020130 | Wrong procedure specialty/population. This retrospective study evaluated post-contrast acute kidney injury in patients undergoing endovascular aneurysm repair for abdominal aortic aneurysms. The procedures were performed within a vascular surgery/endovascular surgery setting rather than an interventional radiology framework and therefore did not meet the predefined eligibility criteria for this review. |
| Argalious MY, Dalton JE, Cywinski JB, et al. Association between preoperative statin therapy and postoperative change in glomerular filtration rate in endovascular aortic surgery. Br J Anaesth. 2012;109(2):161–167. https://doi.org/10.1093/bja/aes143 | Wrong procedure specialty/population. This cohort study evaluated postoperative renal function decline and contrast-induced nephropathy in patients undergoing endovascular aortic repair. The procedures were performed within a vascular surgery/endovascular aortic surgery setting rather than an interventional radiology framework and therefore did not meet the predefined eligibility criteria for this review. |
| Aru RG, Porez F, Le Houérou T, et al. Branched endovascular aortic repair of chronic post-dissection thoracoabdominal aortic aneurysms: an institutional experience on preoperative planning, intraoperative execution, and pitfalls. J Cardiovasc Surg. 2025;66(3):178–193. https://doi.org/10.23736/S0021-9509.25.13325-9 | Wrong procedure specialty/population and ineligible outcomes. This study evaluated technical planning, procedural execution, and postoperative outcomes of branched endovascular repair for chronic post-dissection thoracoabdominal aortic aneurysms within a vascular surgery/endovascular aortic surgery setting. Although acute kidney injury was reported as a postoperative complication, the study did not primarily investigate contrast-associated acute kidney injury/contrast-induced nephropathy following interventional radiology procedures and therefore did not meet the predefined eligibility criteria for this review. |
| Aykaç Ö, Özakın E, Özdemir AÖ, et al. The effect of contrast agent used during vascular imaging and endovascular treatment on nephropathy and prognosis in acute stroke patients admitted to the emergency department. Turk J Cerebrovasc Dis. 2023;29(2):68–76. https://doi.org/10.5505/tbdhd.2023.75437 | Wrong procedure specialty/population. This retrospective study evaluated contrast-associated nephropathy in patients undergoing computed tomography angiography and endovascular treatment for acute ischemic stroke within a neurovascular stroke/interventional neuroradiology setting. The procedures were not performed within the predefined interventional radiology framework of this review and therefore did not meet the eligibility criteria. |
| Azzalini L. Contrast-induced kidney injury: acute, but often with everlasting consequences. Cardiovasc Revasc Med. 2020. https://doi.org/10.1016/j.carrev.2020.01.003 | Ineligible study design and wrong procedure specialty/population. This article is an editorial/commentary discussing contrast-induced acute kidney injury following coronary angiography and percutaneous coronary intervention in cardiology populations. It was not an original clinical study and did not involve interventional radiology procedures, and therefore did not meet the predefined eligibility criteria for this review. |
| Babu M, Bansal D, Mehta SB, et al. Contrast-induced nephropathy in urological imaging: a comparison with cardiology interventions. Indian J Urol. 2017;33(3):241–245. https://doi.org/10.4103/iju.IJU_328_16 | Wrong procedure specialty/population. This prospective study evaluated contrast-induced nephropathy following urological imaging procedures and compared outcomes with cardiology interventions. The included procedures were predominantly diagnostic urological imaging studies rather than interventional radiology procedures and therefore did not meet the predefined eligibility criteria for this review. |
| Bafna AA, Shah HC. Remote ischemic preconditioning for prevention of contrast-induced nephropathy – A randomized control trial. Indian Heart J. 2020;72:244–247. https://doi.org/10.1016/j.ihj.2020.04.010 | Wrong procedure specialty/population. This randomized controlled trial evaluated remote ischemic preconditioning for prevention of contrast-induced nephropathy in patients undergoing coronary angiography and percutaneous coronary intervention procedures. The procedures were performed in an interventional cardiology setting rather than interventional radiology and therefore did not meet the predefined eligibility criteria for this review. |
| Bagshaw SM, Ghali WA. Theophylline for prevention of contrast-induced nephropathy: a systematic review and meta-analysis. Arch Intern Med. 2005;165:1087–1093. http://doi.org/10.1001/archinte.165.10.1087 | Ineligible study design and wrong procedure specialty/population. This article is a systematic review and meta-analysis evaluating theophylline for prevention of contrast-induced nephropathy in patients undergoing angiography or computed tomography procedures, predominantly within cardiology and diagnostic imaging settings. It was not an original clinical study and did not specifically evaluate interventional radiology procedures, and therefore did not meet the predefined eligibility criteria for this review. |
| Barbieri L, Verdoia M, Schaffer A, et al. The role of statins in the prevention of contrast induced nephropathy: a meta-analysis of 8 randomized trials. J Thromb Thrombolysis. 2014;38:493–502. https://doi.org/10.1007/s11239-014-1076-3 | Ineligible study design and wrong procedure specialty/population. This article is a meta-analysis of randomized trials evaluating statin therapy for prevention of contrast-induced nephropathy in patients undergoing coronary angiography and percutaneous coronary intervention procedures. It was not an original clinical study and did not involve interventional radiology procedures, and therefore did not meet the predefined eligibility criteria for this review. |
| Barbieri L, Verdoia M, Schaffer A, et al. Uric acid levels and the risk of contrast induced nephropathy in patients undergoing coronary angiography or PCI. Nutr Metab Cardiovasc Dis. 2015;25:181–186. https://doi.org/10.1016/j.numecd.2014.08.008 | Wrong procedure specialty/population. This observational study evaluated the association between serum uric acid levels and contrast-induced nephropathy in patients undergoing coronary angiography and percutaneous coronary intervention procedures. The procedures were performed in an interventional cardiology setting rather than interventional radiology and therefore did not meet the predefined eligibility criteria for this review. |
| Baxter M, Gooday C, Dhatariya K. The effect of radiological contrast media on renal function and inflammatory markers in people with diabetes – a clinical study and review. Br J Diabetes Vasc Dis. 2015;15:187–191. https://doi.org/10.15277/bjdvd.2015.040 | Wrong procedure specialty/population. This retrospective study evaluated renal function and inflammatory markers following lower limb angiography in patients with diabetes. The included procedures were predominantly diagnostic vascular imaging studies rather than therapeutic interventional radiology procedures and therefore did not meet the predefined eligibility criteria for this review. |
| Bei WJ, Chen SQ, Li HL, et al. Comparing common doses (double-dose vs usual-dose) of atorvastatin for preventing contrast-induced acute kidney injury and mortality after coronary angiography. Medicine (Baltimore). 2017;96(30):e7501. https://doi.org/10.1097/MD.0000000000007501 | Wrong procedure specialty/population. This prospective observational study evaluated the effect of different atorvastatin doses on contrast-induced acute kidney injury in patients undergoing coronary angiography and percutaneous coronary intervention procedures. The procedures were performed in an interventional cardiology setting rather than interventional radiology and therefore did not meet the predefined eligibility criteria for this review. |
| Belabbas D, Koch C, Chaudru S, et al. Effects of remote ischemic pre-conditioning to prevent contrast-induced nephropathy after intravenous contrast medium injection: a randomized controlled trial. Korean J Radiol. 2020;21(11):1230–1238. https://doi.org/10.3348/kjr.2019.0916 | Wrong procedure specialty/population. This randomized controlled trial evaluated remote ischemic pre-conditioning for prevention of contrast-induced nephropathy in patients undergoing contrast-enhanced computed tomography and coronary angiography during transcatheter aortic valve implantation assessment. The procedures were performed within cardiovascular/cardiology imaging settings rather than interventional radiology and therefore did not meet the predefined eligibility criteria for this review. |
| Bell RM, Rear R, Cunningham J, et al. Effect of remote ischaemic conditioning on contrast-induced nephropathy in patients undergoing elective coronary angiography (ERICCIN): rationale and study design of a randomised single-centre, double-blind placebo-controlled trial. Clin Res Cardiol. 2014;103:203–209. https://doi.org/10.1007/s00392-013-0637-3 | Ineligible study design and wrong procedure specialty/population. This article describes the rationale and study design of the ERICCIN trial evaluating remote ischemic conditioning in patients undergoing coronary angiography and percutaneous coronary intervention procedures. It was not a completed original clinical outcomes study and involved an interventional cardiology population rather than interventional radiology procedures, and therefore did not meet the predefined eligibility criteria for this review. |
| Benini A, Scarsini R, Pesarini G, et al. Early small creatinine shift predicts contrast-induced acute kidney injury and persistent renal damage after percutaneous coronary procedures. Cardiovasc Revasc Med. 2020;21:305–311. https://doi.org/10.1016/j.carrev.2019.05.021 | Wrong procedure specialty/population. This retrospective cohort study evaluated contrast-induced acute kidney injury and persistent renal damage in patients undergoing coronary angiography and percutaneous coronary intervention procedures. The procedures were performed in an interventional cardiology setting rather than interventional radiology and therefore did not meet the predefined eligibility criteria for this review. |
| Benjamens S, Yakar D, Slart RHJA, et al. The fear for contrast-induced nephropathy in kidney transplant recipients: time for a paradigm shift? Transpl Int. 2018;31:1050–1051. https://doi.org/10.1111/tri.13277 | Ineligible study design. This article is a letter/editorial discussing contrast-induced nephropathy risk and prophylaxis strategies in kidney transplant recipients undergoing contrast-enhanced diagnostic and interventional procedures. It was not an original clinical study and therefore did not meet the predefined eligibility criteria for this review. |
| Bernardi MH, Haider DG, Domenig CM, et al. Does the choice of intraoperative fluid modify abdominal aneurysm repair outcomes? A cohort analysis. Medicine (Baltimore). 2019;98(28):e16387. https://doi.org/10.1097/MD.0000000000016387 | Wrong procedure specialty/population. This retrospective cohort study evaluated postoperative acute kidney injury and mortality following open abdominal aortic aneurysm repair and endovascular aneurysm repair procedures. The procedures were performed within a vascular surgery/endovascular surgery setting rather than interventional radiology and therefore did not meet the predefined eligibility criteria for this review. |
| Bhatt S, Rajpal N, Rathi V, et al. Contrast induced nephropathy with intravenous iodinated contrast media in routine diagnostic imaging: an initial experience in a tertiary care hospital. Radiol Res Pract. 2016;2016:8792984. https://doi.org/10.1155/2016/8792984 | Wrong procedure specialty/population. This prospective observational study evaluated contrast-induced nephropathy following routine intravenous contrast-enhanced computed tomography and intravenous urography examinations. The included procedures were diagnostic imaging studies rather than interventional radiology procedures and therefore did not meet the predefined eligibility criteria for this review. |
| Bianco M, Mottola FF, Cerrato E, et al. Acute coronary syndrome in very elderly patients—a real-world experience. Heart Vessels. 2023;38:1019–1027. https://doi.org/10.1007/s00380-023-02260-x | Wrong procedure specialty/population. This retrospective observational study evaluated treatment strategies and outcomes in very elderly patients with acute coronary syndromes undergoing coronary angiography and percutaneous coronary intervention. The procedures were performed in an interventional cardiology setting rather than interventional radiology and therefore did not meet the predefined |
| Biernacka-Fiałkowska B, Szuksztul M, Suślik W, et al. Intravenous N-acetylcysteine for the prevention of contrast-induced nephropathy – a prospective, single-center, randomized, placebo-controlled trial. The INPROC trial. Adv Interv Cardiol. 2018;14(1):59–66. https://doi.org/10.5114/aic.2018.74356 | Wrong procedure specialty/population. This randomized placebo-controlled trial evaluated intravenous N-acetylcysteine for prevention of contrast-induced nephropathy in patients undergoing coronary and peripheral angiography and angioplasty procedures, predominantly within an interventional cardiology setting. The study did not specifically evaluate inter |
| Bilen MN, Demiröz Ö, Çetin İ, et al. Association between systolic pulmonary artery pressure and contrast-induced nephropathy in patients with ST-segment elevation myocardial infarction. Acta Cardiol Sin. 2024;40:292–299. https://doi.org/10.6515/ACS.202405_40(3).20240129B | Wrong procedure specialty/population. This retrospective study evaluated contrast-induced nephropathy in patients with ST-segment elevation myocardial infarction undergoing primary percutaneous coronary intervention procedures. The procedures were performed in an interventional cardiology setting rather than interventional radiology and therefore did not meet the predefined eligibility criteria for this review. |
| Bithal PK. Contrast-induced nephropathy in neurological patients: a crying need for prospective randomized controlled trials. J Neuroanaesthesiol Crit Care. 2019;6:1–2. https://doi.org/10.1055/s-0039-1679196 | Ineligible study design. This article is an editorial discussing contrast-induced nephropathy in neurological patients undergoing neurointerventional and angiographic procedures. It was not an original clinical study and therefore did not meet the predefined eligibility criteria for this review. |
| Bjørn M, Kunkel JB, Helgestad O, et al. Long-term outcomes after acute kidney injury in myocardial infarction complicated by cardiogenic shock: a retrospective, observational study. Eur Heart J Acute Cardiovasc Care. 2025;14:385–391. https://doi.org/10.1093/ehjacc/zuaf048 | Wrong procedure specialty/population. This retrospective observational study evaluated acute kidney injury and long-term renal outcomes in patients with acute myocardial infarction complicated by cardiogenic shock, most of whom underwent coronary angiography and percutaneous coronary intervention. The procedures were performed in an interventional cardiology setting rather than interventional radiology and therefore did not meet the predefined eligibility criteria for this review. |
| Boccalandro F, Shreyder K, Harmon L, et al. Five-year follow-up of patients with radio-contrast-induced acute renal injury: can intravenous sodium bicarbonate improve long-term outcomes? Cardiovasc Revasc Med. 2021;31:61–68. https://doi.org/10.1016/j.carrev.2020.11.017 | Wrong procedure specialty/population. This prospective randomized study evaluated radiocontrast-induced acute kidney injury and long-term renal outcomes in patients with chronic kidney disease undergoing elective coronary angiography and percutaneous coronary intervention procedures. The procedures were performed in an interventional cardiology setting rather than interventional radiology and therefore did not meet the predefined eligibility criteria for this review. |
| Boccuto F, Carabetta N, Cacia MA, et al. Clinical impact of cerebral protection during transcatheter aortic valve implantation. Eur J Clin Invest. 2024;54:e14166. https://doi.org/10.1111/eci.14166 | Ineligible study design and wrong procedure specialty/population. This article is a systematic review and meta-analysis evaluating cerebral embolic protection devices during transcatheter aortic valve implantation procedures. It was not an original clinical study and involved structural/interventional cardiology procedures rather than interventional radiology procedures, and therefore did not meet the predefined eligibility criteria for this review. |
| Boucek P, Havrdova T, Oliyarnyk O, et al. Prevention of contrast-induced nephropathy in diabetic patients with impaired renal function: a randomized, double blind trial of sodium bicarbonate versus sodium chloride-based hydration. Diabetes Res Clin Pract. 2013;101:303–308. https://doi.org/10.1016/j.diabres.2013.05.015 | Wrong procedure specialty/population. This randomized double-blind trial evaluated prevention of contrast-induced nephropathy in diabetic patients with renal impairment undergoing elective coronary angiography and percutaneous coronary intervention procedures. The procedures were performed in an interventional cardiology setting rather than interventional radiology and therefore did not meet the predefined eligibility criteria for this review. |
| Boukantar M, Chiaroni PM, Gallet R, et al. A randomized controlled trial of nonfasting vs fasting before interventional coronary procedures: the TONIC trial. JACC Cardiovasc Interv. 2024;17:1200–1210. https://doi.org/10.1016/j.jcin.2024.03.033 | Wrong procedure specialty/population and ineligible outcomes. This randomized controlled trial evaluated fasting versus nonfasting strategies before coronary angiography and percutaneous coronary intervention procedures. Although contrast-induced acute kidney injury was reported as a secondary safety outcome, the study was conducted in an interventional cardiology setting and did not primarily investigate contrast-associated acute kidney injury following interventional radiology procedures, and therefore did not meet the predefined eligibility |
| Braet DJ, Graham NJ, Albright J, et al. A novel preoperative risk assessment tool to identify patients at risk of contrast-associated acute kidney injury after endovascular abdominal aortic aneurysm repair. Ann Vasc Surg. 2023;93:79–91. https://doi.org/10.1016/j.avsg.2023.02.017 | Wrong procedure specialty/population. This retrospective cohort study evaluated contrast-associated acute kidney injury following endovascular abdominal aortic aneurysm repair procedures. The procedures were performed within a vascular surgery/endovascular aortic surgery setting rather than interventional radiology and therefore did not meet the predefined eligibility criteria for this review. |
| Briguori C, D’Amore C, De Micco F, et al. Left ventricular end-diastolic pressure versus urine flow rate–guided hydration in preventing contrast-associated acute kidney injury. JACC Cardiovasc Interv. 2020;13:2065–2074. https://doi.org/10.1016/j.jcin.2020.04.051 | Wrong procedure specialty/population. This randomized multicenter trial evaluated hydration strategies for prevention of contrast-associated acute kidney injury in patients undergoing coronary and peripheral angiography or angioplasty procedures, predominantly within an interventional cardiology setting. The study did not specifically evaluate interventional radiology procedures according |
| Briguori C, Manganelli F, Scarpato P, et al. Acetylcysteine and contrast agent-associated nephrotoxicity. J Am Coll Cardiol. 2002;40:298–303. https://doi.org/10.1016/S0735-1097(02)01958-7 | Wrong procedure specialty/population. This randomized study evaluated acetylcysteine for prevention of contrast-associated nephrotoxicity in patients undergoing coronary and peripheral angiography and angioplasty procedures, predominantly within an interventional cardiology setting. The study did not specifically evaluate interventional radiology procedures according to the predefined eligibility criteria for this review. |
| Briguori C, Airoldi F, D’Andrea D, et al. Renal insufficiency following contrast media administration trial (REMEDIAL): a randomized comparison of 3 preventive strategies. Circulation. 2007;115:1211–1217. https://doi.org/10.1161/CIRCULATIONAHA.106.687152 | Wrong procedure specialty/population. This randomized trial evaluated preventive strategies for contrast-induced nephrotoxicity in patients with chronic kidney disease undergoing coronary and peripheral angiography or angioplasty procedures, predominantly within an interventional cardiology setting. The study did not specifically evaluate interventional radiology procedures according to the predefined eligibility criteria for this review. |
| Briguori C, Visconti G, Rivera NV, et al. Cystatin C and contrast-induced acute kidney injury. Circulation. 2010;121:2117–2122. https://doi.org/10.1161/CIRCULATIONAHA.109.919639 | Wrong procedure specialty/population. This prospective study evaluated cystatin C as an early biomarker of contrast-induced acute kidney injury in patients with chronic kidney disease undergoing coronary and peripheral angiography or angioplasty procedures, predominantly within an interventional cardiology setting. The study did not specifically evaluate interventional radiology procedures according to the predefined eligibility criteria for this review. |
| Brinjikji W, Demchuk AM, Murad MH, et al. Neurons over nephrons: systematic review and meta-analysis of contrast-induced nephropathy in patients with acute stroke. Stroke. 2017;48:1862–1868. https://doi.org/10.1161/STROKEAHA.117.016771 | Ineligible study design and wrong procedure specialty/population. This article is a systematic review and meta-analysis evaluating acute kidney injury after computed tomographic angiography and perfusion imaging in patients with acute ischemic stroke. The included procedures were diagnostic neurovascular imaging studies rather than interventional radiology procedures, and the article was not an original clinical study, and therefore did not meet the predefined eligibility criteria for this review. |
| Brinkert M, de Boeck B, Petryka J, et al. Minimal use of contrast media in patients with severe chronic kidney disease undergoing transcatheter aortic valve implantation. Cardiovasc Med. 2019;22:w02021. https://doi.org/10.4414/cvm.2019.02021 | Wrong procedure specialty/population. This observational study evaluated strategies to minimize contrast media exposure and acute kidney injury in patients with severe chronic kidney disease undergoing transcatheter aortic valve implantation procedures. The procedures were performed within a structural/interventional cardiology setting rather than interventional radiology and therefore did not meet the predefined eligibility criteria for this review. |
| Bruno RR, Nia AM, Wolff G, et al. Early clinical experiences with a novel contrast volume reduction system during invasive coronary angiography. IJC Heart Vasc. 2019;23:100377. https://doi.org/10.1016/j.ijcha.2019.100377 | Wrong procedure specialty/population. This retrospective pilot study evaluated a contrast volume reduction system in patients undergoing invasive coronary angiography and percutaneous coronary intervention procedures. The procedures were performed in an interventional cardiology setting rather than interventional radiology and therefore did not meet the predefined eligibility criteria for this review. |
| Burchardt P, Rzezniczak J, Synowiec T, et al. Lack of serum creatinine decrease after coronary angiography despite prophylactic hydration after routine coronary angiography/angioplasty in stable angina patients – pilot study. Kidney Blood Press Res. 2016;41:222–230. https://doi.org/10.1159/000443425 | Wrong procedure specialty/population. This retrospective pilot study evaluated early renal dysfunction and serum creatinine changes following routine coronary angiography and coronary angioplasty procedures in patients with stable angina. The procedures were performed in an interventional cardiology setting rather than interventional radiology and therefore did not meet the predefined eligibility criteria for this review. |
| Busutti M, Sensoni A, Vacirca A, et al. Renal benefits of CO2 as a contrast media for EVAR procedures: new perspectives on 1 year outcomes. J Endovasc Ther. 2024;31:1180–1189. https://doi.org/10.1177/15266028231162258 | Wrong procedure specialty/population. This retrospective observational study evaluated renal outcomes and post-contrast acute kidney injury following endovascular aneurysm repair procedures using carbon dioxide versus iodinated contrast media. The procedures were performed in a vascular surgery/endovascular aortic repair setting rather than interventional radiology and therefore did not meet the predefined eligibility criteria for this review. |
| Calabrò P, Bianchi R, Crisci M, et al. Use and efficacy of saline hydration and N-acetyl cysteine to prevent contrast-induced nephropathy in low-risk populations undergoing coronary artery angiography. Intern Emerg Med. 2011;6:503–507. https://doi.org/10.1007/s11739-011-0513-y | Wrong procedure specialty/population. This retrospective study evaluated prevention of contrast-induced nephropathy in low-risk patients undergoing coronary artery angiography and angioplasty procedures. The procedures were performed in an interventional cardiology setting rather than interventional radiology and therefore did not meet the predefined eligibility criteria for this review. |
| Cetinkaya Z, Kelesoglu S. The role of pan-immune-inflammation value in predicting contrast-induced nephropathy development in patients undergoing percutaneous coronary intervention due to NSTEMI. Angiology. 2025;76:281–288. https://doi.org/10.1177/00033197231211107 | Wrong procedure specialty/population. This retrospective study evaluated inflammatory predictors of contrast-induced nephropathy in patients with non-ST segment elevation myocardial infarction undergoing percutaneous coronary intervention procedures. The procedures were performed in an interventional cardiology setting rather than interventional radiology and therefore did not meet the predefined eligibility criteria for this review. |
| Chalikias G, Drosos I, Tziakas DN. Prevention of contrast-induced acute kidney injury: an update. Cardiovasc Drugs Ther. 2016;30:515–524. https://doi.org/10.1007/s10557-016-6683-0 | Ineligible study design. This article is a narrative review summarizing preventive strategies for contrast-induced acute kidney injury across coronary angiography, percutaneous coronary intervention, and other contrast-based procedures. It was not an original clinical study and therefore did not meet the predefined eligibility criteria for this review. |
| Chang WT, Sun CK, Wu JY, et al. Association of prognostic nutritional index with risk of contrast induced nephropathy: A meta-analysis. Front Nutr. 2023;10:1154409. https://doi.org/10.3389/fnut.2023.1154409 | Ineligible study design. This article is a systematic review and meta-analysis evaluating the association between prognostic nutritional index and contrast-induced nephropathy in patients undergoing coronary angiography or percutaneous coronary intervention. It was not an original clinical study and therefore did not meet the predefined eligibility criteria for this review. |
| Chatterjee S, Kundu A, Mukherjee D, et al. Risk of contrast-induced acute kidney injury in ST-elevation myocardial infarction patients undergoing multi-vessel intervention—meta-analysis of randomized trials and risk prediction modeling study using observational data. Catheter Cardiovasc Interv. 2017;90:205–212. https://doi.org/10.1002/ccd.26928 | Ineligible study design and wrong procedure specialty/population. This article included a meta-analysis of randomized trials and an observational database analysis evaluating contrast-induced acute kidney injury in patients with ST-elevation myocardial infarction undergoing multivessel percutaneous coronary intervention. The procedures were performed in an interventional cardiology setting rather than interventional radiology, and the article did not constitute a primary interventional radiology clinical study according to the predefined eligibility criteria for this review. |
| Chaudhury P, Armanyous S, Harb SC, et al. Intra-arterial versus intravenous contrast and renal injury in chronic kidney disease: A propensity-matched analysis. Nephron. 2019;141:1–10. https://doi.org/10.1159/000494047 | Wrong procedure specialty/population. This propensity-matched observational study evaluated acute kidney injury after intravenous contrast-enhanced CT and coronary angiography in patients with chronic kidney disease. The included procedures were diagnostic radiology and interventional cardiology procedures rather than interventional radiology procedures, and therefore did not meet the predefined eligibility criteria for this review. |
| Chen YW, Su CS, Chang WC, et al. Feasibility and clinical outcomes of rotational atherectomy for heavily-calcified side branches of complex coronary bifurcation lesions in the real-world practice of the drug-eluting stent era. J Interv Cardiol. 2018;31:486–495. https://doi.org/10.1111/joic.12515 | Wrong procedure specialty/population and ineligible outcomes. This retrospective observational study evaluated rotational atherectomy for heavily calcified coronary bifurcation lesions in patients undergoing complex percutaneous coronary intervention procedures. Although acute contrast-induced nephropathy was reported as a procedural complication, the study primarily focused on interventional cardiology techniques and procedural outcomes rather than contrast-associated acute kidney injury following interventional radiology procedures, and therefore did not meet the predefined eligibility criteria for this review. |
| Chen K, Wang D, Fang M, et al. Predictive value of serum CIRP in acute kidney injury after total aortic arch replacement. J Card Surg. 2024;2024:4814978. https://doi.org/10.1155/2024/4814978 | Wrong procedure specialty/population and ineligible outcomes. This retrospective study evaluated postoperative acute kidney injury after total aortic arch replacement surgery in patients with acute Stanford type A aortic dissection. The study involved open cardiovascular surgery under hypothermic circulatory arrest rather than interventional radiology procedures or iodinated contrast-associated acute kidney injury, and therefore did not meet the predefined eligibility criteria for this review. |
| Chen HY, Ou SH, Chou KJ, et al. Prophylactic hemodialysis following coronary angiography and one-year outcomes in non-dialysis patients with chronic kidney disease: A propensity-matched study. J Formos Med Assoc. 2021;120:629–640. https://doi.org/10.1016/j.jfma.2020.07.020 | Wrong procedure specialty/population. This propensity-matched cohort study evaluated prophylactic hemodialysis after coronary angiography in patients with chronic kidney disease undergoing coronary angiography and percutaneous coronary intervention procedures. The procedures were performed in an interventional cardiology setting rather than interventional radiology and therefore did not meet the predefined eligibility criteria for this review. |
| Chen SY, Scadeng M. Systematic review of intravenous contrast-induced nephropathy and prophylaxis with hydration. J Med Imaging Radiat Oncol. 2026;70:57–72. https://doi.org/10.1111/1754-9485.70051 | Ineligible study design and wrong procedure specialty/population. This article is a systematic review evaluating contrast-associated acute kidney injury following intravenous iodinated contrast administration for computed tomography imaging and prophylactic hydration strategies. The study focused on diagnostic radiology CT imaging rather than interventional radiology procedures and was not an original clinical study, and therefore did not meet the predefined eligibility criteria for this review. |
| Chen TW, Huang CY, Chen PL, et al. Endovascular and hybrid revascularization for complicated aorto-iliac occlusive disease: short-term results in single institute experience. Acta Cardiol Sin. 2018;34:313–320. https://doi.org/10.6515/ACS.201807_34(4).20180301A | Wrong procedure specialty/population and ineligible outcomes. This retrospective study evaluated endovascular and hybrid revascularization procedures for complicated aortoiliac occlusive disease within a vascular surgery/endovascular reconstruction setting. Although acute kidney injury was reported as a postoperative complication, the study primarily focused on vascular patency and revascularization outcomes rather than contrast-associated acute kidney injury following interventional radiology procedures, and therefore did not meet the predefined eligibility criteria for this review. |
| Cheng EL, Hong Q, Yong E, et al. Validating the use of contrast-induced nephropathy prediction models in endovascular aneurysm repairs. J Vasc Surg. 2020;71:1546–1553. https://doi.org/10.1016/j.jvs.2019.07.093 | Wrong procedure specialty/population. This retrospective study evaluated contrast-induced nephropathy prediction models in patients undergoing thoracic and abdominal endovascular aneurysm repair procedures within a vascular surgery/endovascular aneurysm repair setting. The study did not specifically evaluate interventional radiology procedures according to the predefined eligibility criteria for this review. |
| Chillo P, Malaja N, Kisenge P. Magnitude and associated factors of contrast induced nephropathy among patients undergoing coronary angiography and interventions at a cardiac referral hospital in Tanzania - a cross-sectional study. Pan Afr Med J. 2021;38:311. https://doi.org/10.11604/pamj.2021.38.311.24536 | Wrong procedure specialty/population. This cross-sectional study evaluated contrast-induced nephropathy among patients undergoing coronary angiography and percutaneous coronary intervention procedures in a cardiac referral center. The procedures were performed in an interventional cardiology setting rather than interventional radiology and therefore did not meet the predefined eligibility criteria for this review. |
| Cho R, Javed N, Traub D, et al. Oral hydration and alkalinization is noninferior to intravenous therapy for prevention of contrast-induced nephropathy in patients with chronic kidney disease. J Interv Cardiol. 2010;23:460–466. https://doi.org/10.1111/j.1540-8183.2010.00585.x | Wrong procedure specialty/population. This prospective randomized study evaluated oral versus intravenous hydration strategies for prevention of contrast-induced nephropathy in patients with chronic kidney disease undergoing elective coronary angiography procedures. The procedures were performed in an interventional cardiology setting rather than interventional radiology and therefore did not meet the predefined eligibility criteria for this review. |
| Choi MJ, Yoon JW, Han SJ, et al. The prevention of contrast-induced nephropathy by simultaneous hemofiltration during coronary angiographic procedures: A comparison with periprocedural hemofiltration. Int J Cardiol. 2014;176:941–945. https://doi.org/10.1016/j.ijcard.2014.08.095 | Wrong procedure specialty/population. This randomized pilot study evaluated hemofiltration strategies for prevention of contrast-induced nephropathy in chronic kidney disease patients undergoing coronary angiography procedures. The procedures were performed in an interventional cardiology setting rather than interventional radiology and therefore did not meet the predefined eligibility criteria for this review. |
| Chusiri S, Chutinet A, Suwanwela NC, Puttilerpong C. Incidence and risk factors of postcontrast acute kidney injury in patients with acute ischemic stroke. Stroke Res Treat. 2020;2020:7182826. https://doi.org/10.1155/2020/7182826 | Wrong procedure specialty/population. This retrospective study evaluated postcontrast acute kidney injury in patients with acute ischemic stroke undergoing multimodal computed tomography imaging and thrombolytic treatment. The study primarily focused on diagnostic neuroradiology imaging procedures rather than interventional radiology procedures according to the predefined eligibility criteria for this review. |
| Cichoń M, Wybraniec MT, Okoń O, et al. Repeated dose of contrast media and the risk of contrast-induced acute kidney injury in a broad population of patients hospitalized in cardiology department. J Clin Med. 2023;12:2166. https://doi.org/10.3390/jcm12062166 | Wrong procedure specialty/population. This retrospective study evaluated the risk of contrast-induced acute kidney injury in patients hospitalized in a cardiology department undergoing coronary angiography, percutaneous coronary intervention, and computed tomography procedures with repeated contrast exposure. The procedures were predominantly performed in an interventional cardiology setting rather than interventional radiology and therefore did not meet the predefined eligibility criteria for this review. |
| Cinteza E, Balgradean M, Filip C, et al. Iodinated contrast media in pediatric cardiac angiography: nephrotoxic risk evaluation. Rev Chim (Bucharest). 2018;69:511–514. https://doi.org/10.37358/RC.18.2.6137 | Wrong procedure specialty/population and ineligible outcomes. This retrospective study evaluated nephrotoxic risk associated with iodinated contrast media in pediatric cardiac angiography and interventional cardiology procedures in congenital heart disease patients. The procedures were performed in a pediatric/interventional cardiology setting rather than interventional radiology, and no contrast-induced nephropathy events were reported, therefore the study did not meet the predefined eligibility criteria for this review. |
| Cobb KD, Gomella PT, DiBianco JM, et al. Are emergently placed nephrostomy tubes suitable for subsequent percutaneous endoscopic renal surgery? Urology. 2019;126:45–48. https://doi.org/10.1016/j.urology.2019.01.006 | Ineligible outcomes. This retrospective study evaluated the utility of emergently placed nephrostomy tubes for subsequent percutaneous nephrolithotomy or antegrade ureteroscopy procedures in patients with nephrolithiasis. Although acute kidney injury was an indication for emergent nephrostomy placement in some patients, the study did not evaluate contrast-associated acute kidney injury or contrast nephrotoxicity outcomes following interventional radiology procedures and therefore did not meet the predefined eligibility criteria for this review. |
| Corazza I, Taglieri N, Pirazzini E, et al. Carbon dioxide coronary angiography: A mechanical feasibility study with a cardiovascular simulator. AIP Adv. 2018;8:015225. https://doi.org/10.1063/1.5016601 | Ineligible study design and wrong procedure specialty/population. This experimental bench simulation study evaluated the mechanical feasibility of carbon dioxide coronary angiography using a cardiovascular simulator model. The study did not involve human interventional radiology procedures or clinical contrast-associated acute kidney injury outcomes, and therefore did not meet the predefined eligibility criteria for this review. |
| Coryell L, Lott JP, Stavropoulos SW, et al. The case for primary placement of tunneled hemodialysis catheters in acute kidney injury. J Vasc Interv Radiol. 2009;20:1578–1581. https://doi.org/10.1016/j.jvir.2009.08.014 | Ineligible outcomes. This retrospective interventional radiology study evaluated tunneled hemodialysis catheter placement and renal recovery in patients with acute kidney injury requiring hemodialysis access. Although the procedures were performed in an interventional radiology setting, the study did not evaluate contrast-associated acute kidney injury or iodinated contrast-related nephrotoxicity outcomes and therefore did not meet the predefined eligibility criteria for this review. |
| Cossette F, Trifan A, Prévost-Marcotte G, et al. Tailored hydration for the prevention of contrast-induced acute kidney injury after coronary angiogram or PCI: A systematic review and meta-analysis. Am Heart J. 2025;282:93–102. https://doi.org/10.1016/j.ahj.2025.01.002 | Ineligible study design and wrong procedure specialty/population. This article is a systematic review and meta-analysis evaluating tailored hydration strategies for prevention of contrast-induced acute kidney injury in patients undergoing coronary angiography and percutaneous coronary intervention procedures. The included procedures were predominantly performed in an interventional cardiology setting rather than interventional radiology, and the article was not an original clinical study according to the predefined eligibility criteria for this review. |
| Crimi G, Leonardi S, Costa F, et al. Incidence, prognostic impact, and optimal definition of contrast-induced acute kidney injury in consecutive patients with stable or unstable coronary artery disease undergoing percutaneous coronary intervention: insights from the all-comer PRODIGY trial. Catheter Cardiovasc Interv. 2015;86:E19–E27. https://doi.org/10.1002/ccd.25822 | Wrong procedure specialty/population. This post hoc analysis of the PRODIGY trial evaluated contrast-induced acute kidney injury following percutaneous coronary intervention in patients with stable coronary artery disease or acute coronary syndromes. The procedures were performed in an interventional cardiology setting rather than interventional radiology and therefore did not meet the predefined eligibility criteria for this review. |
| Cui X, Xie B, Wang H, et al. Preventing contrast-induced acute kidney injury with probucol and hydration in patients with coronary heart disease: A systematic review and meta-analysis of randomized controlled trials. Medicine (Baltimore). 2023;102:e33273. https://doi.org/10.1097/MD.0000000000033273 | Ineligible study design and wrong procedure specialty/population. This article is a systematic review and meta-analysis evaluating probucol plus hydration for prevention of contrast-induced acute kidney injury in patients undergoing coronary angiography and percutaneous coronary intervention procedures. The included procedures were predominantly performed in an interventional cardiology setting rather than interventional radiology, and the article was not an original clinical study according to the predefined eligibility criteria for this review. |
| Cury MVM, Matielo MF, Brochado Neto FC, et al. The incidence, risk factors, and outcomes of contrast-induced nephropathy in patients with critical limb ischemia following lower limb angiography. Angiology. 2018;69:700–708. https://doi.org/10.1177/0003319718754984 | Wrong procedure specialty/population. This prospective observational study evaluated contrast-induced nephropathy following lower limb angiography and endovascular treatment in patients with critical limb ischemia. The study population primarily represented peripheral vascular intervention and vascular surgery/endovascular practice rather than predefined interventional radiology procedural cohorts according to the eligibility criteria for this review. |
| Danışan G, Taydaş O, Özdemir M, et al. Dynamic thiol-disulphide homeostasis as a biomarker for predicting the development of contrast medium-associated acute kidney injury in the endovascular treatment of peripheral arterial disease. Clin Radiol. 2023;78:466–472. https://doi.org/10.1016/j.crad.2023.02.016 | Wrong procedure specialty/population. This randomized controlled study evaluated contrast medium-associated acute kidney injury following endovascular treatment for peripheral arterial disease, including peripheral angiographic and revascularization procedures. The study population primarily represented peripheral vascular intervention and vascular/endovascular practice rather than predefined interventional radiology procedural cohorts according to the eligibility criteria for this review. |
| Demirci G, Şahin AA, Altunova M, et al. The effect of uric acid and albumin ratio in undergoing lower extremity endovascular interventions for peripheral arterial disease related contrast induced nephropathy. Ann Vasc Surg. 2024;108:452–458. https://doi.org/10.1016/j.avsg.2024.05.032 | Wrong procedure specialty/population. This retrospective cohort study evaluated contrast-induced nephropathy following lower extremity endovascular interventions for peripheral arterial disease, including peripheral angioplasty and stenting procedures. The study population primarily represented peripheral vascular intervention and vascular/endovascular practice rather than predefined interventional radiology procedural cohorts according to the eligibility criteria for this review. |
| Diamantopoulos A, Patrone L, Santonocito S, et al. Carbon dioxide angiography during peripheral angioplasty procedures significantly reduces the risk of contrast-induced nephropathy in patients with chronic kidney disease. CVIR Endovasc. 2020;3:9. https://doi.org/10.1186/s42155-020-0103-z | Wrong procedure specialty/population. This prospective cohort study evaluated contrast-induced nephropathy following peripheral angioplasty procedures using carbon dioxide angiography in patients with chronic kidney disease. The study population primarily represented peripheral vascular intervention and vascular/endovascular practice rather than predefined interventional radiology procedural cohorts according to the eligibility criteria for this review. |
| Dong X, Wang B, Chen S, et al. Association between prognostic nutritional index and contrast-associated acute kidney injury in patients complicated with chronic kidney disease and coronary artery disease. J Interv Cardiol. 2021;2021:2274430. https://doi.org/10.1155/2021/2274430 | Wrong procedure specialty/population. This retrospective observational study evaluated the association between prognostic nutritional index and contrast-associated acute kidney injury in patients with chronic kidney disease and coronary artery disease undergoing coronary angiography and percutaneous coronary intervention procedures. The procedures were performed in an interventional cardiology setting rather than interventional radiology and therefore did not meet the predefined eligibility criteria for this review. |
| Dorval JF, Dixon SR, Zelman RB, et al. Feasibility study of the RenalGuard™ balanced hydration system: A novel strategy for the prevention of contrast-induced nephropathy in high risk patients. Int J Cardiol. 2013;166:482–486. https://doi.org/10.1016/j.ijcard.2011.11.035 | Wrong procedure specialty/population. This prospective multicenter feasibility study evaluated the RenalGuard balanced hydration system for prevention of contrast-induced nephropathy in high-risk patients undergoing diagnostic or therapeutic cardiac catheterization procedures. The study population predominantly underwent interventional cardiology procedures rather than interventional radiology procedures according to the predefined eligibility criteria for this review. |
| Du X, Liu R, Guo D, et al. Predictive value of nutritional scores for contrast-induced acute kidney injury in patients with type 2 diabetes mellitus. Angiology. 2026. https://doi.org/10.1177/00033197251399868 | Wrong procedure specialty/population. This retrospective study evaluated the predictive value of nutritional scores for contrast-induced acute kidney injury in patients with type 2 diabetes mellitus undergoing coronary angiography and percutaneous coronary intervention procedures. The procedures were performed in an interventional cardiology setting rather than interventional radiology and therefore did not meet the predefined eligibility criteria for this review. |
| Duan XH, Li YS, Han XW, et al. C-arm CT-guided renal arterial embolisation followed by radiofrequency ablation for treatment of patients with unresectable renal cell carcinoma. Clin Radiol. 2016;71:79–85. https://doi.org/10.1016/j.crad.2015.10.012 | Ineligible outcomes. This retrospective interventional radiology study evaluated C-arm CT-guided renal arterial embolization followed by radiofrequency ablation for unresectable renal cell carcinoma. Although renal function parameters were monitored during follow-up, the study did not specifically evaluate contrast-associated acute kidney injury or contrast nephrotoxicity outcomes following interventional radiology procedures and therefore did not meet the predefined eligibility criteria for this review. |
| Efe SC, Keskin M, Toprak E, et al. A novel risk assessment model using urinary system contrast blush grading to predict contrast-induced acute kidney injury in low-risk profile patients. Angiology. 2021;72:524–532. https://doi.org/10.1177/00033197211005206 | Wrong procedure specialty/population. This observational study evaluated urinary system contrast blush grading for predicting contrast-induced acute kidney injury in patients undergoing coronary angiography and percutaneous coronary intervention procedures. The procedures were performed in an interventional cardiology setting rather than interventional radiology and therefore did not meet the predefined eligibility criteria for this review. |
| Ekeh AP, Khalaf S, Ilyas S, et al. Complications arising from splenic artery embolization: a review of an 11-year experience. Am J Surg. 2013;205:250–254. https://doi.org/10.1016/j.amjsurg.2013.01.003 | Ineligible outcomes. This retrospective study evaluated complications associated with splenic artery embolization for blunt splenic trauma. Although contrast-induced renal insufficiency was reported among procedural complications, the study did not primarily investigate contrast-associated acute kidney injury or contrast nephrotoxicity outcomes following interventional radiology procedures according to the predefined eligibility criteria for this review. |
| Eng J, Wilson RF, Subramaniam RM, et al. Comparative effect of contrast media type on the incidence of contrast-induced nephropathy: a systematic review and meta-analysis. Ann Intern Med. 2016;164:417–424. https://doi.org/10.7326/M15-1402 | Ineligible study design and wrong procedure specialty/population. This article is a systematic review and meta-analysis evaluating the comparative renal safety of different contrast media types in patients undergoing diagnostic and interventional imaging procedures, predominantly coronary angiography and interventional cardiology procedures. The article was not an original clinical study according to the predefined eligibility criteria for this review. |
| Erben Y, Brownstein AJ, Rajaee S, et al. Natural history and management of splanchnic artery aneurysms in a single tertiary referral center. J Vasc Surg. 2018;68:1079–1087. https://doi.org/10.1016/j.jvs.2017.12.057 | Ineligible outcomes. This retrospective study evaluated the natural history and operative management of splanchnic artery aneurysms, including endovascular embolization and open surgical repair. Although postoperative acute kidney injury was reported among complications, the study did not specifically investigate contrast-associated acute kidney injury or contrast nephrotoxicity outcomes following interventional radiology procedures according to the predefined eligibility criteria for this review. |
| Erol T, Tekin A, Katırcıbaşı MT, et al. Efficacy of allopurinol pretreatment for prevention of contrast-induced nephropathy: a randomized controlled trial. Int J Cardiol. 2013;167:1396–1399. https://doi.org/10.1016/j.ijcard.2012.04.068 | Wrong procedure specialty/population. This randomized controlled trial evaluated allopurinol pretreatment for prevention of contrast-induced nephropathy in patients undergoing cardiac catheterization and coronary intervention procedures. The procedures were performed in an interventional cardiology setting rather than interventional radiology and therefore did not meet the predefined eligibility criteria for this review. |
| Ertas F, Avci E, Kiris T. The ratio of fibrinogen to albumin as a predictor of contrast-induced nephropathy after carotid angiography. Angiology. 2019;70:458–464. https://doi.org/10.1177/0003319718809200 | Wrong procedure specialty/population. This retrospective study evaluated contrast-induced nephropathy following elective carotid angiography procedures in patients with carotid artery disease. The study population primarily represented diagnostic carotid angiography cohorts rather than predefined therapeutic interventional radiology or endovascular procedural populations according to the eligibility criteria for this review. |
| Esmaeili Z, Asgarian F, Aghaei Moghadam E, et al. Prevalence, risk factors, and outcomes of acute kidney injury in a pediatric cardiac intensive care unit: a cross-sectional study. Health Sci Rep. 2024;7:e1791. https://doi.org/10.1002/hsr2.1791 | Wrong population/outcomes. This retrospective cross-sectional study evaluated acute kidney injury in a pediatric cardiac intensive care unit population with congenital or acquired heart disease. Although angiography was identified as a risk factor for acute kidney injury, the study did not specifically investigate contrast-associated acute kidney injury or contrast nephrotoxicity following interventional radiology procedures according to the predefined eligibility criteria for this review. |
| Evola S, Lunetta M, Macaione F, et al. Risk factors for contrast induced nephropathy: A study among Italian patients. Indian Heart J. 2012;64:484–491. https://doi.org/10.1016/j.ihj.2012.07.007 | Wrong procedure specialty/population. This observational study evaluated risk factors for contrast-induced nephropathy in patients undergoing coronary angiography and percutaneous coronary intervention procedures. The procedures were performed in an interventional cardiology setting rather than interventional radiology and therefore did not meet the predefined eligibility criteria for this review. |
| Evsen A, Aktan A, Kılıç R, et al. The predictive value of the CHA2DS2-VASc score in the development of contrast-induced nephropathy after endovascular intervention in peripheral artery disease. Ann Vasc Surg. 2025;116:17–26. https://doi.org/10.1016/j.avsg.2025.03.009 | Wrong procedure specialty/population. This retrospective multicenter study evaluated contrast-induced nephropathy following endovascular interventions for peripheral artery disease, including femoropopliteal and aortoiliac endovascular procedures. The study population primarily represented peripheral vascular intervention and vascular/endovascular practice rather than predefined interventional radiology procedural cohorts according to the eligibility criteria for this review. |
| Faheem MSB, Jahangir N, Malik MH, et al. Managing acute kidney injury: Evaluating amino acid infusion and conventional therapies. Int Urol Nephrol. 2025;57:1035–1036. https://doi.org/10.1007/s11255-024-04219-x | Ineligible study design. This article is a narrative letter to the editor discussing acute kidney injury management strategies and contrast-induced nephropathy prevention concepts. It was not an original clinical study evaluating contrast-associated acute kidney injury following interventional radiology procedures according to the predefined eligibility criteria for this review. |
| Fang Y, Si Y, Yang J, et al. Viabahn Open Revascularization Technique for Renal Artery Revascularization Reduces Renal Ischemia in Thoracoabdominal Aortic Aneurysm Hybrid Open-Endovascular Repair. Ann Vasc Surg. 2019;61:261–269. https://doi.org/10.1016/j.avsg.2019.05.031 | Ineligible outcomes. This retrospective study evaluated renal ischemia time and postoperative acute kidney injury following hybrid open-endovascular repair of thoracoabdominal aortic aneurysms using the Viabahn Open Revascularization Technique. The study focused on ischemic renal injury related to operative renal ischemia rather than contrast-associated acute kidney injury or contrast nephrotoxicity according to the predefined eligibility criteria for this review. |
| Feng Y, Ma XT, Zhang XX, et al. Clinical study of reoperation for acute type A aortic dissection. Front Cardiovasc Med. 2024;11:1340687. https://doi.org/10.3389/fcvm.2024.1340687 | Ineligible outcomes. This retrospective study evaluated outcomes of reoperation for acute type A aortic dissection using endovascular aortic repair and thoracoabdominal aortic replacement techniques. Although postoperative acute kidney injury and renal insufficiency were reported among perioperative complications, the study did not specifically investigate contrast-associated acute kidney injury or contrast nephrotoxicity following interventional radiology procedures according to the predefined eligibility criteria for this review. |
| Ferrario F, Barone MT, Landoni G, et al. Acetylcysteine and non-ionic isosmolar contrast-induced nephropathy—a randomized controlled study. Nephrol Dial Transplant. 2009;24:3103–3107. https://doi.org/10.1093/ndt/gfp306 | Wrong procedure specialty/population. This randomized controlled trial evaluated the prophylactic role of N-acetylcysteine in patients undergoing coronary and/or peripheral angiography and angioplasty procedures. The study population predominantly underwent interventional cardiology procedures rather than interventional radiology procedures according to the predefined eligibility criteria for this review. |
| Filiopoulos V, Biblaki D, Lazarou D, et al. Plasma neutrophil gelatinase-associated lipocalin (NGAL) as an early predictive marker of contrast-induced nephropathy in hospitalized patients undergoing computed tomography. Clin Kidney J. 2013;6:578–583. https://doi.org/10.1093/ckj/sft109 | Wrong procedure type/population. This prospective study evaluated plasma NGAL as an early biomarker of contrast-induced nephropathy in hospitalized patients undergoing intravenous contrast-enhanced computed tomography. The study did not involve interventional radiology or catheter-based endovascular procedures according to the predefined eligibility criteria for this review. |
| Finnesgard EJ, Beck AW, Eagleton MJ, et al. Severity of acute kidney injury is associated with decreased survival after fenestrated and branched endovascular aortic aneurysm repair. J Vasc Surg. 2023;78:892–901. https://doi.org/10.1016/j.jvs.2023.05.034 | Ineligible outcomes. This multicenter cohort study evaluated acute kidney injury severity and survival after fenestrated and branched endovascular aortic aneurysm repair. Although perioperative acute kidney injury was assessed, the study did not specifically investigate contrast-associated acute kidney injury or contrast nephrotoxicity according to the predefined eligibility criteria for this review. |
| Fior D, Di Provvido S, Leni D, et al. Spontaneous Soft Tissue Hematomas in Patients with Coagulation Impairment: Safety and Efficacy of Transarterial Embolization. Tomography. 2023;9:1083–1093. https://doi.org/10.3390/tomography9030089 | Ineligible outcomes. This retrospective interventional radiology study evaluated the safety and efficacy of transarterial embolization for spontaneous soft tissue hematomas in patients with coagulation impairment. Although acute kidney injury occurred as a procedural complication, the study did not specifically investigate contrast-associated acute kidney injury or contrast nephrotoxicity according to the predefined eligibility criteria for this review. |
| Firouzi A, Eshraghi A, Shakerian F, et al. Efficacy of pentoxifylline in prevention of contrast-induced nephropathy in angioplasty patients. Int Urol Nephrol. 2012;44:1145–1149. https://doi.org/10.1007/s11255-011-0053-4 | Wrong procedure specialty/population. This prospective randomized clinical trial evaluated pentoxifylline for prevention of contrast-induced nephropathy in patients undergoing coronary angiography and angioplasty procedures. The procedures were performed in an interventional cardiology setting rather than interventional radiology according to the predefined eligibility criteria for this review. |
| Flashburg E, Ong AW, Muller A, et al. Fall downs should not fall out: Blunt cerebrovascular injury in geriatric patients after low-energy trauma is common. J Trauma Acute Care Surg. 2019;86:1010–1014. https://doi.org/10.1097/TA.0000000000002241 | Wrong procedure type/outcomes. This retrospective trauma study evaluated blunt cerebrovascular injury screening with CT angiography in geriatric patients after low-energy falls. Although acute kidney injury was assessed as a potential imaging-related complication, the study did not specifically investigate contrast-associated acute kidney injury following interventional radiology procedures according to the predefined eligibility criteria for this review. |
| Frank B, Escolà JK, Biermann-Ratjen L, et al. Post-Contrast Acute Kidney Injury after Acute Stroke—Insights from a German Tertiary Care Center. J Clin Med. 2021;10:5684. https://doi.org/10.3390/jcm10235684 | Wrong procedure type/population. This retrospective study evaluated post-contrast acute kidney injury in patients undergoing multimodal acute stroke imaging with CT angiography and CT perfusion, with some patients additionally undergoing digital subtraction angiography/endovascular treatment. The study population was primarily composed of acute stroke imaging patients rather than patients undergoing interventional radiology procedures according to the predefined eligibility criteria for this review. |
| Freitas RAP, Tanajura LFL, Sousa AGMR, et al. Impact of Contrast Volume Used After Percutaneous Coronary Procedures in Patients at Risk for Contrast-Induced Nephropathy. Arq Bras Cardiol. 2025;122:e20250270. https://doi.org/10.36660/abc.20250270 | Wrong procedure specialty/population. This post hoc analysis evaluated the interaction between contrast type and contrast volume in patients undergoing diagnostic coronary angiography and percutaneous coronary intervention procedures. The procedures were performed in an interventional cardiology setting rather than interventional radiology according to the predefined eligibility criteria for this review. |
| Fridling J, Ebadinejad A, Cobar J, et al. Bilateral Versus Unilateral Arterial Embolization in Pelvic Trauma: A ReCONNECT Study. J Surg Res. 2026;321:423–428. https://doi.org/10.1016/j.jss.2026.03.032 | Ineligible outcomes. This multicenter prospective observational study evaluated unilateral versus bilateral pelvic artery embolization strategies in pelvic trauma patients. Although acute kidney injury was reported as a postprocedural complication, the study did not specifically investigate contrast-associated acute kidney injury or contrast nephrotoxicity according to the predefined eligibility criteria for this review. |
| From AM, Bartholmai BJ, Williams AW, et al. Mortality Associated With Nephropathy After Radiographic Contrast Exposure. Mayo Clin Proc. 2008;83:1095–1100. https://doi.org/10.4065/83.10.1095 | Wrong procedure specialty/population. This retrospective cohort study evaluated contrast-induced nephropathy and mortality after both intra-arterial and intravenous radiographic contrast exposure across mixed imaging and coronary angiography populations. The study population was not specific to interventional radiology procedures according to the predefined eligibility criteria for this review. |
| Fujihara M, Kawasaki D, Shintani Y, et al. Endovascular therapy by CO₂ angiography to prevent contrast-induced nephropathy in patients with chronic kidney disease: a prospective multicenter trial of CO₂ angiography registry. Catheter Cardiovasc Interv. 2015;85:870–877. https://doi.org/10.1002/ccd.25722 | Wrong procedure specialty/population. This prospective multicenter registry evaluated contrast-induced nephropathy following CO₂ angiography-guided endovascular therapy for peripheral vascular disease, including renal, aortoiliac, and superficial femoral artery interventions. The study population primarily represented peripheral vascular intervention and vascular/endovascular practice rather than predefined interventional radiology procedural cohorts according to the eligibility criteria for this review. |
| Gaba RC, Lokken RP, Hickey RM, et al. Quality Improvement Guidelines for Transarterial Chemoembolization and Embolization of Hepatic Malignancy. J Vasc Interv Radiol. 2017;28:1210–1223. https://doi.org/10.1016/j.jvir.2017.04.025 | Ineligible study design. This Society of Interventional Radiology quality improvement guideline/review article summarized existing evidence regarding transarterial chemoembolization and embolization for hepatic malignancies, including reported rates of contrast-induced nephropathy/acute kidney injury, but did not present an original patient cohort or primary clinical data eligible for inclusion according to the predefined eligibility criteria for this review. |
| Gallardo X, Almirall J, Castaner E, et al. Assessing the nephrotoxicity of intravenous iodinated contrast agents in patients with chronic renal failure in the absence of interfering acute disease. Acta Radiol. 2019. https://doi.org/10.1177/0284185119840764 | Ineligible procedure type/population. This prospective observational study evaluated nephrotoxicity following intravenous iodinated contrast administration during contrast-enhanced computed tomography (CT) examinations in patients with chronic renal failure undergoing imaging follow-up for aortic aneurysm. The study did not involve interventional radiology procedures according to the predefined eligibility criteria for this review. |
| Gallitto E, Faggioli G, Vacirca A, et al. The benefit of combined carbon dioxide automated angiography and fusion imaging in preserving perioperative renal function in fenestrated endografting. J Vasc Surg. 2020;72:1906–1916. https://doi.org/10.1016/j.jvs.2020.02.051 | Ineligible procedure specialty/population. This observational study evaluated renal outcomes following fenestrated endovascular aneurysm repair (FEVAR) using carbon dioxide angiography and fusion imaging in patients with complex aortic aneurysms. The procedures were vascular surgery/endovascular aortic repair interventions rather than interventional radiology procedures according to the predefined eligibility criteria for this review. |
| Garcia S, Ko B, Adabag S. Contrast-Induced Nephropathy and Risk of Acute Kidney Injury and Mortality After Cardiac Operations. Ann Thorac Surg. 2012;94:772–777. https://doi.org/10.1016/j.athoracsur.2012.04.089 | Wrong procedure specialty/population. This retrospective cohort study evaluated contrast-induced nephropathy after diagnostic coronary angiography and subsequent cardiac surgical procedures, including coronary artery bypass grafting and valve surgery. The study population involved cardiac surgery/coronary angiography patients rather than interventional radiology procedures according to the predefined eligibility criteria for this review. |
| Ghumman SS, Weinerman J, Khan A, et al. Contrast Induced-Acute Kidney Injury following Peripheral Angiography with Carbon Dioxide versus Iodinated Contrast Media: A Meta-Analysis and Systematic Review of Current Literature. Catheter Cardiovasc Interv. 2017;90:437–448. https://doi.org/10.1002/ccd.27051 | Ineligible study design. This meta-analysis and systematic review evaluated acute kidney injury following peripheral angiography using carbon dioxide versus iodinated contrast media and synthesized data from previously published studies. The article did not present an original patient cohort or primary clinical data eligible for inclusion according to the predefined eligibility criteria for this review. |
| Gietzen T, Althoff J, Ochs L, et al. Incidence and clinical impact of renal failure and bleeding following transcatheter tricuspid valve annuloplasty. Clin Res Cardiol. 2025;114:177–186. https://doi.org/10.1007/s00392-024-02388-4 | Wrong procedure specialty/population. This bi-center retrospective study evaluated acute kidney injury and bleeding complications following transcatheter tricuspid valve annuloplasty using the Cardioband system in patients undergoing structural heart/cardiac valve interventions. The study population involved interventional cardiology procedures rather than interventional radiology procedures according to the predefined eligibility criteria for this review. |
| Gill NK, Piccione EA, Vido DA, et al. Gender as a Risk Factor for Contrast Nephropathy: Effects of Hydration and N-Acetylcysteine. Clin Cardiol. 2004;27:554–558. https://doi.org/10.1002/clc.4960271006 | Ineligible procedure specialty/population. This retrospective study evaluated contrast-induced nephropathy prevention strategies in patients with renal insufficiency undergoing cardiac catheterization or peripheral angiography. The study population included mixed cardiac and peripheral angiographic procedures and was not specific to interventional radiology procedures according to the predefined eligibility criteria for this review. |
| Goldfarb S, McCullough PA, McDermott J, et al. Contrast-Induced Acute Kidney Injury: Specialty-Specific Protocols for Interventional Radiology, Diagnostic Computed Tomography Radiology, and Interventional Cardiology. Mayo Clin Proc. 2009;84:170–179. https://doi.org/10.4065/84.2.170 | Ineligible study design. This review article summarized evidence and proposed specialty-specific protocols for prevention of contrast-induced acute kidney injury across interventional radiology, diagnostic radiology, and interventional cardiology settings. The article did not present an original patient cohort or primary clinical data eligible for inclusion according to the predefined eligibility criteria for this review. |
| Grossman PM, Ali SS, Aronow HD, et al. Contrast-induced nephropathy in patients undergoing endovascular peripheral vascular intervention: Incidence, risk factors, and outcomes as observed in the Blue Cross Blue Shield of Michigan Cardiovascular Consortium. J Interven Cardiol. 2017. https://doi.org/10.1111/joic.12379 | Wrong procedure specialty/population. This registry-based observational study evaluated contrast-induced nephropathy following endovascular peripheral vascular interventions performed within an interventional cardiology-led vascular consortium. The study population primarily involved peripheral vascular interventions performed by cardiovascular specialists rather than interventional radiology procedures according to the predefined eligibility criteria for this review. |
| Günebakmaz O, Kaya MG, Koc F, et al. Does Nebivolol Prevent Contrast-Induced Nephropathy in Humans? Clin Cardiol. 2012;35:250–254. https://doi.org/10.1002/clc.21013 | Wrong procedure specialty/population. This prospective randomized study evaluated prophylactic nebivolol use for prevention of contrast-induced nephropathy in patients undergoing coronary angiography and ventriculography. The study population involved cardiac catheterization procedures rather than interventional radiology procedures according to the predefined eligibility criteria for this review. |
| Günebakmaz Ö, Duran M, Karakaya E, et al. Increased serum asymmetric dimethylarginine level is an independent predictor of contrast-induced nephropathy. Turk Kardiyol Dern Ars. 2013;41:581–588. https://doi.org/10.5543/tkda.2013.88403 | Wrong procedure specialty/population. This prospective observational study evaluated serum asymmetric dimethylarginine levels as predictors of contrast-induced nephropathy in patients with stable angina pectoris undergoing coronary angiography and ventriculography. The study population involved cardiac catheterization procedures rather than interventional radiology procedures according to the predefined eligibility criteria for this review. |
| Gupta A, Dosekun AK, Kumar V. Carbon dioxide-angiography for patients with peripheral arterial disease at risk of contrast-induced nephropathy. World J Cardiol. 2020;12:76–90. https://doi.org/10.4330/wjc.v12.i2.76 | Ineligible study design. This review article summarized the use of carbon dioxide angiography in patients with peripheral arterial disease at risk of contrast-induced nephropathy, including technical aspects, safety considerations, and previously published clinical studies. The article did not present an original patient cohort or primary clinical data eligible for inclusion according to the predefined eligibility criteria for this review. |
| Gürses E. Contrast-induced nephropathy after endovascular interventions in peripheral artery disease: Predictive value of the Mehran score. Cardiovasc Surg Int. 2026;13:27–30. https://doi.org/10.4274/e-cvsi.2025.2060 | Wrong procedure specialty/population. This retrospective cohort study evaluated contrast-induced nephropathy following peripheral transcatheter angioplasty in patients with peripheral artery disease within a cardiology/endovascular intervention setting. The study population was not specific to interventional radiology procedures according to the predefined eligibility criteria for this review. |
| Hafiz AM, Jan MF, Mori N, et al. Prevention of Contrast-Induced Acute Kidney Injury in Patients with Stable Chronic Renal Disease Undergoing Elective Percutaneous Coronary and Peripheral Interventions: Randomized Comparison of Two Preventive Strategies. Catheter Cardiovasc Interv. 2012;79:929–937. https://doi.org/10.1002/ccd.23148 | Wrong procedure specialty/population. This randomized controlled trial evaluated preventive strategies for contrast-induced acute kidney injury in patients undergoing elective coronary and peripheral angiography and interventions within a cardiovascular catheterization setting. The study population included mixed coronary and peripheral procedures and was not specific to interventional radiology procedures according to the predefined eligibility criteria for this review. |
| Hall SL, Munich SA, Cress MC, et al. Risk of acute kidney injury associated with neuroimaging obtained during triage and treatment of patients with acute ischemic stroke symptoms. J NeuroInterv Surg. 2016. https://doi.org/10.1136/neurintsurg-2015-012118 | Wrong procedure specialty/population. This retrospective study evaluated acute kidney injury following contrast-enhanced neuroimaging and cerebral angiographic procedures performed during acute ischemic stroke evaluation and treatment. The study population involved neurointerventional stroke imaging/intervention rather than interventional radiology procedures according to the predefined eligibility criteria for this review. |
| Hammami R, Masmoudi O, Jdidi J, et al. Impact of atorvastatin reload on the prevention of contrast-induced nephropathy in patients on chronic statin therapy: A prospective randomized trial. PLoS One. 2023;18:e0270000. https://doi.org/10.1371/journal.pone.0270000 | Wrong procedure specialty/population. This prospective randomized controlled trial evaluated atorvastatin reloading for prevention of contrast-induced nephropathy in patients undergoing coronary angiography or percutaneous coronary intervention within a cardiology catheterization setting. The study population involved coronary procedures rather than interventional radiology procedures according to the predefined eligibility criteria for this review. |
| Han Y, Zhu G, Han L, et al. Short-Term Rosuvastatin Therapy for Prevention of Contrast-Induced Acute Kidney Injury in Patients With Diabetes and Chronic Kidney Disease. J Am Coll Cardiol. 2014;63:62–70. https://doi.org/10.1016/j.jacc.2013.09.017 | Wrong procedure specialty/population. This multicenter randomized controlled trial evaluated rosuvastatin for prevention of contrast-induced acute kidney injury in patients with diabetes mellitus and chronic kidney disease undergoing coronary/peripheral arterial angiography with or without percutaneous intervention within a cardiovascular catheterization setting. The study population included mixed coronary and peripheral procedures and was not specific to interventional radiology procedures according to the predefined eligibility criteria for this review. |
| Hayakawa N, Kodera S, Ohki N, et al. Efficacy and safety of endovascular therapy by diluted contrast digital subtraction angiography in patients with chronic kidney disease. Heart Vessels. 2019;34:1740–1747. https://doi.org/10.1007/s00380-019-01412-2 | Wrong procedure specialty/population. This retrospective study evaluated diluted contrast digital subtraction angiography during endovascular therapy for peripheral artery disease in patients with chronic kidney disease. The study population involved peripheral endovascular interventions within a cardiovascular/endovascular therapy setting rather than interventional radiology procedures according to the predefined eligibility criteria for this review. |
| Hiremath S, Kayibanda JF, Chow BJW, et al. Drug discontinuation before contrast procedures and the effect on acute kidney injury and other clinical outcomes: a systematic review protocol. Syst Rev. 2018;7:34. https://doi.org/10.1186/s13643-018-0701-1 | Ineligible study design. This article was a systematic review protocol evaluating the discontinuation of medications before contrast procedures and their effect on contrast-induced acute kidney injury and other clinical outcomes. The article did not present original patient cohort data or primary clinical outcomes eligible for inclusion according to the predefined eligibility criteria for this review. |
| Ho VT, Nguyen AT, Stern JR, et al. Cost-effectiveness of computed tomography versus ultrasound-based surveillance following endovascular aortic repair of intact infrarenal abdominal aortic aneurysms. J Vasc Surg. 2022;76:707–713. https://doi.org/10.1016/j.jvs.2022.02.057 | Ineligible study design and wrong procedure specialty/population. This cost-effectiveness modeling study evaluated surveillance imaging strategies following endovascular aortic repair for infrarenal abdominal aortic aneurysms and incorporated estimated risks of contrast-induced nephropathy into a decision-analysis framework. The article did not present an original clinical cohort evaluating contrast-associated acute kidney injury after interventional radiology procedures according to the predefined eligibility criteria for this review. |
| Hocine A, Defrance P, Lalmand J, et al. Predictive value of the RIFLE urine output criteria on contrast-induced nephropathy in critically ill patients. BMC Nephrol. 2016;17:36. https://doi.org/10.1186/s12882-016-0243-5 | Wrong procedure specialty/population. This retrospective ICU cohort study evaluated contrast-associated acute kidney injury in critically ill patients undergoing computed tomography or coronary angiography after contrast media administration. The study population involved mixed intensive care imaging and coronary angiography populations rather than interventional radiology procedures according to the predefined eligibility criteria for this review. |
| Holden-Wingate LR, Fitzgibbon JJ, Appah-Sampong A, et al. Safety of pre-operative venogram for hemodialysis access planning in pre-end-stage kidney disease patients. J Vasc Access. 2025;26:1908–1913. https://doi.org/10.1177/11297298241311397 | Wrong procedure specialty/population. This retrospective cohort study evaluated contrast-induced acute kidney injury following pre-operative venography for hemodialysis access planning in patients with chronic kidney disease. The study population involved diagnostic vascular access planning procedures within a nephrology/vascular access setting rather than interventional radiology procedures according to the predefined eligibility criteria for this review. |
| Hosoo H, Tanaka S, Uramaru K, et al. Fifty-percent diluted contrast medium provides acceptable image quality in cerebral angiography. Eur J Radiol. 2026;200:112841. https://doi.org/10.1016/j.ejrad.2026.112841 | Ineligible study design/outcomes. This prospective imaging feasibility study evaluated image quality using 50% diluted contrast medium during cerebral angiography for unruptured aneurysm assessment. Although the study discussed contrast-induced nephropathy risk reduction, it did not primarily evaluate incidence, risk factors, or clinical outcomes of contrast-associated acute kidney injury after interventional radiology procedures according to the predefined eligibility criteria for this review. |
| Hoste EAJ, De Waele JJ, Gevaert SA, et al. Sodium bicarbonate for prevention of contrast-induced acute kidney injury: a systematic review and meta-analysis. Nephrol Dial Transplant. 2010;25:747–758. https://doi.org/10.1093/ndt/gfp389 | Ineligible study design. This systematic review and meta-analysis evaluated sodium bicarbonate for prevention of contrast-induced acute kidney injury across coronary angiography and other contrast-enhanced procedures. The article synthesized previously published studies and did not present an original patient cohort or primary clinical data eligible for inclusion according to the predefined eligibility criteria for this review. |
| Hsieh TM, Tsai TH, Liu YW, et al. Risk factors for contrast-induced nephropathy and their association with mortality in patients with blunt splenic injuries. Int J Surg. 2016;35:69–75. https://doi.org/10.1016/j.ijsu.2016.09.007 | Wrong procedure specialty/population. This retrospective trauma cohort study evaluated contrast-induced nephropathy in patients with blunt splenic injuries undergoing contrast-enhanced computed tomography and selective angiography/angioembolization during trauma management. The study population represented a mixed trauma imaging and intervention cohort rather than patients undergoing interventional radiology procedures according to the predefined eligibility criteria for this review. |
| Hsu TA, Kang SC, Tee YS, et al. The negative effect of preexisting cirrhosis on blunt liver trauma patients: a multifaceted approach from the trauma quality improvement program database. Eur J Trauma Emerg Surg. 2024;50:2209–2217. https://doi.org/10.1007/s00068-024-02655-z | Wrong procedure specialty/population. This retrospective trauma database study evaluated outcomes in blunt liver trauma patients managed non-operatively, including selected patients undergoing transcatheter arterial embolization for hemorrhage control. The study primarily focused on trauma outcomes and pre-existing cirrhosis rather than contrast-associated acute kidney injury following interventional radiology procedures according to the predefined eligibility criteria for this review. |
| Hsu CP, Cheng CT, Huang JF, et al. The effect of transarterial embolization versus nephrectomy on acute kidney injury in blunt renal trauma patients. World J Urol. 2022;40:1859–1865. https://doi.org/10.1007/s00345-022-04049-5 | Wrong procedure specialty/population. This retrospective trauma database study evaluated acute kidney injury in blunt renal trauma patients undergoing transarterial embolization or nephrectomy for hemorrhage control. The study population represented a trauma management cohort rather than patients undergoing interventional radiology procedures according to the predefined eligibility criteria for this review. |
| Hu M, Luo E, Yan G, et al. Microalbuminuria Complicated with Low Estimated Glomerular Filtration Rate: Early Risk Factors for Contrast-Induced Acute Kidney Injury After Coronary Intervention. Med Sci Monit. 2022;28:e935455. https://doi.org/10.12659/MSM.935455 | Wrong procedure specialty/population. This prospective cohort study evaluated contrast-induced acute kidney injury after coronary angiography and percutaneous coronary intervention in patients with coronary artery disease. The study population involved cardiac catheterization procedures rather than interventional radiology procedures according to the predefined eligibility criteria for this review. |
| Huang SS, Huang PH, Wu TC, et al. Association of Serum Bilirubin with Contrast-Induced Nephropathy and Future Cardiovascular Events in Patients Undergoing Coronary Intervention. PLoS One. 2012;7:e42594. https://doi.org/10.1371/journal.pone.0042594 | Wrong procedure specialty/population. This retrospective cohort study evaluated contrast-induced nephropathy and cardiovascular outcomes in patients undergoing coronary intervention procedures. The study population involved coronary angiography and percutaneous coronary intervention within a cardiology catheterization setting rather than interventional radiology procedures according to the predefined eligibility criteria for this review. |
| Huang L, Kan Y, Zhu T, et al. Ten-Year Clinical Characteristics and Early Outcomes of Type B Aortic Dissection Patients With Thoracic Endovascular Aortic Repair. Vasc Endovascular Surg. 2021;55:332–341. https://doi.org/10.1177/1538574420983652 | Wrong study focus/procedure population. This retrospective cohort study evaluated hospitalization characteristics and early outcomes of patients undergoing thoracic endovascular aortic repair for type B aortic dissection. Although acute kidney injury was reported as a postoperative complication, the study did not primarily investigate contrast-associated acute kidney injury incidence, renal risk factors, or contrast-related outcomes following interventional radiology procedures according to the predefined eligibility criteria for this review. |
| Huang JF, Liao CH, Chen SA, et al. The effect of transarterial embolization and nephrectomy on acute kidney injury in patients with blunt renal trauma: An American College of Surgeons - Trauma Quality Improvement Program analysis. Injury. 2024;55:111188. https://doi.org/10.1016/j.injury.2023.111188 | Wrong procedure specialty/population. This retrospective trauma database study evaluated acute kidney injury in patients with blunt renal trauma undergoing transarterial embolization or nephrectomy for hemorrhage control. The study population represented a trauma management cohort rather than patients undergoing interventional radiology procedures according to the predefined eligibility criteria for this review. |
| Hudgi VV, Nishan B, Sivakrishna K, et al. Contrast-Induced Nephropathy Following Peripheral Endovascular Intervention and Its Long-Term Morbidity. Indian J Vasc Endovasc Surg. 2021;8:347–350. https://doi.org/10.4103/ijves.ijves_148_20 | Wrong procedure specialty/population. This prospective observational study evaluated contrast-induced nephropathy following peripheral endovascular revascularization procedures for critical limb ischemia. The study population represented a vascular/endovascular intervention cohort rather than patients undergoing interventional radiology procedures according to the predefined eligibility criteria for this review. |
| Ibrahim TA, El-Mawardy RH, El-Serafy AS, et al. Trimetazidine in the prevention of contrast-induced nephropathy in chronic kidney disease. Cardiovasc Revasc Med. 2017;18:315–319. https://doi.org/10.1016/j.carrev.2017.02.006 | Wrong procedure specialty/population. This prospective study evaluated trimetazidine for prevention of contrast-induced nephropathy in patients with chronic kidney disease undergoing coronary angiography procedures. The study population involved cardiac catheterization procedures rather than interventional radiology procedures according to the predefined eligibility criteria for this review. |
| Ibrahim NE, McCarthy CP, Shrestha S, et al. Blood kidney injury molecule-1 predicts short and longer term kidney outcomes in patients undergoing diagnostic coronary and/or peripheral angiography—Results from the Catheter Sampled Blood Archive in Cardiovascular Diseases (CASABLANCA) study. Am Heart J. 2019;209:36–46. https://doi.org/10.1016/j.ahj.2018.12.001 | Wrong procedure specialty/population. This prospective cohort study evaluated kidney injury molecule-1 as a biomarker for acute kidney injury and chronic kidney disease progression in patients undergoing coronary and/or peripheral angiography procedures. The study population involved cardiovascular catheterization and peripheral angiography cohorts rather than interventional radiology procedures according to the predefined eligibility criteria for this review. |
| Ilis D, Arslan A, Artac I, et al. The predictive value of AGEF score for contrast-induced nephropathy in patients with lower extremity peripheral artery disease. BMC Cardiovasc Disord. 2025;25:781. https://doi.org/10.1186/s12872-025-05240-3 | Wrong procedure specialty/population. This retrospective cohort study evaluated contrast-induced nephropathy following endovascular interventions for lower extremity peripheral artery disease. The study population represented a vascular/endovascular intervention cohort rather than patients undergoing interventional radiology procedures according to the predefined eligibility criteria for this review. |
| Jain V, Mehta Y, Gupta A, et al. The role of neutrophil gelatinase-associated lipocalin in predicting acute kidney injury in patients undergoing off-pump coronary artery bypass graft: A pilot study. Ann Card Anaesth. 2016;19:225–230. https://doi.org/10.4103/0971-9784.179590 | Wrong procedure specialty/population. This prospective cohort study evaluated neutrophil gelatinase-associated lipocalin as a biomarker for acute kidney injury in patients undergoing off-pump coronary artery bypass graft surgery. The study population involved cardiac surgical patients rather than patients undergoing interventional radiology procedures according to the predefined eligibility criteria for this review. |
| Jain S, Nimkar A, Hong S, et al. Cholesterol Embolic Renal Disease: Experience of a Large Healthcare System. Int J Surg Pathol. 2025;33:330–336. https://doi.org/10.1177/10668969241261542 | Ineligible study focus/outcome. This retrospective pathology case series evaluated cholesterol embolic renal disease identified on kidney biopsy specimens in patients with systemic atheroembolic disease, including some with prior intravascular interventional procedures. The study focused on histopathologic features and cholesterol embolic renal disease rather than contrast-associated acute kidney injury following interventional radiology procedures according to the predefined eligibility criteria for this review. |
| Jakobi T, Meyborg M, Freisinger E, et al. Feasibility and impact of carbon dioxide angiography on acute kidney injury following endovascular interventions in patients with peripheral artery disease and renal impairment. J Nephrol. 2021. https://doi.org/10.1007/s40620-020-00909-8 | Wrong procedure specialty/population. This prospective study evaluated post-contrast acute kidney injury following endovascular revascularization procedures in patients with peripheral artery disease and chronic kidney disease using carbon dioxide angiography. The study population represented a vascular/endovascular intervention cohort rather than patients undergoing interventional radiology procedures according to the predefined eligibility criteria for this review. |
| James MT, Har BJ, Tyrrell BD, et al. Effect of Clinical Decision Support With Audit and Feedback on Prevention of Acute Kidney Injury in Patients Undergoing Coronary Angiography: A Randomized Clinical Trial. JAMA. 2022;328:839–849. https://doi.org/10.1001/jama.2022.13382 | Wrong procedure specialty/population. This stepped-wedge cluster randomized clinical trial evaluated clinical decision support strategies for prevention of acute kidney injury in patients undergoing coronary angiography and percutaneous coronary intervention. The study population involved cardiac catheterization procedures rather than interventional radiology procedures according to the predefined eligibility criteria for this review. |
| James MT, Samuel SM, Manning MA, et al. Contrast-Induced Acute Kidney Injury and Risk of Adverse Clinical Outcomes After Coronary Angiography: A Systematic Review and Meta-Analysis. Circ Cardiovasc Interv. 2013;6:37–43. https://doi.org/10.1161/CIRCINTERVENTIONS.112.974493 | Ineligible study design and wrong procedure specialty/population. This systematic review and meta-analysis evaluated contrast-induced acute kidney injury and clinical outcomes following coronary angiography and percutaneous coronary intervention across cardiology catheterization cohorts. The article synthesized previously published studies and did not present original interventional radiology patient cohort data according to the predefined eligibility criteria for this review. |
| Janhofer DE, Lakhiani C, Kim PJ, et al. The Utility of Preoperative Arteriography for Free Flap Planning in Patients with Chronic Lower Extremity Wounds. Plast Reconstr Surg. 2019;143:604–613. https://doi.org/10.1097/PRS.0000000000005265 | Wrong study focus/outcome. This retrospective reconstructive surgery cohort study evaluated the utility of preoperative lower extremity arteriography for recipient vessel selection and free flap planning in patients with chronic lower extremity wounds. Although contrast-induced acute kidney injury after arteriography was reported as a procedural complication, the study did not primarily investigate incidence, risk factors, or outcomes of contrast-associated acute kidney injury following interventional radiology procedures according to the predefined eligibility criteria for this review. |
| Janssens GN, Daemen J, Lemkes JS, et al. The influence of timing of coronary angiography on acute kidney injury in out-of-hospital cardiac arrest patients: a retrospective cohort study. Ann Intensive Care. 2022;12:12. https://doi.org/10.1186/s13613-022-00987-w | Wrong procedure specialty/population. This retrospective multicenter cohort study evaluated acute kidney injury after coronary angiography in out-of-hospital cardiac arrest survivors within a cardiology catheterization setting. The study population involved coronary angiography and percutaneous coronary intervention procedures rather than interventional radiology procedures according to the predefined eligibility criteria for this review. |
| Jhaveri KD, Saratzis AN, Wanchoo R, et al. Endovascular aneurysm repair (EVAR)– and transcatheter aortic valve replacement (TAVR)–associated acute kidney injury. Kidney Int. 2017;91:1312–1323. https://doi.org/10.1016/j.kint.2016.11.030 | Ineligible study design and mixed procedure specialty/population. This narrative review summarized incidence, risk factors, mechanisms, and outcomes of acute kidney injury after endovascular aneurysm repair and transcatheter aortic valve replacement across vascular surgery and cardiology populations. The article did not present original interventional radiology patient cohort data according to the predefined eligibility criteria for this review. |
| Jhou HJ, Chen PH, Yang LY, et al. Contrast-Associated Acute Kidney Injury After Endovascular Therapy for Acute Ischemic Stroke: A Meta-Analysis. Stroke Vasc Interv Neurol. 2022;2:e000296. https://doi.org/10.1161/SVIN.121.000296 | Ineligible study design. This meta-analysis evaluated contrast-associated acute kidney injury after neuroendovascular therapy for acute ischemic stroke across multiple observational cohorts. Although the study investigated image-guided endovascular procedures relevant to neurointerventional radiology, it synthesized previously published studies and did not provide an original patient cohort eligible for inclusion according to the predefined eligibility criteria for this review. |
| Jiang W, Xie Q, Hu J, et al. Effect of angiography timing on acute kidney injury after cardiac surgery in patients with preoperative renal dysfunction. BMC Nephrol. 2023;24:94. https://doi.org/10.1186/s12882-023-03144-y | Wrong procedure specialty/population. This retrospective cohort study evaluated acute kidney injury associated with preoperative coronary angiography timing in patients undergoing cardiac surgery. The study population involved cardiology and cardiac surgical procedures rather than interventional radiology procedures according to the predefined eligibility criteria for this review. |
| Joshi D, Madan T, Charaniya R, et al. Study of endovascular treatment in obstructive aortoiliac lesions: Immediate and short-term results. Heart Vessels Transp. 2022;6. https://doi.org/10.24969/hvt.2021.298 | Wrong study focus/procedure population. This prospective observational study evaluated technical and short-term clinical outcomes following endovascular treatment for aortoiliac occlusive disease. Although contrast-induced nephropathy was reported as a procedural complication, the study primarily focused on peripheral vascular intervention outcomes and stent patency rather than contrast-associated acute kidney injury following interventional radiology procedures according to the predefined eligibility criteria for this review. |
| Kabeer MA, Cross J, Hamilton G, et al. Obesity as a Risk Factor for Radiographic Contrast-Induced Nephropathy. Angiology. 2021;72:274–278. https://doi.org/10.1177/0003319720969536 | Wrong procedure specialty/population. This prospective study evaluated contrast-induced nephropathy in patients with peripheral arterial disease undergoing angiography and peripheral vascular intervention procedures. The study population represented a peripheral vascular angiography/endovascular cohort rather than patients undergoing interventional radiology procedures according to the predefined eligibility criteria for this review. |
| Kama A, Yılmaz S, Yaka E, et al. Comparison of Short-term Infusion Regimens of N-Acetylcysteine Plus Intravenous Fluids, Sodium Bicarbonate Plus Intravenous Fluids, and Intravenous Fluids Alone for Prevention of Contrast-induced Nephropathy in the Emergency Department. Acad Emerg Med. 2014;21:616–622. https://doi.org/10.1111/acem.12400 | Wrong study focus/procedure population. This randomized clinical trial evaluated pharmacologic prophylaxis strategies for prevention of contrast-induced nephropathy in emergency department patients undergoing contrast-enhanced computed tomography. The study population involved diagnostic intravenous contrast-enhanced imaging rather than patients undergoing interventional radiology procedures according to the predefined eligibility criteria for this review. |
| Kanai D, Fujii H, Nakai K, et al. Statin Use Influence on the Occurrence of Acute Kidney Injury in Patients with Peripheral Arterial Disease. J Atheroscler Thromb. 2022;29:1646–1654. http://doi.org/10.5551/jat.63265 | Wrong procedure specialty/population. This retrospective observational study evaluated acute kidney injury following angiography and/or endovascular intervention in patients with peripheral arterial disease. The study population represented a peripheral vascular/endovascular intervention cohort rather than patients undergoing interventional radiology procedures according to the predefined eligibility criteria for this review. |
| Kang C, Han SH, Park JS, et al. Risk factors for post-contrast acute kidney injury in patients sequentially administered iodine- and gadolinium-based contrast media on the same visit to the emergency department: a retrospective study. Kidney Res Clin Pract. 2023;42:358–369. https://doi.org/10.23876/j.krcp.22.026 | Wrong study focus/procedure population. This retrospective emergency department cohort study evaluated post-contrast acute kidney injury following sequential administration of iodine- and gadolinium-based contrast media during enhanced computed tomography and magnetic resonance imaging examinations. The study population involved diagnostic contrast-enhanced imaging rather than patients undergoing interventional radiology procedures according to the predefined eligibility criteria for this review. |
| Karaduman A, Yılmaz C, Keten MF, et al. Prognostic value of nutrition for contrast-induced nephropathy in patients undergoing peripheral vascular intervention. Biomark Med. 2024;18:801–811. https://doi.org/10.1080/17520363.2024.2395248 | Wrong procedure specialty/population. This retrospective observational study evaluated contrast-associated acute kidney injury following peripheral vascular intervention in patients with peripheral arterial disease. The study population represented a peripheral vascular/endovascular intervention cohort rather than patients undergoing interventional radiology procedures according to the predefined eligibility criteria for this review. |
| Karlsberg RP, Dohad SY, Sheng R. Contrast Medium–Induced Acute Kidney Injury: Comparison of Intravenous and Intraarterial Administration of Iodinated Contrast Medium. J Vasc Interv Radiol. 2011;22:1159–1165. https://doi.org/10.1016/j.jvir.2011.03.020 | Wrong procedure specialty/population. This multicenter study evaluated contrast medium–induced acute kidney injury following intravenous computed tomographic angiography and intraarterial digital subtraction angiography in patients with suspected peripheral arterial occlusive disease. The study population represented a peripheral vascular angiographic/endovascular cohort rather than patients undergoing interventional radiology procedures according to the predefined eligibility criteria for this review. |
| Karlsberg RP, Dohad SY, Sheng R. Contrast-induced acute kidney injury (CI-AKI) following intra-arterial administration of iodinated contrast media. J Nephrol. 2010;23:658–666. http://doi.org/10.1093/joneph/23.6.658 | Wrong procedure specialty/population. This multicenter study evaluated contrast-induced acute kidney injury following intraarterial digital subtraction angiography in patients with suspected peripheral arterial occlusive disease. The study population represented a peripheral vascular angiographic/endovascular cohort rather than patients undergoing interventional radiology procedures according to the predefined eligibility criteria for this review. |
| Katsogridakis E, Saha P, Diamantopoulos A, et al. Long-Term Effects of Acute Kidney Injury Following Endovascular Femoropopliteal Intervention: Insights From a Multicenter Trial. J Endovasc Ther. 2024;31:634–642. https://doi.org/10.1177/15266028221136436 | Wrong procedure specialty/population. This multicenter study evaluated acute kidney injury and long-term cardiovascular outcomes following femoropopliteal endovascular intervention in patients with peripheral arterial disease. The study population represented a peripheral vascular/endovascular intervention cohort rather than patients undergoing interventional radiology procedures according to the predefined eligibility criteria for this review. |
| Katsogridakis E, Lea T, Yap T, et al. Acute kidney injury following endovascular intervention for peripheral artery disease. Br J Surg. 2021;108:152–159. https://doi.org/10.1093/bjs/znaa057 | Wrong procedure specialty/population. This multicenter study evaluated acute kidney injury following femoropopliteal endovascular intervention in patients with symptomatic peripheral arterial disease. The study population represented a peripheral vascular/endovascular intervention cohort rather than patients undergoing interventional radiology procedures according to the predefined eligibility criteria for this review. |
| Kawatani Y, Kurobe H, Nakamura Y, et al. The ratio of contrast medium volume to estimated glomerular filtration rate as a predictor of contrast-induced nephropathy after endovascular aortic repair. J Med Invest. 2018;65:116–121. http://doi.org/10.2152/jmi.65.116 | Wrong procedure specialty/population. This retrospective study evaluated contrast-induced nephropathy following endovascular aortic repair in patients with thoracic and abdominal aortic aneurysms. The study population represented an aortic endovascular surgery cohort rather than patients undergoing interventional radiology procedures according to the predefined eligibility criteria for this review. |
| Kelemen JA, Kaserer A, Jensen KO, et al. Prevalence and outcome of contrast-induced nephropathy in major trauma patients. Eur J Trauma Emerg Surg. 2022;48:907–913. https://doi.org/10.1007/s00068-020-01496-w | Wrong study focus/procedure population. This retrospective trauma cohort study evaluated contrast-induced nephropathy following contrast-enhanced whole-body computed tomography in severely injured trauma patients. The study population involved diagnostic contrast-enhanced CT imaging rather than patients undergoing interventional radiology procedures according to the predefined eligibility criteria for this review. |
| Khosravi A, Eghbal A, Kermani-Alghoraishi M, et al. Short and one-year outcome of endovascular aortic repair (EVAR) for abdominal aortic aneurysms (AAAs). ARYA Atheroscler. 2022;18:2518. http://dx.doi.org/10.48305/arya.2022.16298.2518 | Wrong study focus/procedure population. This retrospective cohort study evaluated short- and long-term outcomes following endovascular aortic repair for abdominal aortic aneurysms. Although contrast-induced nephropathy was reported as a postoperative complication, the study primarily focused on EVAR procedural outcomes and mortality in an aortic endovascular surgery cohort rather than contrast-associated acute kidney injury following interventional radiology procedures according to the predefined eligibility criteria for this review. |
| Ki YJ, Kwon SA, Kim HL, et al. The Prevention of Contrast Induced Nephropathy by Sarpogrelate: a Prospective Randomized Controlled Clinical Trial. J Korean Med Sci. 2019;34:e261. https://doi.org/10.3346/jkms.2019.34.e261 | Wrong procedure specialty/population. This prospective randomized controlled trial evaluated prophylactic sarpogrelate therapy for prevention of contrast-induced nephropathy in patients with chronic kidney disease undergoing coronary angiography and percutaneous coronary intervention. The study population involved cardiology catheterization procedures rather than interventional radiology procedures according to the predefined eligibility criteria for this review. |
| Kim GH, Kim J, Ko E, et al. Effectiveness and Safety of Transarterial Radioembolization Following Inferior Phrenic Artery Glue Embolization for Flow Redistribution in Hepatocellular Carcinoma. J Vasc Interv Radiol. 2026;37:108784. http://doi.org/10.1016/j.jvir.2026.108784 | Wrong outcome of interest. This retrospective cohort study evaluated tumor response, survival outcomes, and procedural safety following transarterial radioembolization with inferior phrenic artery glue embolization in hepatocellular carcinoma. Although acute kidney injury was reported as a rare adverse event, renal injury or contrast-associated acute kidney injury was not a predefined primary renal outcome or central study objective according to the predefined eligibility criteria for this review. |
| Kim MY, Jang HR, Huh W, et al. Incidence, Risk Factors, and Prediction of Acute Kidney Injury After Off-Pump Coronary Artery Bypass Grafting. Ren Fail. 2011;33:316–322. https://doi.org/10.3109/0886022X.2011.560406 | Wrong procedure specialty/population. This retrospective cohort study evaluated acute kidney injury following off-pump coronary artery bypass graft surgery in cardiac surgical patients. Although timing of coronary angiography before surgery was analyzed as a potential contributor to postoperative renal injury, the study population involved cardiac surgery rather than patients undergoing interventional radiology procedures according to the predefined eligibility criteria for this review. |
| Kim LE, Park JH, Lee HC, et al. Midterm Clinical Outcomes of Endovascular Treatment for Acute Aortic Dissection with Malperfusion Syndrome. Vasc Specialist Int. 2024;40:17. https://doi.org/10.5758/vsi.240006 | Wrong study focus/procedure population. This retrospective study evaluated midterm clinical outcomes following thoracic endovascular aortic repair and selective branch-vessel stenting for acute type B aortic dissection with malperfusion syndrome. Although acute kidney injury and renal replacement therapy were reported as procedural complications, the study primarily focused on aortic endovascular treatment outcomes rather than contrast-associated acute kidney injury following interventional radiology procedures according to the predefined eligibility criteria for this review. |
| Kim M, Brady JE, Li G. Anesthetic Technique and Acute Kidney Injury in Endovascular Abdominal Aortic Aneurysm Repair. J Cardiothorac Vasc Anesth. 2014;28:572–578. https://doi.org/10.1053/j.jvca.2013.06.001 | Wrong procedure specialty/population. This retrospective cohort study evaluated the association between anesthetic technique and acute kidney injury following endovascular abdominal aortic aneurysm repair in vascular surgery patients. Although perioperative acute kidney injury and contrast nephropathy were discussed as postoperative complications, the study primarily focused on anesthetic management and perioperative outcomes in an aortic endovascular surgery cohort rather than contrast-associated acute kidney injury following interventional radiology procedures according to the predefined eligibility criteria for this review. |
| Kim YT, Bak SH, Han SS, et al. Non-contrast CT-based pulmonary embolism detection using GAN-generated synthetic contrast enhancement: Development and validation of an AI framework. Comput Biol Med. 2025;198:111109. https://doi.org/10.1016/j.compbiomed.2025.111109 | Wrong study focus/procedure population and ineligible study design. This artificial intelligence imaging study developed a generative adversarial network framework for synthetic contrast-enhanced chest CT imaging and pulmonary embolism detection using non-contrast CT scans. Although the article discussed contrast-induced nephropathy as a limitation of CT pulmonary angiography, the study did not evaluate original patient cohorts undergoing interventional radiology procedures or investigate contrast-associated acute kidney injury outcomes according to the predefined eligibility criteria for this review. |
| Kim GS, Ko YG, Shin DH, et al. Elevated serum cystatin C level is an independent predictor of contrast-induced nephropathy and adverse outcomes in patients with peripheral artery disease undergoing endovascular therapy. J Vasc Surg. 2015;61:1223–1230. https://doi.org/10.1016/j.jvs.2014.11.079 | Wrong procedure specialty/population. This retrospective study evaluated contrast-induced nephropathy and long-term outcomes in patients with peripheral artery disease undergoing lower extremity endovascular therapy. The study population represented a peripheral vascular/endovascular intervention cohort rather than patients undergoing interventional radiology procedures according to the predefined eligibility criteria for this review. |
| Klima T, Christ A, Marana I, et al. Sodium chloride vs. sodium bicarbonate for the prevention of contrast medium-induced nephropathy: a randomized controlled trial. Eur Heart J. 2012;33:2071–2079. https://doi.org/10.1093/eurheartj/ehr501 | Wrong procedure specialty/population. This randomized controlled trial evaluated preventive hydration regimens for contrast-induced nephropathy in patients undergoing mixed intravascular contrast procedures, predominantly cardiac catheterization and percutaneous coronary intervention. The study population primarily involved cardiology catheterization procedures rather than interventional radiology procedures according to the predefined eligibility criteria for this review. |
| Kooiman J, de Vries JPPM, Van der Heyden J, et al. Randomized trial of one-hour sodium bicarbonate vs standard periprocedural saline hydration in chronic kidney disease patients undergoing cardiovascular contrast procedures. PLoS One. 2018;13:e0189372. https://doi.org/10.1371/journal.pone.0189372 | Wrong procedure specialty/population. This multicenter randomized trial evaluated preventive hydration regimens for contrast-induced acute kidney injury in patients with chronic kidney disease undergoing mixed cardiovascular contrast procedures, predominantly coronary angiography, percutaneous coronary intervention, and peripheral vascular interventions. The study population primarily involved cardiology and vascular intervention procedures rather than interventional radiology procedures according to the predefined eligibility criteria for this review. |
| Korczak A, Morawiec R, Stegienta M, et al. Acute kidney injury as the most important predictor of poor prognosis after interventional treatment for aortic stenosis. Kardiol Pol. 2022;80:1032–1038. https://doi.org/10.33963/KP.a2022.0182 | Wrong procedure specialty/population. This retrospective study evaluated acute kidney injury and long-term cardiovascular outcomes following surgical aortic valve replacement and transcatheter aortic valve implantation in patients with severe aortic stenosis. The study population involved cardiac surgery and structural heart interventions rather than interventional radiology procedures according to the predefined eligibility criteria for this review. |
| Kord A, Park SJ, Rao A, et al. Postcontrast Acute Kidney Injury After Transarterial Chemoembolization. Semin Intervent Radiol. 2021;38:139–143. https://doi.org/10.1055/s-0041-1724016 | Ineligible study design. This article is a case-based educational review discussing postcontrast acute kidney injury following transarterial chemoembolization, including a single illustrative patient case and a narrative review of pathophysiology, prevention, and management strategies. The study did not include an original cohort analysis or systematic evaluation of contrast-associated acute kidney injury outcomes in patients undergoing interventional radiology procedures according to the predefined eligibility criteria for this review. |
| Kotlyar E, Keogh AM, Thavapalachandran S, et al. Prehydration Alone is Sufficient to Prevent Contrast-induced Nephropathy after Day-only Angiography Procedures—A Randomised Controlled Trial. Heart Lung Circ. 2005;14:245–251. https://doi.org/10.1016/j.hlc.2005.06.007 | Wrong procedure specialty/population. This randomized controlled trial evaluated preventive hydration and intravenous N-acetylcysteine protocols in patients undergoing coronary and peripheral angiography and/or stenting procedures. The study population primarily involved cardiology catheterization and vascular intervention procedures rather than interventional radiology procedures according to the predefined eligibility criteria for this review. |
| Kuźma Ł, Tomaszuk-Kazberuk A, Kurasz A, et al. Atrial Fibrillation and Chronic Kidney Disease—A Risky Combination for Post-Contrast Acute Kidney Injury. J Clin Med. 2021;10:4140. https://doi.org/10.3390/jcm10184140 | Wrong procedure specialty/population. This retrospective observational study evaluated post-contrast acute kidney injury in patients with atrial fibrillation and chronic kidney disease undergoing elective coronary angiography. The study population primarily involved cardiology coronary angiography procedures rather than patients undergoing interventional radiology procedures according to the predefined eligibility criteria for this review. |
| La Manna G, Pancaldi LG, Capecchi A, et al. Risk for Contrast Nephropathy in Patients Undergoing Coronarography. Artif Organs. 2010;34:E193–E199. https://doi.org/10.1111/j.1525-1594.2009.00984.x . | Wrong procedure specialty/population. This observational study evaluated risk factors and prevention strategies for contrast-induced nephropathy in patients undergoing coronarography/coronary angiographic procedures in a cardiology intensive care setting. The study population involved interventional cardiology/coronary procedures rather than interventional radiology procedures according to the predefined eligibility criteria for this review. |
| Lee HN, Yang SB, Goo DE, et al. Impact of Superselective Renal Artery Embolization on Renal Function and Blood Pressure. J Belg Soc Radiol. 2020;104:59. https://doi.org/10.5334/jbsr.2223 | Renal-directed embolization population. This retrospective study evaluated acute kidney injury and renal function changes following therapeutic renal artery embolization procedures. Because renal ischemia and intentional renal parenchymal devascularization were intrinsic components of the intervention, the mechanisms of postprocedural renal injury were not directly comparable to predefined contrast-associated acute kidney injury populations included in this review. |
| Lee SR, Zhuo H, Zhang Y, et al. Risk factors and safe contrast volume thresholds for postcontrast acute kidney injury after peripheral vascular interventions. J Vasc Surg. 2020;72:603–610. https://doi.org/10.1016/j.jvs.2019.09.059 | Wrong procedure specialty/population. This large registry-based study evaluated postcontrast acute kidney injury following peripheral vascular interventions and endovascular revascularization procedures in patients with peripheral arterial disease. The study population represented a peripheral vascular/endovascular intervention cohort rather than patients undergoing interventional radiology procedures according to the predefined eligibility criteria for this review. |
| Lee SR, Dardik A, Ochoa Chaar CI. Postcontrast Acute Kidney Injury after Peripheral Vascular Interventions in Kidney Transplant Recipients. Ann Vasc Surg. 2020;68:8–14. https://doi.org/10.1016/j.avsg.2020.04.057 | Wrong procedure specialty/population. This registry-based study evaluated postcontrast acute kidney injury following peripheral vascular interventions in kidney transplant recipients with peripheral arterial disease. The study population represented a peripheral vascular/endovascular intervention cohort rather than patients undergoing interventional radiology procedures according to the predefined eligibility criteria for this review. |
| Lee JH, Chung B, Lee SC, et al. Lower incidence of contrast-induced nephropathy in patients undergoing fluorescent angiography. BMC Ophthalmol. 2017;17:46. https://doi.org/10.1186/s12886-017-0440-4 | Wrong procedure type/population. This retrospective study evaluated contrast-induced nephropathy following fluorescein angiography performed as part of ophthalmologic diagnostic imaging examinations. The study population underwent diagnostic ophthalmic fluorescent angiography rather than interventional radiology procedures according to the predefined eligibility criteria for this review. |
| Lee HC, Joo HC, Lee SH, et al. Endovascular Repair versus Open Repair for Isolated Descending Thoracic Aortic Aneurysm. Yonsei Med J. 2015;56:904–912. https://doi.org/10.3349/ymj.2015.56.4.904 | Wrong study focus/procedure population. This retrospective comparative study evaluated thoracic endovascular aortic repair versus open surgical repair for descending thoracic aortic aneurysms. Although postoperative acute kidney injury and dialysis were reported as perioperative complications, the study primarily focused on overall surgical and endovascular aortic repair outcomes rather than contrast-associated acute kidney injury following interventional radiology procedures according to the predefined eligibility criteria for this review. |
| Lee SR, Ali S, Cardella J, et al. Carbon dioxide angiography during peripheral vascular interventions is associated with decreased cardiac and renal complications in patients with chronic kidney disease. J Vasc Surg. 2023;78:201–208. https://doi.org/10.1016/j.jvs.2023.03.029 | Wrong procedure specialty/population. This registry-based study evaluated post-contrast acute kidney injury and carbon dioxide angiography during peripheral vascular interventions in patients with chronic kidney disease undergoing endovascular treatment for peripheral arterial disease. The study population represented a peripheral vascular/endovascular intervention cohort rather than patients undergoing interventional radiology procedures according to the predefined eligibility criteria for this review. |
| Lee T, Kim WK, Kim AJ, et al. Low-Osmolar vs. Iso-Osmolar Contrast Media on the Risk of Contrast-Induced Acute Kidney Injury: A Propensity Score Matched Study. Front Med (Lausanne). 2022;9:862023. https://doi.org/10.3389/fmed.2022.862023 | Wrong procedure specialty/population. This retrospective propensity score-matched study evaluated contrast-induced acute kidney injury after coronary angiography and percutaneous coronary intervention using different contrast media types. The study population primarily involved cardiology catheterization procedures rather than patients undergoing interventional radiology procedures according to the predefined eligibility criteria for this review. |
| Lee KB, Porras-Colon J, Scott CK, et al. Early Results and Feasibility of Total Endovascular Aortic Arch Repair Using 3-Vessel Company-Manufactured and Physician-Modified Stent-Grafts. J Endovasc Ther. 2024;31:1197–1207. https://doi.org/10.1177/15266028231163069 | Wrong study focus/procedure population. This retrospective case series evaluated total endovascular aortic arch repair using company-manufactured and physician-modified stent-grafts for complex aortic arch disease. Although acute kidney injury and dialysis were reported as perioperative complications, the study primarily focused on technical feasibility and outcomes of complex aortic endovascular surgery rather than contrast-associated acute kidney injury following interventional radiology procedures according to the predefined eligibility criteria for this review. |
| Lehti L, Nyman U, Söderberg M, et al. 80-kVp CT angiography for endovascular aneurysm repair follow-up with halved contrast medium dose and preserved diagnostic quality. Acta Radiol. 2016;57:279–286. https://doi.org/10.1177/0284185115577251 | Wrong study focus/procedure population. This retrospective imaging study evaluated low-dose computed tomography angiography protocols for surveillance after endovascular aneurysm repair in patients with impaired renal function. Although contrast-induced nephropathy risk reduction was discussed, the study focused on diagnostic CT angiography imaging techniques and image quality rather than original clinical outcomes of contrast-associated acute kidney injury following interventional radiology procedures according to the predefined eligibility criteria for this review. |
| Lei L, Xue Y, Guo Z, et al. A comparison between different definitions of contrast-induced acute kidney injury for long-term mortality in patients with acute myocardial infarction. IJC Heart Vasc. 2020;28:100522. https://doi.org/10.1016/j.ijcha.2020.100522 | Wrong procedure specialty/population. This observational cohort study evaluated different definitions of contrast-induced acute kidney injury and long-term mortality in patients with acute myocardial infarction undergoing coronary angiography and percutaneous coronary intervention. The study population primarily involved cardiology catheterization procedures rather than patients undergoing interventional radiology procedures according to the predefined eligibility criteria for this review. |
| Lembo C, Méot M, Mellul K, et al. Renal function after ductus arteriosus transcatheter closure with or without angiography in very preterm infants. Acta Paediatr. 2024;113:955–961. https://doi.org/10.1111/apa.17101 | Wrong procedure specialty/population. This retrospective comparative study evaluated renal function after transcatheter patent ductus arteriosus closure with or without angiography in very preterm infants undergoing congenital cardiac catheter-based intervention. Although contrast-induced nephropathy and acute kidney injury were assessed as outcomes, the study population involved paediatric congenital cardiology procedures rather than interventional radiology procedures according to the predefined eligibility criteria for this review. |
| Leoncini M, Toso A, Maioli M, et al. Early High-Dose Rosuvastatin for Contrast-Induced Nephropathy Prevention in Acute Coronary Syndrome: Results From the PRATO-ACS Study. J Am Coll Cardiol. 2014;63:71–79. https://doi.org/10.1016/j.jacc.2013.04.105 | Wrong procedure specialty/population. This randomized trial evaluated prevention of contrast-induced acute kidney injury in statin-naïve patients with acute coronary syndrome undergoing coronary angiography and percutaneous coronary intervention. The study population primarily involved cardiology catheterization procedures rather than patients undergoing interventional radiology procedures according to the predefined eligibility criteria for this review. |
| Li L, Shao J, Niu W, et al. Neutrophil Gelatinase-Associated Lipocalin as an Early Predictor of Contrast-Induced Nephropathy Following Endovascular Aortic Repair for Abdominal Aortic Aneurysm. Clin Appl Thromb Hemost. 2021;27:10760296211025618. https://doi.org/10.1177/10760296211025618 | Wrong procedure specialty/population. This prospective cohort study evaluated contrast-induced nephropathy following endovascular aortic repair (EVAR) for abdominal aortic aneurysm and focused on biomarker prediction of acute kidney injury. The study population primarily represented vascular surgery/endovascular aneurysm repair practice rather than predefined interventional radiology procedural cohorts according to the eligibility criteria for this review. |
| Li N, Zhang Z, Wang Y, et al. Risk factors for acute kidney injury and its impact on 3-year mortality after thoracic endovascular repair for type B aortic dissection. Front Cardiovasc Med. 2025;12:1691995. https://doi.org/10.3389/fcvm.2025.1691995 | Wrong procedure specialty/population. This retrospective cohort study evaluated acute kidney injury following thoracic endovascular aortic repair (TEVAR) for acute Stanford type B aortic dissection. The study population primarily represented cardiovascular surgery/endovascular aortic repair practice rather than predefined interventional radiology procedural cohorts according to the eligibility criteria for this review. |
| Li X, Zhang W, Liu J, et al. Contrast-Induced Kidney Nephropathy in Thoracic Endovascular Aortic Repair: A 2-Year Retrospective Study in 470 Patients. Angiology. 2020;71:242–248. https://doi.org/10.1177/0003319719893578 | Wrong procedure specialty/population. This retrospective cohort study evaluated contrast-induced nephropathy following thoracic endovascular aortic repair (TEVAR) in patients with Stanford type B aortic dissection. The study population primarily represented vascular surgery/endovascular aortic repair practice rather than predefined interventional radiology procedural cohorts according to the eligibility criteria for this review. |
| Li S, Wang Q, Mei J, et al. Risk factors of extra-hepatic progression after transarterial chemoembolization for hepatocellular carcinoma patients: a retrospective study in 654 cases. J Cancer. 2019;10:5007–5014. https://doi.org/10.7150/jca.35355 | Wrong study focus/outcomes. This retrospective study evaluated risk factors for extrahepatic progression and survival outcomes after transarterial chemoembolization in patients with hepatocellular carcinoma. Although TACE is an interventional radiology procedure, the study did not investigate contrast-associated acute kidney injury, contrast-induced nephropathy, renal outcomes, or kidney-related complications according to the predefined eligibility criteria for this review. |
| Li J, Peng Y, Zhang X, et al. Endovascular aneurysm repair for aorto-iliac artery pathologies in patients with autosomal dominant polycystic kidney disease. Int Angiol. 2022;41:41–47. https://doi.org/10.23736/S0392-9590.21.04692-7 | Wrong study focus/procedure population. This retrospective case series evaluated the feasibility and safety of endovascular aneurysm repair and thoracic endovascular aortic repair in patients with autosomal dominant polycystic kidney disease. Although contrast-induced nephropathy and renal outcomes were reported as perioperative complications, the primary focus of the study was vascular endovascular aneurysm repair outcomes rather than contrast-associated acute kidney injury following interventional radiology procedures according to the predefined eligibility criteria for this review. |
| Li C, Wang Y, Wang X, et al. Acute kidney injury and cardiogenic shock severity for mortality risk stratification in patients supported with VA ECMO. ESC Heart Fail. 2024;11:3872–3881. https://doi.org/10.1002/ehf2.14967 | Wrong study focus/procedure population. This retrospective observational study evaluated acute kidney injury severity and mortality prediction in patients with cardiogenic shock receiving venoarterial extracorporeal membrane oxygenation support. The study did not involve interventional radiology procedures or evaluate contrast-associated acute kidney injury/contrast-induced nephropathy related to iodinated contrast exposure according to the predefined eligibility criteria for this review. |
| Lim DYZ, Goh JCH, He Y, et al. Contrast-Induced Acute Kidney Injury in Lower Limb Percutaneous Transluminal Angioplasty: A Machine Learning Approach for Preoperative Risk Prediction. Ann Vasc Surg. 2025;115:163–172. https://doi.org/10.1016/j.avsg.2025.01.043 | Wrong procedure specialty/population. This retrospective study evaluated contrast-induced acute kidney injury following lower limb percutaneous transluminal angioplasty in patients undergoing peripheral arterial endovascular intervention. The study population represented peripheral vascular intervention/peripheral arterial disease cohorts rather than patients undergoing interventional radiology procedures according to the predefined eligibility criteria for this review. |
| Lim J, Goh I, Gwini SM, et al. Long-term renal outcome post-multimodal computed tomography in stroke evaluation. Intern Med J. 2022;52:134–138. https://doi.org/10.1111/imj.15643 | Wrong procedure type/population. This retrospective study evaluated contrast-associated acute kidney injury following multimodal computed tomography imaging (CTA/CT perfusion) for acute stroke assessment. The study population underwent diagnostic neuroimaging rather than interventional radiology or endovascular therapeutic procedures according to the predefined eligibility criteria for this review. |
| Liss P, Eklöf H, Hellberg O, et al. Renal Effects of CO₂ and Iodinated Contrast Media in Patients Undergoing Renovascular Intervention: A Prospective, Randomized Study. J Vasc Interv Radiol. 2005;16:57–65. https://doi.org/10.1097/01.RVI.0000144807.81633.79 | Wrong procedure specialty/population. This prospective randomized study evaluated renal function following renovascular angiography and renal artery interventions for suspected renal artery stenosis, including angioplasty and stent placement procedures. The study population primarily represented renovascular/endovascular hypertension intervention practice rather than predefined interventional radiology procedural cohorts according to the eligibility criteria for this review. |
| Liu Z, Shang A, Chen Z, et al. Neutrophil gelatinase-associated lipocalin as an early predictor of contrast-induced nephropathy following endovascular therapy for arteriosclerosis obliterans. Medicine (Baltimore). 2020;99:e21386. https://doi.org/10.1097/MD.0000000000021386 | Wrong procedure specialty/population. This prospective cohort study evaluated contrast-induced nephropathy and renal biomarkers following endovascular therapy for arteriosclerosis obliterans in patients undergoing peripheral vascular intervention. The study population represented peripheral arterial disease/peripheral vascular intervention cohorts rather than patients undergoing interventional radiology procedures according to the predefined eligibility criteria for this review. |
| Liu J, Zhu H, Liu L. Comparison of intravenous hydration and oral hydration in preventing contrast-induced nephropathy among patients undergoing peripheral vascular interventions. BMC Nephrol. 2026;27:119. https://doi.org/10.1186/s12882-025-04737-5 | Wrong procedure specialty/population. This quasi-experimental study evaluated contrast-induced nephropathy prevention strategies in patients undergoing peripheral vascular intervention for peripheral vascular disease. The study population represented peripheral vascular intervention/peripheral arterial disease cohorts rather than patients undergoing interventional radiology procedures according to the predefined eligibility criteria for this review. |
| Liu C, Mor MK, Palevsky PM, et al. Postangiography Increases in Serum Creatinine and Biomarkers of Injury and Repair. Clin J Am Soc Nephrol. 2020;15:xxx–xxx. https://doi.org/10.2215/CJN.15931219 | This biomarker substudy evaluated contrast-associated acute kidney injury and renal injury biomarkers after coronary and noncoronary angiography in a mixed angiographic population from the PRESERVE trial. The study was not specific to interventional radiology procedures, and the majority of participants underwent diagnostic coronary angiography rather than IR-focused interventions according to the predefined eligibility criteria for this review. |
| Locham S, Rodriguez A, Balceniuk MD, et al. Contrast-Associated Acute Kidney Injury in High-Risk Patients Undergoing Peripheral Vascular Interventions. Vasc Endovascular Surg. 2023;57:583–591. https://doi.org/10.1177/15385744231162941 | Wrong procedure specialty/population. This retrospective VQI database study evaluated contrast-associated acute kidney injury in patients undergoing peripheral vascular intervention for peripheral arterial disease and chronic limb-threatening ischemia. The study population represented peripheral vascular intervention/peripheral arterial disease cohorts rather than patients undergoing interventional radiology procedures according to the predefined eligibility criteria for this review. |
| Ma B, James MT, Javaheri PA, et al. Change Management Accompanying Implementation of Decision Support for Prevention of Acute Kidney Injury in Cardiac Catheterization Units: Program Report. Can J Kidney Health Dis. 2023;10:1–13. https://doi.org/10.1177/20543581231206127 | Wrong procedure specialty/population and ineligible study design. This program report evaluated implementation of an electronic decision-support intervention for prevention of contrast-associated acute kidney injury in cardiac catheterization units undergoing coronary angiography and percutaneous coronary intervention. The study focused on workflow integration, provider satisfaction, and change management rather than incidence, risk factors, or clinical outcomes of CA-AKI following interventional radiology procedures according to the predefined eligibility criteria for this review. |
| Maaniitty T, Stenström I, Uusitalo V, et al. Incidence of Persistent Renal Dysfunction After Contrast Enhanced Coronary CT Angiography in Patients with Suspected Coronary Artery Disease. Int J Cardiovasc Imaging. 2016;32:1567–1575. https://doi.org/10.1007/s10554-016-0935-8 | Wrong procedure specialty/population. This retrospective study evaluated persistent renal dysfunction following contrast-enhanced coronary computed tomography angiography (CTA) in patients with suspected coronary artery disease. The study involved diagnostic cardiac CT imaging rather than interventional radiology procedures according to the predefined eligibility criteria for this review. |
| Macdonald DB, Hurrell CD, Costa AF, et al. Canadian Association of Radiologists Guidance on Contrast-Associated Acute Kidney Injury. Can J Kidney Health Dis. 2022;9:1–15. https://doi.org/10.1177/20543581221097455 | Ineligible study design. This article is a clinical practice guideline and narrative evidence-based consensus document on contrast-associated acute kidney injury, including recommendations regarding screening, prophylaxis, hydration, and contrast administration. It did not report original patient-level data on incidence, risk factors, or clinical outcomes of CA-AKI following interventional radiology procedures according to the predefined eligibility criteria for this review. |
| Madershahian N, Scherner M, Liakopoulos O, et al. Renal Impairment and Transapical Aortic Valve Implantation: Impact of Contrast Medium Dose on Kidney Function and Survival. Eur J Cardiothorac Surg. 2012;41:1225–1232. https://doi.org/10.1093/ejcts/ezr199 | Wrong procedure specialty/population. This retrospective study evaluated contrast-induced nephropathy following transapical transcatheter aortic valve implantation (TA-AVI), a structural cardiac surgery/cardiology procedure performed for severe aortic stenosis. The study population was not specific to interventional radiology procedures according to the predefined eligibility criteria for this review. |
| Maioli M, Toso A, Leoncini M, et al. Bioimpedance-Guided Hydration for the Prevention of Contrast-Induced Kidney Injury: The HYDRA Study. J Am Coll Cardiol. 2018;71:2880–2889. https://doi.org/10.1016/j.jacc.2018.04.022 | Wrong procedure specialty/population. This randomized study evaluated bioimpedance-guided hydration strategies for prevention of contrast-induced acute kidney injury in patients undergoing elective coronary angiographic procedures and/or angioplasty for stable coronary artery disease. The study population involved cardiac catheterization/coronary intervention procedures rather than interventional radiology procedures according to the predefined eligibility criteria for this review. |
| Maioli M, Toso A, Leoncini M, et al. Pre-Procedural Bioimpedance Vectorial Analysis of Fluid Status and Prediction of Contrast-Induced Acute Kidney Injury. J Am Coll Cardiol. 2014;63:1387–1394. https://doi.org/10.1016/j.jacc.2014.01.025 | Wrong procedure specialty/population. This prospective study evaluated bioimpedance-guided assessment of fluid status and prediction of contrast-induced acute kidney injury in patients undergoing elective coronary angiography and percutaneous coronary intervention for stable coronary artery disease. The study population involved cardiac catheterization/coronary intervention procedures rather than interventional radiology procedures according to the predefined eligibility criteria for this review. |
| Mankerious N, Hemetsberger R, Samy M, et al. The Target Vessel SYNTAX Score: A Novel Pre-Procedural Predictor for Contrast-Induced Acute Kidney Injury After Rotational Atherectomy. Cardiovasc Revasc Med. 2023;47:18–24. https://doi.org/10.1016/j.carrev.2022.08.035 | Wrong procedure specialty/population. This study evaluated predictors of contrast-induced acute kidney injury following rotational atherectomy during complex percutaneous coronary intervention procedures. The study population involved interventional cardiology/coronary revascularization procedures rather than interventional radiology procedures according to the predefined eligibility criteria for this review. |
| Mariajoseph FP, Lai LT, Moore J, et al. Incidence of Contrast-Induced Neurotoxicity Following Endovascular Treatment of Unruptured Intracranial Aneurysms: A Single-Centre Cohort Study. Acta Neurol Belg. 2024;124:1989–1994. https://doi.org/10.1007/s13760-024-02643-5 | Wrong outcome. This retrospective cohort study evaluated the incidence and predictors of contrast-induced neurotoxicity following endovascular treatment of unruptured intracranial aneurysms. The primary outcome was neurological toxicity related to iodinated contrast administration rather than contrast-associated acute kidney injury or renal outcomes according to the predefined eligibility criteria for this review. |
| Markota D, Markota I, Starčević B, et al. Prevention of Contrast-Induced Nephropathy with Na/K Citrate. Eur Heart J. 2013;34:2362–2367. https://doi.org/10.1093/eurheartj/eht009 | Wrong procedure specialty/population. This randomized study evaluated prevention of contrast-induced nephropathy using Na/K citrate in patients undergoing coronary angiography/coronarography and percutaneous coronary intervention procedures. The study population involved interventional cardiology procedures rather than interventional radiology procedures according to the predefined eligibility criteria for this review. |
| Marquez-Romero JM, Zenteno M, Arauz A. Changes in Blood and Renal Function in Patients After Cerebral Digital Subtraction Angiography. Res Diagn Interv Imaging. 2023;7:100032. https://doi.org/10.1016/j.redii.2023.100032 | Wrong procedure type. This prospective cohort study evaluated contrast-induced acute kidney injury following elective cerebral digital subtraction angiography (DSA), which was performed as a diagnostic angiographic procedure rather than an interventional radiology therapeutic procedure according to the predefined eligibility criteria for this review. |
| Martínez-Rico C, Martí-Mestre X, Romera-Villegas A, et al. Contrast-Induced Nephropathy: Fact or Fiction in Lower Limb Revascularisation? Ann Vasc Surg. 2017. https://doi.org/10.1016/j.avsg.2017.03.193 | Wrong procedure specialty/population. This prospective observational study evaluated contrast-induced nephropathy following lower limb endovascular revascularization procedures in patients with peripheral arterial disease. The study population primarily represented peripheral vascular intervention and vascular/endovascular practice rather than predefined interventional radiology procedural cohorts according to the eligibility criteria for this review. |
| Massaini G, Lazzarotto T, Masciello F, et al. A Pilot Study of Endovascular Repair for Ruptured Aortic Aneurysms With the Use of Carbon Dioxide Angiography Alone. J Endovasc Ther. 2025;32:498–502. https://doi.org/10.1177/15266028231180995 | Wrong procedure specialty/population. This pilot study evaluated acute kidney injury following endovascular aortic repair (EVAR) for ruptured abdominal aortic aneurysm using carbon dioxide angiography. The study population primarily represented vascular surgery/endovascular aneurysm repair practice rather than predefined interventional radiology procedural cohorts according to the eligibility criteria for this review. |
| Matsumoto MM, Reddy SN, Nadolski GJ, et al. Iatrogenic Renal Artery Injury in 90 Cases: Arteriographic Findings and Outcomes after Embolization for Bleeding. J Vasc Interv Radiol. 2023;34:436–444. https://doi.org/10.1016/j.jvir.2022.11.016 | Renal hemorrhage/embolization population. This retrospective cohort study evaluated outcomes after embolization for iatrogenic renal artery injury following partial nephrectomy, renal biopsy, and percutaneous renal access procedures. Acute kidney injury was evaluated as a secondary procedural safety outcome in the context of renal hemorrhage and embolization management, rather than as a primary contrast-associated acute kidney injury population according to the predefined eligibility criteria for this review. |
| Matsushima K, Peng M, Schaefer EW, et al. Posttraumatic Contrast-Induced Acute Kidney Injury: Minimal Consequences or Significant Threat? J Trauma. 2011;70:415–420. https://doi.org/10.1097/TA.0b013e3182095fa6 | Wrong population/procedure type. This retrospective trauma study evaluated contrast-induced acute kidney injury after intravenous contrast administration for diagnostic imaging and angiographic studies in injured trauma patients. The study was not specific to interventional radiology procedures or endovascular interventions according to the predefined eligibility criteria for this review. |
| Moghaddam AA, Rahimi VB, Morovatdar N, et al. Oral Nicorandil Effectively Attenuates the Incidence of Contrast-Induced Nephropathy in Patients Undergoing Cardiac Catheterization: A Randomized, Controlled, Open-Label Clinical Trial. Int Urol Nephrol. 2023;55:2327–2334. https://doi.org/10.1007/s11255-023-03541-0 | Wrong procedure specialty/population. This randomized clinical trial evaluated prevention of contrast-induced nephropathy with Nicorandil in patients undergoing cardiac catheterization for coronary artery disease. The study population involved interventional cardiology/coronary procedures rather than interventional radiology procedures according to the predefined eligibility criteria for this review. |
| Mohebi R, van Kimmenade R, McCarthy C, et al. A Biomarker-Enhanced Model for Prediction of Acute Kidney Injury and Cardiovascular Risk Following Angiographic Procedures: CASABLANCA AKI Prediction Substudy. J Am Heart Assoc. 2022;11:e025729. https://doi.org/10.1161/JAHA.122.025729 | Wrong procedure specialty/population. This prospective cohort substudy evaluated biomarker-based prediction of contrast-associated acute kidney injury in patients undergoing coronary and/or peripheral angiography, primarily within a cardiovascular catheterization population. The study focused on prediction modeling and cardiovascular angiographic procedures rather than interventional radiology procedures according to the predefined eligibility criteria for this review. |
| Mola S, Askin G, Aytekin B, et al. Endovascular aortic repair via carbon-dioxide imaging in renal insufficiency? Turk J Vasc Surg. 2022;31:178–185. https://doi.org/10.9739/tjvs.2022.09.015 | Wrong procedure specialty/population. This retrospective study evaluated renal outcomes and contrast-induced nephropathy following carbon dioxide-guided endovascular aortic repair (EVAR) for infrarenal abdominal aortic aneurysm in patients with renal insufficiency. The study population primarily represented vascular surgery/endovascular aneurysm repair practice rather than predefined interventional radiology procedural cohorts according to the eligibility criteria for this review. |
| Mou Z, Guan T, Chen L. Acute Kidney Injury in Adult Patients With Hepatocellular Carcinoma After TACE or Hepatectomy Treatment. Front Oncol. 2022;12:627895. https://doi.org/10.3389/fonc.2022.627895 . | Ineligible study design. This article was a systematic review and meta-analysis evaluating acute kidney injury after transarterial chemoembolization (TACE) or hepatectomy in patients with hepatocellular carcinoma. The study did not provide original patient-level data from a primary interventional radiology cohort according to the predefined eligibility criteria for this review. |
| Mun JH, Kwon SK, Park JH, et al. Renal function-adjusted contrast medium volume is a major risk factor in the occurrence of acute kidney injury after endovascular aneurysm repair. Medicine (Baltimore). 2021;100:e25381. https://doi.org/10.1097/MD.0000000000025381 | Wrong procedure specialty/population. This retrospective cohort study evaluated acute kidney injury and contrast-related renal risk factors following elective endovascular aneurysm repair (EVAR) for infrarenal abdominal aortic aneurysm. The study population primarily represented vascular surgery/endovascular aneurysm repair practice rather than predefined interventional radiology procedural cohorts according to the eligibility criteria for this review. |
| Muratoglu M, Kavalci C, Kilicli E, et al. Serum Neutrophil Gelatinase-Associated Lipocalin Levels in Early Detection of Contrast-Induced Nephropathy. Clin Invest Med. 2016;38:E88–E94. https://doi.org/10.25011/cim.v39i3.26797 | Wrong procedure type/population. This prospective study evaluated serum NGAL levels for early detection of contrast-induced nephropathy in patients undergoing contrast-enhanced abdominal computed tomography rather than interventional radiology or endovascular procedures according to the predefined eligibility criteria for this review. |
| Murugan R, Boudreaux-Kelly MY, Kellum JA, et al. Contrast-Associated Acute Kidney Injury and Cardiovascular Events: A Secondary Analysis of the PRESERVE Cohort. Clin Kidney J. 2023;16:2626–2638. https://doi.org/10.1093/ckj/sfad214 | Wrong procedure specialty/population and ineligible study focus. This secondary analysis of the PRESERVE trial evaluated cardiovascular outcomes and biomarkers in chronic kidney disease patients undergoing predominantly coronary angiography procedures. The study primarily focused on cardiovascular events and biomarker mediation analysis rather than incidence, risk factors, or renal outcomes specific to interventional radiology procedures according to the predefined eligibility criteria for this review. |
| Nam IC, Lee ES, Shin JH, et al. Cardiac Arrest during Interventional Radiology Procedures: A 7-Year Single-Center Retrospective Study. J Clin Med. 2022;11:511. https://doi.org/10.3390/jcm11030511 | Wrong outcome. This retrospective study evaluated cardiac arrest events and predictors during interventional radiology procedures. The primary outcome focused on cardiopulmonary arrest and procedural mortality rather than contrast-associated acute kidney injury, contrast-induced nephropathy, or renal outcomes according to the predefined eligibility criteria for this review. |
| Nardi G, Marchi E, Allinovi M, et al. Contrast-Induced Acute Kidney Injury in Patients with Heart Failure on Sodium–Glucose Cotransporter-2 Inhibitors Undergoing Radiocontrast Agent Invasive Procedures: A Propensity-Matched Analysis. J Clin Med. 2024;13:2041. https://doi.org/10.3390/jcm13072041 | Wrong procedure specialty/population. This retrospective propensity-matched study primarily evaluated contrast-induced acute kidney injury prevention in patients with heart failure undergoing coronary angiography, percutaneous coronary intervention, and structural cardiology procedures using iodinated contrast media. The study population mainly involved interventional cardiology procedures rather than interventional radiology procedures according to the predefined eligibility criteria for this review. |
| Newgard BJ, Getrajdman GI, Erinjeri JP, et al. Incidence and Consequence of Nontarget Embolization Following Bland Hepatic Arterial Embolization. Cardiovasc Intervent Radiol. 2019;42:1135–1141. https://doi.org/10.1007/s00270-019-02229-2 | Wrong outcome. This retrospective cohort study evaluated the incidence and consequences of nontarget embolization following bland hepatic arterial embolization. The primary focus was procedural complications related to nontarget embolization rather than contrast-associated acute kidney injury or renal outcomes. Although one patient developed contrast-induced nephropathy, renal injury was not a primary or systematically evaluated outcome according to the predefined eligibility criteria for this review. |
| Ni Z, Liang Y, Xie N, et al. Simple Pre-Procedure Risk Stratification Tool for Contrast-Induced Nephropathy. J Thorac Dis. 2019;11:1597–1610. https://doi.org/10.21037/jtd.2019.04.69 | Wrong procedure specialty/population. This retrospective study developed and validated a risk prediction model for contrast-induced nephropathy in patients undergoing coronary angiography and percutaneous coronary intervention. The study population involved interventional cardiology procedures rather than interventional radiology procedures according to the predefined eligibility criteria for this review. |
| Nijssen EC, Nelemans PJ, Rennenberg RJ, et al. Prophylactic Intravenous Hydration to Protect Renal Function From Intravascular Iodinated Contrast Material (AMACING): Long-term Results of a Prospective, Randomised, Controlled Trial. EClinicalMedicine. 2018;4–5:109–116. https://doi.org/10.1016/j.eclinm.2018.10.007 | Wrong population/procedure type. This randomized controlled trial evaluated prophylactic hydration strategies for prevention of contrast-induced nephropathy in patients undergoing mixed elective intravenous and intra-arterial iodinated contrast procedures across multiple specialties, including coronary angiography and CT imaging. The study population was not specific to interventional radiology procedures according to the predefined eligibility criteria for this review. |
| Ntalouka MP, Nana P, Brotis A, et al. Predictors of 30-Day Postoperative Outcome after Elective Endovascular Abdominal Aortic Aneurysm Repair: A Tertiary Referral Center Experience. J Clin Med. 2023;12:6004. https://doi.org/10.3390/jcm12186004 | Wrong procedure specialty/population. This retrospective cohort study evaluated postoperative outcomes, including acute kidney injury, following elective endovascular abdominal aortic aneurysm repair (EVAR). The study population primarily represented vascular surgery/endovascular aneurysm repair practice rather than predefined interventional radiology procedural cohorts according to the eligibility criteria for this review. |
| Olariu N, Maralescu FM, Bob F, et al. Contrast-Induced Nephropathy in Endovascular Patients: A Retrospective Cohort Study from a Vascular Surgery Clinic in Eastern Europe. J Clin Med. 2025;14:1172. https://doi.org/10.3390/jcm14041172 | Wrong procedure specialty/population. This retrospective cohort study evaluated contrast-induced nephropathy following peripheral endovascular procedures performed in a vascular surgery setting. The study population primarily represented peripheral vascular intervention and vascular/endovascular surgery practice rather than predefined interventional radiology procedural cohorts according to the eligibility criteria for this review. |
| Owais T, El Garhy M, Fuchs J, et al. Transcatheter Aortic Valve Replacement Without the Use of Contrast Medium: An Alternative Safe Implantation Technique. J Invasive Cardiol. 2020;32:Epub ahead of print. http://doi.org/10.25270/jic/20.00050 | Wrong procedure specialty/population. This retrospective study evaluated acute kidney injury after transcatheter aortic valve implantation/replacement (TAVI/TAVR) using contrast-free implantation techniques in patients with severe aortic valve stenosis. The study population involved structural heart/interventional cardiology procedures rather than interventional radiology or peripheral endovascular procedures according to the predefined eligibility criteria for this review. |
| Pakfetrat M, Nikoo MH, Malekmakan L, et al. Comparison of Risk Factors for Contrast-Induced Acute Kidney Injury Between Patients With and Without Diabetes. Hemodial Int. 2010;14:387–392. https://doi.org/10.1111/j.1542-4758.2010.00469.x | Wrong procedure specialty/population. This prospective study evaluated risk factors for contrast-induced acute kidney injury in patients undergoing coronary angiography or percutaneous coronary intervention. The study population involved interventional cardiology procedures rather than interventional radiology or peripheral endovascular procedures according to the predefined eligibility criteria for this review. |
| Para SA, Mehdi S, Malik S, et al. Safety and Efficacy of Selective Renal Artery Embolization in the Management of Postprocedural Acute Renal Bleeding: Experience of A Tertiary Care Center. J Urol Surg. 2023;10:326–333. https://doi.org/10.4274/jus.galenos.2023.2022.0097 | Wrong outcome/study focus. This prospective observational study evaluated the safety and efficacy of selective renal artery embolization for management of postprocedural renal hemorrhage following urological procedures such as percutaneous nephrolithotomy, nephron-sparing surgery, nephrostomy, and renal biopsy. The primary focus was hemorrhage control and embolization outcomes rather than contrast-associated acute kidney injury or renal outcomes according to the predefined eligibility criteria for this review. |
| Peng M, Jiang XJ, Dong H, et al. A Comparison of Nephrotoxicity of Contrast Medium in Elderly Patients who Underwent Renal or Peripheral Arterial Vascular Intervention. Intern Med. 2016;55:9–14. https://doi.org/10.2169/internalmedicine.55.5321 | Wrong procedure specialty/population. This prospective cohort study evaluated contrast-induced nephropathy following renal artery and peripheral arterial vascular interventions in elderly patients undergoing endovascular revascularization procedures. The study population primarily represented peripheral vascular and renovascular intervention practice rather than predefined interventional radiology procedural cohorts according to the eligibility criteria for this review. |
| Peres J, Toregeani JF, Pohl AC, et al. Incidence and risk factors for contrast-induced nephropathy after angioplasty: an observational retrospective study. J Vasc Bras. 2025;24:e20250028. https://doi.org/10.1590/1677-5449.202500282 | Wrong procedure specialty/population. This retrospective observational study evaluated contrast-induced nephropathy following heterogeneous angioplasty procedures, including coronary, renal, carotid, lower limb, and peripheral vascular interventions. The study population primarily represented generalized vascular/endovascular revascularization practice rather than predefined interventional radiology procedural cohorts according to the eligibility criteria for this review. |
| Piasecki P, Ząbkowski T, Brzozowski K, et al. The Assessment of the Risk of Acute Kidney Injury in Patients Undergoing an Urgent Endovascular Treatment Due to Severe Renal Bleeding. Cardiovasc Intervent Radiol. 2018;41:398–405. https://doi.org/10.1007/s00270-017-1800-4 | Wrong primary study focus/outcome framework. This retrospective study evaluated acute kidney injury risk in patients undergoing urgent renal artery embolization for severe renal bleeding after trauma or iatrogenic injury. Although renal outcomes and contrast exposure were assessed, the primary focus was management of acute hemorrhage and embolization-related outcomes in critically ill bleeding patients rather than contrast-associated acute kidney injury following planned interventional radiology or vascular endovascular procedures according to the predefined eligibility criteria for this review. |
| Piffaretti G, Mariscalco G, Bonardelli S, et al. Predictors and outcomes of acute kidney injury after thoracic aortic endograft repair. J Vasc Surg. 2012;56:1527–1534. https://doi.org/10.1016/j.jvs.2012.05.106 | Wrong procedure specialty/population. This multicenter cohort study evaluated acute kidney injury following thoracic endovascular aortic repair (TEVAR) and thoracoabdominal endograft procedures. The study population primarily represented vascular surgery/endovascular aortic repair practice rather than predefined interventional radiology procedural cohorts according to the eligibility criteria for this review. |
| Powell SP, Chawla R, Bottinor W, et al. Race, Contrast-Induced Nephropathy and Long-Term Outcomes after Coronary and Peripheral Angiography and Intervention. Cardiovasc Revasc Med. 2018;19:S31–S35. https://doi.org/10.1016/j.carrev.2018.06.016 | Mixed and predominantly coronary procedure population. This retrospective cohort study evaluated contrast-induced nephropathy after a heterogeneous group of coronary and peripheral angiography/intervention procedures, with a substantial proportion of patients undergoing coronary angiography and percutaneous coronary intervention. The study population was not specific to interventional radiology or vascular endovascular procedures according to the predefined eligibility criteria for this review. |
| Prasad A, Hughston H, Michalek J, et al. Acute kidney injury in patients undergoing endovascular therapy for critical limb ischemia. Catheter Cardiovasc Interv. 2019;94:636–641. https://doi.org/10.1002/ccd.28415 | Wrong procedure specialty/population. This study evaluated acute kidney injury following peripheral endovascular interventions for critical limb ischemia using a nationwide administrative database of peripheral vascular procedures, including angioplasty, stenting, and atherectomy. The study population primarily represented peripheral vascular intervention and interventional cardiology/endovascular practice rather than predefined interventional radiology procedural populations according to the eligibility criteria for this review. |
| Prasad A, Ortiz-Lopez C, Khan A, et al. Acute Kidney Injury Following Peripheral Angiography and Endovascular Therapy: A Systematic Review of the Literature. Catheter Cardiovasc Interv. 2016;88:264–273. https://doi.org/10.1002/ccd.26466 | Ineligible study design. This article was a systematic review evaluating acute kidney injury following peripheral angiography and endovascular therapy for peripheral arterial disease. The study did not provide original patient-level data from a primary cohort according to the predefined eligibility criteria for this review. |
| Prasad A, Palevsky PM, Bansal S, et al. Management of Patients With Kidney Disease in Need of Cardiovascular Catheterization: A Scientific Workshop Cosponsored by the National Kidney Foundation and the Society for Cardiovascular Angiography and Interventions. J Soc Cardiovasc Angiogr Interv. 2022;1:100445. https://doi.org/10.1016/j.jscai.2022.100445 | Ineligible study design. This article was a scientific workshop consensus review summarizing evidence and recommendations regarding acute kidney injury prevention and management in patients with chronic kidney disease undergoing cardiovascular catheterization procedures. The publication did not provide original patient-level data from a primary interventional radiology or endovascular cohort according to the predefined eligibility criteria for this review. |
| Prasad A, Sohn A, Morales J, et al. Contemporary Practice Patterns Related to the Risk of Acute Kidney Injury in the Catheterization Laboratory: Results from a Survey of Society of Cardiovascular Angiography and Intervention (SCAI) Cardiologists. Catheter Cardiovasc Interv. 2017;89:383–392. https://doi.org/10.1002/ccd.26628 | Ineligible study design. This study was a survey-based analysis of cardiologists’ practice patterns regarding acute kidney injury risk assessment and prevention during coronary and peripheral angiography. The publication did not provide original patient-level clinical outcome data regarding contrast-associated acute kidney injury following interventional radiology or |
| Quaglino S, Ferrero E, Ferri M, et al. Safety, Effectiveness and Pitfalls of Carbon Dioxide Routine Use as a Contrast Agent for Endovascular Abdominal Aortic Repair. Ann Vasc Surg. 2024;101:120–126. https://doi.org/10.1016/j.avsg.2023.10.009 | Wrong procedure specialty/population. This retrospective cohort study evaluated renal outcomes and technical feasibility of carbon dioxide-guided endovascular abdominal aortic aneurysm repair (EVAR). The study population primarily represented vascular surgery/endovascular aneurysm repair practice rather than predefined interventional radiology procedural cohorts according to the eligibility criteria for this review. |
| Ram P, Mezue K, Pressman G, et al. Acute Kidney Injury Post–Transcatheter Aortic Valve Replacement. Clin Cardiol. 2017;40:1355–1360. https://doi.org/10.1002/clc.22820 | Ineligible study design and wrong procedure specialty. This article was a narrative review summarizing acute kidney injury following transcatheter aortic valve replacement (TAVR). The publication did not provide original patient-level data, and the procedure focus was structural heart/interventional cardiology rather than interventional radiology or vascular endovascular procedures according to the predefined eligibility criteria for this review. |
| Ribichini F, Gambaro A, Pighi M, et al. Effects of Prednisone on Biomarkers of Tubular Damage Induced by Radiocontrast in Interventional Cardiology. J Nephrol. 2013;26:586–593. https://doi.org/10.5301/jn.5000266 | Wrong procedure specialty/population. This pilot study evaluated biomarkers of renal tubular injury and prevention of contrast-induced nephropathy in patients undergoing diagnostic and therapeutic cardiovascular/interventional cardiology procedures, including coronary angiography and percutaneous coronary intervention. The study population was not specific to interventional radiology or peripheral/neurovascular endovascular procedures according to the predefined eligibility criteria for this review. |
| Ribitsch W, Schilcher G, Quehenberger F, et al. Neutrophil Gelatinase-Associated Lipocalin (NGAL) Fails as an Early Predictor of Contrast Induced Nephropathy in Chronic Kidney Disease (ANTI-CI-AKI Study). Sci Rep. 2017;7:41300. https://doi.org/10.1038/srep41300 | Mixed and predominantly coronary procedure population. This prospective randomized study evaluated contrast-induced acute kidney injury biomarkers in patients undergoing mixed intra-arterial angiographic procedures, the majority of whom underwent coronary angiography/percutaneous coronary intervention. Peripheral vascular procedures were not analyzed separately, and the study population was not specific to interventional radiology or peripheral/neurovascular endovascular procedures according to the predefined eligibility criteria for this review. |
| Ribitsch W, Horina JH, Quehenberger F, et al. Contrast Induced Acute Kidney Injury and its Impact on Mid-Term Kidney Function, Cardiovascular Events and Mortality. Sci Rep. 2019;9:16896. https://doi.org/10.1038/s41598-019-53040-5 | Mixed and predominantly coronary procedure population. This prospective observational study evaluated contrast-induced acute kidney injury after mixed intra-arterial angiographic procedures, including coronary angiography/percutaneous coronary intervention and peripheral vascular angiography/angioplasty. Coronary procedures represented the majority of the cohort, and outcomes for peripheral vascular/endovascular procedures were not analyzed separately according to the predefined eligibility criteria for this review. |
| Sadat U, Walsh SR, Norden AG, et al. Does Oral N-Acetylcysteine Reduce Contrast-Induced Renal Injury in Patients With Peripheral Arterial Disease Undergoing Peripheral Angiography? A Randomized-Controlled Study. Angiology. 2011;62:225–230. https://doi.org/10.1177/0003319710377078 | Wrong procedure specialty/population. This randomized controlled study evaluated prevention of contrast-induced nephropathy following peripheral arterial angiography in patients with peripheral arterial disease. The study population primarily represented peripheral vascular imaging/intervention practice rather than predefined interventional radiology procedural cohorts according to the eligibility criteria for this review. |
| Sadineni R, Karthik KR, Swarnalatha G, et al. N-acetyl Cysteine versus Allopurinol in the Prevention of Contrast Nephropathy in Patients with Chronic Kidney Disease: A Randomized Controlled Trial. Indian J Nephrol. 2017;27:93–98. https://doi.org/10.4103/0971-4065.194397 | Wrong procedure specialty/population. This randomized controlled trial evaluated prevention of contrast-induced nephropathy in patients with chronic kidney disease undergoing coronary angiography and percutaneous coronary intervention. The study population involved interventional cardiology procedures rather than interventional radiology or peripheral/neurovascular endovascular procedures according to the predefined eligibility criteria for this review. |
| Safley DM, Salisbury AC, Tsai TT, et al. Acute Kidney Injury Following In-Patient Lower Extremity Vascular Intervention From the National Cardiovascular Data Registry. JACC Cardiovasc Interv. 2021;14:333–341. https://doi.org/10.1016/j.jcin.2020.10.028 | Wrong procedure specialty/population. This study evaluated acute kidney injury following lower extremity peripheral vascular interventions using the National Cardiovascular Data Registry Peripheral Vascular Intervention (NCDR PVI) Registry, including angioplasty, stenting, and atherectomy procedures for peripheral arterial disease and critical limb ischemia. The study population primarily represented peripheral vascular intervention and cardiovascular/endovascular practice rather than predefined interventional radiology procedural populations according to the eligibility criteria for this review. |
| Sailer AM, Nelemans PJ, van Berlo C, et al. Endovascular treatment of complex aortic aneurysms: prevalence of acute kidney injury and effect on long-term renal function. Eur Radiol. 2016;26:1613–1619. https://doi.org/10.1007/s00330-015-3993-8 | Wrong procedure specialty/population. This retrospective cohort study evaluated acute kidney injury and long-term renal function following fenestrated and branched endovascular aortic repair (EVAR) for complex thoracoabdominal aneurysms. The study population primarily represented vascular surgery/endovascular aortic repair practice rather than predefined interventional radiology procedural cohorts according to the eligibility criteria for this review. |
| Saour M, Charbit J, Millet I, et al. Effect of Renal Angioembolization on Post-Traumatic Acute Kidney Injury After High-Grade Renal Trauma: A Comparative Study of 52 Consecutive Cases. Injury. 2014;45:894–901. https://doi.org/10.1016/j.injury.2013.11.030 | Wrong clinical context/population. This retrospective study evaluated acute kidney injury after renal angioembolization in patients with high-grade traumatic renal injuries managed in a trauma setting. Although the study involved interventional radiology embolization procedures and assessed AKI outcomes, the primary clinical context was traumatic renal injury management rather than contrast-associated acute kidney injury following elective or vascular/endovascular interventional radiology procedures according to the predefined eligibility criteria for this review. |
| Saratzis A, Shakespeare J, Jones O, et al. Pre-operative Functional Cardiovascular Reserve Is Associated with Acute Kidney Injury after Intervention. Eur J Vasc Endovasc Surg. 2017;53:717–724. https://doi.org/10.1016/j.ejvs.2017.01.014 | Wrong study focus/outcome framework. This case-control study evaluated the association between pre-operative cardiovascular reserve and acute kidney injury after elective infrarenal endovascular aneurysm repair (EVAR). Although AKI following EVAR was assessed, the primary objective focused on cardiopulmonary reserve and physiological predictors rather than contrast-associated acute kidney injury, contrast exposure, or procedure-related renal injury according to the predefined eligibility criteria for this review. |
| Saratzis AN, Bath MF, Harrison SC, et al. Impact of Fenestrated Endovascular Abdominal Aortic Aneurysm Repair on Renal Function. J Endovasc Ther. 2015;22:889–896. https://doi.org/10.1177/1526602815605311 | Wrong procedure specialty/population. This case-controlled study evaluated acute kidney injury and renal function following fenestrated endovascular abdominal aortic aneurysm repair (fEVAR) for juxtarenal and short-neck abdominal aortic aneurysms. The study population primarily represented vascular surgery/endovascular aneurysm repair practice rather than predefined interventional radiology procedural cohorts according to the eligibility criteria for this review. |
| Savoie-White FH, Gegiia I, Gorak Savard R, et al. Long-term Results of the Neo-Aorto-Iliac System Procedure as a Compelling Choice for the Treatment of Aortic Infections. J Vasc Surg. 2026;83:598–606. https://doi.org/10.1016/j.jvs.2025.09.038 | Wrong study focus/outcome framework. This retrospective cohort study evaluated long-term outcomes, patency, survival, and complications after neo-aorto-iliac system reconstruction for aortic infections. Although postoperative acute kidney injury was reported as a complication, the primary focus was management of infected aortic grafts and aortic infections rather than contrast-associated acute kidney injury following interventional radiology or endovascular contrast procedures according to the predefined eligibility criteria for this review. |
| Schilcher G, Ribitsch W, Otto R, et al. Early Detection and Intervention Using Neutrophil Gelatinase-Associated Lipocalin (NGAL) May Improve Renal Outcome of Acute Contrast Media Induced Nephropathy: A Randomized Controlled Trial in Patients Undergoing Intra-Arterial Angiography (ANTI-CIN Study). BMC Nephrol. 2011;12:39. https://doi.org/10.1186/1471-2369-12-39 | Ineligible study design and mixed procedure population. This publication was a study protocol describing a planned randomized controlled trial evaluating NGAL-guided intervention for contrast-induced nephropathy after mixed intra-arterial angiographic procedures, including coronary, peripheral, and renal angiography/intervention populations. The article did not report original patient outcome data and the study population was not specific to interventional radiology or peripheral/neurovascular endovascular procedures according to the predefined eligibility criteria for this review. |
| Schönefeld E, Höwler S, Osada N, et al. Effectiveness of Nephroprotection by the Selection of Contrast Media used during Vascular Interventions in Patients with Chronic Renal Failure. Zentralbl Chir. 2011;136:426–430. https://doi.org/10.1055/s-0031-1283759 | Wrong procedure specialty/population. This prospective comparison study evaluated contrast-induced nephropathy and contrast media selection in patients with chronic renal insufficiency undergoing angiography with or without vascular intervention. The study population primarily represented generalized vascular/endovascular intervention practice rather than predefined interventional radiology procedural cohorts according to the eligibility criteria for this review. |
| Scridon A, Somkereki C, Nicoară TR, et al. Neutrophil gelatinase-associated lipocalin monitoring reveals persistent subclinical kidney injury following intraarterial administration of iodinated contrast agents. Sci Rep. 2022;12:19464. https://doi.org/10.1038/s41598-022-24169-7 | Wrong procedure specialty/population. This prospective study evaluated contrast-induced nephropathy and subclinical kidney injury following elective percutaneous angioplasty procedures involving intraarterial contrast administration. The study population primarily represented cardiovascular/interventional cardiology angioplasty procedures rather than predefined interventional radiology procedural populations according to the eligibility criteria for this review. |
| Shan XS, Dai HR, Zhao D, et al. Dexmedetomidine reduces acute kidney injury after endovascular aortic repair of Stanford type B aortic dissection: A randomized, double-blind, placebo-controlled pilot study. J Clin Anesth. 2021;75:110498. https://doi.org/10.1016/j.jclinane.2021.110498 | Wrong procedure specialty/population. This randomized controlled trial evaluated acute kidney injury prevention following endovascular aortic repair (EVAR) for Stanford type B aortic dissection. The study population primarily represented cardiovascular surgery/endovascular aortic repair practice rather than predefined interventional radiology procedural cohorts according to the eligibility criteria for this review. |
| Shen G, Dai Y, Wang K, et al. CO₂ Angiography Offers Clinical Advantages Over Iodinated Contrast in Endovascular Aneurysm Repair: A Systematic Review and Meta-Analysis. BMC Surg. 2025;25:578. https://doi.org/10.1186/s12893-025-03329-2 | Ineligible study design. This article is a systematic review and meta-analysis evaluating carbon dioxide versus iodinated contrast media in endovascular aneurysm repair (EVAR). The study did not report original patient-level data and therefore does not meet the predefined eligibility criteria requiring primary clinical studies for inclusion in this review. |
| Shen J, Karki M, Jiang T, et al. Complications Associated With Diagnostic Cerebral Angiography: A Retrospective Analysis of 644 Consecutive Cerebral Angiographic Cases. Neurol India. 2018;66:1154–1158. https://doi.org/10.4103/0028-3886.237018 | Wrong procedure focus. This retrospective study primarily evaluated complications following diagnostic cerebral angiography procedures rather than therapeutic neurointerventional or endovascular interventions. Although contrast-induced nephropathy was reported as a secondary complication, the study population consisted predominantly of diagnostic angiographic procedures and did not align with the predefined eligibility criteria focused on interventional radiology and therapeutic endovascular procedures. |
| Sigterman TA, Bolt LJJ, Krasznai AG, et al. Loss of Kidney Function After Endovascular Treatment of Peripheral Arterial Disease. Ann Vasc Surg. 2016. https://doi.org/10.1016/j.avsg.2016.07.100 | Wrong study focus/outcome framework. This retrospective cohort study evaluated long-term renal decline after endovascular treatment of intermittent claudication and peripheral arterial disease, comparing endovascular intervention with supervised exercise therapy. Although iodinated contrast exposure was discussed as a potential mechanism for renal decline, the study did not directly evaluate contrast-associated acute kidney injury or contrast-induced nephropathy as a primary endpoint according to the predefined eligibility criteria for this review. |
| Sigterman TA, Krasznai AG, Snoeijs MG, et al. Contrast Induced Nephropathy and Long-term Renal Decline After Percutaneous Transluminal Angioplasty for Symptomatic Peripheral Arterial Disease. Eur J Vasc Endovasc Surg. 2016;51:386–393. https://doi.org/10.1016/j.ejvs.2015.08.023 | Wrong procedure specialty/population. This prospective cohort study evaluated contrast-induced nephropathy and long-term renal decline following percutaneous transluminal angioplasty for symptomatic peripheral arterial disease. The study population primarily represented peripheral vascular intervention practice rather than predefined interventional radiology procedural cohorts according to the eligibility criteria for this review. |
| Siriwardana RC, Niriella MA, Dassanayake AS, et al. Factors Affecting Post-Embolization Fever and Liver Failure After Trans-Arterial Chemo-Embolization in a Cohort Without Background Infective Hepatitis: A Prospective Analysis. BMC Gastroenterol. 2015;15:96. https://doi.org/10.1186/s12876-015-0329-8 | Wrong primary study focus/outcome framework. This prospective study primarily evaluated post-embolization fever and acute hepatic decompensation following transarterial chemoembolization for hepatocellular carcinoma. Although acute kidney injury was reported as a secondary complication, contrast-associated acute kidney injury or contrast-induced nephropathy was not a primary study endpoint according to the predefined eligibility criteria for this review. |
| Speir EJ, Newsome JM, Bercu ZL, et al. Correlation of CT Angiography and 99mTechnetium-Labeled Red Blood Cell Scintigraphy to Catheter Angiography for Lower Gastrointestinal Bleeding: A Single Institution Experience. J Vasc Interv Radiol. 2019. https://doi.org/10.1016/j.jvir.2019.04.019 | Wrong primary study focus/outcome framework. This retrospective study primarily compared computed tomography angiography and radionuclide scintigraphy for localization of lower gastrointestinal bleeding prior to catheter angiography. Although contrast-induced nephropathy and renal outcomes were discussed as secondary safety analyses, the primary objective focused on diagnostic imaging correlation and bleeding localization rather than contrast-associated acute kidney injury following interventional radiology procedures according to the predefined eligibility criteria for this review. |
| Staniloae CS, Doucet S, Sharma SK, et al. N-Acetylcysteine Added to Volume Expansion with Sodium Bicarbonate Does Not Further Prevent Contrast-Induced Nephropathy: Results from the Cardiac Angiography in Renally Impaired Patients Study. J Interv Cardiol. 2009;22:261–265. https://doi.org/10.1111/j.1540-8183.2009.00456.x | Wrong procedure specialty/population. This multicenter study evaluated prevention of contrast-induced nephropathy in patients undergoing cardiac angiography and coronary interventions. The study population was limited to interventional cardiology procedures rather than interventional radiology or peripheral/neurovascular endovascular procedures according to the predefined eligibility |
| Sun H, Qi L, Wang G, et al. A Comparative Study of Stepping Angiography and Traditional Segmental Angiography in Lower Limb Anterograde Venography. J Int Med Res. 2025;53. https://doi.org/10.1177/03000605251361099 | Wrong primary study focus/outcome framework. This retrospective study primarily compared imaging quality, radiation exposure, angiography duration, and contrast dosage between stepping angiography and traditional segmental angiography during lower limb venography. Although contrast-induced nephropathy was briefly discussed as a theoretical consideration, the study did not directly evaluate contrast-associated acute kidney injury or renal outcomes as predefined study endpoints according to the eligibility criteria for this review. |
| Sunman H, Arıcı M, Asil S, et al. Incidence of Persistent Renal Dysfunction and Long-Term Survival after Coronary Computed Tomography Angiography. Cardiovasc J Afr. 2025;36:244–248. http://doi.org/10.5830/CVJA-2025-032 | Wrong procedure specialty/population. This retrospective study evaluated contrast-induced nephropathy and persistent renal dysfunction following coronary computed tomography angiography (CTA), which is a noninvasive cardiac imaging procedure rather than an interventional radiology or therapeutic endovascular procedure according to the predefined eligibility criteria for this review. |
| Szczurowska A, Banasik M, Kurcz J, et al. Intra-arterial computed tomography angiography with ultra-low volume of iodine contrast and stent implantation in transplant renal artery stenosis in terms of contrast-induced kidney injury – a preliminary report. Pol J Radiol. 2020;85:e174–e177. https://doi.org/10.5114/pjr.2020.94364 | Wrong procedure specialty/population. This preliminary study evaluated ultra-low-volume intra-arterial computed tomography angiography and stent implantation for transplant renal artery stenosis in renal transplant recipients. The study population primarily represented transplant renovascular intervention practice rather than predefined interventional radiology procedural cohorts according to the eligibility criteria for this review. |
| Taha AG, Saleh M, Ali H. Safety and Efficacy of Hybrid Angiography in Chronic Lower Extremity Ischemia Patients at Risk of Contrast-Induced Acute Kidney Injury. Vasc Endovascular Surg. 2022;56:180–189. https://doi.org/10.1177/15385744211055908 | Wrong procedure specialty/population. This prospective study evaluated hybrid carbon dioxide-iodinated contrast angiography during lower extremity endovascular interventions for chronic limb-threatening ischemia in patients with chronic kidney disease. The study population primarily represented peripheral vascular intervention practice rather than predefined interventional radiology procedural cohorts according to the eligibility criteria for this review. |
| Takahashi EA, Kallmes DF, Mara KC, et al. Nephrotoxicity of Gadolinium-Based Contrast in the Setting of Renal Artery Intervention: Retrospective Analysis with 10-Year Follow-Up. Diagn Interv Radiol. 2018;24:378–384. https://doi.org/10.5152/dir.2018.18172 | Wrong contrast agent exposure. This retrospective study evaluated postcontrast acute kidney injury following renal artery stent placement using gadolinium-based contrast agents rather than iodinated contrast media. The study therefore did not meet the predefined eligibility criteria restricting inclusion to iodinated contrast-associated acute kidney injury during interventional radiology and endovascular procedures. |
| Takahashi EA, Kallmes DF, Fleming CJ, et al. Predictors and Outcomes of Post-Contrast Acute Kidney Injury After Endovascular Renal Artery Intervention. J Vasc Interv Radiol. 2017;28:1687–1692. https://doi.org/10.1016/j.jvir.2017.07.038 | Wrong procedure specialty/population. This retrospective study evaluated post-contrast acute kidney injury following renal artery stent placement for atherosclerotic renal artery stenosis. The study population primarily represented renovascular intervention practice rather than predefined interventional radiology procedural cohorts according to the eligibility criteria for this review. |
| Takata T, Yamada K, Yamamoto M, et al. Generative Deep-Learning-Model Based Contrast Enhancement for Digital Subtraction Angiography Using a Text-Conditioned Image-to-Image Model. Comput Biol Med. 2025;195:110598. https://doi.org/10.1016/j.compbiomed.2025.110598 | Wrong study focus and ineligible outcome framework. This study evaluated an artificial intelligence–based image enhancement technique for digital subtraction angiography and focused on image processing performance metrics such as RMS contrast, Michelson contrast, signal-to-noise ratio, and entropy. Although contrast-induced nephropathy and contrast reduction strategies were discussed in the background, the study did not directly evaluate iodinated contrast-associated acute kidney injury, renal outcomes, or patient-level clinical outcomes following interventional radiology procedures according to the predefined eligibility criteria for this review. |
| Takeuchi Y, Morikage N, Matsuno Y, et al. Midterm Outcomes of Endovascular Aortic Aneurysm Repair with Carbon Dioxide-Guided Angiography. Ann Vasc Surg. 2018;51:170–176. https://doi.org/10.1016/j.avsg.2018.02.036 | Wrong procedure specialty/population. This retrospective study evaluated carbon dioxide-guided endovascular aortic aneurysm repair (EVAR) and renal outcomes in patients with severe renal dysfunction or iodinated contrast allergy. The study population primarily represented vascular surgery/endovascular aortic repair practice rather than predefined interventional radiology procedural cohorts according to the eligibility criteria for this review. |
| Tanhui‐Manzana KK, Crisostomo AC, Bandong IS, et al. Development and Internal Validation of a Prediction Model for Intravenous Contrast‐Associated Acute Kidney Injury. J Hosp Med. 2026;21:142–151. https://doi.org/10.1002/jhm.70136 | Wrong procedure population and imaging modality. This retrospective cohort study evaluated prediction modeling for contrast‐associated acute kidney injury following intravenous contrast‐enhanced computed tomography imaging across mixed inpatient and outpatient populations. The study did not specifically evaluate interventional radiology or therapeutic endovascular procedures according to the predefined eligibility criteria for this review. |
| Tao SM, Kong X, Schoepf UJ, et al. Acute Kidney Injury in Patients with Nephrotic Syndrome Undergoing Contrast-Enhanced CT for Suspected Venous Thromboembolism: A Propensity Score-Matched Retrospective Cohort Study. Eur Radiol. 2018;28:1585–1593. https://doi.org/10.1007/s00330-017-5109-0 | Wrong imaging modality and procedure population. This retrospective propensity score-matched study evaluated acute kidney injury following intravenous contrast-enhanced computed tomography examinations, including CT pulmonary angiography and renal CT venography, in patients with nephrotic syndrome. The study did not involve interventional radiology or therapeutic endovascular procedures according to the predefined eligibility criteria for this review. |
| Taramasso M, Latib A, Denti P, et al. Acute Kidney Injury Following MitraClip Implantation in High Risk Patients: Incidence, Predictive Factors and Prognostic Value. Int J Cardiol. 2013;169:e24–e25. https://doi.org/10.1016/j.ijcard.2013.08.104 | Taramasso M, Latib A, Denti P, et al. Acute Kidney Injury Following MitraClip Implantation in High Risk Patients: Incidence, Predictive Factors and Prognostic Value. Int J Cardiol. 2013;169:e24–e25. https://doi.org/10.1016/j.ijcard.2013.08.104 |
| Tasanarong A, Hutayanon P, Piyayotai D. Urinary Neutrophil Gelatinase-Associated Lipocalin Predicts the Severity of Contrast-Induced Acute Kidney Injury in Chronic Kidney Disease Patients Undergoing Elective Coronary Procedures. BMC Nephrol. 2013;14:270. https://doi.org/10.1186/1471-2369-14-270 | Wrong procedure specialty/population. This prospective study evaluated urinary NGAL as a biomarker for contrast-induced acute kidney injury in chronic kidney disease patients undergoing elective coronary angiography and coronary interventions. The study population belonged to interventional cardiology rather than interventional radiology or non-cardiac endovascular procedures according to the predefined eligibility criteria for this review. |
| Tenorio ER, Balachandran PW, Marcondes GB, et al. Incidence, predictive factors, and outcomes of intraprocedure adverse events during fenestrated-branched endovascular aortic repair of complex abdominal and thoracoabdominal aortic aneurysms. J Vasc Surg. 2022;75:783–793. https://doi.org/10.1016/j.jvs.2021.10.026 | Wrong procedure specialty/population. This retrospective cohort study evaluated intraoperative adverse events and major postoperative complications, including acute kidney injury, following fenestrated-branched endovascular aortic repair (FB-EVAR) for complex abdominal and thoracoabdominal aortic aneurysms. The study population primarily represented complex vascular surgery/endovascular aortic repair practice rather than predefined interventional radiology procedural cohorts according to the eligibility criteria for this review. |
| Tenorio ER, Kärkkäinen JM, Marcondes GB, et al. Impact of intentional accessory renal artery coverage on renal outcomes after fenestrated-branched endovascular aortic repair. J Vasc Surg. 2021;73:805–818. https://doi.org/10.1016/j.jvs.2020.06.123 | Wrong procedure specialty/population. This prospective cohort study evaluated renal outcomes, including acute kidney injury and renal function deterioration, following fenestrated-branched endovascular aortic repair (FB-EVAR) with intentional accessory renal artery coverage for pararenal and thoracoabdominal aortic aneurysms. The study population primarily represented complex vascular surgery/endovascular aortic repair practice rather than predefined interventional radiology procedural cohorts according to the eligibility criteria for this review. |
| Tenorio ER, Oderich GS, Kölbel T, et al. Outcomes of off-the-shelf multibranched stent grafts with intentional occlusion of directional branches using endovascular plugs during endovascular repair of complex aortic aneurysms. J Vasc Surg. 2022;75:1142–1150. https://doi.org/10.1016/j.jvs.2021.09.050 | Wrong procedure specialty/population. This multicenter cohort study evaluated outcomes of branched endovascular aortic repair (BEVAR) using off-the-shelf multibranched thoracoabdominal stent grafts with intentional directional branch occlusion for complex thoracoabdominal aortic aneurysms. The study population primarily represented complex vascular surgery/endovascular aortic repair practice rather than predefined interventional radiology procedural cohorts according to the eligibility criteria for this review. |
| Tinelli G, Sica S, Tsilimparis N, et al. Open surgery versus branched endovascular repair of the aortic arch in residual dissections after type A surgical repair. Br J Surg. 2026;113:znaf300. https://doi.org/10.1093/bjs/znaf300 | Wrong procedure specialty/population. This multicenter study evaluated outcomes following redo open arch repair and branched endovascular aortic repair for residual chronic aortic dissection after prior type A aortic dissection surgery. Although acute kidney injury was reported as a perioperative complication, the study primarily represented cardiovascular/aortic surgical and hybrid endovascular repair populations rather than predefined interventional radiology procedural cohorts according to the eligibility criteria for this review. |
| Traub SJ, Mitchell AM, Jones AE, et al. N-Acetylcysteine Plus Intravenous Fluids Versus Intravenous Fluids Alone to Prevent Contrast-Induced Nephropathy in Emergency Computed Tomography. Ann Emerg Med. 2013;62:511–520. https://doi.org/10.1016/j.annemergmed.2013.04.012 | Wrong imaging modality and procedure population. This randomized controlled trial evaluated prevention of contrast-induced nephropathy following intravenous contrast-enhanced computed tomography of the chest, abdomen, and pelvis in emergency department patients. The study involved diagnostic intravenous CT imaging rather than interventional radiology or therapeutic endovascular procedures according to the predefined eligibility criteria for this review. |
| Turan B, Erkol A, Gül M, et al. Effect of Contrast-Induced Nephropathy on the Long-Term Outcome of Patients with Non-ST Segment Elevation Myocardial Infarction. Cardiorenal Med. 2015;5:116–124. https://doi.org/10.1159/000371900 | Wrong procedure specialty/population. This retrospective study evaluated contrast-induced nephropathy following coronary angiography and percutaneous coronary intervention in patients with non-ST segment elevation myocardial infarction. The study population belonged to interventional cardiology rather than interventional radiology or non-cardiac endovascular procedures according to the predefined eligibility criteria for this review. |
| Turedi S, Erdem E, Karaca Y, et al. The High Risk of Contrast-Induced Nephropathy in Patients with Suspected Pulmonary Embolism Despite Three Different Prophylaxis: A Randomized Controlled Trial. Acad Emerg Med. 2016;23:1136–1145. https://doi.org/10.1111/acem.13051 | Wrong imaging modality and procedure population. This randomized controlled trial evaluated contrast-induced nephropathy prevention strategies following contrast-enhanced computed tomography pulmonary angiography in emergency department patients with suspected pulmonary embolism. The study involved diagnostic intravenous CT imaging rather than interventional radiology or therapeutic endovascular procedures according to the predefined eligibility criteria for this review. |
| Ueda H, Yamada T, Masuda M, et al. Prevention of Contrast-Induced Nephropathy by Bolus Injection of Sodium Bicarbonate in Patients With Chronic Kidney Disease Undergoing Emergent Coronary Procedures. Am J Cardiol. 2011;107:1163–1167. https://doi.org/10.1016/j.amjcard.2010.12.012 | Wrong procedure specialty/population. This prospective randomized controlled trial evaluated prevention of contrast-induced nephropathy in patients with chronic kidney disease undergoing emergent coronary angiography and percutaneous coronary intervention for suspected acute coronary syndrome. The study population belonged to interventional cardiology rather than interventional radiology or non-cardiac endovascular procedures according to the predefined eligibility criteria for this review. |
| Unal EU, Iscan HZ, Erol ME, et al. Carbon dioxide guided endovascular aortic aneurysm repair in impaired renal function: propensity score matched study. Eur J Vasc Endovasc Surg. 2023;66:521–529. https://doi.org/10.1016/j.ejvs.2023.06.039 | Wrong procedure specialty/population. This propensity score matched study evaluated contrast-induced nephropathy following carbon dioxide-guided endovascular aneurysm repair (EVAR) for abdominal aortic aneurysms in patients with impaired renal function. The study population primarily represented vascular surgical/aortic endovascular repair procedures rather than predefined interventional radiology procedural cohorts according to the eligibility criteria for this review. |
| Uyanik SA, Birgi E, Ozturk S, et al. The role of carbon dioxide angiography in reducing contrast-induced nephropathy in diabetic foot patients undergoing endovascular treatment. Turk J Med Sci. 2025;55:877–886. https://doi.org/10.55730/1300-0144.6040 | Wrong procedure specialty/population. This retrospective cohort study evaluated carbon dioxide angiography and contrast-induced nephropathy during infrainguinal endovascular interventions for diabetic foot patients with peripheral arterial disease. The study population primarily represented peripheral vascular intervention/chronic limb-threatening ischemia practice rather than predefined interventional radiology procedural cohorts according to the eligibility criteria for this review. |
| van der Molen AJ, Dekkers IA, Bedioune I, et al. A Systematic Review of the Incidence of Hypersensitivity Reactions and Post-Contrast Acute Kidney Injury After Ioversol: Part 2—Intra-Arterial Administration. Eur Radiol. 2022. https://doi.org/10.1007/s00330-022-08637-2 | Ineligible study design. This systematic review evaluated hypersensitivity reactions and post-contrast acute kidney injury after intra-arterial administration of ioversol across heterogeneous angiographic and interventional populations, including coronary, cerebral, and peripheral procedures. As a review article without original patient-level data, it did not meet the predefined eligibility criteria restricting inclusion to primary clinical studies of iodinated contrast-associated acute kidney injury following interventional radiology or endovascular procedures. |
| Wagner G, Glechner A, Persad E, et al. Risk of Contrast-Associated Acute Kidney Injury in Patients Undergoing Peripheral Angiography with Carbon Dioxide Compared to Iodine-Containing Contrast Agents: A Systematic Review and Meta-Analysis. J Clin Med. 2022;11:7203. https://doi.org/10.3390/jcm11237203 | Ineligible study design. This systematic review and meta-analysis evaluated the risk of contrast-associated acute kidney injury following peripheral angiography and endovascular interventions using carbon dioxide versus iodinated contrast agents across multiple heterogeneous studies. As a secondary evidence synthesis without original patient-level data, it did not meet the predefined eligibility criteria restricting inclusion to primary clinical studies of iodinated contrast-associated acute kidney injury following interventional radiology or endovascular procedures. |
| Wang ZY, Wang YL, Wei J, et al. Role of serum cystatin C in the prediction of contrast-induced nephropathy after intra-arterial interventions. Chin Med J. 2020;133:408–414. https://doi.org/10.1097/CM9.0000000000000641 | Mixed heterogeneous procedure population. This prospective observational study evaluated serum cystatin C as a biomarker for contrast-induced nephropathy across heterogeneous intra-arterial interventions, including cerebrovascular, peripheral vascular, bronchial artery, and oncologic procedures. The study did not provide procedure-specific interventional radiology cohorts according to the predefined eligibility criteria for this review. |
| Weisz G, Filby SJ, Cohen MG, et al. Safety and Performance of Targeted Renal Therapy: The Be-RITe! Registry. J Endovasc Ther. 2009;16:1–12. http://doi.org/10.1583/08-2515.1 | Wrong procedure specialty/population and mixed non-IR cohort. This retrospective registry primarily evaluated targeted renal therapy using the Benephit system for prevention of contrast-induced nephropathy in patients undergoing coronary angiography, percutaneous coronary intervention, cardiovascular surgery, and mixed peripheral procedures. The majority of patients underwent coronary procedures, and the study focused on intrarenal fenoldopam infusion therapy rather than a defined interventional radiology or non-cardiac endovascular cohort according to the predefined eligibility criteria for this review. |
| Wittig T, Fischer S, Winther B, et al. Acute kidney injury after peripheral interventions using carbon dioxide angiography—Risk factors beyond iodinated contrast media. Life. 2025;15:1046. https://doi.org/10.3390/life15071046 | Wrong procedure specialty/population. This retrospective cohort study evaluated acute kidney injury following carbon dioxide-guided peripheral angiography and endovascular interventions in patients with peripheral artery disease and chronic kidney disease. The study population primarily represented peripheral vascular intervention and angiology/endovascular practice rather than predefined interventional radiology procedural cohorts according to the eligibility criteria for this review. |
| Woltemath AA, Gonzalez G, Tecson KM, et al. Contrast-associated acute kidney injury following peripheral angiography. Proc (Bayl Univ Med Cent). 2021;34:678–680. https://doi.org/10.1080/08998280.2021.1941701 | Wrong procedure specialty/population. This retrospective cohort study evaluated contrast-associated acute kidney injury following peripheral angiographic procedures. The study population primarily represented peripheral vascular intervention/angiography practice rather than predefined interventional radiology procedural cohorts according to the eligibility criteria for this review. |
| Xiang Y, Liu Y, Zhao J, et al. Use of angiotensin-converting enzyme inhibitors or angiotensin receptor blockers increases the risk of postoperative acute kidney injury after elective endovascular abdominal aortic aneurysm repair. Chin Med J. 2022;135:2836–2842. https://doi.org/10.1097/CM9.0000000000002352 | Wrong procedure specialty/population. This retrospective cohort study evaluated postoperative acute kidney injury and medication-related risk factors following elective endovascular abdominal aortic aneurysm repair (EVAR). The study population primarily represented vascular surgery/endovascular aortic repair practice rather than predefined interventional radiology procedural cohorts according to the eligibility criteria for this review. |
| Xiong HL, Peng M, Jiang XJ, et al. Nephrotoxicity of iodixanol versus iopamidol in patients undergoing peripheral angiography with or without endovascular therapy. Int Urol Nephrol. 2018;50:1879–1886. https://doi.org/10.1007/s11255-018-1905-y | Mixed heterogeneous procedure population. This prospective randomized study evaluated contrast-associated acute kidney injury and nephrotoxicity across heterogeneous peripheral angiographic and endovascular procedures, including carotid, vertebral, renal, iliac, and femoral artery interventions. The study did not provide procedure-specific interventional radiology cohorts according to the predefined eligibility criteria for this review. |
| Yamamoto R, Cestero RF, Yoshizawa J, et al. Emergency angiography for trauma patients and potential association with acute kidney injury. World J Emerg Surg. 2021;16:56. https://doi.org/10.1186/s13017-021-00400-0 | Wrong procedure specialty/population. This nationwide retrospective cohort study evaluated acute kidney injury following emergency angiography in trauma patients undergoing mixed diagnostic and therapeutic angiographic procedures, including embolization. The study population primarily represented emergency trauma angiography and acute care procedural cohorts rather than predefined interventional radiology procedural populations according to the eligibility criteria for this review. |
| Yang G, Zhang Y, Ni H, et al. Result of a multicenter study on the physician-modified fenestrated and branched endovascular grafts for thoracoabdominal aneurysms. Ann Thorac Surg. 2025;119:1241–1250. https://doi.org/10.1016/j.athoracsur.2025.01.025 | Wrong procedure specialty/population. This multicenter retrospective study evaluated physician-modified fenestrated and branched endovascular grafts (PMEGs) for thoracoabdominal aortic aneurysm repair, including perioperative complications such as acute kidney injury. The study population primarily represented complex vascular surgery/endovascular aortic repair practice rather than predefined interventional radiology procedural cohorts according to the eligibility criteria for this review. |
| Ye CC, Kumolosasi E, Wee CE. N-Acetylcysteine for Prevention of Iodinated Contrast-Induced Nephropathy in Computed Tomography Angiography Procedures in Patients with Chronic Kidney Disease. Afr J Pharm Pharmacol. 2012;6:181–189. https://doi.org/10.5897/AJPP11.619 | Wrong imaging modality and procedure population. This retrospective observational study evaluated contrast-induced nephropathy prevention strategies in patients undergoing computed tomography angiography (CTA) procedures using iodinated contrast media. The study involved diagnostic CT/CTA imaging rather than interventional radiology or therapeutic endovascular procedures according to the predefined eligibility criteria for this review. |
| Yildirim E, Ermis E, Cengiz M. Inflammatory Markers of Contrast-Induced Nephropathy in Patients with Acute Coronary Syndrome. Coron Artery Dis. 2019. https://doi.org/10.1097/MCA.0000000000000823 | Wrong procedure specialty/population. This observational study evaluated inflammatory predictors of contrast-induced nephropathy in patients with acute coronary syndrome undergoing emergency coronary angiography and percutaneous coronary intervention. The study population belonged to interventional cardiology rather than interventional radiology or non-cardiac endovascular procedures according to the predefined eligibility criteria for this review. |
| Yun D, Kim DK, Lee JP, et al. Can Sodium Fluorescein Cause Contrast-Induced Nephropathy? Nephrol Dial Transplant. 2021;36:819–825. https://doi.org/10.1093/ndt/gfz243 | Yun D, Kim DK, Lee JP, et al. Can Sodium Fluorescein Cause Contrast-Induced Nephropathy? Nephrol Dial Transplant. 2021;36:819–825. https://doi.org/10.1093/ndt/gfz243 . |
| Zang J, Liang J, Zhang X, et al. Short Term Sodium Glucose Transport Protein 2 Inhibitors are Associated with Post Contrast Acute Kidney Injury in Patients with Diabetes. Sci Rep. 2024;14:22937. https://doi.org/10.1038/s41598-024-74233-7 | Wrong procedure specialty/population. This prospective cohort study evaluated post-contrast acute kidney injury in patients with type 2 diabetes mellitus undergoing coronary arterial angiography with or without percutaneous coronary intervention. The study population belonged primarily to interventional cardiology rather than interventional radiology or non-cardiac endovascular procedures according to the predefined eligibility criteria for this review. |
| Zevallos CB, Dai B, Dandapat S, et al. Greater Intraprocedural Systolic Blood Pressure and Blood Pressure Variability are Associated with Contrast-Induced Neurotoxicity after Neurointerventional Procedures. J Neurol Sci. 2021;420:117209. https://doi.org/10.1016/j.jns.2020.117209 | Wrong outcome of interest. This matched case-control study evaluated contrast-induced neurotoxicity and blood pressure variability following neurointerventional procedures and cerebral angiography. Although the procedures involved neuroendovascular interventions and iodinated contrast exposure, the primary outcome was neurologic toxicity rather than contrast-associated acute kidney injury or renal outcomes according to the predefined eligibility criteria for this review. |
| Zhang J, Li Y, Tao GZ, et al. Short-term Rosuvastatin Treatment for the Prevention of Contrast-induced Acute Kidney Injury in Patients Receiving Moderate or High Volumes of Contrast Media: A Sub-analysis of the TRACK-D Study. Chin Med J. 2015;128:784–789. http://doi.org/10.4103/0366-6999.152620 | Wrong procedure specialty/population and mixed non-IR cohort. This randomized sub-analysis evaluated contrast-induced acute kidney injury prevention in patients with diabetes and chronic kidney disease undergoing predominantly coronary angiography and percutaneous coronary intervention, with some peripheral arterial angiography procedures included. The study population was primarily interventional cardiology-based rather than a defined interventional radiology or non-cardiac endovascular cohort according to the predefined eligibility criteria for this review. |
| Zhao J, Cheng Q, Zhang X, et al. Efficacy of percutaneous transluminal renal angioplasty with stent in elderly male patients with atherosclerotic renal artery stenosis. Clin Interv Aging. 2012;7:417–422. https://doi.org/10.2147/CIA.S36925 | Wrong procedure specialty/population. This retrospective cohort study evaluated percutaneous transluminal renal angioplasty with stent implantation (PTRAS) for atherosclerotic renal artery stenosis, including procedural complications such as contrast-induced nephropathy. The study population primarily represented renovascular intervention practice rather than predefined interventional radiology procedural cohorts according to the eligibility criteria for this review. |
| Zhou Y, Wang J, Zhao J, et al. Randomised Controlled Trial to Evaluate the Effect of Contrast Material Dilution on Renal Function in Patients after Endovascular Aortic Repair: Rationale and Design for the CULTURE Trial. BMJ Open. 2023;13:e069804. https://doi.org/10.1136/bmjopen-2022-069804 | Ineligible study design. This article describes the rationale and protocol design of the CULTURE randomized controlled trial investigating contrast dilution strategies during thoracic and abdominal endovascular aortic repair. As a study protocol without completed patient outcomes or original clinical results, it did not meet the predefined eligibility criteria restricting inclusion to completed primary clinical studies reporting contrast-associated acute kidney injury or renal outcomes after interventional radiology or endovascular procedures. |

Supplementary Table S3. Characteristics of included studies evaluating contrast-associated acute kidney injury following interventional radiology procedures.

| Study (Author, Year) | Country | Study Design | Analytic Sample Size / Unit of Analysis | Population | Procedure Type | Procedure Domain | Contrast Administration Route | Contrast-Media Type/Agent | Contrast Volume (mL) | Baseline CKD Prevalence (%) | Definition of CA-AKI | Follow-up |
| --- | --- | --- | --- | --- | --- | --- | --- | --- | --- | --- | --- | --- |
| Aoe (2019) [43] | Japan | Single-center retrospective cohort study | 461 TACE sessions in 260 patients | Patients with HCC undergoing TACE | TACE | Embolization/interventional oncology | NR | Low-osmolality iodinated contrast media (iopamidol/Iopamiron) | Mean 77.8 ± 34.9 mL | 27.3% | CIN defined as increase in serum creatinine ≥0.5 mg/dL or ≥25% from baseline within 72 h after TACE | Serum creatinine assessed within 24–72 h after TACE |
| Cao (2021) [42] | China | Single-center retrospective cohort study | 103 patients | Patients with HCC and CKD undergoing TACE | TACE | Embolization/interventional oncology | NR | Ioversol (reported as a “hypotonic” iodinated contrast agent) | Mean contrast agent dose 65.9 ± 11.34 mL | 100% | CIN defined as increase in serum creatinine by ≥0.5 mg/dL within 48–72 h after contrast administration | Serum creatinine reassessed within 48–72 h following TACE |
| Cho (2011) [41] | South Korea | Retrospective cohort study | 221 TACE treatment sessions in 101 patients | Patients with HCC undergoing TACE | TACE | Embolization/interventional oncology | IA | Nonionic low-osmolality iodinated contrast agent | Mean 258.9 ± 64.6 mL | 4.5%^a^ | RCIN defined as ≥25% increase in serum creatinine from baseline between 48 and 96 h after TACE | Serum creatinine assessed within 48–96 h after TACE |
| Cohen (2014) [40] | International multicenter cohort (Israel, Spain, Italy, Hong Kong/China) | International retrospective cohort study/database analysis | 548 patients | Elderly and non-elderly patients with advanced HCC undergoing TACE | TACE | Embolization/interventional oncology | NR | Lipiodol-based chemoembolization using iodinated contrast agents; protocols varied by center^b^ | Variable according to center-specific TACE protocols | NR | AKI reported as post-TACE adverse event; specific diagnostic criteria not reported | Median follow-up 69 months; survival follow-up until death or censorship |
| Delgado Acosta (2020) [39] | Spain | Single-center retrospective cohort study | 189 patients | Patients with AIS undergoing neuroendovascular treatment | EVT for AIS | Neuroendovascular/carotid procedures | Mixed IV + IA | Iohexol 300 for CT/CTA/CTP and non-ionic isosmolar iodixanol (Visipaque 270) for angiography | Mean iohexol volume 97.1 ± 30.1 mL; mean estimated iodixanol angiographic volume 140 ± 74.1 mL | 2.1%^c^ | CIN defined as increase in serum creatinine >25% from baseline or absolute increase ≥0.50 mg/dL after intravascular contrast administration | Serum creatinine assessed during hospitalisation, typically within 48–72 h after contrast exposure |
| Diprose (2019) [38] | New Zealand | Single-center prospective registry cohort study | 336 patients | Patients with AIS undergoing EVT | EVT | Neuroendovascular/carotid procedures | IA | Iohexol (240 or 300 mg/mL) | Mean total iohexol contrast volume 236 ± 77 mL per patient | 27.7%^d^ | CA-AKI defined as increase in serum creatinine ≥26.5 µmol/L or ≥1.5× baseline serum creatinine within 24–72 h following contrast exposure | Serum creatinine assessed 24–72 h after EVT |
| Hao (2014) [37] | China | Single-center prospective cohort study | 166 patients undergoing 316 TACE procedures | Patients with HCC undergoing TACE | TACE | Embolization/interventional oncology | NR | Iohexol and iodixanol (Visipaque) iodinated contrast agents | NR | NR | AKI defined using both Barrett and Parfrey criteria and AKIN criteria | Serum creatinine assessed within 72 h after TACE |
| Hayakawa (2014) [36] | Japan | Single-center retrospective cohort study | 305 TACE sessions in 141 patients | Patients with HCC undergoing TACE | TACE | Embolization/interventional oncology | NR | Nonionic iodinated contrast media: iopamidol 370 mgI/mL and iohexol 350 mgI/mL | Mean 111 ± 29 mL | 15.4%^e^ | CIN defined as increase in serum creatinine >0.5 mg/dL or >25% from baseline within 3 days after TACE without alternative cause of AKI | Serum creatinine assessed on postoperative day 2 or 3 and followed until normalization if serum creatinine increased |
| Hayashi (2020) [35] | Japan | Single-center retrospective cohort study | 270 TACE/TAI sessions in 129 patients | Patients with unresectable HCC undergoing platinum-based TACE/TAI | Platinum-based TACE and TAI | Embolization/interventional oncology | NR | Iopamidol 370/300 mgI/mL or iomeprol 350/300 mgI/mL iodinated contrast agents | Median 75.5 mL (IQR 64.3–87.9) | 29.0%^f^ | AKI diagnosed according to ICA cirrhosis-specific AKI criteria as an increase in serum creatinine ≥0.3 mg/dL or ≥50% above baseline within 7 days after TACE/TAI | Serum creatinine assessed pre-treatment and on days 1, 5, and 30 after TACE/TAI |
| Hsu (2010) [34] | Taiwan | Single-center prospective cohort study | 591 HCC patients undergoing first TACE session, including 91 patients with pre-existing ascites | Patients with unresectable HCC undergoing TACE | TACE | Embolization/interventional oncology | IA | Telebrix or Ultravist iodinated contrast agents | 100–150 mL | NR^g^ | ARF defined as an increase in serum creatinine ≥50% from baseline or absolute increase ≥0.5 mg/dL to a level >1.5 mg/dL within 7 days after TACE | Mean follow-up duration 19 ± 17 months |
| Huo (2004) [33] | Taiwan | Single-center prospective cohort study | 140 consecutive patients undergoing TACE | Patients with unresectable HCC undergoing TACE | TACE | Embolization/interventional oncology | IA | Telebrix or Ultravist iodinated contrast agents | 100–150 mL | Patients with severe baseline renal dysfunction were excluded | ARF defined as an increase in serum creatinine ≥50% from baseline or absolute increase ≥0.5 mg/dL to >1.5 mg/dL within 7 days after TACE | Serum creatinine assessed before TACE and on days 3, 6, and 9 after TACE |
| Huo (2004) [32] | Taiwan | Single-center retrospective cohort study | 235 patients undergoing 843 TACE sessions | Patients with unresectable HCC undergoing TACE | TACE | Embolization/interventional oncology | IA | Telebrix or Ultravist iodinated contrast agents | 100–150 mL | Patients with severe baseline renal dysfunction were excluded | ARF defined as increase in serum creatinine ≥50% from baseline or absolute increase ≥0.5 mg/dL to >1.5 mg/dL within 7 days after TACE | Mean follow-up duration 33 ± 18 months |
| Jia (2019) [31] | China | Single-center prospective cohort study | 181 patients with AIS | Patients with AIS undergoing CTA alone or CTA followed by DSA/endovascular treatment | CTA and DSA-guided neuroendovascular intervention | Neuroendovascular/carotid procedures | Mixed IV + IA | Isotonic iodinated contrast medium (iodixanol, Visipaque 320 mg I/mL) | CTA: mean 95.0 ± 6.9 mL; CTA+DSA: total mean 187.0 ± 15.7 mL | Patients with severe baseline renal dysfunction were excluded | AKI defined as ≥25% increase in serum creatinine from baseline 48 h after CTA | Serum creatinine assessed at baseline and 48 h after CTA |
| Joo (2019) [30] | Republic of Korea | Retrospective two-center cohort study | 230 patients with unruptured cerebral aneurysms undergoing elective coiling | Patients with unruptured cerebral aneurysms undergoing neuroendovascular coiling under general anaesthesia | Cerebral aneurysm coiling/endovascular embolization | Neuroendovascular/carotid procedures | IA | Nonionic low-osmolar and iso-osmolar iodinated contrast agents: iohexol (Iobrix), iopromide (Ultravist), ioversol (Optiray), and iodixanol-320 (Visipaque) | Mean cumulative contrast dose: 206.4 ± 57.2 mL (non-CIN group); 226.9 ± 59.9 mL (CIN group) | 0.9%^h^ | CIN defined as absolute increase in serum creatinine ≥0.5 mg/dL or relative increase ≥25% from baseline serum creatinine value within 48–72 h after contrast exposure | Serum creatinine assessed within 48–72 h after contrast exposure |
| Kim (2018) [29] | United States | Retrospective two-center cohort study | 184 patients with CKD undergoing neuroendovascular procedures | High-risk patients with CKD (eGFR <60 mL/min/1.73 m²) undergoing diagnostic cerebral angiography or neuroendovascular intervention | Diagnostic cerebral angiography and neuroendovascular interventions | Neuroendovascular/carotid procedures | IA | Iso-osmolar nonionic iodinated contrast medium: iodixanol (Visipaque-320) | Diagnostic procedures: mean 51.9 ± 24.1 mL; therapeutic interventions: mean 129.4 ± 28.2 mL | 100%^i^ | CIN defined as absolute increase in serum creatinine ≥0.5 mg/dL or ≥25% rise from baseline within 48–72 h after contrast exposure after excluding other causes of renal impairment | Serum creatinine and eGFR assessed at baseline and 48–72 h post-procedure |
| Kim (2020) [28] | Republic of Korea | Single-center prospective cohort study | 176 patients undergoing elective neurointervention procedures | Patients with cerebrovascular disease undergoing elective diagnostic cerebral angiography or neuroendovascular procedures | Diagnostic cerebral angiography, carotid artery stenting, intracranial artery stenting, and coil embolization of ruptured/unruptured aneurysms | Neuroendovascular/carotid procedures | IA | Iso-osmolar iodinated contrast medium: iodixanol (Visipaque 320) | Mean contrast volume: 109.7 ± 39.3 mL (non-CIN group) vs. 100.2 ± 32.4 mL (CIN group) | 0%^j^ | CIN defined as increase in serum creatinine >0.5 mg/dL or ≥25% from baseline within 48 h after contrast exposure in the absence of alternative causes | Serum creatinine assessed at baseline and 6, 24, and 48 h after contrast exposure; vascular-event follow-up for 3 years |
| Laible (2021) [27] | Germany | Single-center prospective cohort study | 1,169 patients | Adult patients with AIS undergoing EVT for acute large vessel occlusion | EVT after CTA for AIS | Neuroendovascular/carotid procedures | Mixed IV + IA | CTA: iobitridol (Xenetix 350); cerebral angiography: iopamidol (Solutrast 300) | Standardized CTA dose: 65 mL; approximate median total contrast volume per patient: 215 mL | 32.4% | PC-AKI defined as increase in serum creatinine ≥0.5 mg/dL or >25% above baseline within 48 h after admission creatinine measurement | Serum creatinine and eGFR assessed at baseline and within 48 h after EVT; clinical follow-up included in-hospital outcomes and 3-month mRS |
| Lee (2017) [26] | Taiwan | Nationwide retrospective population-based cohort study | 1,132 HCC patients undergoing cTACE matched to 1,132 HCC patients without cTACE | Patients with HCC undergoing cTACE | cTACE | Embolization/interventional oncology | NR | Water-soluble iodinated contrast medium used during cTACE (specific agents not reported) | NR | 0%^k^ | AKI identified using ICD-9-CM diagnostic code 584 during follow-up | Follow-up from index date until AKI occurrence, withdrawal from insurance program, or 31 March 2010; mean follow-up 2.88 ± 2.95 years in cTACE group |
| Lee (2018) [25] | Republic of Korea | Single-center retrospective cohort study | 192 patients | Patients with aSAH undergoing coil embolization | Endovascular coil embolization for ruptured intracranial aneurysms | Neuroendovascular/carotid procedures | IA | Non-ionic low-osmolar and iso-osmolar iodinated contrast agents (iopamidol, iohexol/Omnipaque 300, iodixanol) | Mean total contrast volume 269 ± 75 mL | 0.5%^l^ | CI-AKI defined as increase in serum creatinine >25% or >0.5 mg/dL from baseline within 72 h after coil embolization | One-year clinical follow-up; serial creatinine assessed within 72 h after embolization |
| Lin (2025) [24] | Taiwan | Single-center retrospective cohort study | 265 patients undergoing first TACE session | Patients with HCC undergoing first-session TACE, including patients with CKD | cTACE and DEMs-based TACE | Embolization/interventional oncology | IA | Nonionic water-soluble iodinated contrast medium: iopromide (Ultravist®-370) | Total injected contrast volume per TACE session ranged from 50–70 mL | 40.8%^m^ | Renal-function deterioration evaluated using serial serum creatinine and CG-GFR trajectory changes after TACE; CIN framework discussed, but no fixed creatinine-threshold diagnostic definition applied | Serial renal-function assessment from admission through discharge and first outpatient follow-up |
| Mitaka (2023) [23] | Japan | Single-center retrospective cohort study | 228 patients | Adult patients undergoing elective neuroendovascular surgery requiring ICU admission | Neuroendovascular surgery for cerebral aneurysm, CAS, DAVF, AVM, and carotid artery dissection | Neuroendovascular/carotid procedures | IA | Nonionic low-osmolar iodinated contrast medium: iomeprol (Iomeprol®) | Mean 182.4 ± 76.1 mL | 0.9%^n^ | Clinical CA-AKI defined according to KDIGO criteria: increase in serum creatinine ≥0.3 mg/dL within 48 h, ≥1.5× baseline creatinine, or urine output <0.5 mL/kg/h for 6 h; subclinical CA-AKI defined as urine NGAL/creatinine ratio ≥55.2 μg/g·Cr on POD 0 and/or POD 1 | Serum creatinine and eGFR assessed preoperatively and on POD 0, 1, and 3; urine NGAL assessed on POD 0 and POD 1 in subset of patients |
| Murakami (2016) [22] | Japan | Single-center retrospective cohort study | 186 TACE sessions in 122 patients | Patients with HCC undergoing TACE | TACE | Embolization/interventional oncology | IA | Nonionic low-osmolality iodinated contrast media: iopamidol (Iopamiron 300), iohexol (Omnipaque 300), or iomeprol (Iomeron 300) | Mean 148 ± 45 mL | 39.0% of TACE sessions | CIN defined as increase in serum creatinine ≥0.5 mg/dL or ≥25% from baseline between 48 and 72 h after TACE | Serum creatinine reassessed 48–72 h after TACE |
| Myung (2020) [20] | Republic of Korea | Single-center retrospective cohort study | 601 patients | Patients with AIS undergoing sequential CTA and cerebral angiography | CTA followed by cerebral angiography for ischemic stroke evaluation and endovascular treatment | Neuroendovascular/carotid procedures | Mixed IV + IA | CTA: isotonic iopamidol (Iopamiro 370); cerebral angiography: isotonic iodixanol (Visipaque 320) | CTA upper limit 100 mL; combined exposure approximately 200 mL over short interval | 18.6%^o^ | PC-AKI defined as absolute increase in serum creatinine ≥0.5 mg/dL or relative increase ≥25% from baseline within 72 h after contrast exposure | Serum creatinine assessed within 72 h after contrast exposure |
| Ösken (2021) [21] | Turkey | Single-center retrospective cohort study | 389 patients | Patients with 50–99% CAS undergoing carotid artery stenting | Carotid artery stenting | Neuroendovascular/carotid procedures | IA | Low-osmolality iodinated contrast medium: iohexol or iodixanol | Mean 117.1 ± 40.7 mL | Patients with eGFR <30 mL/min/1.73 m² or dialysis-dependent CKD excluded | CI-AKI defined as increase in serum creatinine ≥0.3 mg/dL or ≥50% from baseline within 48–72 h after contrast exposure according to AKIN criteria | Serum creatinine assessed within 48–72 h after carotid artery stenting |
| Ozdogru (2025) [19] | Turkey | Single-center retrospective cohort study | 281 patients | Patients with AIS undergoing EVT for large vessel occlusion | MT/EVT | Neuroendovascular/carotid procedures | IA | Nonionic low-osmolar iodinated contrast medium: iohexol (Omnipol® 350) | Mean contrast volume: 147 ± 24 mL (non-CI-AKI group) vs. 157 ± 22 mL (CI-AKI group) | 8.9% | CI-AKI defined as increase in serum creatinine ≥0.5 mg/dL or >25% within 48–72 h after EVT compared with baseline | Serum creatinine assessed at baseline and 48–72 h after EVT |
| Park (2008) [18] | Republic of Korea | Single-center retrospective cohort study | 442 TACE treatment sessions in 236 patients | Patients with unresectable HCC undergoing TACE | TACE | Embolization/interventional oncology | IA | Nonionic low-osmolar iodinated contrast medium: iomeprol (350 mg iodine/mL) | Contrast injected during superior mesenteric and hepatic arteriography; exact total volume NR | NR^p^ | AKI defined according to AKIN criteria as absolute increase in serum creatinine ≥0.3 mg/dL or ≥50% from baseline within 48 h after TACE | Serum creatinine assessed daily for at least 2 days after TACE; follow-up at 1 month after discharge and long-term survival follow-up |
| Prasad (2014) [17] | United States | Single-center retrospective cohort study | 79 neuroendovascular procedures in high-dose contrast material cohort; 79 procedures in control cohort | Patients undergoing neuroendovascular procedures with contrast material dose ≥250 mL and no pre-existing CKD | Neuroendovascular procedures including cervical/cerebral angiography and spinal angiography | Neuroendovascular/carotid procedures | IA | Nonionic iodinated contrast media: iohexol (Omnipaque 240 and Omnipaque 300) | High-dose cohort mean: 326 mL; control cohort mean: 172 mL | 0%^q^ | CIN defined as increase in serum creatinine ≥50% above baseline or absolute increase ≥0.3 mg/dL at 24 or 48 h after procedure | Serum creatinine assessed within 24 and 48 h after neuroendovascular procedure |
| Ray (2013) [16] | United States | Single-center retrospective cohort study using prospectively collected database | 75 patients with SAH undergoing at least one contrast-enhanced imaging study | Patients with spontaneous SAH admitted to neurocritical ICU | CTA, CTA/CTP, cerebral angiography, and endovascular vasospasm interventions | Neuroendovascular/carotid procedures | Mixed IV + IA | Iopamidol 300 for CTA and cerebral angiography; iopamidol 370 for CTP | Variable; 30–400 mL depending on procedure type | 9.3%^r^ | CIN defined according to KDIGO criteria as serum creatinine increase >1.5× baseline or >0.3 mg/dL above baseline, or urine output <0.5 mL/kg/h during one 6-h block | Serum creatinine followed up to 72 h after contrast study; 6-month clinical follow-up available for neurological outcomes |
| Schwarz (2026) [15] | Multinational cohort across 16 countries (Europe and United States) | Retrospective multicenter cohort study (CAN-REST study) | 6,638 patients from 73 EVT centers | Patients with AIS undergoing EVT | EVT for AIS, including patients undergoing diagnostic angiography with intended EVT | Neuroendovascular/carotid procedures | Mixed IV + IA | Iodinated contrast media administered during pre-EVT imaging and EVT procedures; specific contrast agents varied by center | Median pre-EVT contrast volume: 90 mL (IQR 70–100); median EVT contrast volume: 80 mL (IQR 50–120) | 39.2%^s^ | CA-AKI defined according to KDIGO criteria as increase in serum creatinine ≥0.3 mg/dL or ≥1.5× baseline within 48 h after contrast administration | Baseline and 48-h post-EVT creatinine required; 90-day mRS follow-up |
| Seki (2021) [14] | Japan | Single-center retrospective cohort study | 47 patients undergoing emergency TAE for uncontrollable postpartum haemorrhage | Patients with uncontrollable postpartum haemorrhage undergoing emergency TAE | Emergency TAE for postpartum haemorrhage | Embolization/interventional oncology | Mixed IV + IA | Low-osmolality nonionic iodinated contrast media for CT and iohexol during TAE | Mean CT contrast volume: 103.8 ± 15.0 mL; mean TAE contrast volume: 172.7 ± 70.8 mL; mean total contrast volume: 239.0 ± 79.8 mL | 2.1^t^ | PC-AKI defined according to ESUR Guidelines v10.0 as increase in serum creatinine >0.3 mg/dL or >1.5× baseline within 48–72 h after intravascular contrast administration | Serum creatinine and eGFR assessed before TAE, 48–72 h after TAE, and before discharge |
| Sharma (2013) [13] | United States | Retrospective single-center cohort study | 194 consecutive patients | Patients with AIS undergoing emergency endovascular treatment | EVT for AIS | Neuroendovascular/carotid procedures | IA | Non-ionic low-osmolar iodinated contrast agent: Optiray 320 | Approximately 150 mL during endovascular procedure | 16.0%^u^ | CIN defined according to AKIN criteria as relative increase in serum creatinine ≥50% above baseline or absolute increase ≥0.3 mg/dL within 48 h after procedure | Baseline and within 48 h postprocedure serum creatinine assessment |
| Si (2021) [12] | China | Single-center retrospective cohort study | 503 consecutive TACE sessions in 284 patients | Patients with HCC undergoing TACE without baseline renal insufficiency | TACE | Embolization/interventional oncology | IA | Non-ionic iodinated contrast agents: Omnipaque or Iopromide | Mean 70.2 ± 16.3 mL (non-CIN) vs. 71.6 ± 12.8 mL (CIN) | 0%^v^ | CIN defined as increase in serum creatinine ≥0.5 mg/dL (50 µmol/L) or ≥25% from baseline within 48–72 h after contrast administration in absence of alternative causes | Serum creatinine assessed within 48–96 h after TACE |
| Sohn (2020) [11] | Republic of Korea | Retrospective historical cohort study | 347 HCC patients undergoing TACE | Child–Pugh class A HCC patients undergoing TACE as initial therapy | TACE | Embolization/interventional oncology | IA | Iodized oil (Lipiodol) with doxorubicin or cisplatin during TACE angiography | NR | 0%^w^ | ICA-AKI criteria: increase in serum creatinine ≥0.3 mg/dL within 48 h or ≥50% increase from baseline within prior 7 days | Renal follow-up at 1 day, 2 months, and 4 months after TACE |
| Soliman (2020) [10] | United States | Single-center retrospective cohort study | 106 HAE procedures in 84 patients | Patients undergoing HAE for primary or secondary hepatic malignancies | HAE | Embolization/interventional oncology | IA | Iohexol (Omnipaque 300) | Mean total contrast volume approximately 174 mL | 0%^x^ | CIN defined as 25% increase above baseline serum creatinine or absolute increase ≥0.5 mg/dL within 72 h post-embolization | Serum creatinine followed within 72 h after embolization |
| Song (2013) [9] | Republic of Korea | Retrospective two-center cohort study | 88 patients | Patients undergoing bronchial arteriography or BAE for haemoptysis | Bronchial arteriography and BAE | Embolization/interventional oncology | IA | Iodixanol (Visipaque, 290 mOsm/kg) and iopromide (Ultravist, 770 mOsm/kg) | Approximate mean contrast volume 218–233 mL | 4.5%^y^ | CIN defined as increase in serum creatinine >25% from baseline or absolute increase ≥0.5 mg/dL between 48 and 96 h after procedure | Serum creatinine assessed between 48 and 96 h after bronchial arteriography or BAE |
| Tao (2017) [8] | China | Single-center retrospective cohort study | 2015 patients | Patients with ischemic or haemorrhagic stroke undergoing cerebral angiography with or without endovascular therapy | Cerebral angiography ± endovascular therapy | Neuroendovascular/carotid procedures | IA | Iopromide (Ultravist 370) in 96.2% of patients; iodixanol (Visipaque) in remaining patients | Mean contrast volume approximately 70 mL | 50.3%^z^ | CI-AKI defined according to ESUR criteria as increase in serum creatinine by ≥44 μmol/L or ≥25% from baseline within 48 h after contrast exposure in absence of alternative etiology | Serum creatinine assessed before and within 48 h after cerebral angiography; 1-year mortality follow-up |
| Xu (2025) [7] | China | Single-center retrospective cohort study | 171 patients | Patients with haemoptysis undergoing sequential CTA and emergency BAE | Sequential CTA and BAE | Embolization/interventional oncology | Mixed IV + IA | Nonionic iodinated contrast medium: iohexol 350 mg I/mL | Approximate total contrast exposure 240–270 mL | 15.2%^†^ | CA-AKI defined as increase in serum creatinine ≥26.5 μmol/L or ≥1.5× baseline serum creatinine after contrast exposure | Serum creatinine assessed 24–72 h after BAE |
| Yoo (2020) [6] | Republic of Korea | Retrospective multicenter registry cohort study | 601 patients | Patients with AIS undergoing EVT | EVT for AIS | Neuroendovascular/carotid procedures | IA | Iodixanol (Visipaque) or iopamidol (Pamiray) | Approximate contrast exposure 70–90 mL | 48.6%^§^ | AKI defined according to KDIGO criteria as increase in serum creatinine ≥0.3 mg/dL within 48 h or ≥1.5× baseline serum creatinine within 7 days | Serum creatinine assessed from next day to 7 days after EVT; 3-month functional and mortality follow-up |
| Zhou (2014) [5] | China | Retrospective cohort study | 132 patients | Patients with HCC undergoing TACE | TACE | Embolization/interventional oncology | NR | Iodinated contrast agent used during TACE (specific agent NR) | NR | NR | AKI defined according to AKIN criteria as increase in serum creatinine ≥0.3 mg/dL or ≥50% from baseline within 48 h after TACE | Serum creatinine assessed within 48 h after TACE |

Abbreviations: AIS, acute ischemic stroke; AKI, acute kidney injury; AKIN, Acute Kidney Injury Network; ARF, acute renal failure; aSAH, aneurysmal subarachnoid haemorrhage; AVM, arteriovenous malformation; BAE, bronchial artery embolization; CA-AKI, contrast-associated acute kidney injury; CAN-REST, Contrast-Associated Nephropathy Risk Evaluation in Acute Ischemic Stroke After Endovascular Thrombectomy; CAS, carotid artery stenosis; CG-GFR, Cockcroft–Gault glomerular filtration rate; CI, confidence interval; CI-AKI, contrast-induced acute kidney injury; CIN, contrast-induced nephropathy; CKD, chronic kidney disease; CT, computed tomography; CTA, computed tomography angiography; cTACE, conventional transarterial chemoembolization; CTP, computed tomography perfusion; DAVF, dural arteriovenous fistula; DEMs, drug-eluting microspheres; DSA, digital subtraction angiography; eGFR, estimated glomerular filtration rate; ESUR, European Society of Urogenital Radiology; EVT, endovascular thrombectomy; GFR, glomerular filtration rate; HAE, hepatic artery embolization; HCC, hepatocellular carcinoma; ICA, International Club of Ascites; IA, intra-arterial; ICD-9-CM, International Classification of Diseases, Ninth Revision, Clinical Modification; ICU, intensive care unit; IV, intravenous; IQR, interquartile range; KDIGO, Kidney Disease: Improving Global Outcomes; mRS, modified Rankin Scale; NGAL, neutrophil gelatinase-associated lipocalin; NR, not reported; PC-AKI, post-contrast acute kidney injury; POD, postoperative day; RCIN, radiocontrast-induced nephropathy; SAH, subarachnoid haemorrhage; TACE, transcatheter arterial chemoembolization; TAE, transcatheter arterial embolization; TAI, transarterial infusion chemotherapy.

^a^ CKD was defined as GFR ≤50 mL/min using the Modification of Diet in Renal Disease (MDRD) equation; 10 patients met this criterion.

^b^ TACE protocols differed across participating centers. The Spanish cohort used doxorubicin or cisplatin with iodized oil/lipiodol and gelatin sponge embolization; the Israeli cohort used doxorubicin with lipiodol and gelfoam slurry/powder; and the Hong Kong cohort used a cisplatin–lipiodol emulsion up to 60 mL with gelatin sponge embolization.

^c^ CKD was defined as GFR <60 mL/min/1.73 m² estimated from serum creatinine; 4/189 patients met this criterion (2.1%).

^d^ Baseline renal impairment defined as eGFR <60 mL/min/1.73 m²; present in 93/336 patients (27.7%), including 86 patients with eGFR 30–59 and 7 patients with eGFR <30 mL/min/1.73 m².

^e^ Baseline renal impairment defined as eGFR <60 mL/min/1.73 m²; present in 47/305 treatment sessions (15.4%), including 45 sessions with eGFR 30–60 and 2 sessions with eGFR 15–30 mL/min/1.73 m².

^f^ CKD defined as eGFR <60 mL/min/1.73 m²; present in 78/270 treatment sessions (29.0%).

^g^ Baseline CKD prevalence was not explicitly reported; however, patients with ascites had significantly lower eGFR and higher serum creatinine levels than patients without ascites.

^h^ Chronic renal failure was present in 2/230 patients (0.9%) and was defined as eGFR <60 mL/min/1.73 m² or markers of kidney damage for ≥3 months.

^i^ All included patients met criteria for CKD, defined as pre-procedural eGFR <60 mL/min/1.73 m², corresponding to CKD stages 3–5.

^j^ Patients with severe baseline renal impairment (GFR <50 mL/min/1.73 m²) and dialysis-dependent chronic kidney disease were excluded.

^k^ Patients with baseline CKD and end-stage renal disease (ESRD) were excluded before cohort matching.

^l^ One patient (0.5%) had pre-existing renal disease prior to coil embolization.

^m^ Baseline CKD defined as CG-GFR <60 mL/min/1.73 m²; present in 108/265 patients.

^n^ CKD present in 2/228 patients (0.9%), defined as eGFR <60 mL/min/1.73 m², albuminuria, or structural kidney abnormalities for >3 months.

^o^ Baseline renal impairment defined as eGFR <60 mL/min/1.73 m²; present in 112/601 patients (18.6%), including 15 patients with eGFR <30 mL/min/1.73 m².

^p^ Patients with baseline serum creatinine >1.5 mg/dL were excluded prior to TACE; therefore formal baseline CKD prevalence was not directly reported.

^q^ Patients with preprocedural eGFR ≤30 mL/min/1.73 m² or documented CKD were excluded; therefore baseline CKD prevalence in the analyzed cohort was effectively 0%.

^r^ Baseline renal impairment defined as admission serum creatinine >1.2 mg/dL in men or >0.95 mg/dL in women; present in 7/75 patients (9.3%).

^s^ Known chronic kidney disease present in 2,604/6,638 patients (39.2%) based on pre-admission clinical history or medical records.

^t^ One patient (2.1%) had baseline eGFR <45 mL/min/1.73 m² before TAE.

^u^ Baseline chronic kidney disease defined as serum creatinine ≥1.5 mg/dL; present in 31/194 patients (16%). Three additional patients were receiving chronic haemodialysis before intervention.

^v^ Patients with baseline renal insufficiency were excluded before enrolment; renal insufficiency defined as serum creatinine >133 µmol/L.

^w^ Patients with pre-TACE serum creatinine >1.5 mg/dL were excluded before enrolment; therefore formal baseline CKD prevalence was effectively 0% in the analyzed cohort.

^x^ Patients with CKD stage ≥3 (GFR <60 mL/min/1.72 m²) were excluded before analysis; therefore baseline CKD prevalence in the analyzed cohort was effectively 0%.

^y^ Baseline renal insufficiency was present in 4/88 patients (4.5%). Patients with severe renal insufficiency (serum creatinine ≥4.0 mg/dL) or dialysis dependence were excluded before enrolment.

^z^ Baseline renal impairment defined as eGFR <60 mL/min/1.73 m²; present in 1014/2015 patients (50.3%).

^†^ Baseline CKD prevalence defined by CKD history; present in 26/171 patients (15.2%). Severe renal impairment (eGFR <30 mL/min/1.73 m²) was present in 5 patients (2.9%).

^§^ Baseline renal impairment classified according to eGFR categories: eGFR <60 mL/min/1.73 m² present in 292/601 patients (48.6%), including 252 patients with eGFR 30–59 and 40 patients with eGFR <30 mL/min/1.73 m².

Note: Procedure domains were classified into predefined endovascular/interventional categories for subgroup synthesis. Baseline CKD prevalence was extracted as reported by the original studies, including studies reporting baseline renal impairment, reduced eGFR, or exclusion of patients with severe renal dysfunction. Contrast volume reflects total iodinated contrast administered during the index procedure where available. Contrast administration route was classified according to information explicitly reported in the original study. IA indicates intra-arterial iodinated contrast administration; Mixed IV + IA indicates both intravenous and intra-arterial iodinated contrast exposure within the same diagnostic–therapeutic pathway; NR indicates that the route of contrast administration was not reported in sufficient detail for classification. Route was not inferred solely from the procedural designation.

Supplementary Table S4. Reported risk factors associated with contrast-associated acute kidney injury following interventional radiology procedures.

| Study (Author, Year) | Risk Factor | Effect Measure | Effect Estimate (95% CI) | Adjusted Analysis | Statistically Significant |
| --- | --- | --- | --- | --- | --- |
| Aoe (2019) [43] | Tumor size >5 cm | OR | 5.76 (2.34–14.14) | No (univariable analysis) | Yes |
|  | CKD | OR | 2.54 (1.05–6.14) | No (univariable analysis) | Yes |
|  | Haemoglobin (per 1 g/dL increase) | OR | 0.79 (0.64–0.98) | No (univariable analysis) | Yes |
|  | Albumin (per 1 g/dL increase) | OR | 0.44 (0.19–1.02) | No (univariable analysis) | Yes |
|  | Tumor size >5 cm | OR | 7.81 (2.99–20.46) | Yes (multivariable logistic regression) | Yes |
|  | Albumin (per 1 g/dL increase) | OR | 0.29 (0.11–0.75) | Yes (multivariable logistic regression) | Yes |
| Cao (2021) [42] | Tumor size >5 cm | OR | 6.22 (1.61–23.93) | No (univariable analysis) | Yes |
|  | TACE frequency | OR | 8.34 (2.81–24.73) | No (univariable analysis) | Yes |
|  | Contrast agent dosage (mL) | OR | 1.06 (1.00–1.12) | No (univariable analysis) | Yes |
|  | Volume of iodized oil (mL) | OR | 1.34 (1.15–1.55) | No (univariable analysis) | Yes |
|  | Solitary kidney | OR | 7.81 (1.40–43.61) | No (univariable analysis) | Yes |
|  | Serum albumin | OR | 0.88 (0.79–0.98) | No (univariable analysis) | Yes |
|  | Haemoglobin | OR | 0.95 (0.92–0.99) | No (univariable analysis) | Yes |
|  | TACE frequency | OR | 8.80 (2.20–35.21) | Yes (multivariable logistic regression) | Yes |
|  | Volume of iodized oil (mL) | OR | 1.31 (1.09–1.57) | Yes (multivariable logistic regression) | Yes |
|  | Serum albumin | OR | 0.90 (0.81–0.99) | Yes (multivariable logistic regression) | Yes |
| Cho (2011) [41] | Child–Pugh score | Mean difference | 6.0 ± 1.3 vs. 6.8 ± 1.9 | No (group comparison) | Yes |
|  | Ascites | % occurrence | 14.4% vs. 40.0% | No (group comparison) | Yes |
|  | Contrast medium volume | Mean difference | 257.3 ± 66.8 vs. 275.0 ± 44.4 mL | No (group comparison) | Yes |
|  | Total bilirubin | Mean difference | 1.3 ± 1.7 vs. 3.43 ± 8.0 mg/dL | No (group comparison) | Yes |
|  | Baseline serum creatinine | Mean difference | 0.9 ± 0.3 vs. 1.0 ± 0.5 mg/dL | No (group comparison) | Yes |
|  | Baseline GFR (MDRD) | Mean difference | 90.5 ± 21.8 vs. 88.4 ± 29.6 mL/min | No (group comparison) | Yes |
|  | Age | OR | 0.95 (0.90–0.99) | Yes (multivariable logistic regression) | Yes |
|  | Child–Pugh score | OR | 1.4 (1.05–1.92) | Yes (multivariable logistic regression) | Yes |
|  | Diabetes mellitus | OR | 0.7 (0.23–2.14) | Yes (multivariable logistic regression) | No |
|  | Contrast volume | OR | 1.0 (0.99–1.01) | Yes (multivariable logistic regression) | No |
|  | Initial GFR | OR | 0.4 (0.06–2.21) | Yes (multivariable logistic regression) | No |
| Cohen (2014) [40] | Age group (<65 vs. 65–75 vs. >75 years) | % occurrence | 21% vs. 16% vs. 10% AKI incidence | No (group comparison) | No |
| Delgado Acosta (2020) [39] | Diabetes mellitus | OR | 2.66 (1.04–6.79) | No (univariable logistic regression) | Yes |
|  | Baseline creatinine 1.3–2.5 mg/dL | OR | 4.39 (1.62–11.95) | No (univariable logistic regression) | Yes |
|  | Baseline creatinine 1.3–2.5 mg/dL | OR | NR | Yes (multivariable logistic regression) | Yes |
|  | Diabetes mellitus | OR | NR | Yes (multivariable logistic regression) | No |
| Diprose (2019) [38] | eGFR <30 mL/min/1.73 m² vs. >90 mL/min/1.73 m² | OR | 31.50 (4.05–244.98) | No (univariable logistic regression) | Yes |
|  | eGFR <30 mL/min/1.73 m² vs. >90 mL/min/1.73 m² | OR | 19.93 (2.33–170.74) | Yes (propensity score–adjusted logistic regression) | Yes |
|  | Diabetes mellitus | OR | 5.31 (1.57–17.94) | No (univariable logistic regression) | Yes |
|  | Dyslipidaemia | OR | 3.66 (0.95–14.06) | No (univariable logistic regression) | Borderline |
|  | Baseline NIHSS (per score increase) | OR | 1.07 (0.99–1.14) | No (univariable logistic regression) | Borderline |
|  | eGFR 60–89 vs. >90 mL/min/1.73 m² | OR | 0.82 (0.13–4.99) | No (univariable logistic regression) | No |
|  | eGFR 30–59 vs. >90 mL/min/1.73 m² | OR | 1.52 (0.25–9.32) | No (univariable logistic regression) | No |
|  | eGFR 60–89 vs. >90 mL/min/1.73 m² | OR | 0.78 (0.12–4.95) | Yes (propensity score–adjusted logistic regression) | No |
|  | eGFR 30–59 vs. >90 mL/min/1.73 m² | OR | 1.48 (0.23–9.37) | Yes (propensity score–adjusted logistic regression) | No |
| Hao (2014) [37] | Total bilirubin >13.5 µmol/L | OR | 1.87 (1.04–3.35) | Yes (multivariable logistic regression; Barrett and Parfrey criteria) | Yes |
|  | Haemoglobin <120 g/L | OR | 1.82 (1.01–3.26) | Yes (multivariable logistic regression; Barrett and Parfrey criteria) | Yes |
|  | Age >55 years | OR | 3.45 (1.10–10.79) | Yes (multivariable logistic regression; AKIN criteria) | Yes |
|  | Post-TACE AKI history | OR | 7.10 (1.38–36.43) | Yes (multivariable logistic regression; AKIN criteria) | Yes |
|  | AST >55 U/L | OR | 4.42 (1.79–10.91) | Yes (multivariable logistic regression; AKIN criteria) | Yes |
|  | Albumin >35 g/L | OR | 1.36 (0.74–2.53) | Yes (multivariable logistic regression; Barrett and Parfrey criteria) | No |
|  | PT-INR >1.05 | OR | 1.52 (0.85–2.71) | Yes (multivariable logistic regression; Barrett and Parfrey criteria) | No |
|  | Total bilirubin >13.5 µmol/L | OR | 2.07 (0.76–5.57) | Yes (multivariable logistic regression; AKIN criteria) | No |
| Hayakawa (2014) [36] | Decreased serum albumin | Mean difference | 2.9 ± 0.4 vs. 3.3 ± 0.4 g/dL | No (group comparison) | Borderline |
|  | Liver cirrhosis | % occurrence | 25.0% vs. 57.6% | No (group comparison) | No |
|  | Diabetes mellitus | % occurrence | 25.0% vs. 34.0% | No (group comparison) | No |
|  | Hypertension | % occurrence | 25.0% vs. 33.7% | No (group comparison) | No |
|  | Ascites | % occurrence | 25.0% vs. 7.4% | No (group comparison) | No |
|  | Child–Pugh score | Mean difference | 6.5 ± 0.9 vs. 6.1 ± 1.1 | No (group comparison) | No |
|  | Advanced HCC | % occurrence | 62.5% vs. 45.4% | No (group comparison) | No |
|  | Baseline eGFR <60 mL/min/1.73 m² | % occurrence | No CIN events observed | No (descriptive analysis) | No |
| Hayashi (2020) [35] | Ascites | Regression coefficient | 0.059 | Yes (multivariable linear regression) | Yes |
|  | eGFR (per 10 mL/min/1.73 m² increase) | Regression coefficient | −0.008 | Yes (multivariable linear regression) | Yes |
|  | Diabetes mellitus | Regression coefficient | 0.072 | Yes (multivariable linear regression) | Yes |
|  | ALBI grade 2 | Regression coefficient | 0.053 | Yes (multivariable linear regression) | Yes |
|  | ALBI grade 3 | Regression coefficient | 0.103 | Yes (multivariable linear regression) | Yes |
|  | Age (per 10 years) | Regression coefficient | 0.010 | Yes (multivariable linear regression) | No |
|  | Male sex | Regression coefficient | −0.007 | Yes (multivariable linear regression) | No |
|  | Volume of contrast/kg | Regression coefficient | 0.006 | Yes (multivariable linear regression) | No |
|  | Use of cisplatin | Regression coefficient | −0.017 | Yes (multivariable linear regression) | No |
|  | Hypertension | Regression coefficient | −0.014 | Yes (multivariable linear regression) | No |
|  | HCC stage | Regression coefficient | −0.006 | Yes (multivariable linear regression) | No |
| Hsu (2010) [34] | Serum albumin <3.3 g/dL | OR | 7.3 (1.47–35.7) | Yes (multivariable logistic regression) | Yes |
|  | Age <66 years | % occurrence | 73% vs. 46% | No (group comparison) | No |
|  | Diabetes mellitus | % occurrence | 45% vs. 26% | No (group comparison) | No |
|  | Child–Turcotte–Pugh class B | % occurrence | 82% vs. 68% | No (group comparison) | No |
|  | Tumor size <5 cm | % occurrence | 27% vs. 42% | No (group comparison) | No |
|  | Multiple tumors | % occurrence | 64% vs. 38% | No (group comparison) | No |
|  | eGFR <65 mL/min/1.73 m² | % occurrence | 64% vs. 49% | No (group comparison) | No |
|  | Serum sodium <139 mmol/L | % occurrence | 64% vs. 42% | No (group comparison) | No |
|  | Post-embolization gastrointestinal bleeding | % occurrence | 18% vs. 4% | No (group comparison) | No |
|  | Nephrotoxic agent exposure | % occurrence | 45% vs. 23% | No (group comparison) | No |
| Huo (2004) [33] | Number of previous TACE sessions | OR | 1.65 (1.13–2.41) | Yes (multivariable logistic regression) | Yes |
|  | Child–Pugh class B | OR | 12.82 (2.44–67.29) | Yes (multivariable logistic regression) | Yes |
|  | Severe postembolization syndrome | OR | 6.64 (1.60–27.49) | Yes (multivariable logistic regression) | Yes |
|  | Diabetes mellitus | % occurrence | 42% vs. 18% | No (group comparison) | Borderline |
|  | Lower baseline serum albumin | Mean difference | 3.6 ± 0.3 vs. 3.8 ± 0.3 g/dL | No (group comparison) | Borderline |
|  | Use of nephrotoxic agents | % occurrence | 33% vs. 16% | No (group comparison) | No |
|  | Multiple tumors | % occurrence | 50% vs. 38% | No (group comparison) | No |
|  | Tumor size >5 cm | % occurrence | 33% vs. 19% | No (group comparison) | No |
|  | Male sex | % occurrence | 100% vs. 79% | No (group comparison) | No |
|  | HBsAg positivity | % occurrence | 75% vs. 77% | No (group comparison) | No |
| Huo (2004) [32] | Child–Pugh class B | OR | 2.6 (1.3–5.3) | Yes (multivariable GEE analysis) | Yes |
|  | Additional TACE session | OR | 1.3 (1.2–1.5) | Yes (multivariable GEE analysis) | Yes |
|  | Diabetes mellitus | OR | 5.2 (2.4–11.2) | Yes (multivariable GEE analysis for prolonged ARF) | Yes |
|  | Child–Pugh class B | OR | 4.3 (2.0–10.0) | Yes (multivariable GEE analysis for prolonged ARF) | Yes |
|  | Diabetes mellitus | % occurrence | 36% vs. 23% | No (group comparison) | Borderline |
|  | ≥6 TACE sessions | % occurrence | 29% vs. 15% | No (group comparison) | Yes |
|  | Child–Pugh class B | % occurrence | 23% vs. 17% | No (group comparison) | No |
|  | Tumor size >5 cm | % occurrence | 41% vs. 31% | No (group comparison) | No |
|  | Multifocal tumors | % occurrence | 54% vs. 49% | No (group comparison) | No |
|  | AFP >400 ng/mL | % occurrence | 27% vs. 32% | No (group comparison) | No |
|  | HBsAg positivity | % occurrence | 54% vs. 63% | No (group comparison) | No |
|  | Exposure to nephrotoxic agents | % exposure | 8% overall exposure | No (descriptive analysis) | No |
| Jia (2019) [31] | Consecutive multidose contrast exposure (CTA + DSA vs. CTA alone) | % occurrence | 7.4% vs. 2.3% AKI incidence | No (group comparison) | No |
| Joo (2019) [30] | Diabetes mellitus | % occurrence | 30.8% vs. 9.7% | No (group comparison) | Yes |
|  | Age ≥75 years | % occurrence | 30.8% vs. 6.5% | No (group comparison) | Yes |
|  | Contrast media volume exceeding MACD | % occurrence | 15.4% vs. 2.8% | No (group comparison) | Borderline |
|  | Cumulative contrast dose ≥250 mL | % occurrence | 8.6% (5/58 patients) | No (descriptive analysis) | No |
|  | Chronic renal failure | % occurrence | 7.7% vs. 0.5% | No (group comparison) | No |
|  | Concurrent extraventricular drainage | % occurrence | 15.4% vs. 2.8% | No (group comparison) | Borderline |
|  | Hypertension | % occurrence | 53.8% vs. 41.5% | No (group comparison) | No |
|  | Dyslipidaemia | % occurrence | 15.4% vs. 7.8% | No (group comparison) | No |
|  | Coronary artery disease | % occurrence | 7.7% vs. 4.6% | No (group comparison) | No |
|  | Baseline eGFR <90 mL/min/1.73 m² | % occurrence | 38.5% vs. 54.8% | No (group comparison) | No |
|  | Anemia | % occurrence | 23.1% vs. 12.4% | No (group comparison) | No |
|  | Diagnostic angiography within previous 48 h | % occurrence | 38.5% vs. 27.6% | No (group comparison) | No |
|  | Postoperative contrast-enhanced CT | % occurrence | 53.8% vs. 32.3% | No (group comparison) | No |
| Kim (2018) [29] | Stage 5 CKD | % occurrence | 16.7% vs. 0% in stage 3–4 CKD | No (descriptive analysis) | NR |
|  | IgA nephropathy | % occurrence | Present in 100% of CIN cases | No (descriptive analysis) | NR |
|  | Therapeutic neuroendovascular intervention vs. diagnostic angiography | % occurrence | 2.3% vs. 0% CIN incidence | No (descriptive analysis) | NR |
| Kim (2020) [28] | hs-CRP >5 mg/dL | OR | 4.58 (1.26–16.66) | No (simple logistic regression) | Yes |
|  | hs-CRP >5 mg/dL | OR | 5.05 (1.34–18.9) | Yes (multivariable logistic regression) | Yes |
|  | uNGAL >30 ng/mL | OR | 2.95 (0.85–10.21) | No (simple logistic regression) | Borderline |
|  | uNGAL >30 ng/mL | OR | 3.56 (0.98–12.8) | Yes (multivariable logistic regression) | Borderline |
|  | Smoking | OR | 2.47 (0.78–7.43) | No (simple logistic regression) | Borderline |
|  | Smoking | OR | 2.32 (0.79–6.78) | Yes (multivariable logistic regression) | No |
|  | Age >75 years | OR | 0.98 (0.20–4.63) | No (simple logistic regression) | No |
|  | Contrast volume >150 mL | OR | 0.98 (0.20–4.63) | No (simple logistic regression) | No |
|  | Anemia | OR | 1.57 (0.56–4.37) | No (simple logistic regression) | No |
|  | Baseline GFR <90 mL/min/1.73 m² | OR | 0.48 (0.17–1.38) | No (simple logistic regression) | No |
| Laible (2021) [27] | Additional thrombolysis (vs. EVT alone) | OR | 3.38 (1.53–7.50) | No (univariable logistic regression) | Yes |
|  | Additional thrombolysis (vs. EVT alone) | OR | 3.47 (1.56–7.71) | Yes (multivariable logistic regression) | Yes |
|  | Baseline renal impairment (eGFR <60 vs. ≥60 mL/min/1.73 m²) | OR | 0.23 (0.07–0.78) | No (univariable logistic regression) | Yes |
|  | Baseline renal impairment (eGFR <60 vs. ≥60 mL/min/1.73 m²) | OR | 0.23 (0.07–0.76) | Yes (multivariable logistic regression) | Yes |
|  | Failed recanalization (TICI 0–2a vs. TICI 2b–3) | OR | 1.86 (0.84–4.14) | No (univariable logistic regression) | No |
|  | Diabetes mellitus | OR | 1.55 (0.70–3.44) | No (univariable logistic regression) | No |
|  | Hypertension | OR | 1.13 (0.46–2.81) | No (univariable logistic regression) | No |
|  | Coronary heart disease | OR | 0.54 (0.21–1.44) | No (univariable logistic regression) | No |
|  | Age (per year increase) | OR | 0.99 (0.96–1.01) | No (univariable logistic regression) | No |
|  | NIHSS at admission (per point increase) | OR | 0.99 (0.94–1.03) | No (univariable logistic regression) | No |
|  | Posterior circulation stroke | OR | 0.67 (0.16–2.84) | No (univariable logistic regression) | No |
|  | Additional contrast administration | OR | 1.23 (0.29–5.26) | No (univariable logistic regression) | No |
|  | Systolic blood pressure at admission (per mmHg increase) | OR | 1.00 (0.99–1.02) | No (univariable logistic regression) | No |
| Lee (2017) [26] | cTACE exposure vs. no cTACE | HR | 1.66 (1.17–2.34) | Yes (multivariable Cox proportional hazards model) | Yes |
|  | Male sex | HR | 1.63 (1.09–2.44) | Yes (multivariable Cox proportional hazards model) | Yes |
|  | Age 50–64 years | HR | 1.90 (1.10–3.29) | Yes (multivariable Cox proportional hazards model) | Yes |
|  | Presence of ≥1 baseline comorbidity | HR | 1.56 (1.02–2.37) | Yes (multivariable Cox proportional hazards model) | Yes |
|  | cTACE ≤3 sessions | HR | 1.74 (1.23–2.48) | Yes (multivariable Cox proportional hazards model) | Yes |
|  | Undergoing contrast-enhanced CT | HR | 1.62 (1.15–2.30) | Yes (multivariable Cox proportional hazards model) | Yes |
|  | No diuretic use | HR | 1.70 (1.09–2.66) | Yes (multivariable Cox proportional hazards model) | Yes |
|  | No ACE inhibitor use | HR | 1.90 (1.27–2.86) | Yes (multivariable Cox proportional hazards model) | Yes |
|  | No ARB use | HR | 1.71 (1.20–2.42) | Yes (multivariable Cox proportional hazards model) | Yes |
|  | No metformin use | HR | 1.68 (1.14–2.48) | Yes (multivariable Cox proportional hazards model) | Yes |
|  | Female sex | HR | 1.66 (0.84–3.28) | Yes (multivariable Cox proportional hazards model) | No |
|  | Age ≤49 years | HR | 1.78 (0.80–3.95) | Yes (multivariable Cox proportional hazards model) | No |
|  | Age ≥65 years | HR | 1.34 (0.76–2.35) | Yes (multivariable Cox proportional hazards model) | No |
|  | cTACE >3 sessions | HR | 1.06 (0.48–2.33) | Yes (multivariable Cox proportional hazards model) | No |
| Lee (2018) [25] | Initial GCS score ≤8 | % occurrence | 50.0% vs. 19.7% | No (group comparison) | Yes |
|  | Older age (≥65 years) | % occurrence | 50.0% vs. 30.3% | No (group comparison) | No |
|  | Female sex | % occurrence | 42.9% vs. 65.7% | No (group comparison) | Borderline |
|  | Large aneurysm (≥10 mm) | % occurrence | 35.7% vs. 17.4% | No (group comparison) | No |
|  | Diabetes mellitus | % occurrence | 14.3% vs. 5.6% | No (group comparison) | No |
|  | Contrast volume | Mean difference | 293 ± 73 vs. 267 ± 76 mL | No (group comparison) | No |
|  | Non-ionic low-osmolar vs. iso-osmolar contrast agents | % occurrence | 11.3% vs. 4.5% | No (group comparison) | Borderline |
|  | Severe neurological status (GCS ≤8) | OR | NR | Yes (multivariable logistic regression) | Yes |
|  | Prior contrast-enhanced imaging before embolization | % occurrence | 14/178 patients vs. 0/14 patients without prior imaging | No (descriptive analysis) | NR |
| Lin (2025) [24] | Proteinuria ≥1+ | OR | 2.25 (1.16–4.37) | No (univariable analysis) | Yes |
|  | HbA1c ≥7% | OR | 2.08 (1.05–4.12) | No (univariable analysis) | Yes |
|  | CG-GFR <45 mL/min/1.73 m² | OR | 0.45 (0.22–0.93) | No (univariable analysis) | Yes |
|  | Hypertension | OR | 1.66 (0.97–2.83) | No (univariable analysis) | Borderline |
|  | AJCC tumor staging ≥III (day-3 creatinine worsening) | OR | 1.61 (0.96–2.70) | No (univariable analysis) | Borderline |
|  | Diabetes mellitus | OR | 1.34 (0.79–2.26) | No (univariable analysis) | No |
|  | Liver cirrhosis | OR | 1.12 (0.66–1.90) | No (univariable analysis) | No |
|  | Serum albumin <3 g/dL | OR | 2.50 (0.81–7.67) | No (univariable analysis) | No |
|  | Hemoglobin <10 g/dL | OR | 0.98 (0.49–1.97) | No (univariable analysis) | No |
|  | Embolized tumor size ≥5 cm | OR | 1.48 (0.88–2.50) | No (univariable analysis) | No |
|  | TACE using DEMs | OR | 1.12 (0.62–2.04) | No (univariable analysis) | No |
|  | Total IV fluid at day of TACE <1000 mL | OR | 1.57 (0.53–4.66) | No (univariable analysis) | No |
|  | Proteinuria excluded + HbA1c ≥7% | OR | 2.94 (1.26–6.85) | No (sensitivity analysis) | Yes |
|  | Proteinuria excluded + HbA1c ≥7.5% | OR | 2.63 (1.08–6.42) | No (sensitivity analysis) | Yes |
|  | Proteinuria excluded + CG-GFR <60 mL/min/1.73 m² | OR | 0.39 (0.19–0.77) | No (sensitivity analysis) | Yes |
|  | Proteinuria excluded + CG-GFR <45 mL/min/1.73 m² | OR | 0.07 (0.009–0.50) | No (sensitivity analysis) | Yes |
|  | Serum albumin <3 g/dL at admission | OR | 4.42 (1.40–13.98) | No (univariable analysis) | Yes |
|  | AJCC tumor staging ≥III (continued renal improvement after discharge) | OR | 0.33 (0.19–0.59) | No (univariable analysis) | Yes |
|  | Embolized tumor size ≥5 cm | OR | 0.39 (0.22–0.71) | No (univariable analysis) | Yes |
|  | Adverse events of TACE ≥Grade 3 | OR | 0.45 (0.25–0.82) | No (univariable analysis) | Yes |
|  | Admission duration ≥7 days | OR | 0.53 (0.30–0.93) | No (univariable analysis) | Yes |
| Mitaka (2023) [23] | Diabetes mellitus | β coefficient | β = 0.183 | Yes (multiple linear regression for clinical CA-AKI) | Yes |
|  | CAS | β coefficient | β = 0.181 | Yes (multiple linear regression for clinical CA-AKI) | Yes |
|  | Diabetes mellitus | β coefficient | β = 0.349 | Yes (multiple linear regression for clinical/subclinical CA-AKI using serum creatinine model) | Yes |
|  | CAS | β coefficient | β = 0.400 | Yes (multiple linear regression for clinical/subclinical CA-AKI using serum creatinine model) | Yes |
|  | Diabetes mellitus | β coefficient | β = 0.357 | Yes (multiple linear regression for clinical/subclinical CA-AKI using eGFR model) | Yes |
|  | CAS | β coefficient | β = 0.348 | Yes (multiple linear regression for clinical/subclinical CA-AKI using eGFR model) | Yes |
|  | Body weight^a^ | Linear regression association | p = 0.023 | No (simple linear regression) | Yes |
|  | Preoperative serum creatinine^a^ | Linear regression association | p = 0.012 | No (simple linear regression) | Yes |
|  | Haemorrhage volume^a^ | Linear regression association | p = 0.012 | No (simple linear regression) | Yes |
|  | Preoperative eGFR^a^ | Linear regression association | p = 0.033 | No (simple linear regression for clinical/subclinical CA-AKI) | Yes |
|  | Hypertension | % occurrence | 100% vs. 48.7% | No (group comparison) | No |
|  | CKD | % occurrence | 0% vs. 0.9% | No (group comparison) | No |
|  | Contrast volume | Mean difference | 210.0 ± 14.1 vs. 182.2 ± 76.4 mL | No (group comparison) | No |
|  | Urine volume in ICU | Mean difference | 41.5 ± 7.8 vs. 182.3 ± 149.4 mL/h | No (group comparison) | Borderline |
|  | Anemia | % occurrence | 25.0% vs. 16.9% | No (group comparison) | No |
|  | Age | Mean difference | 71.5 ± 3.5 vs. 63.5 ± 12.8 years | No (group comparison) | No |
|  | Contrast amount (clinical/subclinical CA-AKI subgroup) | Mean difference | 165.0 ± 106.8 vs. 167.5 ± 50.5 mL | No (group comparison) | No |
| Murakami (2016) [22] | Child–Pugh class B or C | % occurrence | 71% vs. 41% | No (group comparison) | Yes |
|  | Low albumin level | Mean difference | 3.0 ± 0.5 vs. 3.4 ± 0.6 g/dL | No (group comparison) | Yes |
|  | Low haemoglobin level | Mean difference | 10.6 ± 2.0 vs. 11.8 ± 2.0 g/dL | No (group comparison) | Yes |
|  | Haemoglobin level | OR | 1.57 (1.02–2.40) | Yes (multivariable logistic regression) | Yes |
|  | Age | OR | 0.99 (0.91–1.07) | Yes (multivariable logistic regression) | No |
|  | Sex | OR | 0.29 (0.05–1.57) | Yes (multivariable logistic regression) | No |
|  | Baseline eGFR | OR | 0.98 (0.94–1.01) | Yes (multivariable logistic regression) | No |
|  | Contrast volume | OR | 0.99 (0.98–1.00) | Yes (multivariable logistic regression) | No |
|  | Hypertension | OR | 0.47 (0.12–1.84) | Yes (multivariable logistic regression) | No |
|  | Diabetes mellitus | OR | 0.86 (0.23–3.16) | Yes (multivariable logistic regression) | No |
|  | Child–Pugh class B or C | OR | 0.34 (0.07–1.61) | Yes (multivariable logistic regression) | No |
|  | Albumin | OR | 1.00 (0.22–4.43) | Yes (multivariable logistic regression) | No |
|  | Baseline eGFR <60 mL/min/1.73 m² | % occurrence | 36% vs. 40% | No (group comparison) | No |
|  | Previous TACE sessions | Mean difference | 3.4 ± 3.0 vs. 2.5 ± 2.6 | No (group comparison) | No |
|  | Contrast volume | Mean difference | 154 ± 43 vs. 147 ± 45 mL | No (group comparison) | No |
|  | Hypertension | % occurrence | 36% vs. 41% | No (group comparison) | No |
|  | Diabetes mellitus | % occurrence | 36% vs. 35% | No (group comparison) | No |
|  | Ascites | % occurrence | 14% vs. 17% | No (group comparison) | No |
|  | Multiple HCC lesions | % occurrence | 71% vs. 68% | No (group comparison) | No |
|  | Maximum HCC diameter | Mean difference | 37.0 ± 35.8 vs. 28.4 ± 18.8 mm | No (group comparison) | No |
| Myung (2020) [20] | Baseline eGFR <30 mL/min/1.73 m² | % occurrence | 46.7% vs. lower incidence in all other eGFR groups | No (group comparison/post hoc analysis) | Yes |
|  | Age >75 years | OR | NR | Yes (multivariable logistic regression) | Yes |
|  | CKD | OR | NR | Yes (multivariable logistic regression) | Yes |
|  | Medication use after contrast exposure^b^ | OR | NR | Yes (multivariable logistic regression) | Yes |
|  | Low serum albumin level | OR | NR | Yes (multivariable logistic regression) | Yes |
|  | Diabetes mellitus | Univariate association | Significant in univariate analysis | No (univariable logistic regression) | Yes |
|  | Atrial fibrillation | Univariate association | Significant in univariate analysis | No (univariable logistic regression) | Yes |
|  | ACE inhibitor use before CTA | Univariate association | Significant in univariate analysis | No (univariable logistic regression) | Yes |
|  | ARB use before CTA | Univariate association | Significant in univariate analysis | No (univariable logistic regression) | Yes |
|  | Proteinuria | Univariate association | Significant in univariate analysis | No (univariable logistic regression) | Yes |
|  | Low haemoglobin level | Univariate association | Significant in univariate analysis | No (univariable logistic regression) | Yes |
|  | Low haematocrit level | Univariate association | Significant in univariate analysis | No (univariable logistic regression) | Yes |
|  | High delta neutrophil index | Univariate association | Significant in univariate analysis | No (univariable logistic regression) | Yes |
|  | Low platelet count | Univariate association | Significant in univariate analysis | No (univariable logistic regression) | Yes |
|  | Low sodium concentration | Univariate association | Significant in univariate analysis | No (univariable logistic regression) | Yes |
|  | Baseline eGFR cutoff <43 mL/min/1.73 m² | ROC-derived cutoff | AUC 0.711 | Yes (adjusted ROC analysis) | Yes |
| Ösken (2021) [21] | Age | OR | 1.06 (1.01–1.11) | No (univariable logistic regression) | Yes |
|  | Baseline creatinine | OR | 5.33 (1.40–20.22) | No (univariable logistic regression) | Yes |
|  | eGFR | OR | 0.98 (0.96–0.99) | No (univariable logistic regression) | Yes |
|  | Haemoglobin | OR | 0.61 (0.48–0.79) | No (univariable logistic regression) | Yes |
|  | Lymphocyte count | OR | 0.99 (0.99–1.00) | No (univariable logistic regression) | Yes |
|  | Neutrophil-to-lymphocyte ratio | OR | 1.70 (1.25–2.31) | No (univariable logistic regression) | Yes |
|  | Age | OR | 1.06 (1.00–1.12) | Yes (multivariable logistic regression) | Yes |
|  | Baseline creatinine | OR | 4.40 (1.06–18.28) | Yes (multivariable logistic regression) | Yes |
|  | Haemoglobin | OR | 0.61 (0.46–0.80) | Yes (multivariable logistic regression) | Yes |
|  | Neutrophil-to-lymphocyte ratio | OR | 1.91 (1.39–2.63) | Yes (multivariable logistic regression) | Yes |
|  | Diabetes mellitus | OR | 2.02 (0.91–4.50) | No (univariable logistic regression) | No |
|  | Hypertension | OR | 1.73 (0.76–3.93) | No (univariable logistic regression) | No |
|  | Smoking | OR | 0.71 (0.20–2.46) | No (univariable logistic regression) | No |
|  | Atrial fibrillation | OR | 1.71 (0.65–4.47) | No (univariable logistic regression) | No |
|  | Coronary artery disease | OR | 1.24 (0.56–2.76) | No (univariable logistic regression) | No |
|  | Contrast media volume | OR | 1.00 (0.99–1.01) | No (univariable logistic regression) | No |
|  | Platelet-to-lymphocyte ratio | OR | 1.00 (1.00–1.00) | No (univariable logistic regression) | No |
|  | Neutrophil-to-lymphocyte ratio cutoff >2.33 | ROC-derived cutoff | AUC 0.64 (95% CI 0.54–0.75); sensitivity 60%; specificity 65% | Yes (ROC analysis) | Yes |
|  | Platelet-to-lymphocyte ratio cutoff >112.12 | ROC-derived cutoff | AUC 0.64 (95% CI 0.53–0.75); sensitivity 54%; specificity 69% | Yes (ROC analysis) | Yes |
| Ozdogru (2025) [19] | Age (per year increase) | OR | 1.05 (1.02–1.08) | No (univariable logistic regression) | Yes |
|  | Diabetes mellitus | OR | 2.51 (1.36–4.66) | No (univariable logistic regression) | Yes |
|  | Baseline GFR (per mL/min/1.73 m² increase) | OR | 0.97 (0.96–0.99) | No (univariable logistic regression) | Yes |
|  | Cerebral oedema | OR | 3.62 (1.96–6.71) | No (univariable logistic regression) | Yes |
|  | Baseline NIHSS score | OR | 1.19 (1.06–1.33) | No (univariable logistic regression) | Yes |
|  | Time to recanalization (per minute increase) | OR | 1.03 (1.01–1.04) | No (univariable logistic regression) | Yes |
|  | Contrast volume (per mL increase) | OR | 1.02 (1.01–1.03) | No (univariable logistic regression) | Yes |
|  | Body mass index | OR | 1.07 (1.00–1.15) | No (univariable logistic regression) | Yes |
|  | Systemic immune inflammation index | OR | 1.00 (1.00–1.00) | Yes (multivariable logistic regression model) | Yes |
|  | Systemic inflammation response index | OR | 1.17 (1.06–1.30) | No (univariable logistic regression) | Yes |
|  | Systemic inflammation response index | OR | 1.22 (1.06–1.39) | Yes (multivariable logistic regression) | Yes |
|  | Age (per year increase) | OR | 1.03 (0.99–1.07) | Yes (multivariable logistic regression) | No |
|  | Diabetes mellitus | OR | 1.33 (0.61–2.90) | Yes (multivariable logistic regression) | No |
|  | Lower baseline GFR | OR | 0.98 (0.97–1.00) | Yes (multivariable logistic regression) | Borderline |
|  | Cerebral oedema | OR | 3.60 (1.66–7.81) | Yes (multivariable logistic regression) | Yes |
|  | Baseline NIHSS | OR | 1.17 (1.02–1.34) | Yes (multivariable logistic regression) | Yes |
|  | Time to recanalization | OR | 1.03 (1.00–1.07) | Yes (multivariable logistic regression) | Yes |
|  | Contrast volume | OR | 0.99 (0.97–1.02) | Yes (multivariable logistic regression) | No |
|  | Body mass index | OR | 1.02 (0.94–1.12) | Yes (multivariable logistic regression) | No |
|  | Systemic immune inflammation index cutoff >1784 | ROC-derived cutoff | AUC 0.71 (95% CI 0.63–0.78); sensitivity 42.6%; specificity 89.0% | Yes (ROC analysis) | Yes |
|  | Systemic inflammation response index | ROC analysis | AUC 0.65 (95% CI 0.57–0.72); sensitivity 88.9%; specificity 37.9% | Yes (ROC analysis) | Yes |
|  | Neutrophil-to-lymphocyte ratio | ROC analysis | AUC 0.698 (95% CI 0.62–0.77); sensitivity 77.8%; specificity 49.3% | Yes (ROC analysis) | Yes |
|  | Platelet-to-lymphocyte ratio | ROC analysis | AUC 0.66 (95% CI 0.58–0.74); sensitivity 74.1%; specificity 51.1% | Yes (ROC analysis) | Yes |
| Park (2008) [18] | Hypertension | OR | 3.24 (1.21–8.72) | Yes (multivariable GEE analysis) | Yes |
|  | Lower baseline serum albumin | OR | 0.29 (0.15–0.56) | Yes (multivariable GEE analysis) | Yes |
|  | Higher baseline serum creatinine | OR | 12.02 (3.49–41.39) | Yes (multivariable GEE analysis) | Yes |
|  | Hypertension | % occurrence | 20.8% vs. 6.6% | No (group comparison) | Yes |
|  | HCV-Ab seropositivity | % occurrence | 25.0% vs. 9.9% | No (group comparison) | Yes |
|  | Child–Pugh class B or C | % occurrence | 45.8% vs. 26.4% | No (group comparison) | Yes |
|  | ≥3 TACE sessions | % occurrence | 50.0% vs. 20.8% | No (group comparison) | Yes |
|  | Diuretic use | % occurrence | 62.5% vs. 26.9% | No (group comparison) | Yes |
|  | Lower haemoglobin | Mean difference | 11.5 ± 1.4 vs. 12.6 ± 1.9 g/dL | No (group comparison) | Yes |
|  | Lower serum albumin | Mean difference | 3.3 ± 0.5 vs. 3.6 ± 0.6 g/dL | No (group comparison) | Yes |
|  | Higher baseline creatinine | Mean difference | 1.1 ± 0.3 vs. 0.9 ± 0.2 mg/dL | No (group comparison) | Yes |
|  | Diabetes mellitus | % occurrence | 25.0% vs. 12.7% | No (group comparison) | No |
|  | Lower HBsAg seropositivity | % occurrence | 58.3% vs. 79.2% | No (group comparison) | Yes |
|  | MELD score | Mean difference | 9.4 ± 4.1 vs. 8.0 ± 3.7 | No (group comparison) | Borderline |
|  | Amount of contrast agent | Mean difference | 139 ± 31 vs. 134 ± 28 mL | No (group comparison) | No |
|  | NSAID use | % occurrence | 12.5% vs. 18.9% | No (group comparison) | No |
|  | Platelet count | Mean difference | 123 ± 76 vs. 132 ± 75 ×10³/mL | No (group comparison) | No |
|  | AFP >400 ng/mL | % occurrence | 18.2% vs. 30.7% | No (group comparison) | No |
| Prasad (2014) [17] | Diabetes mellitus | % occurrence | 25% vs. 15% | No (group comparison) | Yes^c^ |
|  | Contrast ratio ≥2.9 | Association with CIN occurrence | Higher CIN incidence observed with contrast ratio ≥2.9 | No (group comparison) | Yes |
|  | Age | Mean difference | 59 vs. 53 years | No (group comparison) | No |
|  | Male sex | % occurrence | 50% vs. 32% | No (group comparison) | No |
|  | Baseline serum creatinine | Mean difference | 0.98 vs. 0.80 mg/dL | No (group comparison) | No |
|  | Hypertension | % occurrence | Similar between groups | No (group comparison) | No |
|  | Hyperlipidaemia | % occurrence | Similar between groups | No (group comparison) | No |
|  | Coronary artery disease | % occurrence | Similar between groups | No (group comparison) | No |
| Ray (2013) [16] | Age ≥75 years | % occurrence | 20.0% vs. 11.1% | No (group comparison) | Yes |
|  | Baseline renal impairment | % occurrence | 14.3% vs. 8.3% | No (group comparison) | No |
|  | Diabetes mellitus | % occurrence | 33.3% vs. 11.1% | No (group comparison) | No |
|  | Calculated MCD <200 mL | % occurrence | 33.3% vs. 2.9% | No (group comparison) | No |
|  | Hypertension and/or hyperlipidaemia | % occurrence | 100% vs. 81.9% | No (group comparison) | No |
|  | Use of CIN-predisposing drugs | % occurrence | 0% vs. 27.1% | No (group comparison) | No |
|  | Concurrent 3% hypertonic saline therapy | % occurrence | 0% vs. 37.5% | No (group comparison) | No |
|  | Multiple contrast-enhanced studies within 72 h | Clinical association | No CIN cases observed despite repeated contrast exposure | No (descriptive analysis) | No |
|  | Contrast volume exceeding calculated MCD | Clinical association | No CIN cases observed despite exceeding calculated MCD | No (descriptive analysis) | No |
|  | Smoking | % occurrence | Present in 37.3% of cohort | No (descriptive analysis) | No |
|  | ACE inhibitor or ARB use | % occurrence | 0 CIN cases among 13 exposed patients | No (descriptive analysis) | No |
|  | Metformin use | % occurrence | 0 CIN cases among exposed patients | No (descriptive analysis) | No |
|  | Elevated systolic blood pressure >160 mmHg | % occurrence | 1 CIN case among 16 patients | No (descriptive analysis) | No |
| Schwarz (2026) [15] | Known CKD | % occurrence | 60.7% vs. 38.1% | No (group comparison) | Yes |
|  | Arterial hypertension | % occurrence | 81.2% vs. 73.2% | No (group comparison) | Yes |
|  | Diabetes mellitus | % occurrence | 34.8% vs. 25.6% | No (group comparison) | Yes |
|  | Antiplatelet therapy | % occurrence | 36.8% vs. 27.2% | No (group comparison) | Yes |
|  | Baseline NIHSS score | Median difference | 16 vs. 15 | No (group comparison) | Yes |
|  | Admission blood glucose | Median difference | 131 vs. 123 mg/dL | No (group comparison) | Yes |
|  | Lower admission haemoglobin | Median difference | 13.0 vs. 13.3 g/dL | No (group comparison) | Yes |
|  | Lower baseline eGFR | Median difference | 65 vs. 77 mL/min/1.73 m² | No (group comparison) | Yes |
|  | No IV thrombolysis before EVT | % occurrence | 33.4% vs. 43.9% | No (group comparison) | Yes |
|  | EVT contrast volume | Median difference | 100 vs. 80 mL | No (group comparison) | Yes |
|  | Procedural hypotension | % occurrence | 17.4% vs. 12.0% | No (group comparison) | Yes |
|  | Male sex | β coefficient | 0.2617 | Yes (multivariable predictive model) | Yes |
|  | Non-White ethnicity | β coefficient | 0.4216 | Yes (multivariable predictive model) | Yes |
|  | Arterial hypertension | β coefficient | 0.3947 | Yes (multivariable predictive model) | Yes |
|  | Absence of dyslipidaemia | β coefficient | 0.3849 | Yes (multivariable predictive model) | Yes |
|  | Known CKD | β coefficient | 0.6535 | Yes (multivariable predictive model) | Yes |
|  | Antiplatelet therapy | β coefficient | 0.4025 | Yes (multivariable predictive model) | Yes |
|  | NIHSS score >10 | β coefficient | 0.4061 | Yes (multivariable predictive model) | Yes |
|  | Blood glucose 150–199 mg/dL | β coefficient | 0.4093 | Yes (multivariable predictive model) | Yes |
|  | Blood glucose ≥200 mg/dL | β coefficient | 0.8896 | Yes (multivariable predictive model) | Yes |
|  | Baseline eGFR <15 mL/min | β coefficient | 2.0878 | Yes (multivariable predictive model) | Yes |
|  | Admission haemoglobin <10 g/dL | β coefficient | 0.6698 | Yes (multivariable predictive model) | Yes |
|  | Mean arterial pressure ≥140 mmHg | β coefficient | 0.7527 | Yes (multivariable predictive model) | Yes |
|  | No IV thrombolysis | β coefficient | 0.3844 | Yes (multivariable predictive model) | Yes |
| Seki (2021) [14] | Baseline renal function (eGFR) | Correlation coefficient | r = 0.704 | No (correlation analysis) | Yes |
|  | Total iodine quantity | Correlation coefficient | r = −0.263 | No (correlation analysis) | No |
| Sharma (2013) [13] | Baseline creatinine ≥1.5 mg/dL / chronic renal insufficiency | % occurrence | 1 patient with chronic renal insufficiency developed CIN | No formal effect estimate reported | NR |
|  | Additional CT angiography before intervention | % occurrence | 0/44 patients developed CIN after additional CTA exposure | No formal effect estimate reported | NR |
| Si (2021) [12] | Hypertension | HR | 2.82 (1.11–7.19) | Yes (multivariable logistic regression) | Yes |
|  | Haemoglobin (per g/L increase) | HR | 0.95 (0.93–0.97) | Yes (multivariable logistic regression) | Yes |
|  | Serum creatinine | HR | 1.02 (1.00–1.05) | Yes (multivariable logistic regression) | Yes |
|  | Ascites | HR | 4.37 (0.87–21.82) | Yes (multivariable logistic regression) | Borderline |
|  | White blood cell count | HR | 1.28 (1.13–1.46) | No (univariable logistic regression) | Yes |
|  | Neutrophil count | HR | 1.12 (1.01–1.25) | No (univariable logistic regression) | Yes |
|  | Neutrophil-to-lymphocyte ratio | HR | 1.08 (1.02–1.13) | No (univariable logistic regression) | Yes |
|  | Platelet count | HR | 1.00 (1.00–1.00) | No (univariable logistic regression) | Yes |
|  | Blood urea nitrogen | HR | 1.07 (0.99–1.16) | No (univariable logistic regression) | Borderline |
|  | Iodized oil volume | HR | 1.06 (0.99–1.13) | No (univariable logistic regression) | Borderline |
|  | Glucose | HR | 1.12 (0.98–1.28) | No (univariable logistic regression) | Borderline |
|  | Albumin | HR | 0.95 (0.89–1.00) | No (univariable logistic regression) | Borderline |
|  | Low haemoglobin group after PSM | % occurrence | 14.6% vs. 3.4% CIN incidence | No (group comparison after PSM) | Yes |
|  | Haemoglobin after PSM | HR | 0.62 (0.52–0.74) | Yes (multivariable logistic regression after PSM) | Yes |
|  | Lymphocyte count after PSM | HR | 16.09 (2.38–108.55) | Yes (multivariable logistic regression after PSM) | Yes |
|  | Neutrophil-to-lymphocyte ratio after PSM | HR | 1.26 (1.00–1.59) | Yes (multivariable logistic regression after PSM) | Borderline |
|  | ROC-derived male Hb cutoff | ROC analysis | Hb cutoff 105.5 g/L; AUC 0.782 | Yes (ROC analysis) | Yes |
| Sohn (2020) [11] | Platelet count ≤100 ×10³/mm² | OR | 0.24 (0.07–0.79) | No (univariable logistic regression) | Yes |
|  | Serum AFP ≥100 ng/mL | OR | 3.45 (1.70–6.99) | No (univariable logistic regression) | Yes |
|  | BCLC stage C | OR | 15.91 (6.39–39.59) | No (univariable logistic regression) | Yes |
|  | Cisplatin-based chemoembolization (vs. doxorubicin) | OR | 4.88 (2.38–10.01) | No (univariable logistic regression) | Yes |
|  | Presence of ascites | OR | 12.12 (5.74–25.61) | No (univariable logistic regression) | Yes |
|  | ≥2 TACE sessions | OR | 2.09 (1.05–4.15) | No (univariable logistic regression) | Yes |
|  | Serum albumin ≤3.5 g/dL | OR | 1.96 (0.97–3.96) | No (univariable logistic regression) | Borderline |
|  | Total bilirubin ≥1.2 mg/dL | OR | 1.83 (0.91–3.70) | No (univariable logistic regression) | Borderline |
|  | BCLC stage C | OR | 10.52 (4.08–27.16) | Yes (multivariable logistic regression) | Yes |
|  | Presence of ascites | OR | 7.13 (3.17–16.04) | Yes (multivariable logistic regression) | Yes |
|  | Age ≥60 years | OR | 0.73 (0.37–1.45) | No (univariable logistic regression) | No |
|  | Male sex | OR | 2.23 (0.76–6.52) | No (univariable logistic regression) | No |
|  | Pre-TACE serum creatinine ≥1.0 mg/dL | OR | 0.91 (0.44–1.88) | No (univariable logistic regression) | No |
|  | MELD score ≥10 | OR | 1.08 (0.49–2.40) | No (univariable logistic regression) | No |
|  | Varices | OR | 0.80 (0.39–1.63) | No (univariable logistic regression) | No |
|  | Diabetes mellitus | OR | 1.05 (0.49–2.26) | No (univariable logistic regression) | No |
|  | Hypertension | OR | 1.06 (0.52–2.16) | No (univariable logistic regression) | No |
|  | Metformin use | OR | 0.67 (0.20–2.30) | No (univariable logistic regression) | No |
|  | Beta-blocker use | OR | 1.25 (0.54–2.88) | No (univariable logistic regression) | No |
|  | Diuretic use | OR | 0.51 (0.15–1.72) | No (univariable logistic regression) | No |
|  | ACE inhibitor/ARB use | OR | 1.25 (0.54–2.88) | No (univariable logistic regression) | No |
| Soliman (2020) [10] | Early excretory renal enhancement pattern | % occurrence | 55% vs. 2% | No (group comparison) | Yes |
|  | Late excretory renal enhancement pattern | % occurrence | 45% vs. 98% | No (group comparison) | Yes |
|  | Renal artery calcifications | % occurrence | 55% vs. 21% | No (group comparison) | Yes |
|  | Baseline serum creatinine | p value comparison | No significant difference between CIN and non-CIN groups | No (group comparison) | No |
|  | Total contrast volume administered | p value comparison | No significant difference between CIN and non-CIN groups | No (group comparison) | No |
|  | Procedure duration | p value comparison | No significant difference between CIN and non-CIN groups | No (group comparison) | No |
|  | Diabetes mellitus prevalence | p value comparison | No significant difference between CIN and non-CIN groups | No (group comparison) | No |
| Song (2013) [9] | Low serum albumin | Mean difference | 3.11 ± 0.38 vs. 3.75 ± 0.50 g/dL | No (group comparison) | Yes |
|  | Hypotension | % occurrence | 28.6% vs. 6.2% | No (group comparison) | Yes |
|  | Renal insufficiency | % occurrence | 28.6% vs. 2.5% | No (group comparison) | Yes |
|  | Serum albumin | OR | 0.14 (95% CI 0.03–0.76) | Yes (multivariable logistic regression) | Yes |
|  | Hypotension | OR | 2.17 (95% CI 0.20–23.54) | Yes (multivariable logistic regression) | No |
|  | Age | Mean difference | 65.29 ± 10.48 vs. 57.22 ± 14.91 years | No (group comparison) | No |
|  | Male sex | % occurrence | 71.4% vs. 75.3% | No (group comparison) | No |
|  | Contrast reuse within 3 days | % occurrence | 57.1% vs. 46.9% | No (group comparison) | No |
|  | Baseline serum creatinine | Mean difference | 1.04 ± 0.57 vs. 0.83 ± 0.36 mg/dL | No (group comparison) | No |
|  | Haematocrit | Mean difference | 30.93 ± 4.10 vs. 34.58 ± 5.96% | No (group comparison) | No |
|  | Haematocrit change | Mean difference | 13.22 ± 9.21 vs. 6.73 ± 17.14% | No (group comparison) | No |
|  | Contrast media volume | Mean difference | 232.86 ± 93.22 vs. 217.90 ± 68.61 mL | No (group comparison) | No |
|  | ACE inhibitor or ARB use | % occurrence | 14.3% vs. 8.6% | No (group comparison) | No |
|  | Diuretic use | % occurrence | 14.3% vs. 7.4% | No (group comparison) | No |
|  | NSAID use | % occurrence | 0% vs. 6.2% | No (group comparison) | No |
|  | Nephrotoxic antibiotic exposure | % occurrence | 28.6% vs. 19.8% | No (group comparison) | No |
|  | Diabetes mellitus | % occurrence | 14.3% vs. 13.6% | No (group comparison) | No |
| Tao (2017) [8] | Proteinuria | OR | 5.82 (2.98–11.36) | No (univariable logistic regression) | Yes |
|  | Proteinuria | OR | 5.74 (2.23–14.83) | Yes (multivariable logistic regression) | Yes |
|  | eGFR <60 mL/min/1.73 m² | OR | 1.67 (1.07–2.61) | No (univariable logistic regression) | Yes |
|  | eGFR <60 mL/min/1.73 m² | OR | 2.74 (1.48–5.10) | Yes (multivariable logistic regression) | Yes |
|  | Anemia | OR | 7.17 (4.47–11.51) | No (univariable logistic regression) | Yes |
|  | Anemia | OR | 6.82 (3.67–12.71) | Yes (multivariable logistic regression) | Yes |
|  | NIHSS score | OR | 1.21 (1.18–1.25) | No (univariable logistic regression) | Yes |
|  | NIHSS score | OR | 1.20 (1.16–1.24) | Yes (multivariable logistic regression) | Yes |
|  | Age >65 years | OR | 2.51 (1.57–4.02) | No (univariable logistic regression) | Yes |
|  | Baseline eGFR | Mean difference | 57.30 ± 22.46 vs. 64.82 ± 34.09 mL/min/1.73 m² | No (group comparison) | Yes |
|  | Baseline serum creatinine | Mean difference | 103.02 ± 35.88 vs. 97.05 ± 30.90 μmol/L | No (group comparison) | No |
|  | Diabetes mellitus | % occurrence | 18.8% vs. 13.7% | No (group comparison) | No |
|  | Hypertension | % occurrence | 31.8% vs. 30.1% | No (group comparison) | No |
|  | Contrast volume | Mean difference | 71.18 ± 27.05 vs. 69.11 ± 16.76 mL | No (group comparison) | No |
|  | Contrast exposure time >30 min | % occurrence | 42.4% vs. 42.3% | No (group comparison) | No |
|  | Endovascular therapy | % occurrence | 25.9% vs. 24.4% | No (group comparison) | No |
|  | Hyperuricemia | % occurrence | 60.0% vs. 56.9% | No (group comparison) | No |
|  | Hypercholesterolemia | % occurrence | 10.6% vs. 14.5% | No (group comparison) | No |
|  | Hypertriglyceridemia | % occurrence | 31.8% vs. 34.5% | No (group comparison) | No |
|  | Smoking | % occurrence | 9.4% vs. 7.5% | No (group comparison) | No |
| Xu (2025) [7] | Age >75 years | HR | 2.08 (1.55–3.21) | No (univariable Cox regression) | Yes |
|  | Age >75 years | HR | 3.84 (1.56–5.42) | Yes (multivariable Cox regression) | Yes |
|  | Diabetes mellitus | HR | 3.51 (1.45–3.65) | Yes (multivariable Cox regression) | Yes |
|  | CKD | HR | 7.66 (3.09–11.67) | No (univariable Cox regression) | Yes |
|  | CKD | HR | 4.86 (2.29–12.88) | Yes (multivariable Cox regression) | Yes |
|  | eGFR <30 mL/min/1.73 m² | HR | 22.45 (5.89–287.45) | No (univariable Cox regression) | Yes |
|  | eGFR <30 mL/min/1.73 m² | HR | 11.5 (3.02–43.72) | Yes (multivariable Cox regression) | Yes |
|  | Female sex | HR | 2.10 (1.54–3.12) | No (univariable Cox regression) | Yes |
|  | Contrast dose | HR | 1.35 (0.32–1.59) | No (univariable Cox regression) | No |
|  | Contrast dose | HR | 1.62 (0.54–1.41) | Yes (multivariable Cox regression) | No |
|  | ACE inhibitor/ARB use | HR | 1.58 (0.64–2.08) | No (univariable Cox regression) | Borderline |
|  | Chronic anemia | HR | 1.28 (0.82–1.53) | No (univariable Cox regression) | No |
|  | Hypertension | HR | 1.45 (0.68–2.85) | No (univariable Cox regression) | No |
|  | Coronary artery disease | HR | 1.01 (0.52–1.52) | No (univariable Cox regression) | No |
|  | Diuretic use | HR | HR 2.64 (95% CI 0.57–2.53)^*^ | No (univariable Cox regression) | No |
|  | NSAID use | HR | 1.48 (0.42–2.52) | No (univariable Cox regression) | No |
|  | Number of culprit arteries | HR | 2.62 (0.80–3.53) | No (univariable Cox regression) | No |
|  | IV fluid administration | HR | 0.50 (0.12–1.10) | No (univariable Cox regression) | No |
| Yoo (2020) [6] | Diabetes mellitus | OR | 2.34 (1.28–4.26) | Yes (multivariable logistic regression) | Yes |
|  | Contrast dose (per 10 mL increase) | OR | 1.10 (1.03–1.18) | Yes (multivariable logistic regression) | Yes |
|  | Unsuccessful reperfusion | OR | 1.90 (1.01–3.52) | Yes (multivariable logistic regression) | Yes |
|  | CTA before EVT | OR | 2.11 (0.78–7.40) | Yes (multivariable logistic regression) | No |
|  | Hypertension | OR | 1.97 (0.97–4.20) | Yes (multivariable logistic regression) | Borderline |
|  | NIHSS score on admission | OR | 1.04 (0.99–1.09) | Yes (multivariable logistic regression) | Borderline |
|  | Male sex | OR | 0.58 (0.31–1.05) | Yes (multivariable logistic regression) | Borderline |
|  | Diabetes mellitus | % occurrence | 47.5% vs. 25.6% | No (group comparison) | Yes |
|  | Hypertension | % occurrence | 76.3% vs. 62.0% | No (group comparison) | Yes |
|  | Statin use before admission | % occurrence | 13.6% vs. 4.8% | No (group comparison) | Yes |
|  | Contrast dose | Mean difference | 89.8 ± 42.9 vs. 69.1 ± 36.0 mL | No (group comparison) | Yes |
|  | Haemoglobin | Mean difference | 12.8 ± 2.1 vs. 13.5 ± 1.8 g/dL | No (group comparison) | Yes |
|  | Glucose | Mean difference | 8.9 ± 3.8 vs. 7.7 ± 3.0 mmol/L | No (group comparison) | Yes |
|  | NIHSS score on admission | Median difference | 19 (14.5–21.5) vs. 17 (13–21) | No (group comparison) | Yes |
|  | ASPECTS | Median difference | 5 (3–8) vs. 7 (5–9) | No (group comparison) | Yes |
|  | Unsuccessful reperfusion (mTICI ≤2a) | % occurrence | 39.0% vs. 23.2% | No (group comparison) | Yes |
|  | Any haemorrhagic transformation | % occurrence | 55.9% vs. 30.8% | No (group comparison) | Yes |
|  | Parenchymal hematoma | % occurrence | 27.1% vs. 13.0% | No (group comparison) | Yes |
|  | Baseline eGFR <30 mL/min/1.73 m² | % occurrence | 15.3% vs. 5.7% | No (group comparison) | Borderline |
|  | White blood cell count | Mean difference | 9.8 ± 4.2 vs. 8.7 ± 3.4 ×10⁹/L | No (group comparison) | Borderline |
| Zhou (2014) [5] | Child–Pugh class B | % occurrence | 20.8% vs. 6.3% (Child–Pugh A) | No (group comparison) | Yes |

Abbreviations: ACE, angiotensin-converting enzyme; AFP, alpha-fetoprotein; AJCC, American Joint Committee on Cancer; AKI, acute kidney injury; AKIN, Acute Kidney Injury Network; ALBI, albumin–bilirubin; ARB, angiotensin II receptor blocker; ARF, acute renal failure; ASPECTS, Alberta Stroke Program Early CT Score; AST, aspartate aminotransferase; AUC, area under the curve; BAE, bronchial artery embolization; BCLC, Barcelona Clinic Liver Cancer staging system; CA-AKI, contrast-associated acute kidney injury; CAS, carotid artery stenosis; CG-GFR, Cockcroft–Gault glomerular filtration rate; CI, confidence interval; CIN, contrast-induced nephropathy; CKD, chronic kidney disease; CT, computed tomography; CTA, computed tomography angiography; cTACE, conventional transarterial chemoembolization; DEMs, drug-eluting microspheres; DSA, digital subtraction angiography; eGFR, estimated glomerular filtration rate; EVT, endovascular thrombectomy; GCS, Glasgow Coma Scale; GEE, generalized estimating equation; GFR, glomerular filtration rate; HbA1c, glycated haemoglobin; HBsAg, hepatitis B surface antigen; HCC, hepatocellular carcinoma; HCV-Ab, hepatitis C virus antibody; HR, hazard ratio; hs-CRP, high-sensitivity C-reactive protein; ICU, intensive care unit; IgA, immunoglobulin A; IV, intravenous; MACD, maximum allowable contrast dose; MCD, maximum contrast dose; MDRD, Modification of Diet in Renal Disease; MELD, Model for End-Stage Liver Disease; mTICI, modified thrombolysis in cerebral infarction; NIHSS, National Institutes of Health Stroke Scale; NSAID, nonsteroidal anti-inflammatory drug; OR, odds ratio; PC-AKI, post-contrast acute kidney injury; PT-INR, prothrombin time–international normalized ratio; PSM, propensity score matching; ROC, receiver operating characteristic; TACE, transcatheter arterial chemoembolization; TICI, thrombolysis in cerebral infarction; uNGAL, urine neutrophil gelatinase-associated lipocalin.

^*^Effect estimate and 95% CI are reported as presented in the original publication; the point estimate lies outside the reported confidence interval, suggesting a reporting error in the source study.

^a^ For some variables in Mitaka (2023), only p-values from simple linear regression analyses were reported because standardized effect estimates were not provided in the original study.

^b^ Medication use after contrast exposure included nonsteroidal anti-inflammatory drugs (NSAIDs), ACE inhibitors, ARBs, β blockers, statins, and insulin.

^c^ The manuscript reported diabetes prevalence of 25% versus 15% with p<0.001; however, the reported confidence interval appears inconsistent with the very small number of CIN cases (n = 4), likely reflecting statistical instability related to the limited event numbers in this retrospective cohort.

Note: Effect estimates were extracted as reported by the original studies and may include descriptive comparisons, univariable analyses, multivariable regression models, ROC analyses, or correlation analyses. Adjusted analyses refer to multivariable models controlling for potential confounders. Statistical significance classifications were based on the original study-reported thresholds, confidence intervals, or p-values, and were categorized as Yes, No, Borderline, or NR where appropriate.

Supplementary Table S5. Clinical outcomes associated with contrast-associated acute kidney injury following interventional radiology procedures.

| Study (Author, Year) | Clinical Outcome | Outcome Timeframe | Association with CA-AKI | Effect Measure | Effect Estimate (95% CI) | Adjusted Analysis | Statistically Significant |
| --- | --- | --- | --- | --- | --- | --- | --- |
| Aoe (2019) [43] | Requirement for haemodialysis | Post-TACE | One patient with CIN required transient haemodialysis and recovered | NR | NR | No | NR |
|  | Sustained renal dysfunction | Post-TACE follow-up | Sustained elevated creatinine observed in 2 patients with CIN | NR | NR | No | NR |
|  | Renal recovery | Post-TACE follow-up | Renal function recovered in all but 2 patients | NR | NR | No | NR |
| Cao (2021) [42] | ARF requiring emergency dialysis | Post-TACE | One patient with CIN developed ARF and recovered renal function after emergency dialysis | NR | NR | No | NR |
|  | Mortality | Post-TACE | One patient with CIN died following ARF after TACE | NR | NR | No | NR |
|  | Renal recovery | Post-TACE follow-up | Renal function recovered after emergency dialysis in one patient with ARF | NR | NR | No | NR |
| Cho (2011) [41] | In-hospital mortality | During hospitalisation after TACE | In-hospital mortality was substantially higher in patients with RCIN compared with no-RCIN | % mortality | 30.0% vs. 1.5% | No (group comparison) | Yes |
|  | In-hospital mortality | During hospitalisation after TACE | Occurrence of RCIN independently associated with in-hospital mortality after TACE | OR | 26.7 (CI NR) | Yes (multivariable logistic regression) | Yes |
|  | In-hospital mortality | During hospitalisation after TACE | Higher Child–Pugh score independently associated with in-hospital mortality after TACE | OR | 2.8 (CI NR) | Yes (multivariable logistic regression) | Yes |
| Cohen (2014) [40] | AKI | Post-TACE follow-up | AKI occurred in 43/299 patients with available adverse-event data | % occurrence | 14.4% | No (descriptive analysis) | NR |
|  | Deterioration in liver function | Post-TACE follow-up | Deterioration in liver function occurred in 122/299 patients with available adverse-event data | % occurrence | 41.0% | No (descriptive analysis) | NR |
|  | Vascular complications | Post-TACE follow-up | Rare vascular complications following TACE | % occurrence | 1.3% | No (descriptive analysis) | NR |
|  | Significant haemorrhage requiring transfusion | Post-TACE follow-up | Significant haemorrhage requiring ≥2 blood-unit transfusion | % occurrence | 5.0% | No (descriptive analysis) | NR |
| Delgado Acosta (2020) [39] | Dialysis requirement | During hospitalisation/post-procedure follow-up | No patient with CIN required dialysis | % occurrence | 0% | No (descriptive analysis) | NR |
|  | Poor 90-day functional outcome | 90-day follow-up | CIN independently associated with worse 90-day mRS outcome | p value | p = 0.0307 | Yes (multivariable logistic regression) | Yes |
|  | Late renal failure | Late follow-up | No significant association between CIN and late renal failure | p value | p = 0.9979 | No | No |
|  | Hospital pneumonia | During hospitalisation | No significant association between CIN and hospital pneumonia | p value | p = 0.5332 | No | No |
|  | Urinary tract infection | During hospitalisation | No significant association between CIN and urinary tract infection | p value | p = 0.3333 | No | No |
| Diprose (2019) [38] | RRT | During hospitalisation | No patients with CA-AKI required RRT | % occurrence | 0% | No (descriptive analysis) | NR |
|  | Mortality | 3-month follow-up | Four of 11 patients with CA-AKI died by 3 months | % mortality | 36.4% | No (descriptive analysis) | NR |
|  | Mortality | 3-month follow-up | CA-AKI independently associated with increased 3-month mortality | OR | 4.68 (1.05–20.97) | Yes (multivariable logistic regression) | Yes |
|  | Mortality | 3-month follow-up | Coronary artery disease independently associated with increased mortality | OR | 2.69 (1.13–6.45) | Yes (multivariable logistic regression) | Yes |
|  | Mortality | 3-month follow-up | Higher baseline NIHSS independently associated with increased mortality | OR | 1.07 (1.02–1.13) | Yes (multivariable logistic regression) | Yes |
|  | Mortality | 3-month follow-up | Intravenous thrombolysis (IV tPA) associated with lower mortality | OR | 0.40 (0.18–0.89) | Yes (multivariable logistic regression) | Yes |
|  | Mortality | 3-month follow-up | Anterior circulation occlusion associated with lower mortality | OR | 0.36 (0.15–0.84) | Yes (multivariable logistic regression) | Yes |
| Hao (2014) [37] | Mortality | During hospitalisation | Post-TACE AKI associated with increased inpatient mortality according to Barrett and Parfrey criteria | % mortality | 75.0% vs. 25.0% | No (group comparison) | Yes |
|  | Mortality | During hospitalisation | Post-TACE AKI associated with increased inpatient mortality according to AKIN criteria | % mortality | 75.0% vs. 25.0% | No (group comparison) | Yes |
|  | Chronic renal insufficiency | Post-TACE follow-up | Three patients with AKIN-defined AKI developed chronic renal insufficiency | % occurrence | 12.5% | No (descriptive analysis) | Yes |
|  | CVVH | During hospitalisation/post-TACE follow-up | Two patients with AKIN-defined AKI required CVVH | % occurrence | 8.33% | No (descriptive analysis) | Yes |
|  | Hospitalisation cost | During hospitalisation | Total hospitalisation cost significantly higher in patients with AKI according to Barrett and Parfrey criteria | Cost comparison | ¥24,902 vs. ¥23,339 | No (group comparison) | Yes |
|  | Hospital stay | During hospitalisation | No significant difference in hospital stay between AKI and non-AKI groups | Median days | 11 vs. 12 days | No (group comparison) | No |
| Hayakawa (2014) [36] | Haemodialysis requirement | During hospitalisation/post-TACE follow-up | No patients with CIN required haemodialysis | % occurrence | 0% | No (descriptive analysis) | NR |
|  | ARF symptoms | During hospitalisation | No patients developed clinical signs or symptoms of ARF | % occurrence | 0% | No (descriptive analysis) | NR |
|  | Renal recovery | Within 7 days after TACE | All patients with CIN showed normalization of serum creatinine within 7 days | % recovery | 100% | No (descriptive analysis) | NR |
|  | In-hospital mortality | During hospitalisation | One patient with CIN died from HCC rupture on postoperative day 8 | % mortality | 12.5% | No (descriptive analysis) | NR |
|  | In-hospital mortality | During hospitalisation | Two patients without CIN died from liver failure unrelated to TACE | % mortality | 0.67% | No (descriptive analysis) | NR |
|  | In-hospital mortality association | During hospitalisation | Higher mortality observed in patients with CIN compared with no CIN | % mortality comparison | 12.5% vs. 0.67% | No (group comparison) | Borderline |
|  | Congestive heart failure | During hospitalisation/post-TACE follow-up | One patient developed overhydration-related congestive heart failure and recovered with medical treatment | % occurrence | 0.33% | No (descriptive analysis) | NR |
| Hayashi (2020) [35] | Overall survival | Follow-up after initial TACE/TAI | Development of AKI was associated with poor prognosis | HR | 3.18 (1.41–7.17) | Yes (multivariable Cox proportional hazards model) | Yes |
|  | Overall survival | Follow-up after initial TACE/TAI | Patients with AKI had significantly lower survival rates than patients without AKI | Log-rank test | p = 0.034 | No (Kaplan–Meier analysis) | Yes |
|  | Persistent serum creatinine elevation | 30-day follow-up | Serum creatinine remained significantly elevated in AKI patients at day 30 | Median serum creatinine change | +0.11 mg/dL vs. +0.01 mg/dL | No (group comparison) | Yes |
|  | Persistent reduction in eGFR | 30-day follow-up | eGFR remained significantly lower in AKI patients at day 30 | Median eGFR change | −12 vs. −2 mL/min/1.73 m² | No (group comparison) | Yes |
|  | AKI severity | Within 7 days after TACE/TAI | All AKI cases were stage 1 AKI | % occurrence | 100% stage 1 AKI | No (descriptive analysis) | NR |
| Hsu (2010) [34] | Overall survival | Mean follow-up 19 ± 17 months | Patients with ARF had significantly decreased survival compared with patients without ARF | Log-rank test | p = 0.029 | No (Kaplan–Meier analysis) | Yes |
|  | Mortality risk | Mean follow-up 19 ± 17 months | Post-TACE ARF independently associated with increased mortality in ascitic HCC patients | RR | 2.17 (1.05–4.48) | Yes (multivariable Cox proportional hazards model) | Yes |
|  | Persistent renal dysfunction | During hospitalisation/discharge follow-up | Four patients with ARF developed persistent renal insufficiency until discharge or death | % occurrence | 36.4% | No (descriptive analysis) | NR |
|  | Renal recovery | During hospitalisation/follow-up | Seven patients recovered renal function to baseline serum creatinine levels | % recovery | 63.6% | No (descriptive analysis) | NR |
|  | Haemodialysis requirement | During hospitalisation | No patients required haemodialysis for post-TACE ARF | % occurrence | 0% | No (descriptive analysis) | NR |
|  | Early post-TACE mortality | Within 1 month after TACE | Two patients with ARF died within 1 month because of liver and renal failure | % mortality | 18.2% | No (descriptive analysis) | NR |
| Huo (2004) [33] | Irreversible renal impairment | During hospitalisation/follow-up | Four patients with ARF developed irreversible renal impairment | % occurrence | 33.3% | No (descriptive analysis) | NR |
|  | Reversible renal recovery | During hospitalisation/follow-up | Eight patients with ARF recovered renal function to baseline levels | % recovery | 66.7% | No (descriptive analysis) | NR |
|  | Hepatorenal syndrome | Within 5 weeks after TACE | One patient with irreversible ARF progressed to hepatorenal syndrome | % occurrence | 8.3% | No (descriptive analysis) | NR |
|  | Mortality | Within 5 weeks after TACE | One patient with hepatorenal syndrome died after TACE | % mortality | 8.3% | No (descriptive analysis) | NR |
|  | Persistent renal dysfunction | 6–11 weeks after TACE | Serum creatinine remained elevated in three patients with irreversible ARF | Duration of dysfunction | 6–11 weeks | No (descriptive analysis) | NR |
|  | Post-TACE serum creatinine elevation | Days 3–9 after TACE | Patients with ARF showed significantly higher serum creatinine levels after TACE than patients without ARF | Mean serum creatinine | Day 3: 1.9 ± 0.2 vs. 1.1 ± 0.2 mg/dL | No (group comparison) | Yes |
|  | Post-TACE serum creatinine elevation | Days 3–9 after TACE | Patients with ARF showed persistently higher creatinine levels at day 6 | Mean serum creatinine | Day 6: 1.7 ± 0.4 vs. 1.0 ± 0.2 mg/dL | No (group comparison) | Yes |
|  | Post-TACE serum creatinine elevation | Days 3–9 after TACE | Elevated creatinine persisted at day 9 in ARF patients | Mean serum creatinine | Day 9: 1.5 ± 0.7 vs. 1.1 ± 0.2 mg/dL | No (group comparison) | Borderline |
| Huo (2004) [32] | Overall mortality | Mean follow-up 33 ± 18 months | Patients with prolonged ARF had significantly higher mortality than patients with transient ARF | % mortality | 63% vs. 28% | No (group comparison) | Yes |
|  | Overall survival | Long-term follow-up | Prolonged ARF independently predicted decreased survival | RR | 2.3 (1.4–4.0) | Yes (multivariable Cox proportional hazards model) | Yes |
|  | Overall survival among ARF patients | Mean follow-up 17 ± 11 months after ARF | Prolonged ARF independently predicted decreased survival among patients with ARF | RR | 3.5 (1.3–9.3) | Yes (multivariable Cox proportional hazards model) | Yes |
|  | One-year survival | 1-year follow-up after ARF | Patients with prolonged ARF had substantially lower survival than transient ARF patients | % survival | 58% vs. 85% | No (Kaplan–Meier analysis) | Yes |
|  | Two-year survival | 2-year follow-up after ARF | Patients with prolonged ARF had substantially lower survival than transient ARF patients | % survival | 25% vs. 71% | No (Kaplan–Meier analysis) | Yes |
|  | Prolonged renal impairment | During hospitalisation/follow-up | Twenty-seven patients developed prolonged ARF | % occurrence | 27/56 ARF patients | No (descriptive analysis) | NR |
|  | Transient renal recovery | During hospitalisation/follow-up | Twenty-nine patients recovered renal function during hospitalisation | % recovery | 29/56 ARF patients | No (descriptive analysis) | NR |
|  | Renal failure–related mortality | Long-term follow-up | Patients with prolonged ARF more frequently died from renal failure than transient ARF patients | % mortality | 15% vs. 0% | No (group comparison) | Yes |
|  | Hepatorenal syndrome | Within 3 months after TACE | Two patients with prolonged ARF developed hepatorenal syndrome | % occurrence | 2/27 patients | No (descriptive analysis) | NR |
|  | Persistent serum creatinine elevation | Approximately 20 days after TACE | Serum creatinine remained elevated in prolonged ARF patients | Mean serum creatinine | 2.6 ± 0.8 mg/dL | No (descriptive analysis) | Yes |
| Jia (2019) [31] | AKI incidence | 48 h after CTA | Consecutive multidose contrast exposure (CTA + DSA) showed numerically higher AKI incidence than CTA alone | % occurrence | 7.4% vs. 2.3% | No (group comparison) | No |
|  | Serum creatinine increase in AKI patients | 48 h after CTA | Patients in the CTA + DSA group with AKI demonstrated substantially greater creatinine elevation than CTA-only patients | Mean creatinine increase | 1.71 ± 2.42 mg/dL vs. 0.24 ± 0.06 mg/dL | No (group comparison) | NR |
|  | Relative creatinine increase in AKI patients | 48 h after CTA | Relative serum creatinine increase was greater in CTA + DSA patients with AKI | % creatinine increase | 131.6% ± 160.2% vs. 31.0% ± 6.9% | No (group comparison) | NR |
|  | Functional outcome | 3-month follow-up | No significant difference in favourable 3-month functional outcomes between groups | mRS ≤3 | CTA: 97.7% vs. CTA+DSA: 91.5% | No (group comparison) | No |
| Joo (2019) [30] | Renal recovery | Within 1 week after coiling/follow-up | All patients with CIN returned to baseline creatinine levels | % recovery | 100% | No (descriptive analysis) | NR |
|  | Pulmonary oedema | During hospitalisation | One patient with CIN developed pulmonary oedema requiring continuous furosemide infusion | % occurrence | 7.7% of CIN patients | No (descriptive analysis) | NR |
|  | Haemodialysis requirement | During hospitalisation/follow-up | No patients required haemodialysis | % occurrence | 0% | No (descriptive analysis) | NR |
|  | In-hospital mortality | During hospitalisation | No in-hospital deaths occurred among patients undergoing elective aneurysm coiling | % mortality | 0% | No (descriptive analysis) | NR |
|  | Grade 1 CIN severity | 48–72 h after contrast exposure | Most CIN cases were mild grade 1 nephropathy | % occurrence | 11/13 patients (84.6%) | No (descriptive analysis) | NR |
|  | Grade 2 CIN severity | 48–72 h after contrast exposure | Severe CIN (grade 2) occurred infrequently | % occurrence | 2/13 patients (15.4%) | No (descriptive analysis) | NR |
|  | CIN incidence in patients with cumulative contrast dose ≥250 mL | 48–72 h after contrast exposure | Higher CIN incidence observed with cumulative contrast exposure ≥250 mL | % occurrence | 8.6% (5/58 patients) | No (group comparison) | No |
|  | CIN incidence with contrast volume exceeding MACD | 48–72 h after contrast exposure | Patients exceeding MACD had numerically higher CIN incidence | % occurrence | 15.4% vs. 2.8% | No (group comparison) | Borderline |
| Kim (2018) [29] | CIN occurrence | 48–72 h after neuroendovascular procedure | Only one high-risk CKD patient developed CIN after neuroendovascular procedure | % occurrence | 0.54% (1/184 patients) | No (descriptive analysis) | NR |
|  | Dialysis requirement | During hospitalisation/post-procedure follow-up | Patient who developed CIN did not require dialysis | % occurrence | 0% dialysis requirement among CIN cases | No (descriptive analysis) | NR |
|  | Post-procedure renal function after diagnostic angiography | 48–72 h after procedure | Mean renal function improved slightly after diagnostic cerebral angiography | Mean eGFR change | +5.7 mL/min/1.73 m² | No (descriptive analysis) | NR |
|  | Post-procedure serum creatinine after diagnostic angiography | 48–72 h after procedure | Mean serum creatinine decreased after diagnostic angiography | Mean creatinine change | −0.2 mg/dL (−12.5%) | No (descriptive analysis) | NR |
|  | Post-procedure renal function after therapeutic neuroendovascular intervention | 48–72 h after procedure | Mean renal function improved slightly after therapeutic intervention | Mean eGFR change | +7.1 mL/min/1.73 m² | No (descriptive analysis) | NR |
|  | Post-procedure serum creatinine after therapeutic neuroendovascular intervention | 48–72 h after procedure | Mean serum creatinine decreased after therapeutic intervention | Mean creatinine change | −0.19 mg/dL (−11.5%) | No (descriptive analysis) | NR |
|  | Renal transplantation status | Long-term follow-up | Patient who developed CIN remained on renal transplant waiting list | Clinical status | Persistent advanced CKD with ongoing renal transplant evaluation | No (descriptive analysis) | NR |
| Kim (2020) [28] | Renal recovery | Within 2–7 days after neurointervention | All patients with CIN recovered baseline creatinine levels with supportive care | % recovery | 100% | No (descriptive analysis) | NR |
|  | Dialysis requirement | During hospitalisation/follow-up | No patients required dialysis after CIN | % occurrence | 0% | No (descriptive analysis) | NR |
|  | Permanent renal sequelae | Follow-up period | No patients developed permanent renal sequelae after CIN | % occurrence | 0% | No (descriptive analysis) | NR |
|  | Vascular events in CIN group | 3-year follow-up | No cerebrovascular or cardiovascular events occurred in the CIN group | % occurrence | 0% | No (descriptive analysis) | NR |
|  | Vascular events in non-CIN group | 3-year follow-up | Three vascular events occurred in the non-CIN group | % occurrence | 3/159 patients (1.9%) | No (descriptive analysis) | NR |
|  | Myocardial infarction | 3-year follow-up | One patient in the non-CIN group developed myocardial infarction | % occurrence | 0.6% | No (descriptive analysis) | NR |
|  | Ischemic stroke | 3-year follow-up | Two patients in the non-CIN group developed ischemic stroke | % occurrence | 1.3% | No (descriptive analysis) | NR |
|  | Serum creatinine increase | 24–48 h after neurointervention | Serum creatinine increased after neurointervention in the CIN group | Temporal trend | Serum creatinine increased within 24–48 h after neurointervention | No (descriptive analysis) | Yes |
|  | Association between CIN and long-term cerebrovascular events | 3-year follow-up | CIN was not associated with additional cerebrovascular events during long-term follow-up | Clinical association | No association observed | No | No |
| Laible (2021) [27] | In-hospital mortality | During acute hospital stay | PC-AKI was associated with increased in-hospital mortality after EVT | OR | 2.87 (1.16–7.13) | Yes (multivariable logistic regression) | Yes |
|  | In-hospital mortality rate | During hospitalisation | Higher mortality observed in patients with PC-AKI compared with non-PC-AKI patients | % mortality | 24.1% vs. 13.9% | No (group comparison) | No |
|  | 3-month mortality | 3-month follow-up | PC-AKI independently associated with increased 3-month mortality after hospital discharge | OR | 3.70 (1.19–11.53) | Yes (multivariable logistic regression) | Yes |
|  | 3-month mortality rate | 3-month follow-up | Higher 3-month mortality observed in patients with PC-AKI | % mortality | 36.4% vs. 14.2% | No (group comparison) | Yes |
|  | Functional dependence (mRS >2) | 3-month follow-up | No significant association between PC-AKI and unfavourable functional outcome | OR | 1.57 (0.52–4.72) | Yes (multivariable logistic regression) | No |
|  | Functional dependence (mRS >1) | 3-month follow-up | No significant association between PC-AKI and unfavourable functional outcome | OR | 1.64 (0.47–5.77) | Yes (multivariable logistic regression) | No |
|  | Early neurological deterioration | During hospitalisation | PC-AKI not associated with early neurological deterioration | OR | 1.34 (0.45–4.02) | Yes (multivariable logistic regression) | No |
|  | Symptomatic intracerebral haemorrhage | During hospitalisation | PC-AKI not associated with symptomatic intracerebral haemorrhage | OR | 1.50 (0.34–6.56) | Yes (multivariable logistic regression) | No |
|  | In-hospital mortality in patients without baseline renal impairment | During hospitalisation | PC-AKI independently associated with increased in-hospital mortality even in patients without CKD | OR | 3.63 (1.35–9.74) | Yes (multivariable logistic regression) | Yes |
|  | 3-month mortality in patients without baseline renal impairment | 3-month follow-up | PC-AKI not independently associated with 3-month mortality after excluding baseline CKD | OR | 2.38 (0.63–8.99) | Yes (multivariable logistic regression) | No |
|  | In-hospital mortality in anterior circulation stroke | During hospitalisation | PC-AKI not independently associated with in-hospital mortality in anterior circulation stroke subgroup | OR | 2.26 (0.82–6.20) | Yes (multivariable logistic regression) | No |
|  | 3-month mortality in anterior circulation stroke | 3-month follow-up | PC-AKI independently associated with increased 3-month mortality in anterior circulation stroke subgroup | OR | 2.51 (1.67–3.78) | Yes (multivariable logistic regression) | Yes |
| Lee (2017) [26] | AKI occurrence after cTACE | Longitudinal follow-up | cTACE independently associated with increased AKI risk compared with non-cTACE HCC controls | HR | 1.66 (1.17–2.34) | Yes (multivariable Cox proportional hazards model) | Yes |
|  | Cumulative incidence of AKI | Longitudinal follow-up | Higher cumulative AKI incidence observed in cTACE patients | Log-rank test | p = 0.003 | No (Kaplan–Meier analysis) | Yes |
|  | AKI incidence rate | Longitudinal follow-up | AKI incidence rate higher in cTACE group than non-cTACE group | Incidence rate | 22.1 vs. 13.2 per 1,000 person-years | No (incidence comparison) | Yes |
|  | AKI incidence in cTACE cohort | Mean follow-up 2.88 ± 2.95 years | AKI developed in cTACE patients during study period | % occurrence | 72/1132 patients (6.4%) | No (descriptive cohort analysis) | NR |
|  | AKI incidence in non-cTACE cohort | Mean follow-up 4.14 ± 3.52 years | AKI developed in matched non-cTACE controls | % occurrence | 62/1132 patients (5.5%) | No (descriptive cohort analysis) | NR |
| Lee (2018) [25] | One-year mortality | 1-year follow-up after coil embolization | CI-AKI was associated with increased one-year mortality | OR | 16.85 (3.43–82.66) | Yes (multivariable logistic regression) | Yes |
|  | One-year mortality rate | 1-year follow-up | Higher mortality observed in patients with CI-AKI | % mortality | 37.5% vs. 4.5% | No (group comparison) | Yes |
|  | Poor long-term functional outcome (mRS ≥3) | 1-year follow-up | CI-AKI independently associated with poor long-term functional outcome | OR | 11.28 (2.13–59.52) | Yes (multivariable logistic regression) | Yes |
|  | Poor long-term functional outcome (mRS ≥3) | 1-year follow-up | Higher frequency of poor functional outcome observed in CI-AKI patients | % occurrence | 20.4% vs. 2.8% | No (group comparison) | Yes |
|  | Severe neurological status (GCS ≤8) and mortality | 1-year follow-up | Severe initial neurological status independently associated with increased mortality | OR | 5.56 (1.70–18.18) | Yes (multivariable logistic regression) | Yes |
|  | Severe neurological status (GCS ≤8) and poor functional outcome | 1-year follow-up | Severe initial neurological status independently associated with poor long-term outcome | OR | 31.02 (10.66–90.18) | Yes (multivariable logistic regression) | Yes |
|  | Older age (≥65 years) and poor functional outcome | 1-year follow-up | Older age independently associated with poor functional outcome | OR | 7.92 (2.97–21.07) | Yes (multivariable logistic regression) | Yes |
|  | Posterior circulation ruptured aneurysm and poor functional outcome | 1-year follow-up | Posterior circulation aneurysm independently associated with poor long-term outcome | OR | 4.27 (1.31–13.92) | Yes (multivariable logistic regression) | Yes |
|  | Hypertension treatment history and mortality | 1-year follow-up | Prior antihypertensive medication use inversely associated with mortality | OR | 0.12 (0.01–0.78) | Yes (multivariable logistic regression) | Yes |
| Lin (2025) [24] | Renal-function improvement at discharge in CKD patients | At hospital discharge | Most CKD patients demonstrated improved renal function after TACE | % improvement | 73.15% | No (serial renal-function comparison) | Yes |
|  | Renal-function improvement at discharge in non-CKD patients | At hospital discharge | Most non-CKD patients demonstrated improved renal function after TACE | % improvement | 63.69% | No (serial renal-function comparison) | Yes |
|  | Renal-function worsening at discharge in CKD patients | At hospital discharge | Renal-function exacerbation occurred in minority of CKD patients | % occurrence | 24.07% | No (serial renal-function comparison) | NR |
|  | Renal-function worsening at discharge in non-CKD patients | At hospital discharge | Renal-function exacerbation occurred in non-CKD patients after TACE | % occurrence | 31.21% | No (serial renal-function comparison) | NR |
|  | Improved serum creatinine at day 3 after TACE in CKD patients | Third day after TACE | Significant early renal-function improvement observed after hydration and inpatient care | % improvement | 70.75% | No (serial creatinine comparison) | Yes |
|  | Improved serum creatinine at day 3 after TACE in non-CKD patients | Third day after TACE | Significant early creatinine improvement observed after TACE | % improvement | 58.71% | No (serial creatinine comparison) | Yes |
|  | Continued renal-function improvement after discharge in CKD patients | First outpatient follow-up | Renal function continued improving after discharge in some CKD patients | % improvement | 23/99 patients (23.2%) | No (serial renal-function comparison) | Yes |
|  | Continued renal-function improvement after discharge in non-CKD patients | First outpatient follow-up | Renal function continued improving after discharge in non-CKD patients | % improvement | 42/152 patients (27.6%) | No (serial renal-function comparison) | Yes |
|  | Mortality during hospitalisation | During admission | Two patients died during hospitalisation after TACE | % mortality | 0.75% (2/265 patients) | No (descriptive analysis) | NR |
|  | Grade ≥3 creatinine adverse events | During hospitalisation | No patients developed grade ≥3 creatinine adverse events | % occurrence | 0% | No (descriptive analysis) | NR |
|  | Acute myocardial infarction with cardiogenic shock | During hospitalisation | One patient died from myocardial infarction after DEMs-TACE | % occurrence | 0.38% | No (descriptive analysis) | NR |
|  | Oesophageal variceal rupture death | During hospitalisation | One patient died from variceal haemorrhage after DEMs-TACE | % occurrence | 0.38% | No (descriptive analysis) | NR |
|  | Renal-function worsening at first outpatient follow-up relative to discharge | First outpatient follow-up | Renal function worsened again after discharge in many patients despite discharge improvement | % worsening | 75.76% CKD; 69.08% non-CKD | No (serial renal-function comparison) | Yes |
| Mitaka (2023) [23] | Clinical CA-AKI incidence | POD 0–3 after neuroendovascular surgery | Very low incidence of creatinine-defined clinical CA-AKI after neuroendovascular surgery | % occurrence | 0.88% (2/228 patients) | No (descriptive analysis) | NR |
|  | Subclinical CA-AKI incidence | POD 0 and/or POD 1 | Subclinical CA-AKI incidence substantially exceeded clinical CA-AKI incidence when assessed using urine NGAL | % occurrence | 10.4% (7/67 patients) | No (descriptive analysis) | NR |
|  | Long-term CKD worsening | 1-year follow-up | One patient with subclinical CA-AKI developed worsening CKD 1 year later | Serum creatinine change | 1.84 → 3.05 mg/dL | No (descriptive longitudinal follow-up) | NR |
|  | Persistent long-term renal dysfunction | Long-term follow-up | No additional patients demonstrated persistent renal dysfunction after neuroendovascular surgery | % occurrence | Rare/none observed beyond single CKD progression case | No (descriptive analysis) | NR |
| Murakami (2016) [22] | Dialysis requirement | During hospitalisation/post-TACE follow-up | No patients with CIN required dialysis after TACE | % occurrence | 0% | No (descriptive analysis) | NR |
|  | Mortality due to ARF | During hospitalisation/post-TACE follow-up | No patients died because of ARF after TACE | % occurrence | 0% | No (descriptive analysis) | NR |
| Myung (2020) [20] | CRRT requirement | During hospitalisation | Five patients with PC-AKI required CRRT | % occurrence | 5/57 PC-AKI patients (8.7%); 0.8% of total cohort | No (descriptive analysis) | NR |
|  | Renal recovery after CRRT | Within 4 days after CRRT | All patients receiving CRRT recovered renal function | % recovery | 100% recovery | No (descriptive analysis) | NR |
|  | Renal recovery without dialysis | Several days after PC-AKI | Remaining PC-AKI patients recovered renal function without dialysis | Clinical course | Renal recovery observed in non-dialysis PC-AKI patients | No (descriptive analysis) | NR |
|  | Fatal renal adverse events | During hospitalisation/follow-up | No fatal renal adverse effects documented despite PC-AKI occurrence | % occurrence | 0% fatal renal events | No (descriptive analysis) | NR |
|  | Severe PC-AKI requiring CRRT in eGFR <30 mL/min/1.73 m² group | During hospitalisation | Patients with severely impaired baseline renal function had highest PC-AKI incidence and CRRT need | % occurrence | 46.7% PC-AKI incidence in eGFR <30 group | No (group comparison) | Yes |
| Ozdogru (2025) [19] | Poor functional outcome at discharge | Hospital discharge | Patients with CI-AKI had significantly worse functional outcomes | Mean mRS score | 5.0 ± 1.7 vs. 2.8 ± 2.0 | No (group comparison) | Yes |
|  | Cerebral oedema | During hospitalisation/post-EVT imaging | Cerebral oedema occurred more frequently in patients with CI-AKI | % occurrence | 51.9% vs. 22.9% | Yes (multivariable logistic regression) | Yes |
|  | Haemorrhagic transformation | During hospitalisation/post-EVT imaging | Haemorrhagic transformation more common in CI-AKI patients | % occurrence | p<0.001 overall distribution difference | No (group comparison) | Yes |
|  | Severe parenchymal haematoma type II | During hospitalisation/post-EVT imaging | More severe haemorrhagic transformation observed in CI-AKI patients | % occurrence | 24.1% vs. 4.8% | No (group comparison) | Yes |
| Park (2008) [18] | Prolonged renal insufficiency | 1 month after discharge | Prolonged renal insufficiency developed after AKI episodes | % occurrence | 24.1% of AKI episodes | No (descriptive analysis) | NR |
|  | Complete renal recovery | Within 1 month after discharge | Most AKI episodes demonstrated complete renal recovery | % recovery | 75.9% of AKI episodes | No (descriptive analysis) | NR |
|  | Progression to chronic renal failure | ≥12-month follow-up | Persistent chronic renal failure developed in some patients with prolonged renal insufficiency | % occurrence | 4/5 patients with prolonged renal insufficiency | No (descriptive longitudinal follow-up) | NR |
|  | Recovery of renal function after prolonged AKI | 6-month follow-up | One patient recovered normal renal function after prolonged AKI | % recovery | 1/5 patients | No (descriptive analysis) | NR |
|  | Requirement for RRT | During hospitalisation/post-TACE follow-up | No patients with AKI required RRT | % occurrence | 0% | No (descriptive analysis) | NR |
|  | Long-term survival | Mean follow-up 21 ± 17 months | AKI after TACE did not significantly reduce long-term survival | Median survival | 14 vs. 17 months | No (Kaplan–Meier/log-rank analysis) | No |
|  | Overall mortality | Mean follow-up 21 ± 17 months | Mortality numerically higher in patients with AKI | % mortality | 79.2% vs. 74.5% | No (group comparison) | No |
|  | HCC rupture death | 1.5 months after TACE | One patient with prolonged renal insufficiency died from HCC rupture | % occurrence | 1 patient | No (descriptive analysis) | NR |
|  | Persistent renal dysfunction after prolonged AKI | 12-month follow-up | Persistent renal dysfunction remained in prolonged AKI patients | Mean serum creatinine | 1.9 ± 0.6 mg/dL | No (descriptive analysis) | NR |
| Prasad (2014) [17] | Dialysis requirement | During hospitalisation/post-procedure follow-up | No patients with CIN required dialysis | % occurrence | 0% | No (descriptive analysis) | NR |
|  | Transient renal dysfunction | During hospitalisation/discharge follow-up | Most CIN cases demonstrated mild and transient creatinine elevation | Clinical course | Mild/transient serum creatinine increases | No (descriptive analysis) | NR |
|  | Persistent elevated creatinine at discharge | Hospital discharge | Two patients had persistent creatinine elevation at discharge despite improvement | Clinical course | Persistent but improving renal dysfunction | No (descriptive analysis) | NR |
|  | Mortality | During hospitalisation | One patient with ruptured cerebellar AVM and CIN died from unknown cause | % occurrence | 1 patient | No (descriptive analysis) | NR |
| Ray (2013) [16] | Renal recovery | Within 72 h after contrast study | All CIN cases resolved with hydration only | % recovery | 100% recovery within 72 h | No (descriptive analysis) | NR |
|  | Requirement for RRT/dialysis | During hospitalisation | No patients with CIN required RRT or dialysis | % occurrence | 0% | No (descriptive analysis) | NR |
|  | Recurrent AKI episodes | During hospitalisation/post-bleed days 7–15 | Two patients with initial CIN developed subsequent AKI episodes later during hospitalisation | % occurrence | 2/3 CIN patients | No (descriptive analysis) | NR |
|  | Possible structural renal disease identified after CIN | During hospitalisation/follow-up | One patient with recurrent AKI demonstrated possible polycystic kidney disease on renal ultrasound | Clinical finding | 1 patient | No (descriptive analysis) | NR |
|  | CIN with concurrent hypertonic saline therapy | During hospitalisation | No CIN cases occurred among patients receiving 3% hypertonic saline | % occurrence | 0% among hypertonic saline-treated patients | No (descriptive analysis) | No |
|  | CIN after repeated contrast exposure within 72 h | During hospitalisation | No CIN cases occurred despite repeated contrast-enhanced imaging within 72 h | % occurrence | 0% among repeatedly exposed patients | No (descriptive analysis) | No |
| Schwarz (2026) [15] | In-hospital mortality | During index hospitalisation | CA-AKI independently associated with increased in-hospital mortality | OR | 2.26 (1.61–3.19) | Yes (multivariable logistic regression) | Yes |
|  | Severe disability or death at 90 days | 90-day follow-up | CA-AKI independently associated with severe disability or death | OR | 1.53 (1.05–2.21) | Yes (multivariable logistic regression) | Yes |
|  | Length of hospitalisation | During hospitalisation | Longer hospitalisation observed in patients with CA-AKI | Median days | 9 vs. 8 days | No (group comparison) | Yes |
| Seki (2021) [14] | PC-AKI occurrence | 48–72 h after TAE and before discharge | No patients developed PC-AKI after emergency TAE for postpartum haemorrhage | % occurrence | 0% (0/47 patients) | No (descriptive analysis) | NR |
|  | Renal impairment on follow-up | Before discharge/follow-up renal assessment | No patients demonstrated renal impairment on follow-up examinations | % occurrence | 0% | No (descriptive analysis) | NR |
|  | Haemorrhage control | During hospitalisation | Postpartum haemorrhage successfully controlled in all patients after emergency TAE | Technical/clinical success rate | 100% | No (descriptive analysis) | NR |
|  | Hospital discharge | During hospitalisation | All patients were discharged after successful treatment | % occurrence | 100% | No (descriptive analysis) | NR |
|  | Repeat TAE requirement | During hospitalisation/follow-up | Four patients required repeat TAE procedures | % occurrence | 4/47 patients (8.5%) | No (descriptive analysis) | NR |
|  | Renal outcome after repeat TAE | Follow-up after repeat embolization | No renal impairment observed after repeat TAE procedures | % occurrence | 0% renal impairment | No (descriptive analysis) | NR |
|  | Change in serum creatinine after TAE | 48–72 h after TAE | Minimal postprocedural creatinine change observed overall | Mean creatinine change | 0.05 ± 0.11 mg/dL | No (correlation analysis) | NR |
|  | Post-discharge renal function | Before discharge | All patients had preserved renal function before discharge | eGFR category | 100% with eGFR >60 mL/min/1.73 m² | No (descriptive analysis) | NR |
| Sharma (2013) [13] | CIN occurrence | Within 48 h after EVT | Very low CIN incidence after emergency endovascular stroke treatment | % occurrence | 1.5% (3/191 patients) | No (descriptive analysis) | NR |
|  | CIN occurrence in patients with baseline creatinine >1.5 mg/dL | Within 48 h after EVT | One patient with chronic renal insufficiency developed CIN | % occurrence | 3.2% (1/31 patients) | No (descriptive analysis) | NR |
|  | CIN occurrence in patients with normal baseline creatinine | Within 48 h after EVT | Two patients with normal baseline creatinine developed CIN | % occurrence | 1.2% (2/163 patients) | No (descriptive analysis) | NR |
|  | CIN after additional CTA before EVT | Within 48 h after EVT | No CIN cases occurred despite additional CTA exposure before EVT | % occurrence | 0% (0/44 patients) | No (descriptive analysis) | NR |
|  | CIN in chronic haemodialysis patients | Within 48 h after EVT | No CIN-related complications observed in chronic haemodialysis patients | % occurrence | 0% (0/3 patients) | No (descriptive analysis) | NR |
|  | Requirement for dialysis/RRT | During hospitalisation/post-procedure follow-up | No new dialysis requirement reported among CIN patients | % occurrence | 0% | No (descriptive analysis) | NR |
|  | Mortality before follow-up creatinine assessment | During hospitalisation | Four patients died due to stroke severity before follow-up creatinine measurement | % occurrence | 4/194 patients | No (descriptive analysis) | NR |
| Si (2021) [12] | CIN occurrence after TACE | 48–96 h after TACE | CIN developed after TACE in patients with HCC | % occurrence | 5.6% (28/503 TACE sessions) | No (descriptive cohort analysis) | NR |
|  | CIN incidence in low haemoglobin group before PSM | 48–96 h after TACE | CIN incidence substantially higher in low-haemoglobin group before matching | % occurrence | 14.6% (16/110) vs. 3.1% (12/393) | No (group comparison) | Yes |
|  | CIN incidence in low haemoglobin group after PSM | 48–96 h after TACE | CIN incidence remained significantly higher in low-haemoglobin group after propensity score matching | % occurrence | 14.6% (16/110) vs. 3.4% (4/116) | No (group comparison after PSM) | Yes |
| Sohn (2020) [11] | Overall survival | Long-term follow-up after TACE | AKI was associated with markedly reduced overall survival after TACE | HR | AKI stage 1: 6.09 (2.54–14.57) | Yes (multivariable Cox regression) | Yes |
|  | Overall survival | Long-term follow-up after TACE | More severe AKI associated with progressively worse survival | HR | AKI stage 2: 8.51 (3.68–19.67) | Yes (multivariable Cox regression) | Yes |
|  | Overall survival | Long-term follow-up after TACE | Severe AKI strongly associated with mortality after TACE | HR | AKI stage 3: 17.64 (8.23–37.82) | Yes (multivariable Cox regression) | Yes |
|  | Overall mortality | During follow-up | Death occurred in HCC patients undergoing TACE | % mortality | 31.4% (109/347 patients) | No (descriptive cohort analysis) | NR |
|  | Overall survival according to AKI stage | 12–120 month follow-up | Survival decreased progressively with increasing AKI stage | Kaplan–Meier survival analysis | Non-AKI: 49.9% 120-month survival vs. 0% in AKI stage 2/3 | No (Kaplan–Meier analysis) | Yes |
|  | Survival in BCLC stage 0/A subgroup | Long-term follow-up | AKI stage remained strongly associated with poor survival even in earlier-stage HCC | Kaplan–Meier subgroup analysis | 0% survival in AKI stage 2/3 subgroup | No (subgroup survival analysis) | Yes |
|  | Survival in BCLC stage B/C subgroup | Long-term follow-up | AKI stage remained strongly associated with poor survival in advanced HCC | Kaplan–Meier subgroup analysis | 0% survival in AKI stage 1–3 subgroups over long-term follow-up | No (subgroup survival analysis) | Yes |
|  | Survival according to serum albumin | Long-term follow-up | AKI associated with poor survival regardless of baseline albumin level | Kaplan–Meier subgroup analysis | Significant survival separation by AKI stage | No (subgroup survival analysis) | Yes |
|  | Survival according to ascites status | Long-term follow-up | AKI associated with poor survival regardless of ascites status | Kaplan–Meier subgroup analysis | Significant survival separation by AKI stage | No (subgroup survival analysis) | Yes |
| Soliman (2020) [10] | CIN occurrence after HAE | Within 72 h after embolization | CIN developed following HAE procedures | % occurrence | 10.3% (11/106 procedures) | No (descriptive cohort analysis) | NR |
|  | Renal recovery before discharge | During hospitalisation | Patients hospitalized ≥2 days demonstrated normalization of serum creatinine before discharge | % recovery | 100% recovery among patients hospitalized ≥2 days | No (descriptive analysis) | NR |
|  | Requirement for haemodialysis or hemofiltration | During hospitalisation/post-embolization follow-up | No patients with CIN required dialysis or hemofiltration | % occurrence | 0% | No (descriptive analysis) | NR |
|  | Oliguria requiring nephrology consultation | During hospitalisation | One patient developed oliguria despite Foley catheter placement | % occurrence | 1 patient | No (descriptive analysis) | NR |
|  | Peak serum creatinine timing | Post-embolization hospitalisation | Peak serum creatinine typically occurred shortly after embolization | Median time | 2 days (IQR 1–2.5 days) | No (descriptive analysis) | NR |
|  | Persistent renal dysfunction at discharge | Hospital discharge | No persistent severe renal dysfunction requiring RRT reported | Clinical course | Conservative recovery observed overall | No (descriptive analysis) | NR |
| Song (2013) [9] | CIN occurrence after bronchial arteriography or BAE | Within 48–96 h after procedure | CIN developed following bronchial arteriography/BAE | % occurrence | 8.0% (7/88 patients) | No (descriptive cohort analysis) | NR |
|  | Renal recovery | Post-procedure follow-up | Most patients with CIN recovered to baseline creatinine levels | % recovery | 71.4% (5/7 patients) | No (descriptive analysis) | NR |
|  | Dialysis requirement | During hospitalisation/post-procedure follow-up | One patient with CIN required dialysis | % occurrence | 14.3% (1/7 CIN patients) | No (descriptive analysis) | NR |
|  | Mortality | During hospitalisation/post-procedure follow-up | One patient with CIN died | % occurrence | 14.3% (1/7 CIN patients) | No (descriptive analysis) | NR |
|  | Chronic renal failure progression | Follow-up after CIN development | No patients progressed to chronic renal failure | % occurrence | 0% | No (descriptive analysis) | NR |
| Tao (2017) [8] | CI-AKI occurrence after cerebral angiography | Within 48 h after contrast exposure | CI-AKI developed after cerebral angiography/endovascular procedures | % occurrence | 4.2% (85/2015 patients) | No (descriptive cohort analysis) | NR |
|  | RRT requirement | During hospitalisation | Five patients with CI-AKI required RRT | % occurrence | 5/85 CI-AKI patients (5.9%) | No (descriptive analysis) | NR |
|  | Proteinuria and in-hospital mortality | During hospitalisation | Proteinuria not independently associated with increased in-hospital mortality | OR | 1.25 (0.49–3.17) | Yes (multivariable logistic regression) | No |
|  | One-year mortality | 1-year follow-up | Proteinuria independently associated with increased 1-year mortality | HR | 2.30 (1.55–3.41) | Yes (multivariable Cox proportional hazards model) | Yes |
|  | One-year mortality in patients with proteinuria | 1-year follow-up | Higher 1-year mortality observed in patients with proteinuria | % mortality | 41.5% vs. 18.7% | No (Kaplan–Meier analysis) | Yes |
|  | One-year mortality and NIHSS score | 1-year follow-up | Higher NIHSS score independently associated with increased 1-year mortality | HR | 1.02 (1.00–1.04) | Yes (multivariable Cox proportional hazards model) | Yes |
| Xu (2025) [7] | CA-AKI occurrence after sequential CTA and BAE | 24–72 h after BAE | CA-AKI developed following sequential CTA and emergency BAE | % occurrence | 7.6% (13/171 patients) | No (descriptive cohort analysis) | NR |
|  | RRT requirement | During hospitalisation/post-procedure follow-up | No patients with CA-AKI required RRT | % occurrence | 0% | No (descriptive analysis) | NR |
|  | Renal recovery | Several days after BAE | Patients with CA-AKI recovered renal function within several days | Clinical course | Recovery observed without permanent renal injury | No (descriptive analysis) | NR |
|  | Irreversible postoperative renal deterioration | Post-procedure follow-up | No irreversible postoperative deterioration of kidney function observed | % occurrence | 0% irreversible renal injury reported | No (descriptive analysis) | NR |
| Yoo (2020) [6] | RRT requirement | During hospitalisation | Five patients with AKI required RRT after EVT | % occurrence | 5/59 AKI patients (8.5%); 0.8% of total cohort | No (descriptive analysis) | NR |
|  | Poor functional outcome (mRS ≥3) | 3-month follow-up | Presence of AKI independently associated with poor functional outcome | OR | 5.14 (2.17–13.85) | Yes (multivariable logistic regression) | Yes |
|  | Poor functional outcome in AKI stage 2 | 3-month follow-up | AKI stage 2 independently associated with poor functional outcome | OR | 13.70 (2.10–280.18) | Yes (multivariable logistic regression) | Yes |
|  | Poor functional outcome in AKI stage 3 | 3-month follow-up | AKI stage 3 independently associated with poor functional outcome | OR | 6.02 (1.45–42.59) | Yes (multivariable logistic regression) | Yes |
|  | Poor functional outcome rate | 3-month follow-up | Poor outcomes more frequent among AKI patients | % occurrence | 88.1% vs. 51.3% | No (group comparison) | Yes |
|  | Mortality at 3 months | 3-month follow-up | Presence of AKI independently associated with mortality | OR | 8.16 (4.04–16.70) | Yes (multivariable logistic regression) | Yes |
|  | Mortality in AKI stage 2 | 3-month follow-up | AKI stage 2 independently associated with mortality | OR | 20.84 (5.90–82.05) | Yes (multivariable logistic regression) | Yes |
|  | Mortality in AKI stage 3 | 3-month follow-up | AKI stage 3 independently associated with mortality | OR | 13.67 (4.74–41.92) | Yes (multivariable logistic regression) | Yes |
|  | Mortality rate at 3 months | 3-month follow-up | Mortality substantially higher among AKI patients | % mortality | 49.2% vs. 10.5% | No (group comparison) | Yes |
|  | Any haemorrhagic transformation | During hospitalisation | Haemorrhagic transformation more common in AKI patients | % occurrence | 55.9% vs. 30.8% | No (group comparison) | Yes |
|  | Parenchymal hematoma | During hospitalisation | Parenchymal hematoma more frequent in AKI patients | % occurrence | 27.1% vs. 13.0% | No (group comparison) | Yes |
|  | Parenchymal hematoma type 2 and poor outcome | 3-month follow-up | Parenchymal hematoma type 2 independently associated with poor functional outcome | OR | 4.43 (1.79–12.87) | Yes (multivariable logistic regression) | Yes |
|  | Parenchymal hematoma type 2 and mortality | 3-month follow-up | Parenchymal hematoma type 2 independently associated with mortality | OR | 5.17 (2.45–10.83) | Yes (multivariable logistic regression) | Yes |

Abbreviations: ¥, Chinese yuan; AKI, acute kidney injury; AKIN, Acute Kidney Injury Network; ARF, acute renal failure; AVM, arteriovenous malformation; BAE, bronchial artery embolization; BCLC, Barcelona Clinic Liver Cancer staging system; CA-AKI, contrast-associated acute kidney injury; CI, confidence interval; CI-AKI, contrast-induced acute kidney injury; CIN, contrast-induced nephropathy; CKD, chronic kidney disease; CRRT, continuous renal replacement therapy; CT, computed tomography; CTA, computed tomography angiography; cTACE, conventional transarterial chemoembolization; CVVH, continuous veno-venous hemofiltration; DEMs, drug-eluting microspheres; DSA, digital subtraction angiography; eGFR, estimated glomerular filtration rate; EVT, endovascular thrombectomy; GCS, Glasgow Coma Scale; HAE, hepatic artery embolization; HCC, hepatocellular carcinoma; HR, hazard ratio; MACD, maximum allowable contrast dose; mRS, modified Rankin Scale; NGAL, neutrophil gelatinase-associated lipocalin; NIHSS, National Institutes of Health Stroke Scale; NR, not reported; OR, odds ratio; PC-AKI, post-contrast acute kidney injury; POD, postoperative day; PSM, propensity score matching; RCIN, radiocontrast-induced nephropathy; ROC, receiver operating characteristic; RR, relative risk; RRT, renal replacement therapy; SAH, subarachnoid haemorrhage; TACE, transcatheter arterial chemoembolization; TAE, transcatheter arterial embolization; TAI, transarterial infusion chemotherapy; tPA, tissue plasminogen activator.

Note: Clinical outcomes and effect estimates were extracted as reported by the original studies and may include descriptive analyses, group comparisons, survival analyses, or multivariable regression models. Adjusted analyses refer to multivariable models controlling for potential confounding variables. Outcome timeframes were defined according to individual study follow-up periods.

Supplementary Table S6. Risk of bias assessment of included studies using the Newcastle–Ottawa Scale (NOS).

| Study | Selection (0–4) | Comparability (0–2) | Outcome (0–3) | Total Score (0–9) | Risk of Bias | Main reasons scores were reduced |
| --- | --- | --- | --- | --- | --- | --- |
| Aoe (2019) [43] | 2 | 1 | 2 | 5 | Moderate | Retrospective single-centre study with substantial exclusion because post-TACE renal measurements were unavailable; incomplete assessment of potentially important confounders; non-KDIGO CIN definition. |
| Cao (2021) [42] | 3 | 1 | 2 | 6 | Moderate | Retrospective design and selected clinical population; limited control of potential confounding; outcome ascertainment dependent on available post-procedural renal measurements. |
| Cho (2011) [41] | 3 | 2 | 2 | 7 | Low | Retrospective single-centre design and selected TACE population; otherwise clearly defined renal outcome and multivariable assessment of relevant risk factors. |
| Cohen (2014) [40] | 2 | 1 | 2 | 5 | Moderate | Heterogeneous retrospective datasets; AKI information available only for a subset of the overall cohort; no formal creatinine-based AKI definition reported; limited ascertainment of renal adverse events. |
| Delgado Acosta (2020) [39] | 3 | 2 | 2 | 7 | Low | Retrospective single-centre cohort; otherwise consecutive inclusion, clearly defined CIN outcome and assessment of relevant clinical predictors. |
| Diprose (2019) [38] | 3 | 2 | 3 | 8 | Low | Prospective registry-based cohort with standardized renal outcome ascertainment and appropriate adjustment; minor selection concerns related to availability of renal measurements. |
| Hao (2014) [37] | 3 | 2 | 2 | 7 | Low | Prospective cohort with clearly defined AKI ascertainment, but repeated procedures were analyzed and the study population was relatively selected. |
| Hayakawa (2014) [36] | 3 | 2 | 2 | 7 | Low | Retrospective design; predominantly procedure/session-level analysis; otherwise consecutive procedural inclusion with relatively complete renal-function ascertainment and adjusted analysis. |
| Hayashi (2020) [35] | 3 | 2 | 2 | 7 | Low | Retrospective single-centre study and selected procedural population; appropriate objective renal assessment and adjustment for relevant clinical variables. |
| Hsu (2010) [34] | 2 | 2 | 2 | 6 | Moderate | Selected HCC population with advanced liver disease/ascites; retrospective design; potential overlap with other reports from the same research group could not be excluded. |
| Huo (2004) [33] | 3 | 2 | 2 | 7 | Low | Prospective study with scheduled renal-function assessment but relatively small, single-centre HCC cohort and potential cohort overlap with related reports. |
| Huo (2004) [32] | 3 | 2 | 2 | 7 | Low | Retrospective single-centre cohort with repeated TACE sessions; GEE appropriately addressed correlated observations, but potential overlap with related reports could not be excluded. |
| Jia (2019) [31] | 3 | 1 | 2 | 6 | Moderate | Prospective data collection but patients lacking post-imaging laboratory measurements were excluded; baseline differences between exposure groups and limited adjustment for confounding. |
| Joo (2019) [30] | 3 | 1 | 2 | 6 | Moderate | Retrospective single-centre cohort; selected population undergoing aneurysm coiling; limited adjustment for potential confounding. |
| Kim (2018) [29] | 3 | 1 | 2 | 6 | Moderate | Retrospective selected CKD/neuroendovascular cohort with relatively small sample and limited ability to adjust for important confounders. |
| Kim (2020) [28] | 3 | 2 | 3 | 8 | Low | Prospective study with prespecified renal measurements and multivariable assessment; generalizability reduced by exclusion of several higher-risk groups, including patients with substantial renal impairment. |
| Laible (2021) [27] | 3 | 2 | 3 | 8 | Low | Prospectively and consecutively recruiting registry with objective renal assessment and near-complete clinical follow-up; minor selection concern from requirement for available renal measurements. |
| Lee (2017) [26] | 4 | 2 | 2 | 8 | Low | Large nationwide population-based cohort with propensity-score matching and extensive covariate adjustment; limitations inherent to retrospective administrative database data and outcome coding. |
| Lee (2018) [25] | 3 | 2 | 2 | 7 | Low | Retrospective single-centre aneurysmal SAH cohort; objective renal outcome and adjusted analysis, but selected procedural population and retrospective ascertainment. |
| Lin (2025) [24] | 3 | 1 | 3 | 7 | Low | Consecutive first-TACE cohort with detailed serial renal measurements; single-centre selected population and comparatively limited confounder adjustment. |
| Mitaka (2023) [23] | 3 | 2 | 2 | 7 | Low | Structured renal-function assessment and multivariable analysis, but single-centre population and very small number of clinical CA-AKI events limited precision of risk-factor estimates. |
| Murakami (2016) [22] | 2 | 2 | 2 | 6 | Moderate | Retrospective single-centre study of 186 TACE sessions in 122 patients; repeated observations and small sample reduce representativeness and precision despite multivariable analysis. |
| Myung (2020) [20] | 2 | 2 | 2 | 6 | Moderate | Retrospective cohort with exclusions related to early death, discharge/transfer, or unavailable postcontrast creatinine; potential selection bias despite multivariable adjustment. |
| Ösken (2021) [21] | 3 | 1 | 2 | 6 | Moderate | Retrospective single-centre cohort; incomplete information on potentially important hemodynamic and procedural confounders; otherwise objective renal outcome assessment. |
| Ozdogru (2025) [19] | 3 | 1 | 2 | 6 | Moderate | Retrospective single-centre study; exact contrast volume was unavailable and estimated indirectly; residual procedural and clinical confounding remained possible. |
| Park (2008) [18] | 3 | 2 | 2 | 7 | Low | Retrospective single-centre cohort restricted to sessions with available renal measurements; urine-output criteria were unavailable and creatinine follow-up was limited to 2 days in a substantial proportion of sessions. |
| Prasad (2014) [17] | 3 | 1 | 2 | 6 | Moderate | Retrospective study; exclusion for unavailable postprocedural creatinine; 48-h creatinine unavailable in 15%; only four CIN events precluded meaningful independent risk-factor analysis. |
| Ray (2013) [16] | 3 | 1 | 2 | 6 | Moderate | Prospectively collected database but retrospective clinical-record analysis; small single-centre cohort, missing baseline variables, and inadequate statistical power for robust risk-factor assessment. |
| Schwarz (2026) [15] | 2 | 2 | 2 | 6 | Moderate | Large multicentre consecutive cohort with strong adjustment, but substantial exclusions, including patients without requisite creatinine or 90-day outcome data; nonuniform timing of creatinine measurements and potential selection bias. |
| Seki (2021) [14] | 2 | 0 | 1 | 3 | High | Small retrospective single-centre cohort; substantial missing renal measurements; only a subset had creatinine assessed within the prespecified 48–72-h window; no meaningful multivariable confounder adjustment. |
| Sharma (2013) [13] | 3 | 0 | 2 | 5 | Moderate | Retrospective cohort; only three CIN events, preventing meaningful adjusted risk-factor analysis; several patients died without postprocedure creatinine measurement; no long-term renal follow-up. |
| Si (2021) [12] | 3 | 2 | 2 | 7 | Low | Retrospective analysis of 503 TACE sessions in 284 patients; selected population excluding renal insufficiency and other high-risk conditions; PSM and multivariable analysis strengthened comparability. |
| Sohn (2020) [11] | 2 | 2 | 2 | 6 | Moderate | Retrospective selected HCC cohort with substantial exclusions; restricted baseline renal/liver characteristics; AKI ascertainment extending over several months creates uncertainty in temporal attribution despite multivariable analysis. |
| Soliman (2020) [10] | 2 | 1 | 2 | 5 | Moderate | Retrospective single-centre study; only 84/162 initially identified patients entered the final analysis; CKD patients were excluded; small sample and event count limited multivariable control of confounding. |
| Song (2013) [9] | 3 | 1 | 2 | 6 | Moderate | Retrospective two-centre study with only 88 participants and seven CIN events; exclusions for missing renal measurements/severe renal disease and limited multivariable precision. |
| Tao (2017) [8] | 3 | 2 | 3 | 8 | Low | Large consecutive cohort with standardized CI-AKI definition and extensive multivariable adjustment; selection bias remains possible because patients lacking renal/urine measurements or 1-year follow-up and several high-risk clinical groups were excluded. |
| Xu (2025) [7] | 2 | 2 | 1 | 5 | Moderate | Large proportion of potentially eligible patients excluded, including those lacking postprocedural creatinine and those who died/transferred; authors acknowledged potential underestimation of CA-AKI; retrospective single-centre design. |
| Yoo (2020) [6] | 2 | 2 | 2 | 6 | Moderate | Multicentre registry with adjusted analysis, but patients without renal measurements were excluded and differed systematically from included patients; authors acknowledged potential overestimation of AKI. |
| Zhou (2014) [5] | 2 | 2 | 1 | 5 | Moderate | More than 2,000 TACE procedures were performed during the study period but only 453 cases with complete required records were analyzed, creating substantial selection/missing-data concern; repeated procedural observations were included. |

NOS domain scores: Selection, 0–4; Comparability, 0–2; Outcome, 0–3; total score, 0–9. For interpretive purposes, total scores of 7–9 were classified as low risk of bias, 4–6 as moderate risk of bias, and 0–3 as high risk of bias. Assessments were based on information reported in the original publications; where a methodological criterion was insufficiently reported or could not be established, the corresponding domain score was reduced.

Supplementary Table S7. Additional subgroup, severity-specific, and timepoint-specific incidence findings of contrast-associated acute kidney injury across included studies.

| Study (Author, Year) | Subgroup/Category | Analytic Sample Size / Unit of Analysis | CA-AKI Cases (n) | Incidence (%) | Definition of CA-AKI | Timing of AKI Assessment |
| --- | --- | --- | --- | --- | --- | --- |
| Hao (2014) [37] | Alternative diagnostic definition | 316 TACE procedures in 166 patients | 69 | 21.8% | Barrett and Parfrey criteria: ≥25% increase in serum creatinine from baseline | Within 72 h after TACE |
| Hayashi (2020) [35] | Patient-level incidence | 129 patients | 14 | 10.8% | ICA AKI criteria | Within 7 days after TACE/TAI |
| Huo (2004) [32] | Treatment-session incidence | 843 TACE treatment sessions | 29 | 6.6% | Same ARF definition | Within 7 days after each TACE session |
| Jia (2019) [31] | Imaging strategy subgroup | CTA-only group: 87 patients | 2 | 2.3% | Same AKI definition | 48 h after CTA |
|  | Imaging strategy subgroup | CTA + DSA/endovascular treatment group: 94 patients | 7 | 7.4% | Same AKI definition | 48 h after CTA |
| Joo (2019) [30] | AKI severity stage | 230 patients | 11 | 4.8% | Grade 1 CIN: ≥25% increase in serum creatinine from baseline with <0.5 mg/dL absolute increase | 48–72 h after contrast exposure |
|  | AKI severity stage | 230 patients | 2 | 0.9% | Grade 2 CIN: serum creatinine increase ≥0.5 mg/dL above baseline | 48–72 h after contrast exposure |
| Kim (2018) [29] | Procedure type subgroup | 140 diagnostic cerebral angiography procedures | 0 | 0% | Same CIN definition | 48–72 h after contrast exposure |
|  | Procedure type subgroup | 44 neuroendovascular therapeutic interventions | 1 | 2.3% | Same CIN definition | 48–72 h after contrast exposure |
|  | Baseline renal function subgroup | Stage 3 CKD patients (154 patients) | 0 | 0% | Same CIN definition | 48–72 h after contrast exposure |
|  | Baseline renal function subgroup | Stage 4 CKD patients (24 patients) | 0 | 0% | Same CIN definition | 48–72 h after contrast exposure |
|  | Baseline renal function subgroup | Stage 5 CKD patients (6 patients) | 1 | 16.7% | Same CIN definition | 48–72 h after contrast exposure |
| Laible (2021) [27] | Baseline renal function subgroup | Patients with baseline renal impairment (379 patients) | 3 | 0.8% | Same PC-AKI definition | Within 48 h after EVT |
|  | Baseline renal function subgroup | Patients without baseline renal impairment (790 patients) | 26 | 3.3% | Same PC-AKI definition | Within 48 h after EVT |
| Lee (2017) [26] | Treatment frequency subgroup | HCC patients with cTACE ≤3 sessions (981 patients) | 65 | 6.6% | Same ICD-9-CM AKI definition | During longitudinal follow-up |
|  | Treatment frequency subgroup | HCC patients with cTACE >3 sessions (151 patients) | 7 | 4.6% | Same ICD-9-CM AKI definition | During longitudinal follow-up |
| Lee (2018) [25] | Prior imaging exposure subgroup | Patients with prior contrast-enhanced imaging within 72 h before embolization | 14 | 7.9% | Same CI-AKI definition | Within 72 h after embolization |
|  | Prior imaging exposure subgroup | Patients without prior contrast-enhanced imaging before embolization | 0 | 0% | Same CI-AKI definition | Within 72 h after embolization |
| Lin (2025) [24] | Non-standard renal outcome | 265 patients undergoing first-session TACE | 75 | 28.3% | Renal-function exacerbation defined by worsening CG-GFR at discharge compared with admission | Serial assessment from admission through discharge |
|  | Baseline renal function subgroup | Patients with CKD (108 patients) | 26 | 24.1% | Same renal-function deterioration definition | At discharge after TACE |
|  | Baseline renal function subgroup | Patients without CKD (157 patients) | 49 | 31.2% | Same renal-function deterioration definition | At discharge after TACE |
|  | Timepoint-specific analysis | 261 patients with creatinine assessment on day 3 after TACE | 86 | 33.0% | Increase in serum creatinine at day 3 compared with admission | Third day after TACE |
|  | Baseline renal function subgroup | Patients with CKD assessed on day 3 (106 patients) | 29 | 27.4% | Same creatinine-increase definition | Third day after TACE |
|  | Baseline renal function subgroup | Patients without CKD assessed on day 3 (155 patients) | 57 | 36.8% | Same creatinine-increase definition | Third day after TACE |
| Mitaka (2023) [23] | Subclinical AKI definition | 67 patients with urine NGAL measurements | 7 | 10.4% | Subclinical CA-AKI defined as urine NGAL/creatinine ratio ≥55.2 μg/g·Cr on POD 0 and/or POD 1 | POD 0 and POD 1 |
|  | Procedure subgroup | Patients undergoing surgery for CAS (22 patients) | 4 | 18.2% | Combined clinical/subclinical CA-AKI criteria | POD 0–3 |
| Murakami (2016) [22] | Liver disease severity subgroup | Child–Pugh class B/C TACE sessions (80 sessions) | 10 | 12.5% | Same CIN definition | 48–72 h after TACE |
|  | Liver disease severity subgroup | Child–Pugh class A TACE sessions (106 sessions) | 4 | 3.8% | Same CIN definition | 48–72 h after TACE |
| Myung (2020) [20] | Baseline renal function subgroup | Patients with baseline eGFR <30 mL/min/1.73 m² (15 patients) | 7 | 46.7% | Same PC-AKI definition | Within 72 h after contrast exposure |
|  | Baseline renal function subgroup | Patients with baseline eGFR 30–59 mL/min/1.73 m² (97 patients) | 6 | 6.2% | Same PC-AKI definition | Within 72 h after contrast exposure |
|  | Baseline renal function subgroup | Patients with baseline eGFR 60–89 mL/min/1.73 m² (302 patients) | 33 | 10.9% | Same PC-AKI definition | Within 72 h after contrast exposure |
|  | Baseline renal function subgroup | Patients with baseline eGFR ≥90 mL/min/1.73 m² (187 patients) | 11 | 5.9% | Same PC-AKI definition | Within 72 h after contrast exposure |
| Park (2008) [18] | Treatment-session incidence | 442 TACE treatment sessions in 236 patients | 29 | 6.6% | AKI defined according to AKIN criteria as absolute increase in serum creatinine ≥0.3 mg/dL or ≥50% from baseline within 48 h after TACE | Serum creatinine assessed daily for at least 2 days after TACE |
|  | Timepoint-specific analysis | 236 patients undergoing TACE | 24 | 10.2% | Same AKIN definition | First 48 h after TACE |
|  | AKI severity stage | 29 AKI episodes | 26 | 89.7% | AKIN stage 1: increase ≥0.3 mg/dL or 1.5–2× baseline creatinine | During hospitalisation |
|  | AKI severity stage | 29 AKI episodes | 2 | 6.9% | AKIN stage 2: increase >2–3× baseline creatinine | During hospitalisation |
|  | AKI severity stage | 29 AKI episodes | 1 | 3.4% | AKIN stage 3: increase >3× baseline creatinine | During hospitalisation |
| Prasad (2014) [17] | Contrast volume subgroup | High-dose contrast media subgroup: 250–299 mL (36 procedures) | 1 | 2.8% | Same CIN definition | 24–48 h after procedure |
|  | Contrast volume subgroup | High-dose contrast media subgroup: 300–399 mL (29 procedures) | 2 | 6.9% | Same CIN definition | 24–48 h after procedure |
|  | Contrast volume subgroup | High-dose contrast media subgroup: 400–499 mL (9 procedures) | 1 | 11.1% | Same CIN definition | 24–48 h after procedure |
|  | Contrast volume subgroup | High-dose contrast media subgroup: >500 mL (5 procedures) | 0 | 0% | Same CIN definition | 24–48 h after procedure |
|  | Comparator subgroup | Control cohort: 75–249 mL contrast media (79 procedures) | 0 | 0% | Same CIN definition | 24–48 h after procedure |
|  | Timepoint-specific analysis | CIN detected at 24 h | 3 | 3.8% | Same CIN definition | 24 h after procedure |
|  | Timepoint-specific analysis | CIN detected at 48 h | 1 | 1.3% | Same CIN definition | 48 h after procedure |
| Ray (2013) [16] | Repeated contrast exposure subgroup | Patients undergoing additional contrast-enhanced studies within 72 h (49 patients) | 0 | 0% | Same KDIGO CIN definition | Within 72 h after repeated contrast exposure |
| Schwarz (2026) [15] | Contrast volume subgroup | Patients receiving intra-procedural contrast volume >90th percentile (205 patients) | 20 | 9.8% | Same KDIGO CA-AKI definition | Within 48 h after EVT |
| Seki (2021) [14] | Repeat procedure subgroup | Patients undergoing repeat TAE procedures (4 patients) | 0 | 0% | Same ESUR PC-AKI definition | Follow-up after repeat embolization procedures |
| Sharma (2013) [13] | Baseline renal function subgroup | Patients with baseline creatinine ≤1.5 mg/dL (163 patients) | 2 | 1.2% | Same AKIN CIN definition | Within 48 h after procedure |
|  | Baseline renal function subgroup | Patients with baseline creatinine >1.5 mg/dL (31 patients) | 1 | 3.2% | Same AKIN CIN definition | Within 48 h after procedure |
|  | Additional imaging subgroup | Patients undergoing additional CTA before EVT (44 patients) | 0 | 0% | Same AKIN CIN definition | Within 48 h after procedure |
|  | Dialysis subgroup | Chronic haemodialysis patients (3 patients) | 0 | 0% | Same AKIN CIN definition | Within 48 h after procedure |
| Si (2021) [12] | Haemoglobin subgroup | Low haemoglobin group after PSM (110 patients) | 16 | 14.6% | Same CIN definition | 48–96 h after TACE |
|  | Haemoglobin subgroup | Normal haemoglobin group after PSM (116 patients) | 4 | 3.4% | Same CIN definition | 48–96 h after TACE |
|  | Haemoglobin subgroup | Low haemoglobin group before PSM (110 patients) | 16 | 14.6% | Same CIN definition | 48–96 h after TACE |
|  | Haemoglobin subgroup | Normal haemoglobin group before PSM (393 patients) | 12 | 3.1% | Same CIN definition | 48–96 h after TACE |
| Sohn (2020) [11] | Timepoint-specific analysis | Follow-up timepoint | 7 | 2.0% | Same ICA-AKI definition | 1 day after TACE |
|  | Timepoint-specific analysis | Follow-up timepoint | 6 | 2.0% | Same ICA-AKI definition | 2 months after TACE |
|  | Timepoint-specific analysis | Follow-up timepoint | 27 | 8.0% | Same ICA-AKI definition | 4 months after TACE |
|  | AKI severity stage | AKI stage 1 | 10 | 3.0% | ICA-AKI stage 1: increase in serum creatinine ≥0.3 mg/dL or 1.5–2× baseline | Within 4 months after TACE |
|  | AKI severity stage | AKI stage 2 | 10 | 3.0% | ICA-AKI stage 2: increase in serum creatinine 2–3× baseline | Within 4 months after TACE |
|  | AKI severity stage | AKI stage 3 | 17 | 5.0% | ICA-AKI stage 3: increase in serum creatinine >3× baseline, serum creatinine ≥4.0 mg/dL with acute increase ≥0.3 mg/dL, or initiation of RRT | Within 4 months after TACE |
|  | Comparator subgroup | Non-AKI patients | 310 | 89.0% | No increase in serum creatinine or increase <0.3 mg/dL | Within 4 months after TACE |
| Soliman (2020) [10] | CIN subgroup | Group A (patients developing CIN) | 11 | 10.3% | Same CIN definition | Peak serum creatinine reached median 2 days post-embolization (IQR 1–2.5 days) |
|  | Non-CIN subgroup | Group B (patients without CIN) | 0 | 89.7% | Same CIN definition | Followed within 72 h after embolization |
| Tao (2017) [8] | Proteinuria subgroup | Patients with proteinuria | 12 | 18.5% | Same CI-AKI definition | Within 48 h after cerebral angiography |
|  | Proteinuria subgroup | Patients without proteinuria | 73 | 3.7% | Same CI-AKI definition | Within 48 h after cerebral angiography |
|  | Stroke subtype subgroup | Ischemic stroke patients with proteinuria | NR | 16.7% | Same CI-AKI definition | Within 48 h after cerebral angiography |
|  | Stroke subtype subgroup | Ischemic stroke patients without proteinuria | NR | 4.0% | Same CI-AKI definition | Within 48 h after cerebral angiography |
|  | Stroke subtype subgroup | Haemorrhagic stroke patients with proteinuria | NR | 40.0% | Same CI-AKI definition | Within 48 h after cerebral angiography |
|  | Stroke subtype subgroup | Haemorrhagic stroke patients without proteinuria | NR | 1.1% | Same CI-AKI definition | Within 48 h after cerebral angiography |
| Xu (2025) [7] | Baseline renal function subgroup | Patients with eGFR ≥90 mL/min/1.73 m² | 5 | 5.3% | Same CA-AKI definition | 24–72 h after BAE |
|  | Baseline renal function subgroup | Patients with eGFR 60–89 mL/min/1.73 m² | 3 | 6.0% | Same CA-AKI definition | 24–72 h after BAE |
|  | Baseline renal function subgroup | Patients with eGFR 30–59 mL/min/1.73 m² | 2 | 9.5% | Same CA-AKI definition | 24–72 h after BAE |
|  | Baseline renal function subgroup | Patients with eGFR <30 mL/min/1.73 m² | 3 | 60.0% | Same CA-AKI definition | 24–72 h after BAE |
| Yoo (2020) [6] | AKI severity stage | AKI stage 1 | 22 | 3.7% | KDIGO stage 1 AKI | Within 7 days after EVT |
|  | AKI severity stage | AKI stage 2 | 14 | 2.3% | KDIGO stage 2 AKI | Within 7 days after EVT |
|  | AKI severity stage | AKI stage 3 | 23 | 3.8% | KDIGO stage 3 AKI | Within 7 days after EVT |
|  | Baseline renal function subgroup | Patients with baseline eGFR ≥90 mL/min/1.73 m² | 14 | 14.3% | Same KDIGO AKI definition | Within 7 days after EVT |
|  | Baseline renal function subgroup | Patients with baseline eGFR 60–89 mL/min/1.73 m² | 17 | 8.1% | Same KDIGO AKI definition | Within 7 days after EVT |
|  | Baseline renal function subgroup | Patients with baseline eGFR 30–59 mL/min/1.73 m² | 19 | 7.5% | Same KDIGO AKI definition | Within 7 days after EVT |
|  | Baseline renal function subgroup | Patients with baseline eGFR <30 mL/min/1.73 m² | 9 | 22.5% | Same KDIGO AKI definition | Within 7 days after EVT |
|  | Severe AKI outcome subgroup | Patients requiring RRT | 5 | 0.8% | KDIGO stage 3 AKI requiring RRT | During hospitalisation |
| Zhou (2014) [5] | AKI severity stage | AKIN stage 1 AKI | 13 | 9.8% | AKIN stage 1 criteria | Within 48 h after TACE |
|  | AKI severity stage | AKIN stage 2 AKI | 2 | 1.5% | AKIN stage 2 criteria | Within 48 h after TACE |
|  | AKI severity stage | AKIN stage 3 AKI | 1 | 0.8% | AKIN stage 3 criteria | Within 48 h after TACE |
|  | Liver disease severity subgroup | Patients with Child–Pugh class A | 5 | 6.3% | Same AKIN definition | Within 48 h after TACE |
|  | Liver disease severity subgroup | Patients with Child–Pugh class B | 11 | 20.8% | Same AKIN definition | Within 48 h after TACE |

Abbreviations: AIS, acute ischemic stroke; AKI, acute kidney injury; AKIN, Acute Kidney Injury Network; ARF, acute renal failure; BAE, bronchial artery embolization; CA-AKI, contrast-associated acute kidney injury; CAS, carotid artery stenosis; CG-GFR, Cockcroft–Gault glomerular filtration rate; CI-AKI, contrast-induced acute kidney injury; CIN, contrast-induced nephropathy; CKD, chronic kidney disease; CTA, computed tomography angiography; cTACE, conventional transarterial chemoembolization; eGFR, estimated glomerular filtration rate; ESUR, European Society of Urogenital Radiology; EVT, endovascular thrombectomy; h, hour; HCC, hepatocellular carcinoma; ICA, International Club of Ascites; ICD-9-CM, International Classification of Diseases, Ninth Revision, Clinical Modification; IQR, interquartile range; KDIGO, Kidney Disease: Improving Global Outcomes; NGAL, neutrophil gelatinase-associated lipocalin; PC-AKI, post-contrast acute kidney injury; POD, postoperative day; PSM, propensity score matching; TACE, transcatheter arterial chemoembolization; TAE, transcatheter arterial embolization; TAI, transarterial infusion chemotherapy.

Note: Incidence (%) was extracted or calculated from the original studies and generally represents the proportion of patients, procedures, or treatment sessions complicated by contrast-associated acute kidney injury (CA-AKI), including studies reporting contrast-induced nephropathy (CIN) or acute kidney injury (AKI). Definitions of CA-AKI and timing of assessment were extracted as reported by the original studies.

Supplementary Table S8. GRADE assessment of the certainty of evidence for CA-AKI incidence estimates.

| Outcome / estimate | No. of studies | Study design | Summary estimate (95% CI) | Risk of bias | Inconsistency | Indirectness | Imprecision | Publication bias | Overall certainty of evidence | Rationale |
| --- | --- | --- | --- | --- | --- | --- | --- | --- | --- | --- |
| Overall CA-AKI incidence | 38 | Observational studies | 6.91% (5.69–8.24) | Seriousᵃ | Seriousᵇ | Seriousᶜ | Not seriousᵈ | Suspectedᵉ | Very low | Important methodological limitations were identified across the observational evidence base; heterogeneity was substantial (I² = 89.5%); populations, procedures, CA-AKI definitions, ascertainment windows, and units of analysis varied across studies. The pooled CI was relatively narrow, and sensitivity analyses demonstrated numerical stability of the summary estimate. Funnel-plot assessment and Egger’s regression test identified small-study effects (p = 0.0246); however, given the substantial heterogeneity, the observed asymmetry was not considered definitive evidence of selective publication. |
| CA-AKI incidence: embolization/interventional oncology procedures | 20 | Observational studies | 8.44% (6.53–10.56) | Seriousᵃ | Seriousᵇ | Seriousᶜ | Not seriousᵈ | Not separately assessedᵉ | Very low | Methodological limitations were present across contributing observational studies; substantial residual heterogeneity remained (I² = 84.4%); studies included clinically heterogeneous embolization/interventional oncology populations, with variation in underlying disease, procedure characteristics, CA-AKI definitions, and ascertainment. |
| CA-AKI incidence: neuroendovascular/carotid procedures | 18 | Observational studies | 5.48% (4.05–7.09) | Seriousᵃ | Seriousᵇ | Seriousᶜ | Not seriousᵈ | Not separately assessedᵉ | Very low | Methodological limitations were present across contributing observational studies; substantial residual heterogeneity remained (I² = 90.5%); neuroendovascular/carotid cohorts differed in clinical populations, procedural pathways, contrast exposure, CA-AKI definitions, and ascertainment practices. |
| CA-AKI incidence restricted to patient-level estimates | 30 | Observational studies | 7.27% (5.77–8.92) | Seriousᵃ | Seriousᵇ | Seriousᶜ | Not seriousᵈ | Not separately assessedᵉ | Very low | Restriction to 30 studies representing 16,825 unique patients addressed the mixed unit-of-analysis concern and yielded an estimate similar to the primary analysis; however, substantial heterogeneity persisted (I² = 91.5%), and important methodological and clinical heterogeneity remained. |

Abbreviations: CA-AKI, contrast-associated acute kidney injury; CI, confidence interval; GRADE, Grading of Recommendations Assessment, Development and Evaluation; I², Higgins inconsistency statistic.

ᵃ Risk of bias: Downgraded one level for serious risk of bias. All included studies were observational, and the revised Newcastle–Ottawa Scale assessment identified 14 studies at low risk of bias, 24 at moderate risk, and one at high risk. Frequent limitations included retrospective designs, selection according to availability of post-procedural renal measurements, incomplete follow-up or missing outcome data, limited control of confounding, and repeated procedure/session observations.

ᵇ Inconsistency: Downgraded one level for serious inconsistency because substantial unexplained between-study heterogeneity persisted. Heterogeneity was I² = 89.5% for the overall analysis, 84.4% for embolization/interventional oncology procedures, 90.5% for neuroendovascular/carotid procedures, and 91.5% in the patient-level sensitivity analysis.

ᶜ Indirectness: Downgraded one level for serious indirectness because the evidence encompassed clinically and methodologically diverse populations and procedures, including TACE, neuroendovascular thrombectomy/coiling, carotid interventions, hepatic and bronchial artery embolization, with variation in baseline renal function, underlying disease, contrast exposure, CA-AKI definitions, ascertainment windows, and units of analysis.

ᵈ Imprecision: Not downgraded. The confidence intervals around the overall and principal procedure-specific pooled estimates were considered sufficiently informative for descriptive incidence estimation. The overall estimate was 6.91% (95% CI 5.69–8.24), and leave-one-out estimates remained between 6.56% and 7.16%.

ᵉ Publication bias: Small-study effects were assessed for the primary 38-study incidence meta-analysis using visual inspection of the funnel plot and Egger’s linear regression test. Statistically significant funnel-plot asymmetry was identified (t = 2.35, df = 36, p = 0.0246). Given the substantial between-study heterogeneity (I² = 89.5%) and clinical and methodological diversity across studies, the observed asymmetry may reflect factors other than selective publication and was therefore interpreted cautiously as evidence of small-study effects rather than definitive evidence of publication bias. Publication bias was not separately assessed for the procedure-specific subgroup estimates or the patient-level sensitivity analysis.
